# Mature Red Blood Cells Contain Long DNA Fragments and Could Acquire DNA from Lung Cancer Tissue

**DOI:** 10.1002/advs.202206361

**Published:** 2023-01-04

**Authors:** Naixin Liang, Zichen Jiao, Cong Zhang, Yifan Wu, Tao Wang, Shanqing Li, Yadong Wang, Tianqiang Song, Jian‐Qun Chen, Hongwei Liang, Qihan Chen

**Affiliations:** ^1^ Department of Thoracic Surgery Peking Union Medical College Hospital Chinese Academy of Medical Sciences Beijing 100730 China; ^2^ Department of Thoracic Surgery Nanjing Drum Tower Hospital The Affiliated Hospital of Nanjing University Medical School Nanjing Jiangsu 210093 China; ^3^ The State Key Laboratory of Pharmaceutical Biotechnology School of Life Sciences Nanjing University Nanjing Jiangsu 210023 China; ^4^ School of Life Sciences and Technology China Pharmaceutical University Nanjing Jiangsu 210009 China; ^5^ Medical School of Nanjing University Nanjing Jiangsu 210093 China

**Keywords:** copy number variation, DNA fragments, lung cancer, red blood cell

## Abstract

Red blood cells (RBC) are commonly known as cells with no nucleus or mitochondria and are assumed to be a transportation vehicle. This study confirms that RBC contain long DNA fragments inside with stain by both microscope and flow cytometry, which covers most nuclear and mitochondrial genome regions by next‐generation sequencing (NGS). Such characteristics demonstrate a significant difference compared with A549 cell line or paired peripheral blood mononuclear cell as nucleated cells. To further explore the characteristics of RNA DNA, DNA from 20 RBC samples is sequenced by NGS. Interestingly, several gaps and multiple regions with copy number variation are observed significantly different between different samples, which could be used to distinguish samples with different health status accurately. Using an in vitro co‐culture system, it is shown that RBC could absorb DNA‐bearing tumorigenic mutations from cancer cell lines but requires cell‐to‐cell contact. Finally, based on a small scale clinical trial, it is confirmed that common genetic mutations of cancer tissues could be detected in RBC from patients with early‐stage non‐small‐cell lung cancer. This study highlights a new biological phenomenon involving RBC and its translational potential as a novel liquid biopsy technology platform for early cancer screening and diagnosis of malignancy.

## Graphical abstract

Although there is no nucleus or mitochondria, mature red blood cells (RBC) contain large DNA fragments. RBCs can obtain DNA fragments from tumor cells in vivo and in vitro. This discovery provides a new direction for the diagnosis and mechanism research of tumors and other diseases.

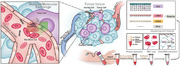

## 1 Introduction

Mammalian red blood cells (RBC) are commonly known as cells with no nucleus or mitochondria^[^ ^1^ ^]^ and are assumed to be a transportation vehicle for oxygen, carbon dioxide, and metabolic by‐products of cells. During the maturation of mammalian erythrocytes, erythroid precursor cells undergo chromatin condensation and nuclear polarization to one side of the cell, eventually extruding the nucleus out of the cell with the help of mitochondria to produce nucleus‐free reticulocytes.^[^ ^2^ ^]^ Reticulocytes begin to mature in the bone marrow and are subsequently released into the circulation to further mature into erythrocytes by clearing mitochondria and other organelles.^[^ ^3^ ^]^ Inclusion bodies with DNA, called Howell–Jolly bodies (HJBs),^[^ ^4^ ^]^ have been found to exist in recycled erythrocytes from certain patients with defective spleen function. The formation of HJBs results from an abnormal process of erythroid development and most HJBs contain DNA of the centromeric region, with only a few HJBs having DNA derived from the euchromatin region.^[^ ^5^ ^]^ Meanwhile, micronucleus DNA was found in mature mammalian erythrocytes, which can be used to evaluate chemicals' clastogenic and aneugenic potential.^[^ ^6^ ^]^ In conclusion, whether mature red blood cells contain DNA and what kind of DNA remains unclear.

Recent studies have revealed that RBC has other functions as the most abundant cell type in the circulatory system. For example, RBC regulates the function of body immunity by binding cell‐free mtDNA, and pathogenic DNA through Toll‐like receptors 9 (TLR9)expressed on the RBC surface.^[^ ^7^ ^]^ In addition, RBC was also reported to interact with cancer cells through galectin‐4 and play an essential role in tumor progression and metastasis.^[^ ^8^ ^]^ On the other hand, scientists developed multiple platforms to use RBC as drug delivery carriers based on their material exchange ability.^[^ ^9^ ^]^ Meanwhile, numerous studies have found the presence of cell‐surface‐bound DNA (csbDNA)on the outer membrane of erythrocytes,^[^ ^10^ ^]^ but whether RBC from the external environment captures these DNA remains unknown. Together, RBC demonstrated various functions and potential applications than we knew previously.^[^ ^11^ ^]^

In 2011, Nilsson et al. discovered that platelets in the circulatory system could receive membrane vesicles secreted by cancer cells carrying tumor RNA.^[^ ^12^ ^]^ Later on, platelets were reported to be educated by cancer cells and contain RNA patterns that can be used as a diagnostic marker.^[^ ^13^ ^]^ Given the higher abundance of RBC in the blood circulation system, whether RBC also contains cargos derived from cancer cells is also worth investigating. In addition, as the primary cells participate in the transportation of oxygen and nutrients, red blood cells play an essential role in the occurrence and development of cancer.^[^ ^14^ ^]^ Therefore, this study aimed to confirm that RBC contained DNA and whether the DNA was endogenous or exogenous. In addition, we want to explore and evaluate the potential application of RBC DNA in diagnosis.

## 2 Results

### 2.1 Mature RBC Contained Long DNA Fragments with Special Characteristic

Our study started from whether mature RBC contained DNA or not. In order to ensure the accuracy of the results, the most important point was that the DNA we studied really came from RBC rather than other blood cells. To answer this question, RBC was separated from fresh whole blood collected from healthy donor according to the steps described in Section 4 (Figure S1A, Supporting Information). The purity of separated RBC was confirmed by microscopy and flow cytometry with CD235a(+) and CD45(−)^[^ ^15^ ^]^ (Figure S1B,C, Supporting Information). To further ensure the purity of separated RBC, we used flow cytometry to screen potential monocyte (CD11B as marker), T cell (CD3 as marker), white blood cell (CD45 as marker), and none of them showed positive results (Figure S1D, Supporting Information).

**Figure 1:**
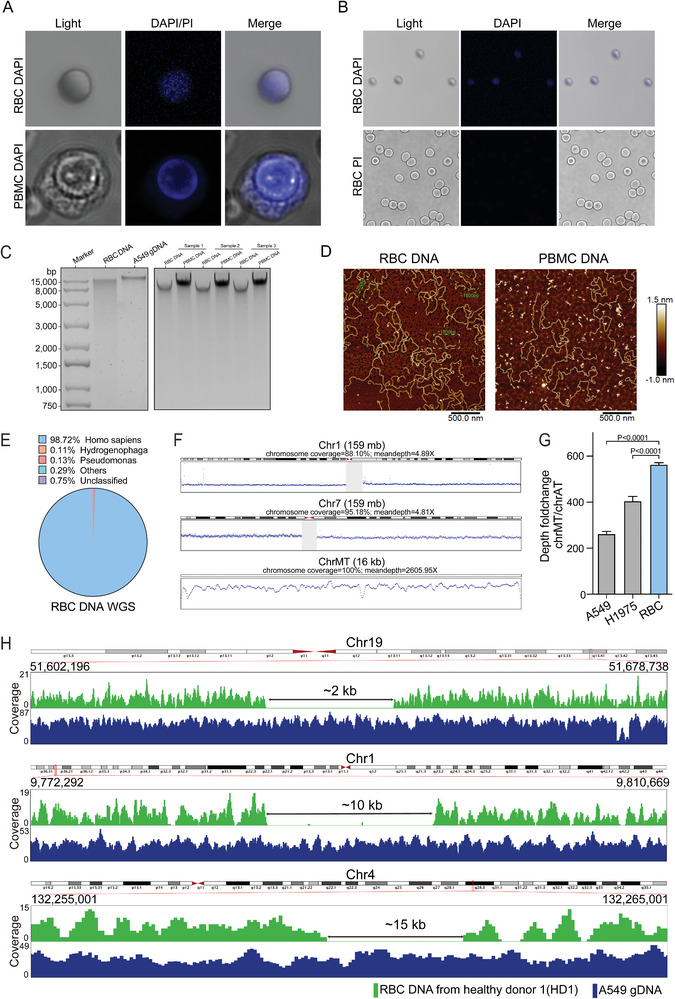
The properties and characteristics of red blood cells DNA. A) Confocal image of DAPI stained RBC (top) and PBMC (bottom) were shown by light/DAPI fluorescence/merge images. B) Confocal image of DAPI (top) and PI (bottom) stained RBC and were shown by light/DAPI or PI fluorescence/merge images. C) Gel electrophoresis analysis and comparison of RBC DNA with nucleated cell DNA (A549 cells and PBMC). D) RBC DNA and PBMC DNA scanned with AFM. Representative medium‐length RBC DNA is boxed with green dashed lines, and the approximate lengths are marked based on image. E) Analysis of the composition and origin of RBC DNA based on next‐generation sequencing data. F) An illustration shows the representative mapping results of RBC DNA whole genome sequencing data. DNA fragments from RBC covered most part of nuclear and mitochondrial genomes. Gray areas represented centromeres. G) Sequencing depth ratio of mitochondrial DNA to genomic chromosomes based on whole genome sequencing of RBC DNA. A549 and H1975 sequencing data from public database (A549 from ENCODE ENCSR521ELB). The bar graph shows mean ± SEM of sequencing depth ratio of mitochondrial DNA to 22 chromosomes. ***P < 0.001, unpaired *t*‐test. H) The mapping results of RBC DNA sequencing data in representative discontinuous regions. RBC DNA exhibits multiple gap regions as large as several kilobases compared to the continuous A549 genomic DNA.

To explore whether purified mature RBC contained DNA, we used DAPI to stain the cells and analyzed by both microscope and flow cytometry. To our surprise, flow cytometry analysis revealed significant fluorescent signal occurred in RBC after staining, indicating the existence of DNA in RBC (Figure S2A, Supporting Information). Meanwhile, confocal microscopy of DAPI‐stained RBC showed that DNA filled with the whole RBC without nucleus structure, which was quite different with nucleated cells that DNA clustered in the nucleus (**Figure** **1**A and Figure S2B, Supporting Information). Since several studies demonstrated DNA fragments could adhere to the surface of cells as csbDNA, we want to further confirm whether DNA we observed was inside or outside RBC.^[^ ^10a^ ^]^ Both DAPI and PI were used to stain the separated RBC, but only DAPI showed obvious positive result (Figure 1B). Considering PI could not pass through the membrane of cell, such results suggested the DNA we observed was at least mainly inside RBC but not csbDNA.

To better understand RBC DNA, the DNA was extracted from RBC by adding lysis buffer as described in Experimental Section. Although we believe the separated RBC was quite pure, such step was well designed to avoid any potential interference of nucleated cells, which would remain complete in lysis buffer and eliminated in the following step. The DNA from A549, three RBC samples, and their paired peripheral blood mononuclear cell (PBMC) were then separated on agarose gel. The size of all three RBC DNA presented the main band larger than the upper limit of DNA ladder 15 000 bps but significantly smaller than extracted DNA from A549 and their paired PBMC (Figure 1C). The Agilent Bioanalyzer 2100 showed that DNA fragments were larger than 1000 bps, and most of them were out of the detection range 10 380 bps (Figure S1E, Supporting Information). Notably, no DNA was detected within the 170–340 bps range, which is usually considered the main size range of cfDNA^[^ ^16^ ^]^ (Figure S1E, Supporting Information). Recently, Atomic Force Microscope (AFM) was used in the field of biology to observe materials at nanometer scale.^[^ ^17^ ^]^ Here we tried to use AFM to further understand RBC DNA. Consistent with the results of the previous two assays, multiple DNA fragments with middle length from thousands bps were observed in DNA from RBC but not PBMC (Figure 1D).

Next step, we used next‐generation sequencing to acquire sequence information of those DNA fragments. Although it was reported that RBC could bind bacterial DNA fragments,^[^ ^7b^ ^]^ here more than 98.72% of the DNA fragment originated from human genome (Figure 1E). To quantify the covering regions and coverage accurately, we added unique Molecular Identifiers (UMI) during the library preparation step to adjust the potential bias in the sequencing step. After mapping to the human genome, DNA fragments covered most regions of both nuclear (average 90.02%) and mitochondrial genome (100%) with mean depths of ≈5× and 2605× (Figure 1F). To our surprise, the depth ratio between mitochondrial/nuclear was ≈500‐fold‐change, which was usually 200–300× in normal nucleated cells and much higher than 265× of A549 and 408× of H1975 (Figure 1G). Since this result represented DNA from a large population of RBC, we were curious about the DNA each RBC contains. Since it was hard to study DNA of single RBC, we diluted the separated RBC sample to approximate 100 000, 10 000, 1000, 100, and 10 RBC cells per µL, and collected 1 µL of each to three groups (Figure S3A, Supporting Information). The cells were then collected and lysed, *GAPDH* (representor of genome) and *CO1* (representor of mitochondria) were quantified by qPCR. The results demonstrated that DNA was unevenly distributed among RBC, and this phenomenon becomes more obvious with decreased number of RBC (Figure S3B, Supporting Information). On the other hand, even the RBC DNA was from more than 10^8 cells, the fragments still remained some large gaps where A549 demonstrated continues distribution. Here we illustrated three regions as examples, while more large gaps can be found in every chromosome (Figure 1H).

### 2.2 Characteristics of DNA Fragments in Multiple Samples

Since platelets were reported to be “educated” by cancer cells in previous studies,^[^ ^13^ ^]^ we wondered if RBC DNA presented unique information under different health status. Therefore, we collected 20 blood samples from Nanjing Drum Tower Hospital (detailed information of patients can be seen in Table S1, Supporting Information) and performed the same analysis as above, including three healthy donors, six patients with small benign lung nodules, and 11 patients with lung cancers. To explore whether the DNA purification method would affect our discovery, we used magnetic beads as well as column to purify the DNA from LC10 patient as described in Section 4, which was labeled as “LC10*” in the following study.

**Figure 2:**
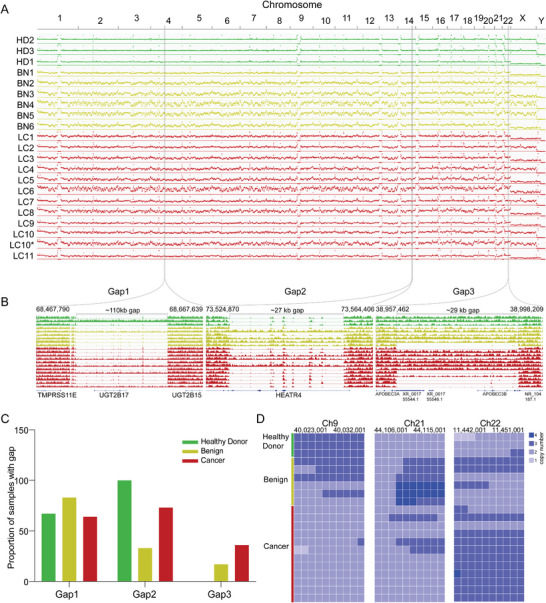
Analysis of the properties and fragment characteristics of RBC DNA from healthy donors and patients with malignant tumors or benign nodules. A) An illustration shows the representative mapping results of RBC DNA whole genome sequencing data of 3 healthy donors, 6 patients with benign nodules, and 11 patients with lung cancer. LC10* was DNA from the sample patient as LC10 but with magnetic beads extraction as described in section 4. B) Three representative discontinuous regions (gap region) of the RBC DNA. C) Proportion of samples’ RBC DNA with gap in the corresponding groups. D) The copy number variation within and between samples in each 1 kb windows in three representative regions. The sample order in the vertical column is consistent with (A). HD: healthy donor; BN: benign; LC: lung cancer.

Interestingly, RBC DNA from healthy donors demonstrated quite equal distribution among the whole genome, while most RBC DNA from patients with benign nodule or cancer obvious copy number variation (**Figure** **2**A). Gaps were observed as well in multiple regions among different samples (Figure 2B,C). For example, A 110 kb gap containing gene *UGT2B17* were observed in more than half samples in all groups; A 27 kb gap containing most exons of gene *HEATR4* were observed in all three samples from healthy donor group, most samples from cancer group, but only 1/3 samples from benign nodule group; A 29 kb gap containing gene *APOBEC3B* were observed in some samples from benign nodule and cancer group, but not in healthy donors.

On the other hand, we tried to better compare the CNV (copy number variation) within and between samples quantitatively. Based on the analysis methods developed and used by Gabrielaite, M. in the previous study,^[^ ^18^ ^]^ we divided the genome into multiple 1 kb windows and converted the average CNV of each window to 0–5. Although most windows demonstrated “2” as diploid, many windows showed obvious variation but somehow similar patterns in samples from the same groups. For example, windows from 40 023 001 on Chr9, 44 106 001 on Chr21, and 11 442 001 on Chr 22 demonstrated higher copy number in healthy donor group, benign nodule group, and lung cancer group comparing with nearby windows and same windows of other samples (Figure 2D).

### 2.3 RBC DNA CNV Was a Potential Direction to Distinguish Early Stage Lung Cancer Patients

**Figure 3:**
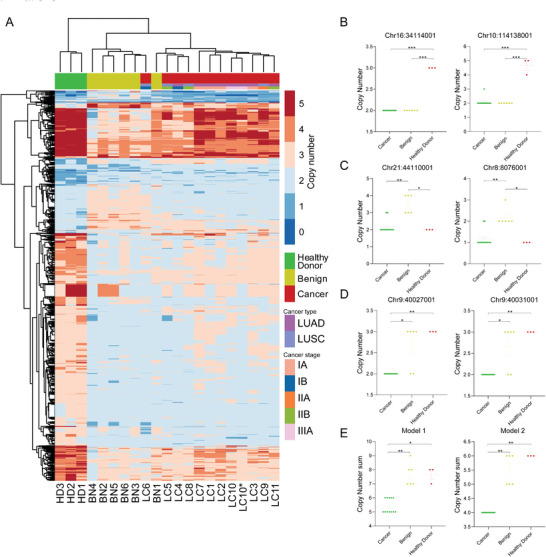
DNA copy number distribution characteristics of RBC DNA from cancer patients, patients with benign nodules, and healthy donors. A) The clustering of samples based on the copy number variations of 4202 selected 1 kb windows. B–D) The average copy number of the selected RBC DNA regions (1kp) can be used to distinguish healthy donors (B), patients with benign nodules (C), and patients with lung cancer (D). The score of the two combination models to screen patients with early stage lung cancer. Each model included three windows and the score was calculated by the sum of their copy numbers. Model1 included Chr8:8076001, Chr10:114138001, and Chr21:44110,00. Model2 included Chr9:40027001, Chr12:8417001, and Chr16:34114001. Mean ± SEM. are shown; ***P < 0.001, **P = 0.001–0.01, *P = 0.01–0.05, Kruskal–Wallis test and Dunn's multiple comparisons test were used to compare copy number for each genomic region. HD: healthy donor; BN: benign; LC: lung cancer.

Since RBC DNA CNV showed potential unique characteristics in samples from different groups, we wanted to further explore whether lung cancer patients could be screened. Based on 20 samples we used, a total of 4202 1 kb regions with significant CNV differences between groups were screened (Table S2, Supporting Information). The clustering of 20 samples were constructed, which clearly formed three groups (**Figure** **3**A). Although sample LC6 was clustered to the benign nodule group and BN1 was cluttered to the cancer group, the rest samples demonstrated accurate clustering.

Considering the convenience of future study and application, several windows should be enough to screen lung cancer patients from the population. For example, samples from healthy donor group showed significate higher copy number in windows Chr16:34114001 and Chr10:114138001 (Figure 3B); samples from benign nodule group showed significate higher copy number in windows Chr21:44110001 and Chr8:8076001 (Figure 3C); samples from lung cancer group showed significate higher copy number in windows Chr9:40027001 and Chr9:40031001 (Figure 3D). To further increase the specificity and sensitivity, a combination of multiple windows as a screen model would be a good strategy. Here we illustrated two simple 3‐window additive sum models as examples, one included windows Chr8:8076001, Chr10:114138001, and Chr21:44110001 and the other included Chr9:40027001, Chr12:8417001, and Chr16:34114001. Both models could screen all samples of lung cancer groups with lower score (Figure 3E).

### 2.4 RBC Acquired DNA from Lung Cancer Cell Lines In Vitro by Direct Contact

**Figure 4:**
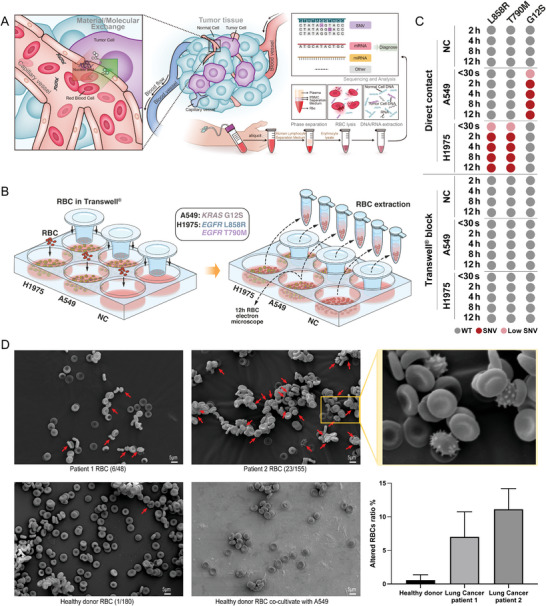
Comparison of RBC from patients with lung cancer and healthy individuals and the ability of RBC to absorb DNA from tumor cells. A) Schematic diagram depicting the exchange of cargo between tumor cells and red blood cells. RBC containing tumor cell‐derived cargo may be used as a potential biomarker source for a liquid biopsy cancer diagnostic. B) Schematic diagram depicting the in vitro co‐culture system using RBC and cancer cell lines A549 and H1975. Briefly, the co‐culture assay was designed with three groups: negative control with no cancer cell but medium, direct contact with cancer cells, and separated by transwell with cancer cells. The transwell contained 0.4 µm pore polycarbonate membrane, which allowed exosomes and cfDNA but not cells to transport. C) Detection of *EGFR* L858R, T790M, and *KRAS* G12S in isolated RBC co‐cultured with cancer cell lines and collected at labeled time points. Gray spots indicate that no mutation was detected, pink spots represent low‐frequency mutations were detected, red spots represent apparent mutations detected. D) Scanning electron microscopy of RBC patients with lung cancer or healthy donor. The numbers in parentheses represent altered RBC/total RBC. The red arrows depict the altered RBC. More additional views are shown in Figure S5, Supporting Information. The bar graph shows mean ratio of altered RBC ± SD (count RBC separately from multiple electron microscopic fields, *n* = 4).

Based on the fact that: 1) RBC DNA from healthy donors and patients with lung cancer demonstrated apparent differences. 2) Mature RBC had no nucleus or mitochondria. 3) Cancer cells recruited RBC to deliver more oxygen for fast development, we hypothesized that molecular (DNA/RNA/protein/other) exchange between RBC and cancer cells might occur at the early stages of cancer development (**Figure** **4**A).

To explore this hypothesis, we designed an in vitro co‐culture assay of RBC from healthy donor and lung cancer cell lines (A549 with *KRAS* G12S and H1975 with *EGFR* L858R, T790M). Briefly, the co‐culture assay was designed with three groups: negative control with no cancer cell but medium, direct contact with cancer cells, and separated by transwell from cancer cells (Figure 4B). The transwell contained a 0.4 µm pore polycarbonate membrane, allowing exosomes and cfDNA but not cells to transport. RBC was collected at time points 30 s, 2, 4, 8, and 12 h, and potential mutation sites *EGFR* L858R, T790M, and *KRAS* G12S were detected by amplicons Sanger sequencing as described in Section 4. To our surprise, the relative *EGFR* mutations can be detected in RBC of direct contact group as short as 30 s incubation at about 10% and increased up to 70% from 2–12 h (Figure 4C and Figure S4, Supporting Information). However, such DNA fragment transfer was not seen in the transwell group in this time range, which confirmed the importance of direct contact. To further eliminate the possible contribution of mutation contained cfDNA, we designed a pair of primers to amplify a 591 bps product of *EGFR* L858R, which was much longer than usual cfDNA. The result was consistent with short amplicon (Figure S4, Supporting Information), which confirmed this DNA transfer was based on larger DNA fragments with direct contact but not cfDNA with long‐distance delivery.

Recently, RBC would acquire exogenous DNA fragments through TLR‐9 receptor and induced the swallow of macrophage by altering the shape, which further induced the innate immune response.^[^ ^7b^ ^]^ With the help of scanning electron microscopy, we observed more altered RBC in samples from both RBC samples of two patients with lung cancer but not in the healthy donor (Figure 4D and Figure S5, Supporting Information). The ratio of altered RBC to normal RBC was around 10% in both patients with lung cancer. However, after co‐culture of RBC with cancer cells A549 (co‐cultured with A549 cell for 12 h, direct contact), no significant change in RBC morphology was seen, suggesting that the exchange of material we detected was not due to the cell‐surface attachment of DNA fragments. This suggested that RBC uptake of cancer cell DNA was through the channel from cancer cells directly into the inner RBC, and the related mechanism needs to be further investigated. On the other hand, it again confirmed the RBC DNA we observed was not csbDNA.

### 2.5 Cancer Mutations can be Detected in Patient RBC

In order to further explore the physiological significance of this phenomenon, we carried out a clinical study to further verify the substance exchange between cancer cells and RBC by targeting mutations that can be produced in cancer cells but not in normal cells.

We analyzed the tissue and RBC samples of 26 patients, including 13 cases from Nanjing Drum Tower Hospital and 13 cases from Peking Union Medical College Hospital. Among those, 21 patients were diagnosed with stage I lung cancer and accounted for more than 80% of the overall population, containing four patients at stage IA, eight at stage IA2, three at stage IA3, and six at stage IB. Therefore, the majority of patients enrolled in this cohort were at the very early stages of cancer development. Therefore, our sample composition is mainly composed of very early patients with lung cancer.

**Figure 5:**
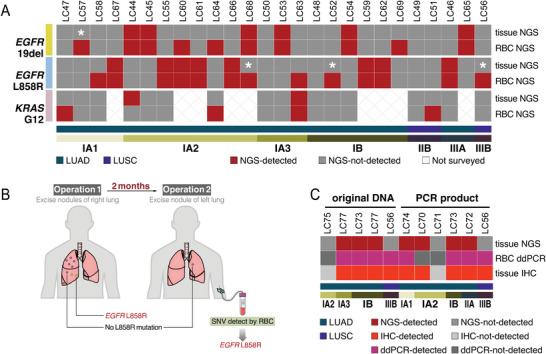
Detection of tumor gene mutations from patients with lung cancer' RBC. A) Heat map showing the detection of *EGFR* 19del/L858R and *KRAS* G12/13 (include G12S, G12R, G12C, G12A, G12D, G12V, G13C, G13D) inpatient tissue or red blood cells. The methods used for detection of the mutations by next‐generation sequencing (NGS). *Represents our tissue NGS results showing negative mutation but positive mutation detected by immunohistochemistry or third party. Patient IDs are labeled at the top of the panel. B) Schematic diagram depicting the surgical process and sample collection steps for patient LC68. (The detailed CT diagram is shown in Figure S11, Supporting Information) C) Heat map showing the detection of *EGFR* L858R in patient tissue using NGS or IHC and RBC using ddPCR. Both original DNA directly obtained after extraction, and PCR product obtained after the amplification of extracted DNA, were used in our analysis. Patient IDs are labeled at the top of the panel.

Based on the next‐generation sequencing (NGS) analysis and tissue mutation status as a reference, seven *EGFR* 19del, 11 *EGFR* L858R, and one *KRAS* G12A were detected in DNA extracted from RBC and classified as true positive results (**Figure** **5**A; Figure S6 and Table S3, Supporting Information). The IHC results of pathology and the NGS results of the third party were used as the reference (Table S3, Supporting Information). Generally, only one patient with quite low mutation ratio (patient LC44, 4% *KRAS* G12S) was missed by RBC detection. However, we detected four “false positive” results in *EGFR* 19del, five “false positive” results in *EGFR* L858R, and three “false positive” results in *KRAS* G12/13 detection. LC57 showed *EGFR* 19del negative in tissue NGS but positive in RBC, which led to a “false positive” case. However, the tissue NGS from the independent organization demonstrated *EGFR* 19del positive with the same cancer tissue but different part with what we acquired, which implied the tumor heterogeneity may cause some “false positive” cases. Similar examples were also seen in patient LC52, LC56, and LC68 *EGFR* L858R (Table S3, Supporting Information).

Meanwhile, since droplet digital PCR (ddPCR) has a better sensitivity to detect low‐frequency mutations, we used ddPCR to analyze nine patient samples from Drum Tower Hospital to confirm the existence of cancer cell‐derived DNA fragments carrying *EGFR* L858R mutations in RBC (Figure 5C and Table S4, Supporting Information). The samples were divided into two variations for analysis: five samples directly used DNA extracted from RBC as a template. Six samples used DNA as templates for PCR amplification first and then used the purified products as templates. Samples LC56 and LC73 were replicated in both variations of experiments. It can be seen that the RBC *EGFR* L858R results measured by ddPCR are generally consistent with the corresponding tissue *EGFR* L858R mutation detected by IHC, except a false negative result of sample LC70 that used its PCR amplified product for ddPCR analysis. Again, as NGS results, *EGFR* L858R was detected in both extracted DNA and amplification products in LC56. Once again, these data proved that RBC contained DNA fragments from cancer cells through material exchange and were not caused by PCR amplification artifacts.

## 3 Discussion

In the beginning part of this study, we aimed to determine whether RBC contained DNA, and if so, what kind of DNA it is? Based on multiple methods, including agarose gel, Agilent chips, and NGS, we clearly captured DNA fragments from RBC from healthy donor and lung cancer patients. However, the accurate sizes of RBC DNA remained unclear due to the fragmentation step of library preparation, which limited our next step to figure out further information about those DNA fragments. Oxford Nanopore Technology may be an excellent choice to access such information since it could acquire ultra‐long reads (>100 kb).^[^ ^19^ ^]^ Previous, csbDNA was reported to be found on blood cells, such as white blood cells and platelets.^[^ ^20^ ^]^ However, csbDNA has not been reported on RBC. In addition, the major RBC DNA fragments we observed were obvious longer than cfDNA or csbDNA.^[^ ^10c^ ^]^ On the other hand, RBC DNA was observed actually inside the cells but with no nucleus structure, the help of stain (Figure 1A,B). Although mature RBC lost nucleus and mitochondria while generating from multipotent hematopoietic stem cells, the destiny of nuclear and mitochondrial DNA was still disrupted.^[^ ^1^ ^]^ Most studies reported that enucleation and nucleus fragmentation occur during erythrocyte maturation,^[^ ^5^ ^]^ however, fewer studies believed some DNA fragments remained in the mature RBC.^[^ ^6^, ^7^, ^10^ ^]^ Now, we confirmed the existence of DNA fragments in the mature RBC both from nuclear and mitochondrial DNA, which excluded the possibility of contamination from other nucleated cells or bacteria (Figure 1E).

Furthermore, the DNA fragments from nuclear and mitochondrial genome seemed to go through different mechanisms, which led to a dramatically different coverage depth ratio between them compared with nucleated cells. To further explore this discovery, we collected 45 RBC samples, including 25 patients with lung cancer, 15 patients with benign lung nodules, five healthy donors together with four lung cancer tissue samples, six lung normal tissue samples, and A549, 293t, Hela cell line, and used quantitative PCR to measure the relative ratio between mitochondrial (used *CO1* gene as marker) and nuclear genome (used *GADPH* gene as marker) copy number. The results demonstrated a highly stable ratio of RBC samples from 25 patients with lung cancer and 15 patients with benign lung nodules between eight to 72‐fold, significantly lower than three cell lines and normal tissue samples but no significant difference with tumor tissue samples (Figure S7, Supporting Information). Such discovery indicated that the process of mature RBC generation was more complicated than previous understanding and was worth further exploration.

As the main cells of oxygen and nutrient transport, RBC plays an important role in the occurrence and development of cancer. This study confirmed that RBC could acquire DNA from cancer cells both in vitro assay and patients with lung cancer using mutations as markers. While a live cell cannot separate a piece of genomic DNA and give it to another cell while it is alive, one possible material was the micronucleus, which was reported to be discrete DNA aggregates separate from the primary nucleus in cells.^[^ ^21^ ^]^ Based on the previous studies, micronucleus was found in tumor cells, lymphocytes, epithelial cells, and other cells, a common outcome of many cell division defects.^[^ ^22^ ^]^ Meanwhile, the frequency of micronucleus in lymphocytes is associated with cancer development.^[^ ^23^ ^]^ In the late 19th and early 20th centuries, micronucleus was found in RBC by William Howell and Justin Jolly. Those RBC were called micronucleated erythrocytes and were served as an indicator of genotoxic exposure in splenectomized individuals.^[^ ^24^ ^]^ Furthermore, incidence of micronucleated erythrocytes in human peripheral blood circulation can be used to index recent cytogenetic damage.^[^ ^6^ ^]^ However, abundant DNA fragments were also discovered in RBC healthy donor, which could not be simply explained by micronucleus. Another possible DNA fragments were circular extrachromosomal DNA (ecDNA) reported in many studies about cancer.^[^ ^25^ ^]^ ecDNA release from the cancer cell can be achieved through different mechanisms, including large extracellular vesicles, exosomes, or extracellular particles like exomeres or chromatimeres.^[^ ^26^ ^]^ However, we did not discover any potential ecDNA‐like read in our NGS data. As one of the most famous cancer‐driven genes, *EGFR* was reported to form ecDNA and overexpressed in lung cancer.^[^ ^27^ ^]^ Here, when we enlarge the region of *EGFR* and *GAPDH*, there is a clear difference in DNA distribution and copy number (Figure S8, Supporting Information). To further confirm this discovery, quantitative PCR was applied to measure the relative copy number of *EGFR*/*GAPDH* with the same 45 RBC samples in the previous part. The results revealed no significant amplification of *EGFR* gene copy number in RBC DNA of cancer patients compared to healthy subjects and benign nodules (Figure S9, Supporting Information). However, in different patients, the copy number ratio of *EGFR*/*GAPDH* in RBC DNA showed large differences compared to normal tissue DNA (Figure S10, Supporting Information). In addition, we observed the altered RBC of patients, which may be related to the acquirement of extra DNA from other cells. Based on recent studies, RBC would acquire exogenous DNA fragments through TLR‐9 receptor and induce the swallow of macrophage by altering the shape, which further induces the innate immune response.^[^ ^7b^ ^]^ Such a process demonstrated a potentially important role of RBC in the immune system,^[^ ^28^ ^]^ which recognized cancer cells in the early and presented the information to macrophages.

Our study also highlights the potential translational application of our discovery. Specifically, we demonstrated that we could consistently detect common *EGFR* and *KRAS* mutations using RBC collected from patients with early‐stage NSCLC, implying that our discovery can form the basis of a new liquid biopsy technology. One of the issues in our study was that we seemed to obtain a high false‐positive rate. Although some samples could be explained by tumor heterogeneity as we showed in results, other false‐positive cases still need further study to figure out the reason. In our follow‐up study, we will optimize our method of tissue sampling, extract DNA from multiple parts of cancer tissues and cover more primary nodules to obtain accurate tissue mutation results to the greatest extent. Furthermore, the ddPCR results suggested that when the proportion of mutation fragments in the sample is deficient, the PCR process may further reduce the proportion of mutation fragments in the product due to the sampling deviation, resulting in the inaccurate final detection results. Therefore, optimizing PCR sampling deviation and enrichment of mutation fragments should be considered.^[^ ^29^ ^]^

Interestingly, patient LC68, who had multiple primary lung adenocarcinoma and nodules on separate lobes, underwent two consecutive operations in a short period (Figure 5B). The patient underwent the first operation in November 2019 to remove nine cancer nodules from the patient's right lung lobe. Then, in January 2020, the patient underwent a second operation to remove the left lung superior nodule with one cancer nodule (Figure S11, Supporting Information). In our study, we were first given obtained tissue samples and RBC samples from the second operation. Our results showed no *EGFR* L858R mutation in the tissue, but *EGFR* L858R mutation was detected in RBC. Fortunately, tissue samples from both operations were sent to a third party for NGS analysis. Although no *EGFR* L858R mutation was detected in the second operation's cancer tissue, *EGFR* L858R mutation was detected in the two largest nodules from the first operation. These data revealed tumor tissue genomic heterogeneity in this patient while also revealing that RBC‐associated cancer DNA fragments may remain in the whole blood circulation of patients for at least 2 months, which is much longer than the half‐life of circulating tumor DNA.^[^ ^30^ ^]^

Distinguishing benign and malignant lung nodules, especially those smaller than 1 cm, has always been a dilemma that confuses clinicians and patients. Among all new methods, liquid biopsy seemed to be a potential direction to non‐invasively provide additional information for nodule pathology determination.^[^ ^31^ ^]^ This study demonstrated an apparent clustering of small benign and malignant nodules based on RBC DNA CNV, which provided a novel direction for future cancer diagnosis studies. Interestingly, some regions showed great potent in distinguishing samples from different groups. Although the combination model of multiple windows screened samples of lung cancer group accurately, a larger‐scale validation study was needed to further evaluate this discovery. On the other hand, the mechanism why those regions demonstrated significant copy number variations within and between samples worth further study.

## 4 Experimental Section

### Sample Collection

This study recruited 84 patients with suspected lung cancer and 15 patients with benign nodules. The specific inclusion and exclusion criteria are shown in the Table S1, Supporting Information. The blood samples were collected within 1 h before biopsy, 1 h before or during operation, and were further separated into plasma, PBMC, and RBC based on the description in Section 4; the tissue samples were punctured samples or surgical tissue samples, which were further diagnosed as lung cancer by the hospital pathology department through HE staining, and then the cancer tissues in the whole tissue samples were further separated based on the pathological sections.

The study was conducted at Peking Union Medical College Hospital and Nanjing Drum Tower Hospital. Based on the different hospital situations, the research plans of the two centers were slightly different: in Peking Union Medical College Hospital, the separation of blood samples was completed locally in Beijing and transported the separated RBC and tissue to the laboratory in Nanjing within 7 days to complete the subsequent NGS or ddPCR test; Peking Union Medical College Hospital screened the diagnosis of the same patient based on NGS in a third‐party company as well as the mutation detection based on serum cfDNA. In Nanjing Drum Tower Hospital, the separation of blood samples was completed and the following NGS or ddPCR in Nanjing laboratory, and mutation of cancer tissue was screened through NGS; the pathology department completed the detection of L858R and 19del mutations of *EGFR* of the same patient by tissue IHC staining method. Follow up statistics based on RBC and tissue detection results for statistical comparison. The tissue IHC or third‐party NGS results were considered as a reference. The ethics committee has approved the relevant research of the two hospitals. The clinical trial of this study is registered with the Chinese Clinical Trial Registry (registration number: ChiCTR2100042157; ChiCTR2100042604)

### RBC Isolation and DNA Extraction

Whole blood was taken from patients and collected in Vacutainer tubes containing EDTA. Blood samples were processed within 12 h of collection. RBC was separated by using the density gradient method. First, the blood was separated by centrifugation (Eppendorf, Centrifuge 5810R) at 150 g for 10 min, and the supernatant was removed. Then, an equal volume of PBS was added to the precipitate and mixed gently. The diluted precipitate was then slowly added dropwise to 1.5 times the precipitate volume of the Human Lymphocyte Separation Medium (Dakewe Biotech, China) and centrifuged at 800 g for 15 min then slowly removed the supernatant. Then, an equal volume of PBS was added to the precipitate, mixed gently, and then centrifuged at 150 g for 15 min. 3× volume of RBC lysis buffer was added to purified RBCs, and the supernatant lysate was collected after centrifugation. DNA from RBC was extracted using the FastPure Blood/Cell/Tissue/Bacterial DNA Isolation Mini Kit (Vazyme, China) based on the principle of affinity column. The RBC DNA of patient LC10 with lung cancer was also extracted with Nextractor Whole Blood DNA Kit (Genolution, Korea) based on the principle of magnetic beads, according to the manufacturer's protocol.

### Flow Cytometry

Red blood cells were incubated with FITC anti‐human CD45 Antibody (HI30) (Biolegend, USA) and PE anti‐human CD235a (Glycophorin A) Antibody (HI264) (Biolegend, USA) for 60 min. Subsequently, the red blood cells were washed three times with PBS for 5 min each in the dark and analyzed by a Becton Dickinson FACScan. To analyze the contamination in the isolated red blood cells, the red blood cells were incubated with parcific blue anti‐human CD45 Antibody for white blood cell (#368 521, Biolegend, USA), FITC anti‐human CD11b Antibody for monocyte (#101 205, Biolegend, USA), PE/cy5.5 anti‐human CD3 Antibody for T lymphocyte (#17 335, Biolegend, USA), and PE anti‐human CD235a Antibody for red blood cells (#306 604, Biolegend, USA).

### DAPI and PI Staining

Purified RBC was diluted with PBS to concentration approximately 10^7^ mL^−1^. 100 µL of RBC was then fixed with glutaraldehyde for 5 min, followed by PBS wash for two times, and finally resuspended with 100 µL PBS. 100 µL DAPI or PI dye was added to the RBC (100 µL PBS for the control group) and incubated for 15 min, followed by centrifugation to remove the supernatant. Samples were washed by PBS for two times and resuspended with 100 µL staining buffer, followed by subsequently flow cytometry and confocal microscopy observation.

### AFM Imaging

1/5 volume of 5× imaging buffer (50 mm magnesium acetate, 50 mm Tris‐HCl pH 8.0) was added to the sample to reach a final DNA concentration of 1.0 ng µL^−1^, and 100 µL of the mixture was then spread on mica surface. After 5 min of incubation, the specimen was rinsed twice with 100 µL of 2 mm magnesium acetate, drying before and after the rinses with compressed air. Images were acquired by Bioscope Resolve (Bruker, USA) atomic‐force microscope.

### NGS Library Preparation and Sequencing

A total of 21 RBC DNA were used for NGS, with samples from three healthy donors, six patients with benign nodules, and 11 patients with lung cancer (RBC DNA from LC10 was extracted by both column and magnetic beads as two independent samples). The DNA library of each sample was built using adaptors of Insert UMI Kit for ILM (iGeneTech, China) based on NEBNext Ultra II FS DNA Library Prep Kit (New England BioLabs, USA) and according to the manufacturer's protocol. The product was quality checked, and sequencing was performed in paired‐end 150 mode with Illumina Novaseq (Illumina, USA).

### NGS Data Analysis

Cutadapt 3.5^[^ ^32^ ^]^ was used to trim adapters and filter the low‐quality sequences from the raw data. Then UMI‐tools V1.1.2^[^ ^33^ ^]^ was used to extract 6 bp UMI contained on both 5′ and 3′ end of inserted sequences. BWA‐MEM2 2.0pre2^[^ ^34^ ^]^ was used to align reads to the GRCh38 reference genome, and output data was processed with SAMtools v1.14.^[^ ^35^ ^]^ UMI marked reads were duplicated by UMI‐tools from the BAM files. The mapping results were visualized by IGV 2.11.4.^[^ ^36^ ^]^ Mosdepth^[^ ^37^ ^]^ default parameter was used to calculate the average sequencing depth of WGS and additionally set “window = 1000” and “depth <1” to missing windows in RBC DNA. Next, Perl scripts were used to join the contiguous missing windows into regions (length >10k, average depth <0.1).

### Copy Number Analysis

CNVs of RBC DNA sequencing data were identified with GATK (v4.2.6.1) GermLineCNVCaller in “–cohort” mode.^[^ ^38^ ^]^ In brief, 21 RBC DNA sequencing data were used for analysis, and the copy number calculation for each RBC DNA was based on the overall calculation. Using 1 kb as a moving window, the average copy number of DNA within 1 kb was calculated, and the copy number was divided into integers 1–5, with values greater than 5 set to 5.

The data were then divided into three groups based on the sample source: healthy donors, patients with benign nodules, and patients with lung cancer. Loci with significant differences in copy number between the three groups were screened, and the samples were clustered based on these loci.

### Electron Microscopy

Purified RBC were washed with PBS and fixed with 0.05% glutaraldehyde. Scanning electron microscopy sample preparation and acquisition was performed by the Zhongjingkeyi Technology company(China).

### Quantitative PCR

To compare the relative content of *EGFR* or mitochondrial DNA in different samples, the *EGFR* gene or the mitochondrial *MT‐CO1* (mitochondrial encoded cytochrome *c* oxidase subunit I) gene was used as the target gene and the *GAPDH* gene as the reference. All reactions were performed with 20 ng of sample or control DNA, 200 nm primer concentrations, and SYBR Green 2X master mix chemistry reagents. According to the manufacturer's protocol, DNA in samples was measured on LightCycler 96 System (Roche, Germany) using ChamQ Universal SYBR qPCR Master Mix (Vazyme, China).

### Red Blood Cells Co‐Culture with H1975 and A549 Cells

H1975 and A549 cells were seeded at a density of 1  ×  10^4^ cells per well in the 24‐well plates. After 24 h, the obtained red blood cells, which were isolated from healthy donors by using Ficoll–Paque density centrifugation were added to each well at a ratio of 0.5:1. RBCs were separated from the co‐culture system via differential centrifugation method after the experiment (<30 s) and 2–12 h. The purification of red blood cells was confirmed by optical microscope examination.

### Amplicon NGS Sequencing

According to the manufacturer's instructions, DNA from RBC and tumor tissues was extracted using TIANamp Genomic DNA Kit (Tiangen). PCR reactions were prepared in 50 µL volumes containing 1 × Ex Taq Buffer (Mg^2+^ plus) (Takara, Japan), 0.4 µm of each primer, 0.2 mm of each of the four deoxynucleoside triphosphates (dNTPs), 10–100 ng of template DNA, 1.25 U of TaKaRa Ex Taq HS (Takara Japan). The PCR conditions consisted of a denaturation period at 98 °C for 2 min followed by 35–38 cycles at 98 °C for 10 s, 60 °C for 30 s, and 72 °C for 30 s, followed by a final elongation at 72 °C for 5 min. The PCR products were purified using AxyPrep PCR Clean‐Up Kit (Axygen, USA) according to the manufacturer's instructions and quantified by Nanodrop One (Thermofisher, USA). Purified PCR products were used for library construction, and sequencing was performed in paired‐end 150 mode with Illumina HiSeq Xten (Illumina, USA).

### Mutation Detection by ddPCR

Bio‐Rad QX200 did both original DNA and amplification products ddPCR with *EGFR* L858R mutation detection kit following the manual. A two‐step PCR prepared amplification products: the first‐round PCR reaction was done with only forward primer for 15 cycles in 50 µL volumes containing 1 × TransStart FastPfu Buffer (Transgen, China), 0.2 µm forward primer, 0.2 mm of each of the four deoxynucleoside triphosphates (dNTPs), 5–10 ng of template DNA, 2.5 units of FastPfu DNA Polymerase (Transgen, China). For the second‐round PCR, 0.2 µm reverse primer was added to the reaction solution to amplify for 25 cycles. The PCR products were purified using AxyPrep PCR Clean‐Up Kit (Axygen, USA) according to the manufacturer's instructions and quantified by Nanodrop One (Thermofisher, USA).

## Conflict of Interest

The authors declare no conflict of interest.

## Author Contributions

N.L., Z.J., C.Z., and Y.W. contributed equally to this work. Q.C., H.L., and J.C. designed this research; N.L., Z.J., C.Z., T.W., and S.L. performed this research; Q.C. and Z.C. wrote the manuscript; Y.W. and T.S. analyzed the data; N.L. and Z.J. provided the clinical samples and pretreatment.

## Supporting information

Supporting Information

## Data Availability

The data that support the findings of this study are available on request from the corresponding author. The data are not publicly available due to privacy or ethical restrictions.
