# Supplementary material for: Mature Red Blood Cells Contain Long DNA Fragments and Could Acquire DNA from Lung Cancer Tissue
Source: Adv Sci (Weinh). 2023 Jan 4;10(7):2206361. doi: 10.1002/advs.202206361 (PMC9982546; doi:10.1002/advs.202206361)
Supplement: Supplementary file 1 — Supporting Information [file ADVS-10-2206361-s001.pdf]

ADVANCED SCIENCE
Open Access

Supporting Information

for *Adv. Sci.*, DOI 10.1002/advs.202206361

Mature Red Blood Cells Contain Long DNA Fragments and Could Acquire DNA from Lung Cancer Tissue

Naixin Liang, Zichen Jiao, Cong Zhang, Yifan Wu, Tao Wang, Shanqing Li, Yadong Wang, Tianqiang Song, Jian-Qun Chen, Hongwei Liang and Qihan Chen**

Supplemental Fig1

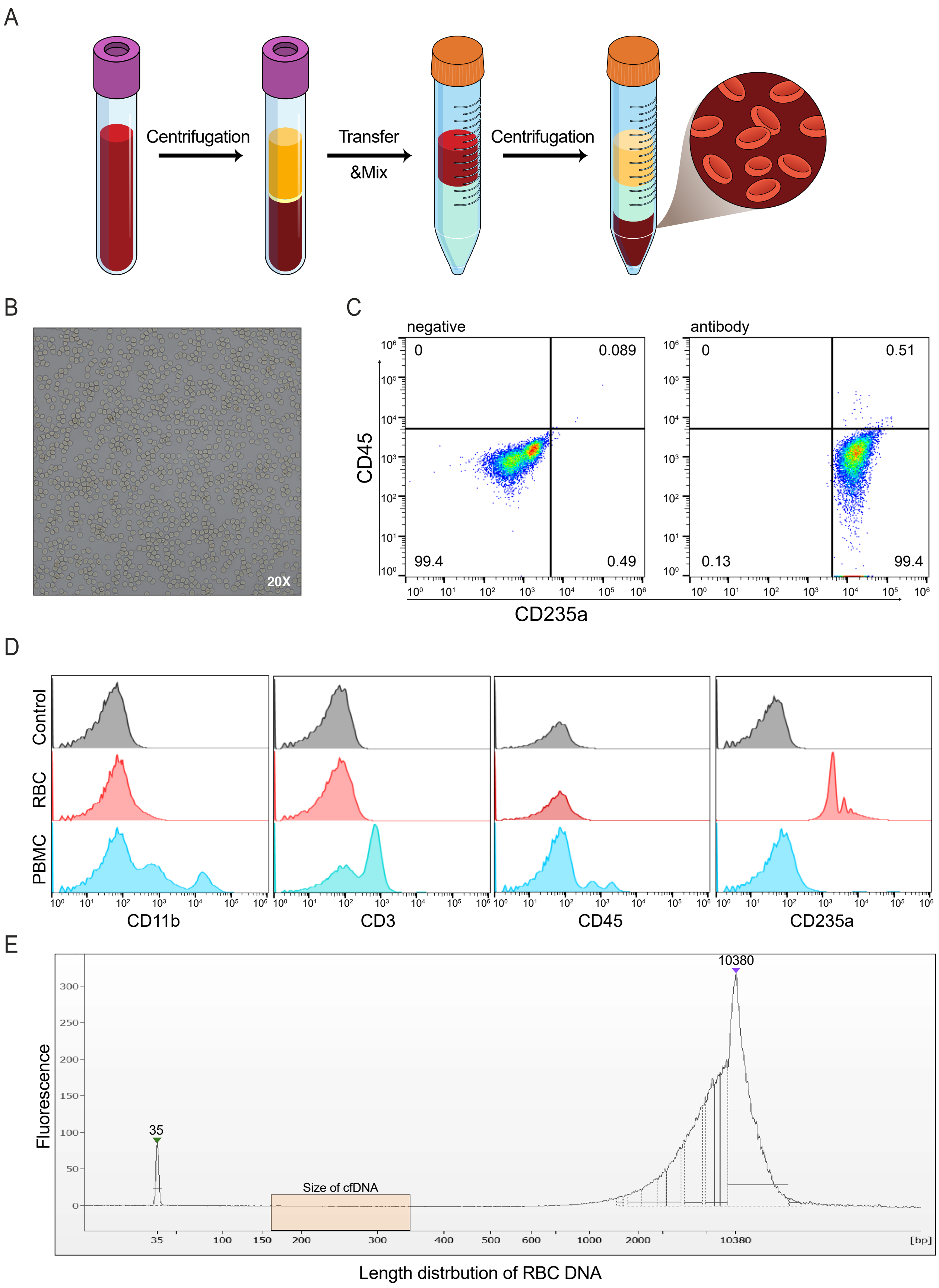

Supplemental Fig1. A. Schematic diagram depicting the purification process of red blood cells from whole blood. Following centrifugation of whole blood, the bottom layer RBC are collected and centrifuged using the density gradient method to obtain purified RBC. Please refer to the method section for detailed methods. **B.** Light microscopy analysis of purified red blood cells. **C.** Flow cytometry analysis detecting the presence of CD45 and CD235a to determine the purity of RBC preparation. **D.** Flow cytometry detection of CD11b, CD3, CD45 and CD235a antigens in purified RBC and PBMC respectively. **E.** The size of DNA extracted from RBC of healthy donor was analyzed by Agilent 2100 Bioanalyzer. Marker: 35 bps and 10380 bps (indicated by inverted triangle). According to the signal, the fragment size of RBC DNA starts from ~1000 bp, and most DNA fragments were larger than the maximum marker (10380 bp).

Supplemental Fig2

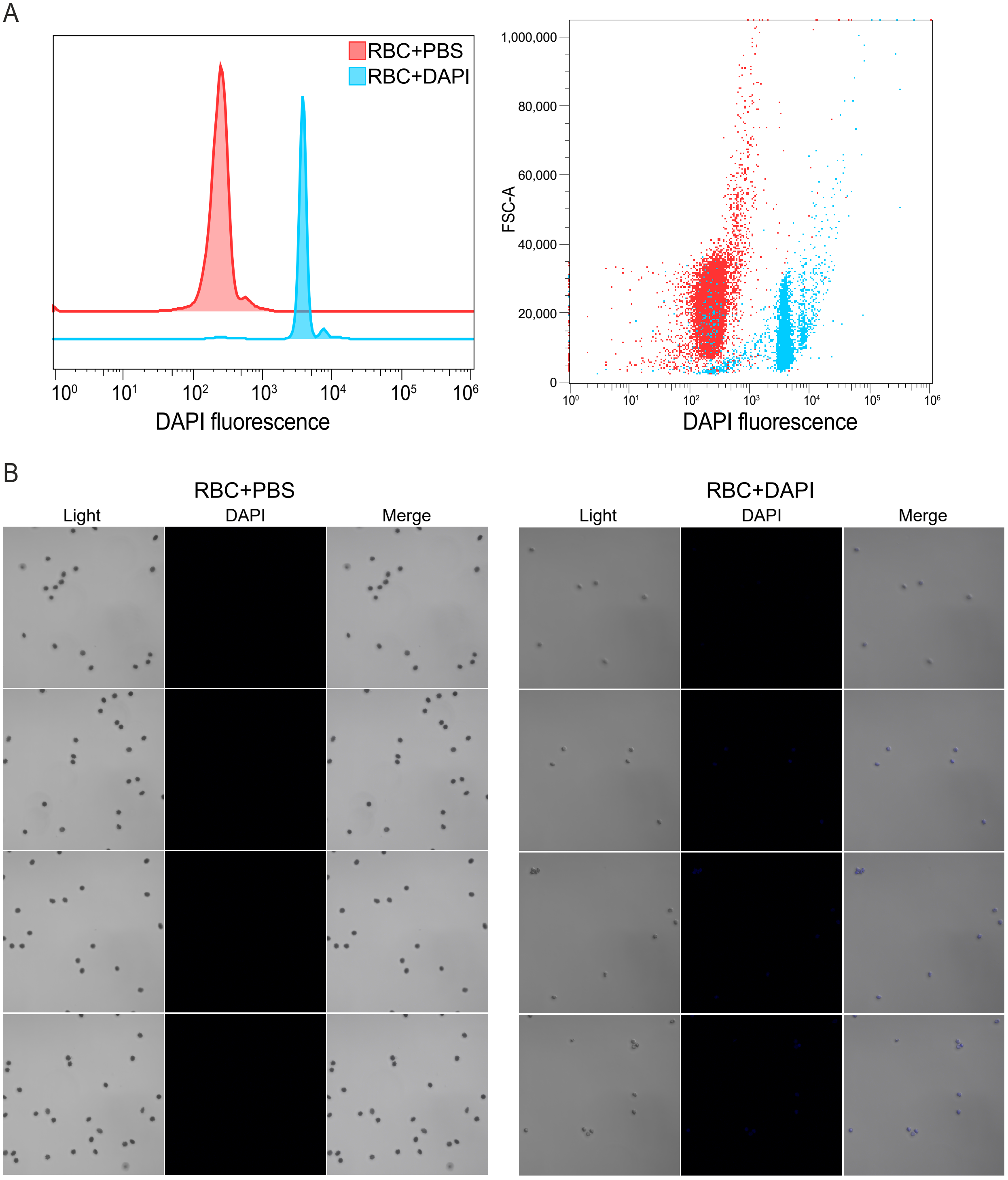

Supplemental Fig2. A. (left) Flow cytometry by DAPI florescence of no-stained (red) and DAPI-stained RBC samples (blue) were shown. **(right)**Imaging flow cytometry reveals no-stained (red) and DAPI-stained RBC (blue) populations. **B.** More confocal images show DAPI stained RBC and no stained RBC**.**

Supplemental Fig3

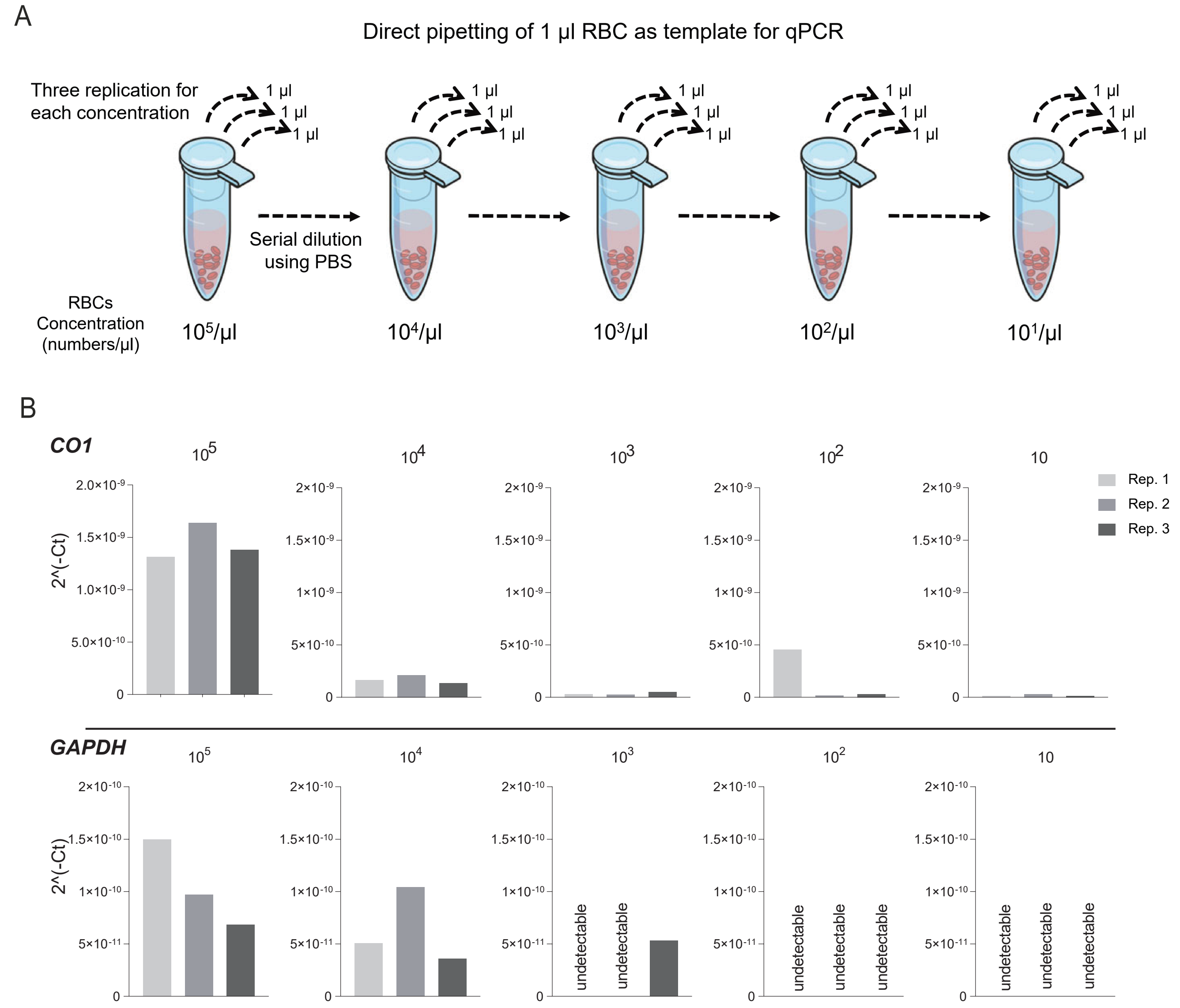

Supplemental Fig3. A. Schematic diagram of the relative quantification of DNA fragments in red blood cells. The RBCs were diluted in a gradient concentration and 1μl of each concentration was directly pipetted as template for the qPCR reaction, and three replicates were performed for each concentration. Whether there is a difference in the DNA present in equal numbers of RBCs can be reflected by the difference in the Ct values of the three replicate experiments. **B.** Relative quantification of *GAPDH* and mitochondrial gene *CO1* for DNA in RBCs with the same number of cells.

Supplemental Fig4

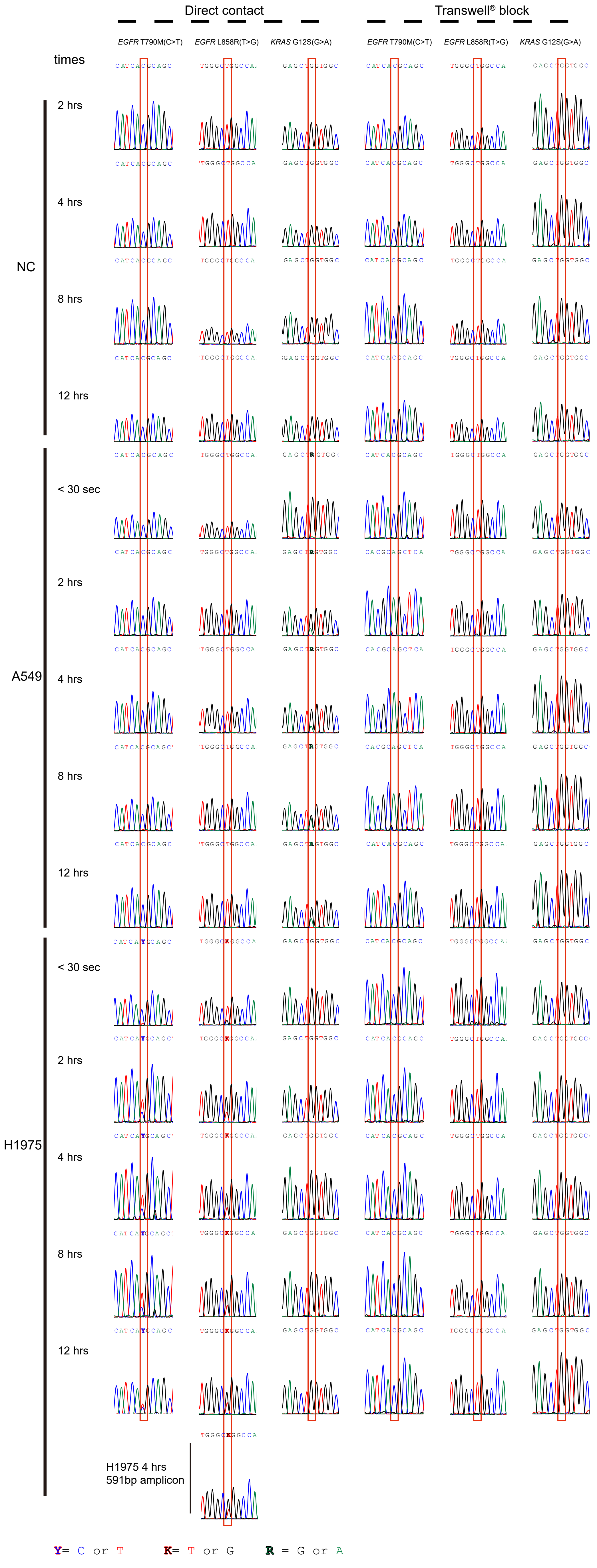

Supplemental Fig4. Sanger sequencing evaluation of the DNA mutation results of red blood cells co-cultured with A549 cells (with *KRAS* G12S mutation), H1975 cells (with *EGFR* L858R and T790M mutation), and no tumor cells (as the negative control, NC) treated by PCR after just started the experiment (< 30 sec) and 2-12 hrs. The *in vitro* co-culture system contains two different types, red blood cells in direct contact with tumor cells or blocked by transwell with 0.4 μm pore polycarbonate membrane. Red boxes indicate the mutation site. Mutation ratio (%) was calculated using peak height of mutated bases/(mutated bases + unmutated bases)

Supplemental Fig5

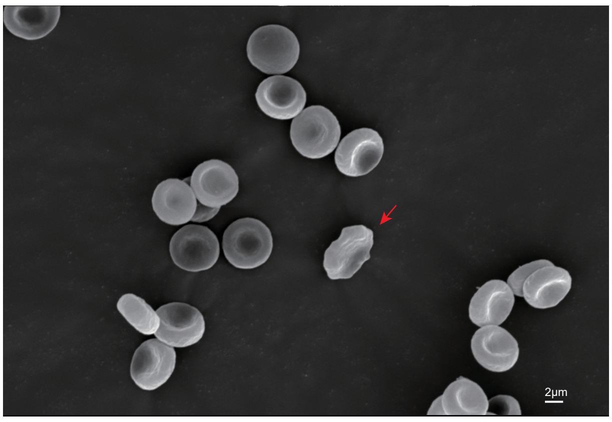

patient 1 (1/18)

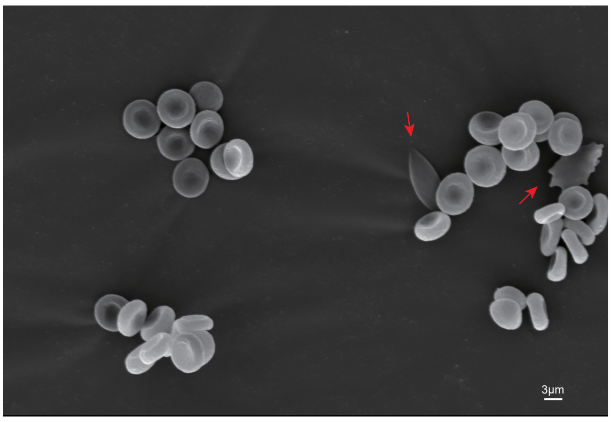

patient 1 (2/35)

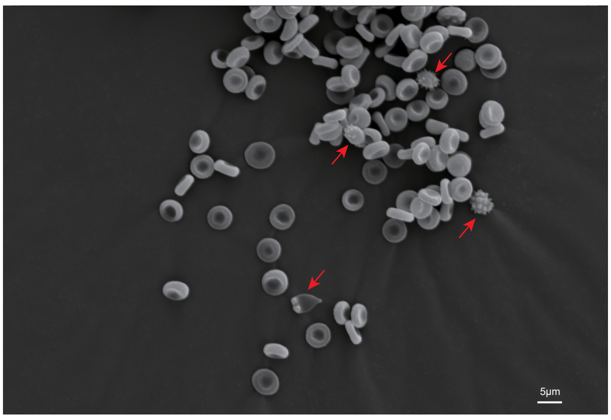

patient 1 (4/97)

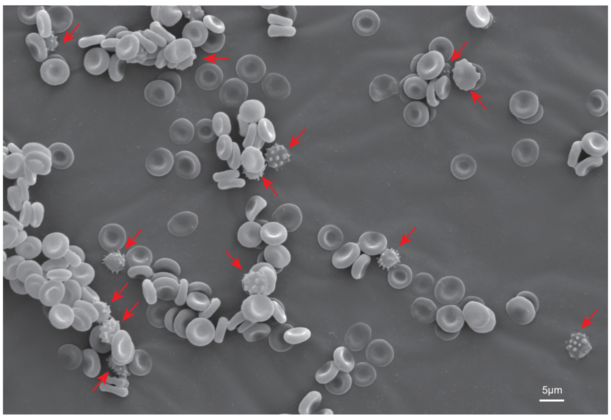

patient 2 (14/150)

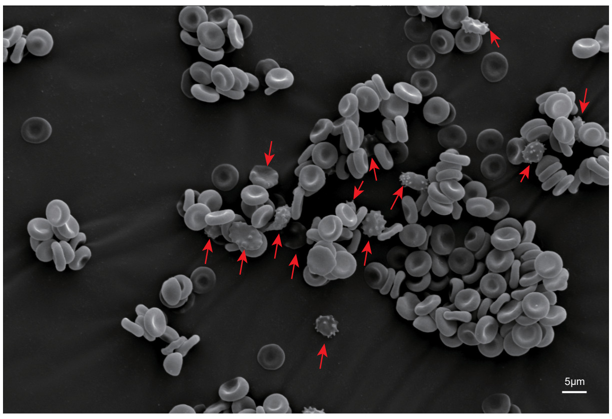

patient 2 (13/162)

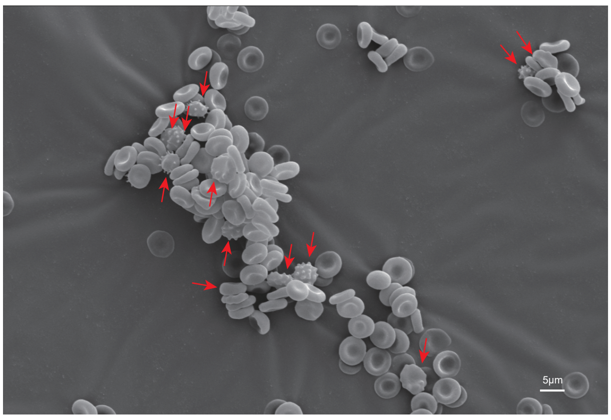

patient 2 (12/97)

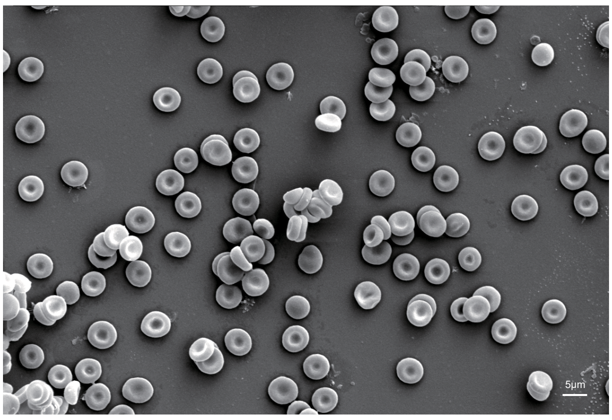

healthy donor (0/100)

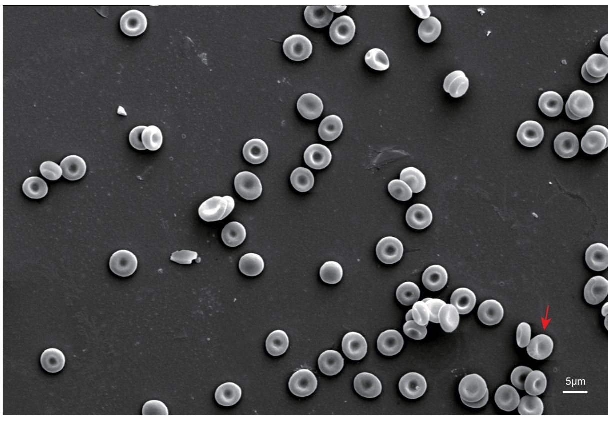

healthy donor (1/60)

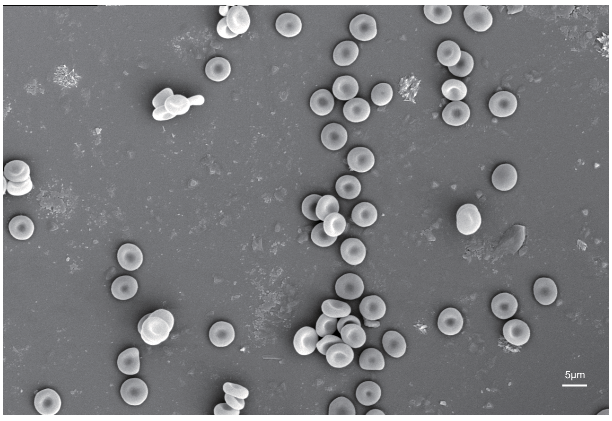

healthy donor (0/55)

Supplemental Fig5. Scanning electron microscope image view with altered RBC. Altered RBC were counted and displayed as altered RBC/total RBC

Supplemental Fig6

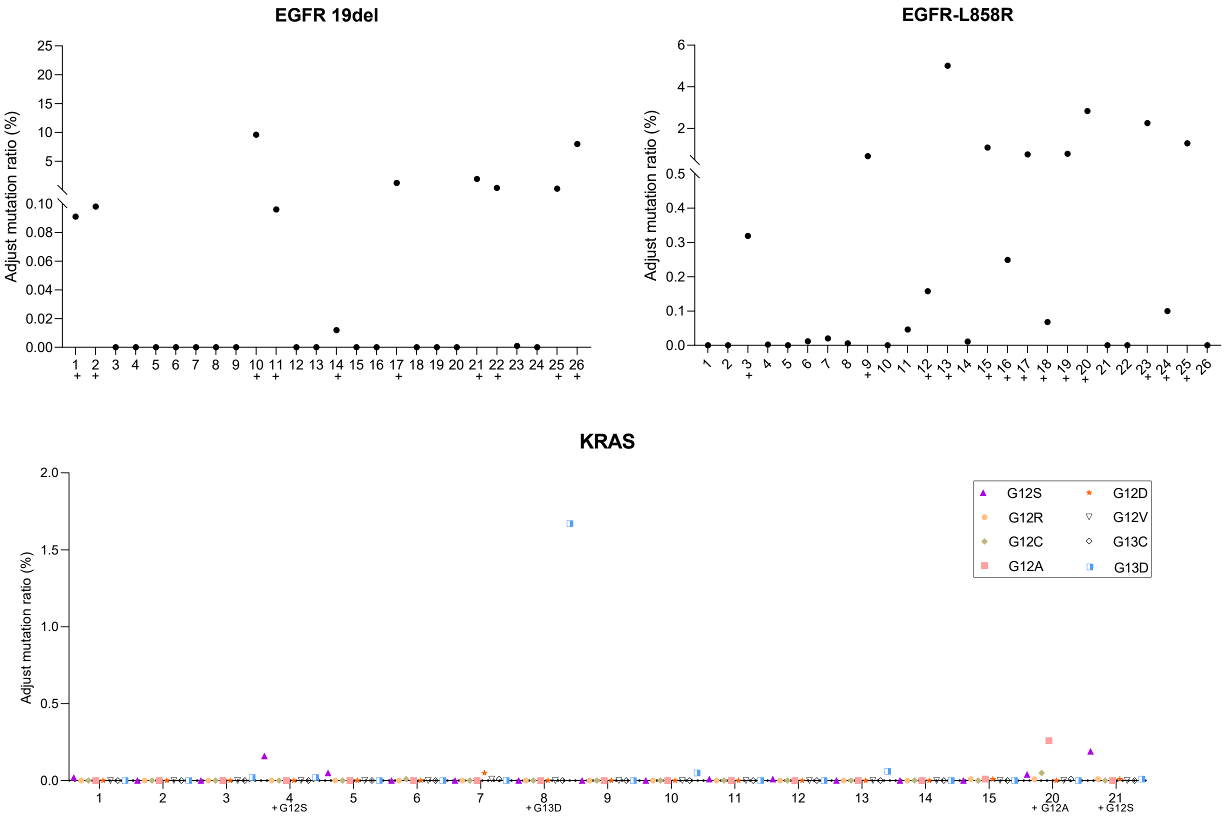

Supplemental Fig6. *EGFR* and *KRAS* gene mutations were detected by NGS sequencing in RBC DNA of 26 clinical patients. The mutations included: *EGFR* 19del, L858R and *KRAS* G12/13 (include G12S, G12R, G12C, G12A, G12D, G12V, G13C, G13D). The symbol + means that the corresponding mutation was detected in the RBC DNA of this patient.

Supplemental Fig7

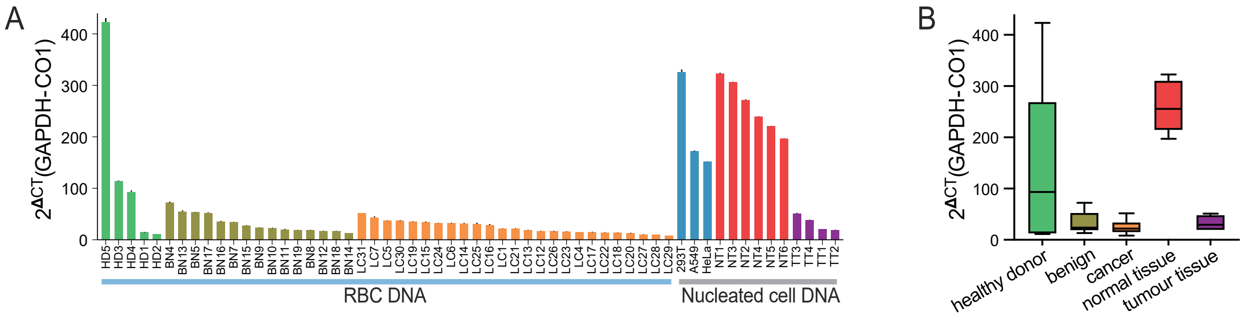

Supplemental Fig7. A. Detection of the relative ratio of GAPDH to EGFR gene using real-time quantitative PCR. Bar graph shows mean±SEM (n=3 technical repeats). The data from the A-plot was integrated with respect to the sample type to obtain the B.

Supplemental Fig8

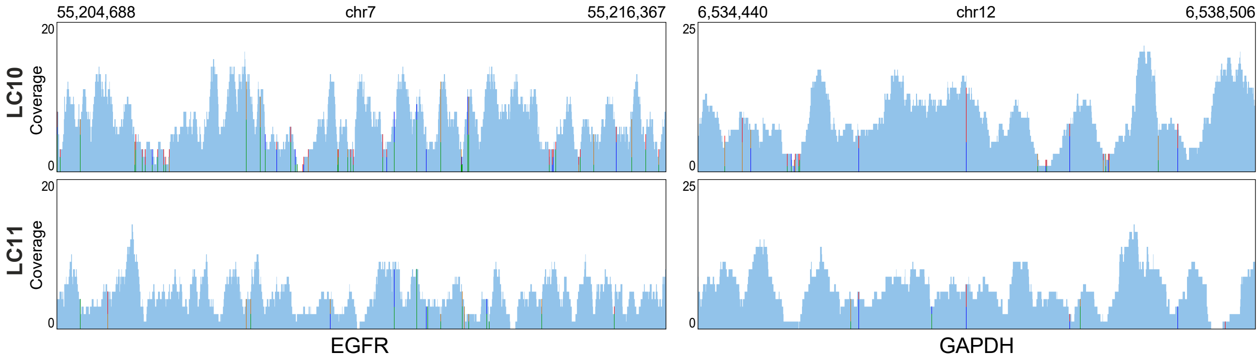

Supplemental Fig8. RBC DNA-seq mapping results in the *EGFR* and *GAPDH* regions from 2 patients with lung cancer.

Supplemental Fig9

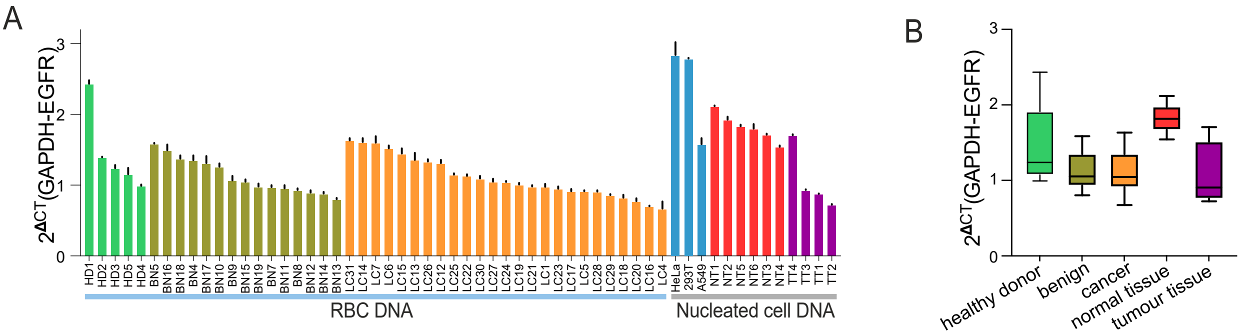

Supplemental Fig9. A. Detection of the relative ratio of *GAPDH* to the mitochondrial *CO1* gene using real-time quantitative PCR. Bar graphA shows mean±SEM (n=3 technical repeats). The data from the A-plot was integrated with respect to the sample type to obtain the B-plot.

Supplemental Fig10

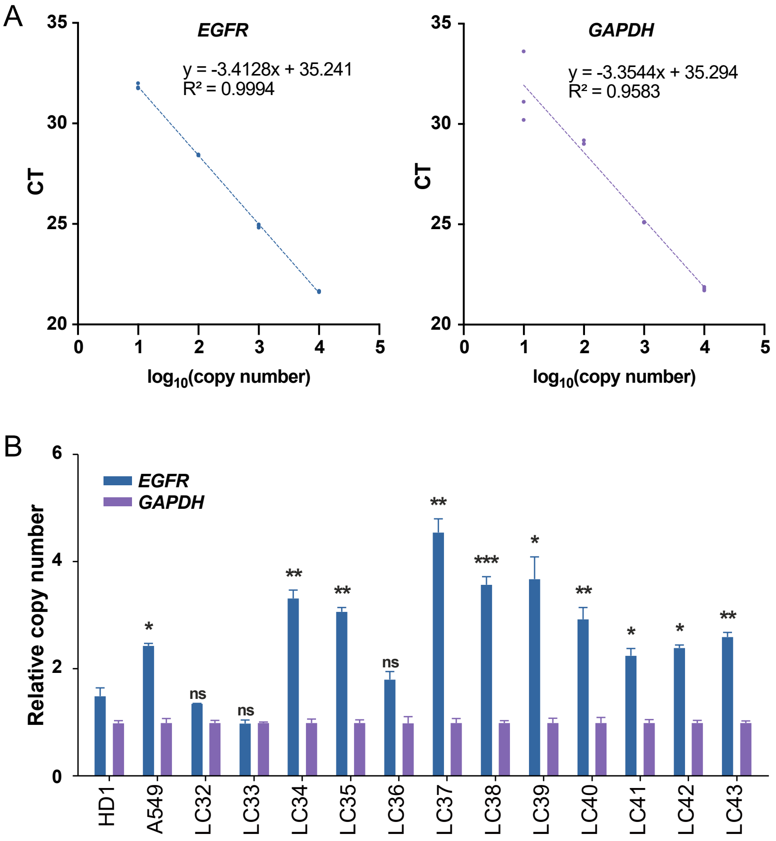

Supplemental Fig10. Detection of *EGFR* and *GAPDH* gene absolute copy numbers in RBC DNA of patients with lung cancer and healthy donor and the genomic DNA of A549 cells by quantitative PCR (three technical repeats). **A** graph shows the standard curve for the absolute quantification of qPCR. The bar graph shows mean±SEM. ***P<0.001, **P=0.001~0.01, *P=0.01~0.05, ns>0.05, unpaired t-test with Welch's correction.

Supplemental Fig11

Right lung surgery in November 2019 - total of 9 cancer nodules

Upper lobe of right lung: 6 cancer nodules

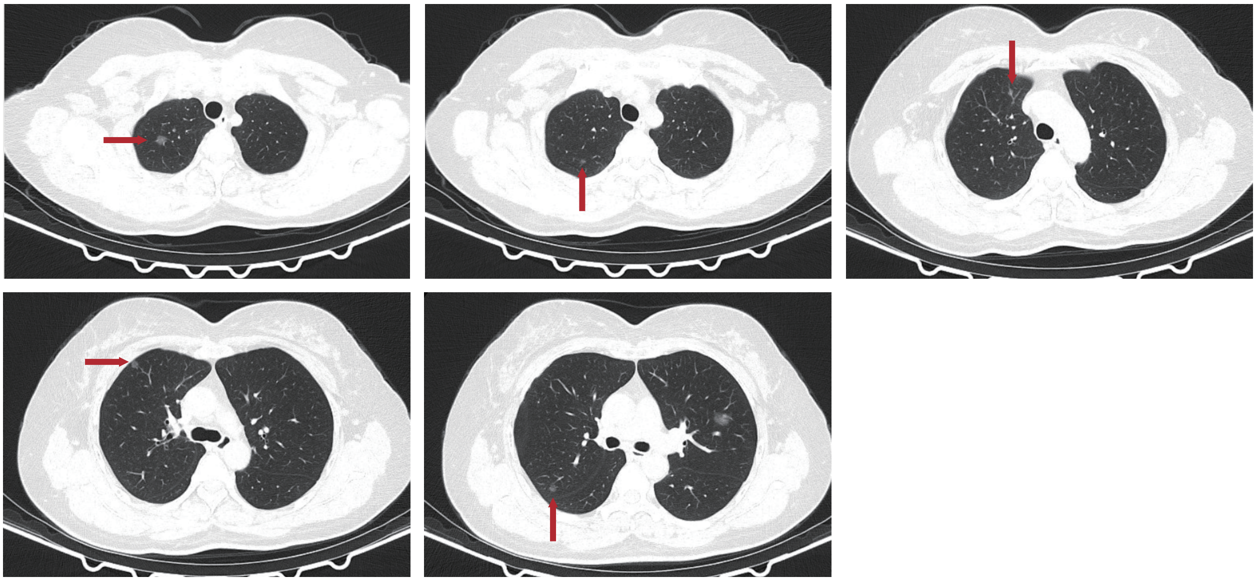

Middle lobe of right lung: 3 cancer nodules

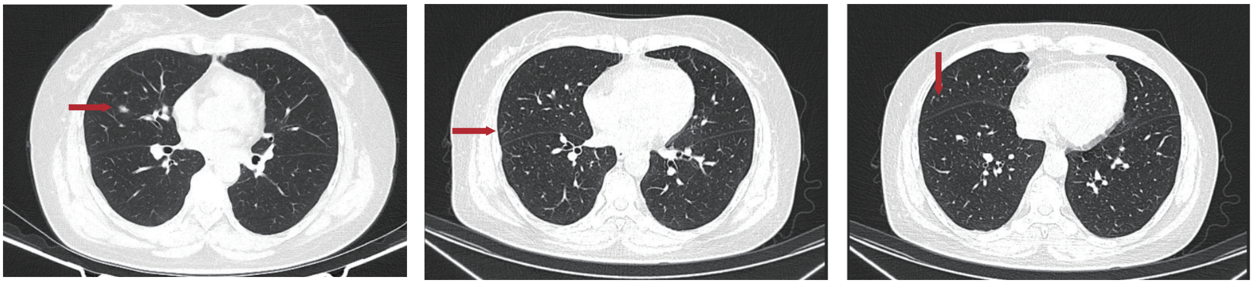

Lower lobe of right lung: 1 cancer nodules

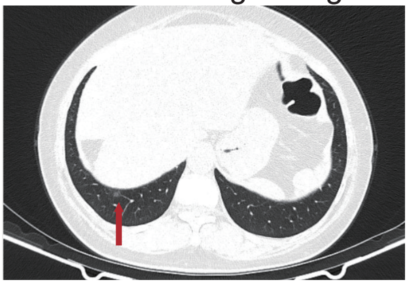

Left lung surgery in January 2020 - 1 cancer nodule

Upper lobe of left lung: 1 cancer nodule

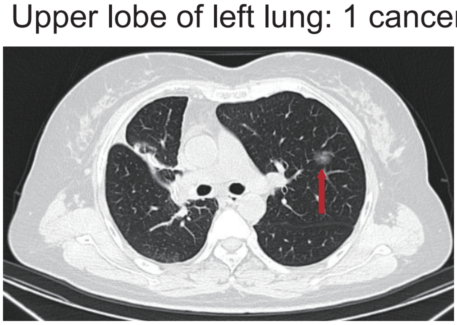

Supplemental Fig11. Computed tomography (CT) image of LC68 patient's lung, arrows represent tumor nodules.

Supplemental Table 1. Clinical samples used in this study

SampleID	Institution	Sample Type	Type	Stage	TNM	Other info.	
HD1	GL	RBC DNA	N/A	N/A	N/A	healthy donor, RBC DNA WGS, qPCR detection of *EGFR* and *MT-CO1* DNA fragments	Fig1, Fig 2, Fig3,Supplemetal Fig 6, Supplemetal Fig 8
HD2	GL	RBC DNA	N/A	N/A	N/A	healthy donor, RBC DNA WGS, qPCR detection of *EGFR* and *MT-CO1* DNA fragments	Fig 2, Fig3,Supplemetal Fig 6, Supplemetal Fig 8
HD3	GL	RBC DNA	N/A	N/A	N/A	healthy donor, RBC DNA WGS, qPCR detection of *EGFR* and *MT-CO1* DNA fragments	Fig 2, Fig3,Supplemetal Fig 6, Supplemetal Fig 8
HD4	GL	RBC DNA	N/A	N/A	N/A	healthy donor, qPCR detection of *EGFR* and *MT-CO1* DNA fragments	Supplemetal Fig 6, Supplemetal Fig 8
HD5	GL	RBC DNA	N/A	N/A	N/A	healthy donor, qPCR detection of *EGFR* and *MT-CO1* DNA fragments	Supplemetal Fig 6, Supplemetal Fig 8
BN1	GL	RBC DNA	N/A	N/A	N/A	patient with non-cancerous benign nodules, RBC DNA WGS	Fig 2, Fig3
BN2	GL	RBC DNA	N/A	N/A	N/A	patient with non-cancerous benign nodules, RBC DNA WGS	Fig 2, Fig3
BN3	GL	RBC DNA	N/A	N/A	N/A	patient with non-cancerous benign nodules, RBC DNA WGS	Fig 2, Fig3
BN4	GL	RBC DNA	N/A	N/A	N/A	patient with non-cancerous benign nodules, RBC DNA WGS, qPCR detection of *EGFR* and *MT-CO1* DNA fragments	Fig 2, Fig3,Supplemetal Fig 6, Supplemetal Fig 8
BN5	GL	RBC DNA	N/A	N/A	N/A	patient with non-cancerous benign nodules, RBC DNA WGS, qPCR detection of *EGFR* and *MT-CO1* DNA fragments	Fig 2, Fig3,Supplemetal Fig 6, Supplemetal Fig 8
BN6	GL	RBC DNA	N/A	N/A	N/A	patient with non-cancerous benign nodules, RBC DNA WGS	Fig 2, Fig3
BN7	GL	RBC DNA	N/A	N/A	N/A	patient with non-cancerous benign nodules, qPCR detection of *EGFR* and *MT-CO1* DNA fragments	Supplemetal Fig 6, Supplemetal Fig 8
BN8	GL	RBC DNA	N/A	N/A	N/A	patient with non-cancerous benign nodules, qPCR detection of *EGFR* and *MT-CO1* DNA fragments	Supplemetal Fig 6, Supplemetal Fig 8
BN9	GL	RBC DNA	N/A	N/A	N/A	patient with non-cancerous benign nodules, qPCR detection of *EGFR* and *MT-CO1* DNA fragments	Supplemetal Fig 6, Supplemetal Fig 8
BN10	GL	RBC DNA	N/A	N/A	N/A	patient with non-cancerous benign nodules, qPCR detection of *EGFR* and *MT-CO1* DNA fragments	Supplemetal Fig 6, Supplemetal Fig 8
BN11	GL	RBC DNA	N/A	N/A	N/A	patient with non-cancerous benign nodules, qPCR detection of *EGFR* and *MT-CO1* DNA fragments	Supplemetal Fig 6, Supplemetal Fig 8
BN12	GL	RBC DNA	N/A	N/A	N/A	patient with non-cancerous benign nodules, qPCR detection of *EGFR* and *MT-CO1* DNA fragments	Supplemetal Fig 6, Supplemetal Fig 8
BN13	GL	RBC DNA	N/A	N/A	N/A	patient with non-cancerous benign nodules, qPCR detection of *EGFR* and *MT-CO1* DNA fragments	Supplemetal Fig 6, Supplemetal Fig 8
BN14	GL	RBC DNA	N/A	N/A	N/A	patient with non-cancerous benign nodules, qPCR detection of *EGFR* and *MT-CO1* DNA fragments	Supplemetal Fig 6, Supplemetal Fig 8
BN15	GL	RBC DNA	N/A	N/A	N/A	patient with non-cancerous benign nodules, qPCR detection of *EGFR* and *MT-CO1* DNA fragments	Supplemetal Fig 6, Supplemetal Fig 8
BN16	GL	RBC DNA	N/A	N/A	N/A	patient with non-cancerous benign nodules, qPCR detection of *EGFR* and *MT-CO1* DNA fragments	Supplemetal Fig 6, Supplemetal Fig 8
BN17	GL	RBC DNA	N/A	N/A	N/A	patient with non-cancerous benign nodules, qPCR detection of *EGFR* and *MT-CO1* DNA fragments	Supplemetal Fig 6, Supplemetal Fig 8
BN18	GL	RBC DNA	N/A	N/A	N/A	patient with non-cancerous benign nodules, qPCR detection of *EGFR* and *MT-CO1* DNA fragments	Supplemetal Fig 6, Supplemetal Fig 8
BN19	GL	RBC DNA	N/A	N/A	N/A	patient with non-cancerous benign nodules, qPCR detection of *EGFR* and *MT-CO1* DNA fragments	Supplemetal Fig 6, Supplemetal Fig 8
LC1	GL	RBC DNA	LUAD	IA1	T1a,N0,cM0	patient with Lung Cancer, RBC DNA WGS, qPCR detection of *EGFR* and *MT-CO1* DNA fragments	Fig 2, Fig3,Supplemetal Fig 6, Supplemetal Fig 8
LC2	GL	RBC DNA	LUAD	IA3	T1c,N0,cM0	patient with Lung Cancer, RBC DNA WGS	Fig 2, Fig3
LC3	GL	RBC DNA	LUAD	IA2	T1b,N0,cM0	patient with Lung Cancer, RBC DNA WGS	Fig 2, Fig3
LC4	GL	RBC DNA	LUAD	IB	T2a,N0,cM0	patient with Lung Cancer, RBC DNA WGS, qPCR detection of *EGFR* and *MT-CO1* DNA fragments	Fig 2, Fig3,Supplemetal Fig 6, Supplemetal Fig 8
LC5	GL	RBC DNA	LUSC	IIB	T2b,N0,cM0	patient with Lung Cancer, RBC DNA WGS, qPCR detection of *EGFR* and *MT-CO1* DNA fragments	Fig 2, Fig3,Supplemetal Fig 6, Supplemetal Fig 8
LC6	GL	RBC DNA	LUSC	IB	T2a,N0,cM0	patient with Lung Cancer, RBC DNA WGS, qPCR detection of *EGFR* and *MT-CO1* DNA fragments	Fig 2, Fig3,Supplemetal Fig 6, Supplemetal Fig 8
LC7	GL	RBC DNA	LUAD	IA3	T1c,N0,cM0	patient with Lung Cancer, RBC DNA WGS, qPCR detection of *EGFR* and *MT-CO1* DNA fragments	Fig 2, Fig3,Supplemetal Fig 6, Supplemetal Fig 8
LC8	GL	RBC DNA	LUSC	IIB	T2b,N1,cM0	patient with Lung Cancer, RBC DNA WGS	Fig 2, Fig3
LC9	GL	RBC DNA	LUSC	IIA	T2b,N0,cM0	patient with Lung Cancer, RBC DNA WGS	Fig 2, Fig3
LC10	GL	RBC DNA	LUAD	IIIA	T1b,N2,cM0	patient with Lung Cancer, RBC DNA WGS	Fig 2, Fig3
LC11	GL	RBC DNA	LUAD	IIB	T1b,N1,cM0	patient with Lung Cancer, RBC DNA WGS	Fig 2, Fig3
LC12	GL	RBC DNA	LUSC	IB	T2a,N0,cM0	patient with Lung Cancer, qPCR detection of *EGFR* and *MT-CO1* DNA fragments	Supplemetal Fig 6, Supplemetal Fig 8
LC13	GL	RBC DNA	LUAD	IA1	T1mi,N0,cM0	patient with Lung Cancer, qPCR detection of *EGFR* and *MT-CO1* DNA fragments	Supplemetal Fig 6, Supplemetal Fig 8
LC14	GL	RBC DNA	LUAD	IA1	T1mi,Nx,cM0	patient with Lung Cancer, qPCR detection of *EGFR* and *MT-CO1* DNA fragments	Supplemetal Fig 6, Supplemetal Fig 8
LC15	GL	RBC DNA	LUAD	IA2	T1b(m),N0,cM0	patient with Lung Cancer, qPCR detection of *EGFR* and *MT-CO1* DNA fragments	Supplemetal Fig 6, Supplemetal Fig 8
LC16	GL	RBC DNA	LUAD	IB	T2a,N0,cM0	patient with Lung Cancer, qPCR detection of *EGFR* and *MT-CO1* DNA fragments	Supplemetal Fig 6, Supplemetal Fig 8
LC17	GL	RBC DNA	LUAD	in situ	Tis,Nx,cM0	patient with Lung Cancer, qPCR detection of *EGFR* and *MT-CO1* DNA fragments	Supplemetal Fig 6, Supplemetal Fig 8
LC18	GL	RBC DNA	LUAD	IA2	T1b,N0,cM0	patient with Lung Cancer, qPCR detection of *EGFR* and *MT-CO1* DNA fragments	Supplemetal Fig 6, Supplemetal Fig 8
LC19	GL	RBC DNA	LUAD	in situ	Tis,Nx,cM0	patient with Lung Cancer, qPCR detection of *EGFR* and *MT-CO1* DNA fragments	Supplemetal Fig 6, Supplemetal Fig 8
LC20	GL	RBC DNA	LUAD	in situ	Tis,Nx,cM0	patient with Lung Cancer, qPCR detection of *EGFR* and *MT-CO1* DNA fragments	Supplemetal Fig 6, Supplemetal Fig 8
LC21	GL	RBC DNA	LUAD	IA3	T1c,N0,cM0	patient with Lung Cancer, qPCR detection of *EGFR* and *MT-CO1* DNA fragments	Supplemetal Fig 6, Supplemetal Fig 8
LC22	GL	RBC DNA	LUAD	IA1	T1mi,N0,cM0	patient with Lung Cancer, qPCR detection of *EGFR* and *MT-CO1* DNA fragments	Supplemetal Fig 6, Supplemetal Fig 8
LC23	GL	RBC DNA	LUAD	IA2	T1b,N0,cM0	patient with Lung Cancer, qPCR detection of *EGFR* and *MT-CO1* DNA fragments	Supplemetal Fig 6, Supplemetal Fig 8
LC24	GL	RBC DNA	LUAD	in situ	Tis,N0,cM0	patient with Lung Cancer, qPCR detection of *EGFR* and *MT-CO1* DNA fragments	Supplemetal Fig 6, Supplemetal Fig 8
LC25	GL	RBC DNA	LUSC	IB	T2a,N0,cM0	patient with Lung Cancer, qPCR detection of *EGFR* and *MT-CO1* DNA fragments	Supplemetal Fig 6, Supplemetal Fig 8
LC26	GL	RBC DNA	LUAD	IA1	T1mi,N0,cM0	patient with Lung Cancer, qPCR detection of *EGFR* and *MT-CO1* DNA fragments	Supplemetal Fig 6, Supplemetal Fig 8
LC27	GL	RBC DNA	LUAD	in situ	Tis,N0,cM0	patient with Lung Cancer, qPCR detection of *EGFR* and *MT-CO1* DNA fragments	Supplemetal Fig 6, Supplemetal Fig 8
LC28	GL	RBC DNA	LUAD	IA2	T1b,N0,cM0	patient with Lung Cancer, qPCR detection of *EGFR* and *MT-CO1* DNA fragments	Supplemetal Fig 6, Supplemetal Fig 8
LC29	GL	RBC DNA	LUAD	IIB	T2b,N1,cM0	patient with Lung Cancer, qPCR detection of *EGFR* and *MT-CO1* DNA fragments	Supplemetal Fig 6, Supplemetal Fig 8
LC30	GL	RBC DNA	LUAD	IIIA	T1c,N2,cM0	patient with Lung Cancer, qPCR detection of *EGFR* and *MT-CO1* DNA fragments	Supplemetal Fig 6, Supplemetal Fig 8
LC31	GL	RBC DNA	LUAD	IA1	T1a,N0,cM0	patient with Lung Cancer, qPCR detection of *EGFR* and *MT-CO1* DNA fragments	Supplemetal Fig 6, Supplemetal Fig 8
LC32	GL	RBC DNA	LUAD	IA1	T1mi,Nx,cM0	patient with Lung Cancer, absolute quantification of *EGFR* and *GAPDH* DNA fragments using qPCR	Supplemetal Fig 9
LC33	GL	RBC DNA	LUAD	IA2	T1b,N0,cM0	patient with Lung Cancer, absolute quantification of *EGFR* and *GAPDH* DNA fragments using qPCR	Supplemetal Fig 9
LC34	GL	RBC DNA	LUSC	IIIA	T1b,N2,cM0	patient with Lung Cancer, absolute quantification of *EGFR* and *GAPDH* DNA fragments using qPCR	Supplemetal Fig 9
LC35	GL	RBC DNA	LUAD	IA1	T1mi,N0,cM0	patient with Lung Cancer, absolute quantification of *EGFR* and *GAPDH* DNA fragments using qPCR	Supplemetal Fig 9
LC36	GL	RBC DNA	LUAD	IA1	T1mi,N0,cM0	patient with Lung Cancer, absolute quantification of *EGFR* and *GAPDH* DNA fragments using qPCR	Supplemetal Fig 9
LC37	GL	RBC DNA	LUAD	IA1	T1mi,N0,cM0	patient with Lung Cancer, absolute quantification of *EGFR* and *GAPDH* DNA fragments using qPCR	Supplemetal Fig 9
LC38	GL	RBC DNA	LUAD	IA3	T1c,N0,cM0	patient with Lung Cancer, absolute quantification of *EGFR* and *GAPDH* DNA fragments using qPCR	Supplemetal Fig 9
LC39	GL	RBC DNA	LUAD	IA2	T1b,N0,cM0	patient with Lung Cancer, absolute quantification of *EGFR* and *GAPDH* DNA fragments using qPCR	Supplemetal Fig 9
LC40	GL	RBC DNA	LUAD	IA2	T1b,N0,cM0	patient with Lung Cancer, absolute quantification of *EGFR* and *GAPDH* DNA fragments using qPCR	Supplemetal Fig 9
LC41	GL	RBC DNA	LUAD	IIIA	T2a,N2,cM0	patient with Lung Cancer, absolute quantification of *EGFR* and *GAPDH* DNA fragments using qPCR	Supplemetal Fig 9
LC42	GL	RBC DNA	LUAD	IIB	T1c,N1,cM0	patient with Lung Cancer, absolute quantification of *EGFR* and *GAPDH* DNA fragments using qPCR	Supplemetal Fig 9
LC43	GL	RBC DNA	LUAD	IA3	T1c,N0,cM0	patient with Lung Cancer, absolute quantification of *EGFR* and *GAPDH* DNA fragments using qPCR	Supplemetal Fig 9
LC44	GL	RBC DNA	LUAD	IA2	T1b,N0,cM0	patient with Lung Cancer, Detection of RBC DNA mutations by NGS	Fig 5
LC45	GL	RBC DNA	LUAD	IA2	T1b,N0,cM0	patient with Lung Cancer, Detection of RBC DNA mutations by NGS	Fig 5
LC46	GL	RBC DNA	LUAD	IIIA	T2a,N2,cM0	patient with Lung Cancer, Detection of RBC DNA mutations by NGS	Fig 5
LC47	GL	RBC DNA	LUAD	IA1	T1mi,N0,cM0	patient with Lung Cancer, Detection of RBC DNA mutations by NGS	Fig 5
LC48	GL	RBC DNA	LUAD	IB	T2a,N0,cM0	patient with Lung Cancer, Detection of RBC DNA mutations by NGS	Fig 5
LC49	GL	RBC DNA	LUSC	IIB	T3,N0,cM0	patient with Lung Cancer, Detection of RBC DNA mutations by NGS	Fig 5
LC50	GL	RBC DNA	LUAD	IA3	T1c,N0,Mx	patient with Lung Cancer, Detection of RBC DNA mutations by NGS	Fig 5
LC51	GL	RBC DNA	LUSC	IIB	T1c,N1,cM0	patient with Lung Cancer, Detection of RBC DNA mutations by NGS	Fig 5
LC52	GL	RBC DNA	LUAD	IB	T2a,N0,cM0	patient with Lung Cancer, Detection of RBC DNA mutations by NGS	Fig 5
LC53	GL	RBC DNA	LUAD	IA3	T1c,N1,cM0	patient with Lung Cancer, Detection of RBC DNA mutations by NGS	Fig 5
LC54	GL	RBC DNA	LUAD	IB	T2a,N0,cM0	patient with Lung Cancer, Detection of RBC DNA mutations by NGS	Fig 5
LC55	GL	RBC DNA	LUAD	IA2	T1b,N0,cM0	patient with Lung Cancer, Detection of RBC DNA mutations by NGS	Fig 5
LC56	GL	RBC DNA	LUSC	IIIB	T3,N2,cM0	patient with Lung Cancer, Detection of RBC DNA mutations by NGS and ddPCR	Fig 5
LC57	XH	RBC DNA	LUAD	IA1	T1a,N0,M0	patient with Lung Cancer, Detection of RBC DNA mutations by NGS	Fig 5
LC58	XH	RBC DNA	LUAD	IA1	T1a,N0,M0	patient with Lung Cancer, Detection of RBC DNA mutations by NGS	Fig 5
LC59	XH	RBC DNA	LUAD	IB	T2a,N0,M0	patient with Lung Cancer, Detection of RBC DNA mutations by NGS	Fig 5
LC60	XH	RBC DNA	LUAD	IA2	T1b,N0,M0	patient with Lung Cancer, Detection of RBC DNA mutations by NGS	Fig 5
LC61	XH	RBC DNA	LUAD	IA2	T1b,N0,M0	patient with Lung Cancer, Detection of RBC DNA mutations by NGS	Fig 5
LC62	XH	RBC DNA	LUAD	IB	T2a,N0,M0	patient with Lung Cancer, Detection of RBC DNA mutations by NGS	Fig 5
LC63	XH	RBC DNA	LUAD	IA3	T1c,N1,M0	patient with Lung Cancer, Detection of RBC DNA mutations by NGS	Fig 5
LC64	XH	RBC DNA	LUAD	IA2	T1b,N0,M0	patient with Lung Cancer, Detection of RBC DNA mutations by NGS	Fig 5
LC65	XH	RBC DNA	LUAD	IIIA	T1b,N2,M0	patient with Lung Cancer, Detection of RBC DNA mutations by NGS	Fig 5
LC66	XH	RBC DNA	LUAD	IA2	T1b,N0,M0	patient with Lung Cancer, Detection of RBC DNA mutations by NGS	Fig 5
LC67	XH	RBC DNA	LUAD	IA1	T1a,N0,M1	patient with Lung Cancer, Detection of RBC DNA mutations by NGS	Fig 5
LC68	XH	RBC DNA	LUAD	IA2	T1b,N0,M0	patient with Lung Cancer, Detection of RBC DNA mutations by NGS	Fig 5
LC69	XH	RBC DNA	LUAD	IB	T2a,N0,M0	patient with Lung Cancer, Detection of RBC DNA mutations by NGS	Fig 5
LC70	GL	RBC DNA	LUAD	IA2	T1b,Nx,cM0	patient with Lung Cancer, Detection of RBC DNA mutations by ddPCR	Fig 5
LC71	GL	RBC DNA	LUAD	IA2	T1b,N0,cM0	patient with Lung Cancer, Detection of RBC DNA mutations by ddPCR	Fig 5
LC72	GL	RBC DNA	LUAD	IIA	T2b,N0,cM0	patient with Lung Cancer, Detection of RBC DNA mutations by ddPCR	Fig 5
LC73	GL	RBC DNA	LUAD	IB	T2a,N0,cM0	patient with Lung Cancer, Detection of RBC DNA mutations by ddPCR	Fig 5
LC74	GL	RBC DNA	LUAD	IA1	T1mi,Nx,cM0	patient with Lung Cancer, Detection of RBC DNA mutations by ddPCR	Fig 5
LC75	GL	RBC DNA	LUAD	IA2	T1b,N0,cM0	patient with Lung Cancer, Detection of RBC DNA mutations by ddPCR	Fig 5
LC76	GL	RBC DNA	LUAD	IB	T2a,N0,cM0	patient with Lung Cancer, Detection of RBC DNA mutations by ddPCR	Fig 5
LC77	GL	RBC DNA	LUAD	IA3	T1c,N0,cM0	patient with Lung Cancer, Detection of RBC DNA mutations by ddPCR	Fig 5
TT1	XH	tumor tissue DNA	LUAD	IA1	T1mi,N0,cM0	patient with Lung Cancer, qPCR detection of *EGFR* and *MT-CO1* DNA fragments	Supplemetal Fig 6, Supplemetal Fig 8
TT2	XH	tumor tissue DNA	LUAD	IA1	T1mi,N0,cM0	patient with Lung Cancer, qPCR detection of *EGFR* and *MT-CO1* DNA fragments	Supplemetal Fig 6, Supplemetal Fig 8
TT3	XH	tumor tissue DNA	LUAD	IIIA	T1c,N2,cM0	patient with Lung Cancer, qPCR detection of *EGFR* and *MT-CO1* DNA fragments	Supplemetal Fig 6, Supplemetal Fig 8
TT4	XH	tumor tissue DNA	LUAD	IA2	T1b,N0,cM0	patient with Lung Cancer, qPCR detection of *EGFR* and *MT-CO1* DNA fragments	Supplemetal Fig 6, Supplemetal Fig 8
NT1	XH	normal tissue DNA	N/A	N/A	N/A	Paracancerous tissue in cancer patient TT2, qPCR detection of *EGFR* and *MT-CO1* DNA fragments	Supplemetal Fig 6, Supplemetal Fig 8
NT2	XH	normal tissue DNA	N/A	N/A	N/A	Paracancerous tissue in cancer patient TT3, qPCR detection of *EGFR* and *MT-CO1* DNA fragments	Supplemetal Fig 6, Supplemetal Fig 8
NT3	XH	normal tissue DNA	N/A	N/A	N/A	Paracancerous tissue in cancer patient TT4, qPCR detection of *EGFR* and *MT-CO1* DNA fragments	Supplemetal Fig 6, Supplemetal Fig 8
NT4	XH	normal tissue DNA	N/A	N/A	N/A	Paracancerous tissue in cancer patient, qPCR detection of *EGFR* and *MT-CO1* DNA fragments	Supplemetal Fig 6, Supplemetal Fig 8
NT5	XH	normal tissue DNA	N/A	N/A	N/A	Paracancerous tissue in cancer patient, qPCR detection of *EGFR* and *MT-CO1* DNA fragments	Supplemetal Fig 6, Supplemetal Fig 8
NT6	XH	normal tissue DNA	N/A	N/A	N/A	Paracancerous tissue in cancer patient, qPCR detection of *EGFR* and *MT-CO1* DNA fragments	Supplemetal Fig 6, Supplemetal Fig 8

HD: healthy donor; BN: benign; LC: lung cancer. XH: Peking Union Medical College Hospital; GL: Nanjing Drum Tower Hospital. LUAD: Lung adenocarcinoma; LUSC: Lung squamous carcinoma.

Supplemental Table 2. Screened DNA regions with differential copy number variation

Region	CHR	start	end	HD2	HD3	HD1	BN1	BN2	BN3	BN4	BN5	BN6	LC1	LC2	LC3	LC4	LC5	LC6	LC7	LC8	LC9	LC10	LC10*	LC11
chr9:41234001	chr9	41,234,001	41,235,000	5	5	5	4	5	4	4	5	5	5	5	5	5	5	5	5	5	5	5	5	5
chr3:75668001	chr3	75,668,001	75,669,000	5	5	5	5	4	4	3	4	4	5	5	4	5	5	4	5	5	5	5	5	5
chr3:75669001	chr3	75,669,001	75,670,000	5	5	5	5	4	4	3	4	4	5	5	4	5	5	4	5	5	5	5	5	5
chr9:63828001	chr9	63,828,001	63,829,000	5	5	5	5	4	4	3	4	3	5	5	5	4	5	4	5	5	5	5	4	5
chr10:133642001	chr10	133,642,001	133,643,000	4	4	3	5	5	5	5	5	4	4	4	5	5	5	5	5	5	5	5	5	4
chr10:133643001	chr10	133,643,001	133,644,000	4	4	3	5	5	5	5	5	4	4	4	5	5	5	5	5	5	5	5	5	4
chr10:133644001	chr10	133,644,001	133,645,000	4	4	3	5	5	5	5	5	4	4	4	5	5	5	5	5	5	5	5	5	4
chr10:133645001	chr10	133,645,001	133,646,000	4	4	3	5	5	5	5	5	4	4	4	5	5	5	5	5	5	5	5	5	4
chr10:133646001	chr10	133,646,001	133,647,000	4	4	3	5	5	5	5	5	4	4	4	5	5	5	5	5	5	5	5	5	4
chr4:190095001	chr4	190,095,001	190,096,000	5	5	5	4	5	3	3	3	4	5	5	5	4	5	4	5	4	4	5	5	5
chr4:190096001	chr4	190,096,001	190,097,000	5	5	5	4	5	3	3	3	4	5	5	5	4	5	4	5	4	4	5	5	5
chr4:190097001	chr4	190,097,001	190,098,000	5	5	5	4	5	3	3	3	4	5	5	5	4	5	4	5	4	4	5	5	5
chr4:190098001	chr4	190,098,001	190,099,000	5	5	5	4	5	3	3	3	4	5	5	5	4	5	4	5	4	4	5	5	5
chr4:190099001	chr4	190,099,001	190,100,000	5	5	5	4	5	3	3	3	4	5	5	5	4	5	4	5	4	4	5	5	5
chr4:190100001	chr4	190,100,001	190,101,000	5	5	5	4	5	3	3	3	4	5	5	5	4	5	4	5	4	4	5	5	5
chr4:190101001	chr4	190,101,001	190,102,000	5	5	5	4	5	3	3	3	4	5	5	5	4	5	4	5	4	4	5	5	5
chr4:190102001	chr4	190,102,001	190,103,000	5	5	5	4	5	3	3	3	4	5	5	5	4	5	4	5	4	4	5	5	5
chr4:190103001	chr4	190,103,001	190,104,000	5	5	5	4	5	3	3	3	4	5	5	5	4	5	4	5	4	4	5	5	5
chr4:190104001	chr4	190,104,001	190,105,000	5	5	5	4	5	3	3	3	4	5	5	5	4	5	4	5	4	4	5	5	5
chr4:190105001	chr4	190,105,001	190,106,000	5	5	5	4	5	3	3	3	4	5	5	5	4	5	4	5	4	4	5	5	5
chr4:190106001	chr4	190,106,001	190,107,000	5	5	5	4	5	3	3	3	4	5	5	5	4	5	4	5	4	4	5	5	5
chr4:190107001	chr4	190,107,001	190,108,000	5	5	5	4	5	3	3	3	4	5	5	5	4	5	4	5	4	4	5	5	5
chr4:190108001	chr4	190,108,001	190,109,000	5	5	5	4	5	3	2	3	4	5	5	5	4	5	4	5	4	4	5	5	5
chr4:190109001	chr4	190,109,001	190,110,000	5	5	5	4	5	3	2	3	4	5	5	5	4	5	4	5	4	4	5	5	5
chr4:190110001	chr4	190,110,001	190,111,000	5	5	5	4	5	3	3	3	4	5	5	5	4	5	4	5	4	4	5	5	5
chr4:190111001	chr4	190,111,001	190,112,000	5	5	5	4	5	3	3	3	4	5	5	5	4	5	4	5	4	4	5	5	5
chr4:190087001	chr4	190,087,001	190,088,000	5	5	5	4	3	2	1	3	3	5	5	5	4	5	3	5	4	5	5	4	5
chr4:190088001	chr4	190,088,001	190,089,000	5	5	5	4	3	2	1	3	3	5	5	5	4	5	3	5	4	5	5	4	5
chr4:190089001	chr4	190,089,001	190,090,000	5	5	5	4	3	2	1	3	3	5	5	5	4	5	3	5	4	5	5	4	5
chr4:190090001	chr4	190,090,001	190,091,000	5	5	5	4	3	2	1	3	3	5	5	5	4	5	3	5	4	5	5	4	5
chr4:190091001	chr4	190,091,001	190,092,000	5	5	5	4	3	2	1	3	3	5	5	5	4	5	3	5	4	5	5	4	5
chr4:190092001	chr4	190,092,001	190,093,000	5	5	5	4	3	3	1	3	3	5	5	5	4	5	3	5	4	5	5	4	5
chr4:190093001	chr4	190,093,001	190,094,000	5	5	5	4	5	3	3	3	3	5	5	5	4	5	4	5	4	4	5	4	5
chr4:190094001	chr4	190,094,001	190,095,000	5	5	5	4	5	3	3	3	4	5	5	5	4	5	4	5	4	4	5	4	5
chr17:21928001	chr17	21,928,001	21,929,000	5	5	5	4	4	3	3	4	4	5	5	5	3	4	5	5	4	5	5	5	4
chr10:125906001	chr10	125,906,001	125,907,000	3	3	4	5	5	4	4	4	4	5	5	4	5	4	4	5	4	5	5	5	4
chr1:16661001	chr1	16,661,001	16,662,000	3	4	3	4	5	5	4	5	4	4	4	5	5	5	5	4	5	5	4	4	5
chr10:133641001	chr10	133,641,001	133,642,000	3	4	3	5	5	5	5	5	4	3	4	5	5	5	5	5	5	4	5	5	4
chr4:49263001	chr4	49,263,001	49,264,000	5	5	5	4	3	3	2	3	4	5	5	4	4	5	2	5	4	5	5	5	5
chr4:49264001	chr4	49,264,001	49,265,000	5	5	5	4	3	3	2	3	4	5	5	4	4	5	2	5	4	5	5	5	5
chr4:49265001	chr4	49,265,001	49,266,000	5	5	5	4	3	3	2	3	4	5	5	4	4	5	2	5	4	5	5	5	5
chr4:49266001	chr4	49,266,001	49,267,000	5	5	5	4	3	3	1	3	4	5	5	4	4	5	2	5	4	5	5	5	5
chr4:49267001	chr4	49,267,001	49,268,000	5	5	5	4	3	3	1	3	4	5	5	4	4	5	2	5	4	5	5	5	5
chr4:49268001	chr4	49,268,001	49,269,000	5	5	5	4	3	3	1	3	4	5	5	4	4	5	2	5	4	5	5	5	5
chr4:49269001	chr4	49,269,001	49,270,000	5	5	5	4	3	3	1	3	4	5	5	4	4	5	2	5	4	5	5	5	5
chr4:49270001	chr4	49,270,001	49,271,000	5	5	5	4	3	3	1	3	4	5	5	4	4	5	2	5	4	5	5	5	5
chr4:190076001	chr4	190,076,001	190,077,000	5	5	5	4	3	2	1	2	3	5	5	4	4	5	3	5	4	5	5	4	5
chr4:190077001	chr4	190,077,001	190,078,000	5	5	5	4	3	2	1	2	3	5	5	4	4	5	3	5	4	5	5	4	5
chr4:190078001	chr4	190,078,001	190,079,000	5	5	5	4	3	2	1	2	3	5	5	4	4	5	3	5	4	5	5	4	5
chr4:190079001	chr4	190,079,001	190,080,000	5	5	5	4	3	2	1	2	3	5	5	4	4	5	3	5	4	5	5	4	5
chr4:190080001	chr4	190,080,001	190,081,000	5	5	5	4	3	2	1	2	3	5	5	4	4	5	3	5	4	5	5	4	5
chr4:190085001	chr4	190,085,001	190,086,000	5	5	5	4	3	2	1	3	3	5	5	4	4	5	3	5	4	5	5	4	5
chr4:190086001	chr4	190,086,001	190,087,000	5	5	5	4	3	2	1	3	3	5	5	4	4	5	3	5	4	5	5	4	5
chr10:38882001	chr10	38,882,001	38,883,000	5	5	5	5	4	2	1	3	3	5	5	4	3	4	3	5	5	5	5	5	5
chr10:38889001	chr10	38,889,001	38,890,000	5	5	5	5	4	2	1	3	3	5	5	4	3	4	3	5	5	5	5	5	5
chr10:38897001	chr10	38,897,001	38,898,000	5	5	5	5	4	2	2	3	3	5	5	4	3	4	3	5	5	5	5	5	5
chr17:21888001	chr17	21,888,001	21,889,000	5	5	5	4	4	3	3	4	4	5	5	5	3	4	4	5	4	5	5	5	4
chr17:21889001	chr17	21,889,001	21,890,000	5	5	5	4	4	3	3	4	4	5	5	5	3	4	4	5	4	5	5	5	4
chr17:21929001	chr17	21,929,001	21,930,000	5	5	5	4	4	3	3	4	4	5	5	5	3	4	4	5	4	5	5	5	4
chr17:21930001	chr17	21,930,001	21,931,000	5	5	5	4	4	3	3	4	4	5	5	5	3	4	4	5	4	5	5	5	4
chr17:21931001	chr17	21,931,001	21,932,000	5	5	5	4	4	3	3	4	4	5	5	5	3	4	4	5	4	5	5	5	4
chr17:21932001	chr17	21,932,001	21,933,000	5	5	5	4	4	3	3	4	4	5	5	5	3	4	4	5	4	5	5	5	4
chr17:21933001	chr17	21,933,001	21,934,000	5	5	5	4	4	3	3	4	4	5	5	5	3	4	4	5	4	5	5	5	4
chr19:50109001	chr19	50,109,001	50,110,000	5	5	5	4	4	4	4	2	4	5	5	4	5	4	4	4	5	5	4	4	5
chr19:50110001	chr19	50,110,001	50,111,000	5	5	5	4	4	4	4	2	4	5	5	4	5	4	4	4	5	5	4	4	5
chr19:50111001	chr19	50,111,001	50,112,000	5	5	5	4	4	4	4	2	4	5	5	4	5	4	4	4	5	5	4	4	5
chr19:50112001	chr19	50,112,001	50,113,000	5	5	5	4	4	4	4	2	4	5	5	4	5	4	4	4	5	5	4	4	5
chr19:50113001	chr19	50,113,001	50,114,000	5	5	5	4	4	4	4	2	4	5	5	4	5	4	4	4	5	5	4	4	5
chr19:50114001	chr19	50,114,001	50,115,000	5	5	5	4	4	4	4	2	4	5	5	4	5	4	4	4	5	5	4	4	5
chr19:50115001	chr19	50,115,001	50,116,000	5	5	5	4	4	4	4	2	4	5	5	4	5	4	4	4	5	5	4	4	5
chr19:50116001	chr19	50,116,001	50,117,000	5	5	5	4	4	4	4	2	4	5	5	4	5	4	4	4	5	5	4	4	5
chr19:50117001	chr19	50,117,001	50,118,000	5	5	5	4	4	4	4	2	4	5	5	4	5	4	4	4	5	5	4	4	5
chr19:50118001	chr19	50,118,001	50,119,000	5	5	5	4	4	4	4	2	4	5	5	4	5	4	4	4	5	5	4	4	5
chr19:50119001	chr19	50,119,001	50,120,000	5	5	5	4	4	4	4	3	4	5	5	4	5	4	4	4	5	5	4	4	5
chr19:50120001	chr19	50,120,001	50,121,000	5	5	5	4	4	4	4	3	4	5	5	4	5	4	4	4	5	5	4	4	5
chr1:16662001	chr1	16,662,001	16,663,000	3	4	3	4	5	5	4	5	4	4	4	5	5	5	5	4	5	4	4	4	5
chr1:16663001	chr1	16,663,001	16,664,000	3	4	3	4	5	5	4	5	4	4	4	5	5	5	5	4	5	4	4	4	5
chr1:16664001	chr1	16,664,001	16,665,000	3	4	3	4	5	5	4	5	4	4	4	5	5	5	5	4	5	4	4	4	5
chr1:16665001	chr1	16,665,001	16,666,000	3	4	3	4	5	5	4	5	4	4	4	5	5	5	5	4	5	4	4	4	5
chr1:16666001	chr1	16,666,001	16,667,000	3	4	3	4	5	5	4	5	4	4	4	5	5	5	5	4	5	4	4	4	5
chr1:16667001	chr1	16,667,001	16,668,000	3	4	3	4	5	5	4	5	4	4	4	5	5	5	5	4	5	4	4	4	5
chr1:16668001	chr1	16,668,001	16,669,000	3	4	3	4	5	5	4	5	4	4	4	5	5	5	5	4	5	4	4	4	5
chr4:49252001	chr4	49,252,001	49,253,000	5	5	5	4	3	3	1	3	4	4	5	4	4	5	2	5	4	5	5	5	5
chr4:49253001	chr4	49,253,001	49,254,000	5	5	5	4	3	3	1	3	4	4	5	4	4	5	2	5	4	5	5	5	5
chr4:49254001	chr4	49,254,001	49,255,000	5	5	5	4	3	3	2	3	4	4	5	4	4	5	2	5	4	5	5	5	5
chr4:49255001	chr4	49,255,001	49,256,000	5	5	5	4	3	3	2	3	4	4	5	4	4	5	2	5	4	5	5	5	5
chr4:49256001	chr4	49,256,001	49,257,000	5	5	5	4	3	3	2	3	4	4	5	4	4	5	2	5	4	5	5	5	5
chr4:49257001	chr4	49,257,001	49,258,000	5	5	5	4	3	3	2	3	4	4	5	4	4	5	2	5	4	5	5	5	5
chr4:49258001	chr4	49,258,001	49,259,000	5	5	5	4	3	3	2	3	4	4	5	4	4	5	2	5	4	5	5	5	5
chr4:49259001	chr4	49,259,001	49,260,000	5	5	5	4	3	3	2	3	4	4	5	4	4	5	2	5	4	5	5	5	5
chr4:49260001	chr4	49,260,001	49,261,000	5	5	5	4	3	3	2	3	4	4	5	4	4	5	2	5	4	5	5	5	5
chr4:49261001	chr4	49,261,001	49,262,000	5	5	5	4	3	3	2	3	4	4	5	4	4	5	2	5	4	5	5	5	5
chr4:49262001	chr4	49,262,001	49,263,000	5	5	5	4	3	3	2	3	4	4	5	4	4	5	2	5	4	5	5	5	5
chr4:49271001	chr4	49,271,001	49,272,000	5	5	5	4	3	3	1	3	4	5	5	4	4	5	2	5	4	5	5	5	4
chr4:49272001	chr4	49,272,001	49,273,000	5	5	5	4	3	3	1	3	4	5	5	4	4	5	2	5	4	5	5	5	4
chr4:49273001	chr4	49,273,001	49,274,000	5	5	5	4	3	3	1	3	4	5	5	4	4	5	2	5	4	5	5	5	4
chr4:49274001	chr4	49,274,001	49,275,000	5	5	5	4	3	3	2	3	4	5	5	4	4	5	2	5	4	5	5	5	4
chr4:49275001	chr4	49,275,001	49,276,000	5	5	5	4	3	3	2	3	4	5	5	4	4	5	2	5	4	5	5	5	4
chr4:49276001	chr4	49,276,001	49,277,000	5	5	5	4	3	3	2	3	4	5	5	4	4	5	2	5	4	5	5	5	4
chr4:49277001	chr4	49,277,001	49,278,000	5	5	5	4	3	3	1	3	4	5	5	4	4	5	2	5	4	5	5	5	4
chr4:49334001	chr4	49,334,001	49,335,000	5	5	5	4	3	3	2	3	4	5	5	4	4	5	2	5	4	5	5	5	4
chr4:49335001	chr4	49,335,001	49,336,000	5	5	5	4	3	3	2	3	4	5	5	4	4	5	2	5	4	5	5	5	4
chr4:49336001	chr4	49,336,001	49,337,000	5	5	5	4	3	3	2	3	4	5	5	4	4	5	2	5	4	5	5	5	4
chr4:190067001	chr4	190,067,001	190,068,000	5	5	5	4	3	2	1	2	3	5	5	4	4	5	2	5	4	5	5	4	5
chr4:190068001	chr4	190,068,001	190,069,000	5	5	5	4	3	2	1	2	3	5	5	4	4	5	2	5	4	5	5	4	5
chr4:190069001	chr4	190,069,001	190,070,000	5	5	5	4	3	2	1	2	3	5	5	4	4	5	2	5	4	5	5	4	5
chr4:190070001	chr4	190,070,001	190,071,000	5	5	5	4	3	2	1	2	3	5	5	4	4	5	2	5	4	5	5	4	5
chr4:190071001	chr4	190,071,001	190,072,000	5	5	5	4	3	2	1	2	3	5	5	4	4	5	2	5	4	5	5	4	5
chr4:190072001	chr4	190,072,001	190,073,000	5	5	5	4	3	2	1	2	3	5	5	4	4	5	2	5	4	5	5	4	5
chr4:190073001	chr4	190,073,001	190,074,000	5	5	5	4	3	2	1	2	3	5	5	4	4	5	2	5	4	5	5	4	5
chr4:190074001	chr4	190,074,001	190,075,000	5	5	5	4	3	2	1	2	3	5	5	4	4	5	2	5	4	5	5	4	5

Region	CHR	start	end	HD2	HD3	HD1	BN1	BN2	BN3	BN4	BN5	BN6	LC1	LC2	LC3	LC4	LC5	LC6	LC7	LC8	LC9	LC10	LC10*	LC11
chr4:190075001	chr4	190,075,001	190,076,000	5	5	5	4	3	2	1	2	3	5	5	4	4	5	2	5	4	5	5	4	5
chr4:190081001	chr4	190,081,001	190,082,000	5	5	5	4	3	2	1	2	3	5	5	4	4	5	2	5	4	5	5	4	5
chr4:190082001	chr4	190,082,001	190,083,000	5	5	5	4	3	2	1	2	3	5	5	4	4	5	2	5	4	5	5	4	5
chr4:190083001	chr4	190,083,001	190,084,000	5	5	5	4	3	2	1	2	3	5	5	4	4	5	2	5	4	5	5	4	5
chr4:190084001	chr4	190,084,001	190,085,000	5	5	5	4	3	2	1	3	3	5	5	4	4	5	2	5	4	5	5	4	5
chr5:17517001	chr5	17,517,001	17,518,000	5	4	5	5	2	2	2	2	3	5	4	5	4	4	4	5	5	3	5	4	5
chr5:17518001	chr5	17,518,001	17,519,000	5	4	5	5	2	2	2	3	3	5	4	5	4	4	4	5	5	3	5	4	5
chr5:17519001	chr5	17,519,001	17,520,000	5	4	5	5	2	2	2	3	3	5	4	5	4	4	4	5	5	3	5	4	5
chr5:17520001	chr5	17,520,001	17,521,000	5	4	5	5	2	2	2	3	3	5	4	5	4	4	4	5	5	3	5	4	5
chr5:17521001	chr5	17,521,001	17,522,000	5	4	5	5	2	2	2	3	3	5	4	5	4	4	4	5	5	3	5	4	5
chr5:17522001	chr5	17,522,001	17,523,000	5	4	5	5	2	2	2	3	3	5	4	5	4	4	4	5	5	3	5	4	5
chr5:17523001	chr5	17,523,001	17,524,000	5	4	5	5	2	2	2	3	3	5	4	5	4	4	4	5	5	3	5	4	5
chr5:17524001	chr5	17,524,001	17,525,000	5	4	5	5	2	2	2	3	3	5	4	5	4	4	4	5	5	3	5	4	5
chr5:17525001	chr5	17,525,001	17,526,000	5	4	5	5	2	2	2	3	3	5	4	5	4	4	4	5	5	3	5	4	5
chr5:17526001	chr5	17,526,001	17,527,000	5	4	5	5	2	2	2	3	3	5	4	5	4	4	4	5	5	3	5	4	5
chr5:17527001	chr5	17,527,001	17,528,000	5	4	5	5	2	2	2	3	3	5	4	5	4	4	4	5	5	3	5	4	5
chr5:17528001	chr5	17,528,001	17,529,000	5	4	5	5	2	2	2	3	3	5	4	5	4	4	4	5	5	3	5	4	5
chr5:17529001	chr5	17,529,001	17,530,000	5	4	5	5	2	2	2	3	3	5	4	5	4	4	4	5	5	3	5	4	5
chr9:63815001	chr9	63,815,001	63,816,000	5	5	5	4	4	4	3	4	4	5	5	4	4	4	4	5	4	5	4	4	5
chr9:63829001	chr9	63,829,001	63,830,000	5	5	5	3	3	4	3	4	3	5	5	5	4	3	4	5	3	5	5	4	5
chr10:38922001	chr10	38,922,001	38,923,000	5	5	5	4	4	3	3	3	4	5	5	4	4	4	3	5	4	5	5	4	5
chr17:21873001	chr17	21,873,001	21,874,000	5	5	5	4	3	3	2	4	4	5	4	5	3	4	3	5	4	5	5	5	5
chr17:21971001	chr17	21,971,001	21,972,000	5	5	5	4	4	3	2	3	4	5	5	5	4	4	3	5	3	5	5	5	4
chr17:21974001	chr17	21,974,001	21,975,000	5	5	5	4	4	3	2	3	4	5	5	5	4	4	3	5	3	5	5	5	4
chr17:21975001	chr17	21,975,001	21,976,000	5	5	5	4	4	3	2	3	4	5	5	5	4	4	3	5	3	5	5	5	4
chr17:21976001	chr17	21,976,001	21,977,000	5	5	5	4	4	3	2	3	4	5	5	5	4	4	3	5	3	5	5	5	4
chr17:21977001	chr17	21,977,001	21,978,000	5	5	5	4	4	3	2	3	4	5	5	5	4	4	3	5	3	5	5	5	4
chr17:21978001	chr17	21,978,001	21,979,000	5	5	5	4	4	3	2	3	4	5	5	5	4	4	3	5	3	5	5	5	4
chr17:21979001	chr17	21,979,001	21,980,000	5	5	5	4	4	3	2	3	4	5	5	5	4	4	3	5	3	5	5	5	4
chr17:21980001	chr17	21,980,001	21,981,000	5	5	5	4	4	3	2	3	4	5	5	5	4	4	3	5	3	5	5	5	4
chr17:21981001	chr17	21,981,001	21,982,000	5	5	5	4	4	3	2	3	4	5	5	5	4	4	3	5	3	5	5	5	4
chr17:26858001	chr17	26,858,001	26,859,000	5	5	5	4	3	3	2	3	4	5	4	5	3	4	4	5	4	5	5	5	4
chr17:26859001	chr17	26,859,001	26,860,000	5	5	5	4	3	3	2	3	4	5	4	5	3	4	4	5	4	5	5	5	4
chr17:26860001	chr17	26,860,001	26,861,000	5	5	5	4	3	4	2	3	4	5	4	5	3	4	4	5	4	5	5	5	4
chr17:26861001	chr17	26,861,001	26,862,000	5	5	5	4	3	4	2	3	4	5	4	5	3	4	4	5	4	5	5	5	4
chr19:37294001	chr19	37,294,001	37,295,000	5	5	5	4	2	4	2	2	4	5	5	5	4	4	5	4	3	4	5	4	5
chr19:50121001	chr19	50,121,001	50,122,000	5	5	5	4	4	4	4	3	4	5	4	4	5	4	4	4	5	5	4	4	5
chr19:47908001	chr19	47,908,001	47,909,000	5	5	3	4	3	3	2	2	4	5	5	4	4	4	3	4	4	5	5	5	5
chr4:49159001	chr4	49,159,001	49,160,000	5	5	5	4	4	3	2	3	4	4	4	4	4	5	3	5	4	5	5	4	5
chr4:49251001	chr4	49,251,001	49,252,000	5	5	5	4	3	3	1	3	4	4	5	4	4	5	2	5	4	5	5	5	4
chr4:49278001	chr4	49,278,001	49,279,000	5	5	5	4	3	3	1	2	4	5	5	4	3	5	2	5	4	5	5	5	4
chr4:49279001	chr4	49,279,001	49,280,000	5	5	5	4	3	3	1	2	4	5	5	4	3	5	2	5	4	5	5	5	4
chr4:49280001	chr4	49,280,001	49,281,000	5	5	5	4	3	3	1	2	4	5	5	4	3	5	2	5	4	5	5	5	4
chr4:49281001	chr4	49,281,001	49,282,000	5	5	5	4	3	3	1	2	4	5	5	4	3	5	2	5	4	5	5	5	4
chr4:49282001	chr4	49,282,001	49,283,000	5	5	5	4	3	3	1	2	4	5	5	4	3	5	2	5	4	5	5	5	4
chr4:49283001	chr4	49,283,001	49,284,000	5	5	5	4	3	3	1	2	4	5	5	4	3	5	2	5	4	5	5	5	4
chr4:49284001	chr4	49,284,001	49,285,000	5	5	5	4	3	3	1	2	4	5	5	4	3	5	2	5	4	5	5	5	4
chr4:49285001	chr4	49,285,001	49,286,000	5	5	5	4	3	4	1	2	1	5	5	4	3	5	2	5	4	5	5	5	4
chr4:49289001	chr4	49,289,001	49,290,000	5	5	5	4	3	2	1	2	4	5	5	4	3	5	2	5	4	5	5	5	4
chr4:49290001	chr4	49,290,001	49,291,000	5	5	5	4	3	2	1	2	4	5	5	4	3	5	2	5	4	5	5	5	4
chr4:49291001	chr4	49,291,001	49,292,000	5	5	5	4	3	2	1	2	4	5	5	4	3	5	2	5	4	5	5	5	4
chr4:49292001	chr4	49,292,001	49,293,000	5	5	5	4	3	2	2	2	4	5	5	4	3	5	2	5	4	5	5	5	4
chr4:49316001	chr4	49,316,001	49,317,000	5	5	5	4	3	3	1	2	4	5	5	4	3	5	2	5	4	5	5	5	4
chr4:49317001	chr4	49,317,001	49,318,000	5	5	5	4	3	3	1	2	4	5	5	4	3	5	2	5	4	5	5	5	4
chr4:49318001	chr4	49,318,001	49,319,000	5	5	5	4	3	3	1	2	4	5	5	4	3	5	2	5	4	5	5	5	4
chr4:49319001	chr4	49,319,001	49,320,000	5	5	5	4	3	3	1	2	4	5	5	4	3	5	2	5	4	5	5	5	4
chr4:49320001	chr4	49,320,001	49,321,000	5	5	5	4	3	3	1	2	4	5	5	4	3	5	2	5	4	5	5	5	4
chr4:49321001	chr4	49,321,001	49,322,000	5	5	5	4	3	3	1	2	4	5	5	4	3	5	2	5	4	5	5	5	4
chr4:49322001	chr4	49,322,001	49,323,000	5	5	5	4	3	3	1	2	4	5	5	4	3	5	2	5	4	5	5	5	4
chr4:49323001	chr4	49,323,001	49,324,000	5	5	5	4	3	3	1	2	4	5	5	4	3	5	2	5	4	5	5	5	4
chr4:49325001	chr4	49,325,001	49,326,000	5	5	5	4	3	3	1	2	4	5	5	4	3	5	2	5	4	5	5	5	4
chr4:49326001	chr4	49,326,001	49,327,000	5	5	5	4	3	3	1	2	4	5	5	4	3	5	2	5	4	5	5	5	4
chr4:49327001	chr4	49,327,001	49,328,000	5	5	5	4	3	3	1	2	4	5	5	4	3	5	2	5	4	5	5	5	4
chr4:49328001	chr4	49,328,001	49,329,000	5	5	5	4	3	3	1	2	4	5	5	4	3	5	2	5	4	5	5	5	4
chr4:49329001	chr4	49,329,001	49,330,000	5	5	5	4	3	3	1	2	4	5	5	4	3	5	2	5	4	5	5	5	4
chr4:49330001	chr4	49,330,001	49,331,000	5	5	5	4	3	3	1	2	4	5	5	4	3	5	2	5	4	5	5	5	4
chr9:63783001	chr9	63,783,001	63,784,000	5	5	5	4	4	3	3	3	4	4	4	5	4	4	4	5	4	5	4	4	5
chr9:63784001	chr9	63,784,001	63,785,000	5	5	5	4	4	3	3	3	4	4	4	5	4	4	4	5	4	5	4	4	5
chr9:63785001	chr9	63,785,001	63,786,000	5	5	5	4	4	3	3	3	4	4	4	5	4	4	4	5	4	5	4	4	5
chr9:63786001	chr9	63,786,001	63,787,000	5	5	5	4	4	4	3	3	4	4	4	5	4	4	4	5	4	5	4	4	5
chr9:63787001	chr9	63,787,001	63,788,000	5	5	5	4	4	4	3	3	4	4	4	5	4	4	4	5	4	5	4	4	5
chr9:63788001	chr9	63,788,001	63,789,000	5	5	5	4	4	4	3	3	4	4	4	5	4	4	4	5	4	5	4	4	5
chr9:63789001	chr9	63,789,001	63,790,000	5	5	5	4	4	4	3	4	4	4	4	5	4	4	4	5	4	5	4	4	5
chr9:63790001	chr9	63,790,001	63,791,000	5	5	5	4	4	4	3	4	4	4	4	5	4	4	4	5	4	5	4	4	5
chr9:63791001	chr9	63,791,001	63,792,000	5	5	5	4	4	4	3	4	4	4	4	5	4	4	4	5	4	5	4	4	5
chr9:63792001	chr9	63,792,001	63,793,000	5	5	5	4	4	4	3	4	4	4	4	5	4	4	4	5	4	5	4	4	5
chr9:63811001	chr9	63,811,001	63,812,000	5	5	5	4	4	4	3	4	4	5	4	4	4	4	4	5	4	5	4	4	5
chr9:63812001	chr9	63,812,001	63,813,000	5	5	5	4	4	4	3	4	4	5	4	4	4	4	4	5	4	5	4	4	5
chr9:63813001	chr9	63,813,001	63,814,000	5	5	5	4	4	4	3	4	4	5	4	4	4	4	4	5	4	5	4	4	5
chr9:63814001	chr9	63,814,001	63,815,000	5	5	5	4	4	4	3	4	4	5	4	4	4	4	4	5	4	5	4	4	5
chr9:63830001	chr9	63,830,001	63,831,000	5	5	5	3	3	4	3	3	3	5	5	5	3	3	4	5	3	5	5	4	5
chr10:38923001	chr10	38,923,001	38,924,000	5	5	5	4	4	3	3	3	4	5	4	4	4	4	3	5	4	5	5	4	5
chr10:38924001	chr10	38,924,001	38,925,000	5	5	5	4	4	3	3	3	4	5	4	4	4	4	3	5	4	5	5	4	5
chr10:38925001	chr10	38,925,001	38,926,000	5	5	5	4	4	3	3	3	4	5	4	4	4	4	3	5	4	5	5	4	5
chr17:21853001	chr17	21,853,001	21,854,000	5	5	5	4	4	3	2	3	3	5	4	5	3	4	3	5	4	5	5	5	4
chr17:21854001	chr17	21,854,001	21,855,000	5	5	5	4	4	3	2	3	3	5	4	5	3	4	3	5	4	5	5	5	4
chr17:21855001	chr17	21,855,001	21,856,000	5	5	5	4	4	3	2	3	3	5	4	5	3	4	3	5	4	5	5	5	4
chr17:21856001	chr17	21,856,001	21,857,000	5	5	5	4	4	3	2	3	3	5	4	5	3	4	3	5	4	5	5	5	4
chr17:21857001	chr17	21,857,001	21,858,000	5	5	5	4	4	3	2	3	3	5	4	5	3	4	3	5	4	5	5	5	4
chr17:21858001	chr17	21,858,001	21,859,000	5	5	5	4	4	3	2	3	3	5	4	5	3	4	3	5	4	5	5	5	4
chr17:21859001	chr17	21,859,001	21,860,000	5	5	5	4	4	3	2	3	3	5	4	5	3	4	3	5	4	5	5	5	4
chr17:21860001	chr17	21,860,001	21,861,000	5	5	5	4	4	3	2	3	3	5	4	5	3	4	3	5	4	5	5	5	4
chr17:21861001	chr17	21,861,001	21,862,000	5	5	5	4	4	3	2	3	3	5	4	5	3	4	3	5	4	5	5	5	4
chr17:26783001	chr17	26,783,001	26,784,000	5	5	5	4	3	3	2	3	3	5	4	5	3	4	3	5	4	5	5	5	4
chr17:26784001	chr17	26,784,001	26,785,000	5	5	5	4	3	3	2	3	3	5	4	5	3	4	3	5	4	5	5	5	4
chr17:26785001	chr17	26,785,001	26,786,000	5	5	5	4	3	3	2	3	3	5	4	5	3	4	3	5	4	5	5	5	4
chr17:26786001	chr17	26,786,001	26,787,000	5	5	5	4	3	3	2	3	3	5	4	5	3	4	3	5	4	5	5	5	4
chr17:26787001	chr17	26,787,001	26,788,000	5	5	5	4	3	3	2	3	3	5	4	5	3	4	3	5	4	5	5	5	4
chr17:26788001	chr17	26,788,001	26,789,000	5	5	5	4	3	3	2	3	3	5	4	5	3	4	3	5	4	5	5	5	4
chr17:26789001	chr17	26,789,001	26,790,000	5	5	5	4	3	3	2	3	3	5	4	5	3	4	3	5	4	5	5	5	4
chr17:26790001	chr17	26,790,001	26,791,000	5	5	5	4	3	3	2	3	3	5	4	5	3	4	3	5	4	5	5	5	4
chr17:26834001	chr17	26,834,001	26,835,000	5	5	5	4	3	3	2	3	3	5	4	5	3	4	3	5	4	5	5	5	4
chr17:26835001	chr17	26,835,001	26,836,000	5	5	5	4	3	3	2	3	3	5	4	5	3	4	3	5	4	5	5	5	4
chr17:26836001	chr17	26,836,001	26,837,000	5	5	5	4	3	3	2	3	3	5	4	5	3	4	3	5	4	5	5	5	4
chr17:26837001	chr17	26,837,001	26,838,000	5	5	5	4	3	3	2	3	3	5	4	5	3	4	3	5	4	5	5	5	4
chr17:26838001	chr17	26,838,001	26,839,000	5	5	5	4	3	3	2	3	3	5	4	5	3	4	3	5	4	5	5	5	4
chr17:26839001	chr17	26,839,001	26,840,000	5	5	5	4	3	3	2	3	3	5	4	5	3	4	3	5	4	5	5	5	4
chr17:26840001	chr17	26,840,001	26,841,000	5	5	5	4	3	3	2	3	3	5	4	5	3	4	3	5	4	5	5	5	4
chr17:26841001	chr17	26,841,001	26,842,000	5	5	5	4	3	3	2	3	3	5	4	5	3	4	3	5	4	5	5	5	4
chr17:26842001	chr17	26,842,001	26,843,000	5	5	5	4	3	3	2	3	3	5	4	5	3	4	3	5	4	5	5	5	4
chr17:26843001	chr17	26,843,001	26,844,000	5	5	5	4	3	3	2	3	3	5	4	5	3	4	3	5	4	5	5	5	4
chr17:26844001	chr17	26,844,001	26,845,000	5	5	5	4	3	3	2	3	3	5	4	5	3	4	3	5	4	5	5	5	4
chr17:26845001	chr17	26,845,001	26,846,000	5	5	5	4	3	3	2	3	3	5	4	5	3	4	3	5	4	5	5	5	4

Region	CHR	start	end	HD2	HD3	HD1	BN1	BN2	BN3	BN4	BN5	BN6	LC1	LC2	LC3	LC4	LC5	LC6	LC7	LC8	LC9	LC10	LC10*	LC11
chr17:26846001	chr17	26,846,001	26,847,000	5	5	5	4	3	3	2	3	3	5	4	5	3	4	3	5	4	5	5	5	4
chr17:26847001	chr17	26,847,001	26,848,000	5	5	5	4	3	3	2	3	3	5	4	5	3	4	3	5	4	5	5	5	4
chr17:26848001	chr17	26,848,001	26,849,000	5	5	5	4	3	3	2	3	3	5	4	5	3	4	3	5	4	5	5	5	4
chr17:26849001	chr17	26,849,001	26,850,000	5	5	5	4	3	3	2	3	4	5	4	5	3	4	3	5	4	5	5	5	4
chr17:26850001	chr17	26,850,001	26,851,000	5	5	5	4	3	3	2	3	4	5	4	5	3	4	3	5	4	5	5	5	4
chr17:26851001	chr17	26,851,001	26,852,000	5	5	5	4	3	3	2	3	4	5	4	5	3	4	3	5	4	5	5	5	4
chr17:26852001	chr17	26,852,001	26,853,000	5	5	5	4	3	3	2	3	4	5	4	5	3	4	3	5	4	5	5	5	4
chr17:26853001	chr17	26,853,001	26,854,000	5	5	5	4	3	3	2	3	4	5	4	5	3	4	3	5	4	5	5	5	4
chr17:26854001	chr17	26,854,001	26,855,000	5	5	5	4	3	3	2	3	4	5	4	5	3	4	3	5	4	5	5	5	4
chr17:26855001	chr17	26,855,001	26,856,000	5	5	5	4	3	3	2	3	4	5	4	5	3	4	3	5	4	5	5	5	4
chr17:26856001	chr17	26,856,001	26,857,000	5	5	5	4	3	3	2	3	4	5	4	5	3	4	3	5	4	5	5	5	4
chr17:26857001	chr17	26,857,001	26,858,000	5	5	5	4	3	3	2	3	4	5	4	5	3	4	3	5	4	5	5	5	4
chr17:26862001	chr17	26,862,001	26,863,000	5	5	5	4	3	4	2	3	4	5	4	5	3	4	4	5	4	5	5	4	4
chr17:26863001	chr17	26,863,001	26,864,000	5	5	5	4	3	4	2	3	4	5	4	5	3	4	4	5	4	5	5	4	4
chr17:26864001	chr17	26,864,001	26,865,000	5	5	5	4	3	4	2	3	4	5	4	5	3	4	4	5	4	5	5	4	4
chr17:26865001	chr17	26,865,001	26,866,000	5	5	5	4	3	4	2	3	4	5	4	5	3	4	4	5	4	5	5	4	4
chr17:26866001	chr17	26,866,001	26,867,000	5	5	5	4	3	4	2	3	4	5	4	5	3	4	4	5	4	5	5	4	4
chr17:26867001	chr17	26,867,001	26,868,000	5	5	5	4	3	4	2	3	4	5	4	5	3	4	4	5	4	5	5	4	4
chr9:41237001	chr9	41,237,001	41,238,000	5	5	5	4	4	4	4	4	4	4	4	4	4	4	4	5	4	5	4	4	5
chr9:41239001	chr9	41,239,001	41,240,000	5	5	5	4	4	4	4	4	4	4	4	4	4	4	4	5	4	5	4	4	5
chr9:41240001	chr9	41,240,001	41,241,000	5	5	5	4	4	4	4	4	4	4	4	4	4	4	4	5	4	5	4	4	5
chr9:41241001	chr9	41,241,001	41,242,000	5	5	5	4	4	4	4	4	4	4	4	4	4	4	4	5	4	5	4	4	5
chr9:41242001	chr9	41,242,001	41,243,000	5	5	5	4	4	4	4	4	4	4	4	4	4	4	4	5	4	5	4	4	5
chr9:41243001	chr9	41,243,001	41,244,000	5	5	5	4	4	4	4	4	4	4	4	4	4	4	4	5	4	5	4	4	5
chr9:41244001	chr9	41,244,001	41,245,000	5	5	5	4	4	4	4	4	4	4	4	4	4	4	4	5	4	5	4	4	5
chr9:41245001	chr9	41,245,001	41,246,000	5	5	5	4	4	4	4	4	4	4	4	4	4	4	4	5	4	5	4	4	5
chr9:41246001	chr9	41,246,001	41,247,000	5	5	5	4	4	4	4	4	4	4	4	4	4	4	4	5	4	5	4	4	5
chr9:41247001	chr9	41,247,001	41,248,000	5	5	5	4	4	4	4	4	4	4	4	4	4	4	4	5	4	5	4	4	5
chr9:41248001	chr9	41,248,001	41,249,000	5	5	5	4	4	4	4	4	4	4	4	4	4	4	4	5	4	5	4	4	5
chr9:41249001	chr9	41,249,001	41,250,000	5	5	5	4	4	4	4	3	4	4	4	4	4	4	4	5	4	5	4	4	5
chr9:41250001	chr9	41,250,001	41,251,000	5	5	5	4	4	4	4	3	4	4	4	4	4	4	4	5	4	5	4	4	5
chr9:41251001	chr9	41,251,001	41,252,000	5	5	5	4	4	4	4	3	4	4	4	4	4	4	4	5	4	5	4	4	5
chr9:63782001	chr9	63,782,001	63,783,000	5	5	5	4	4	3	3	3	4	4	4	5	4	4	3	5	4	5	4	4	5
chr9:63793001	chr9	63,793,001	63,794,000	5	5	5	4	4	4	3	4	4	4	4	4	4	4	4	5	4	5	4	4	5
chr9:63794001	chr9	63,794,001	63,795,000	5	5	5	4	4	4	3	4	4	4	4	4	4	4	4	5	4	5	4	4	5
chr9:63795001	chr9	63,795,001	63,796,000	5	5	5	4	4	4	3	4	4	4	4	4	4	4	4	5	4	5	4	4	5
chr9:63796001	chr9	63,796,001	63,797,000	5	5	5	4	4	4	3	4	4	4	4	4	4	4	4	5	4	5	4	4	5
chr9:63797001	chr9	63,797,001	63,798,000	5	5	5	4	4	4	3	4	4	4	4	4	4	4	4	5	4	5	4	4	5
chr9:63798001	chr9	63,798,001	63,799,000	5	5	5	4	4	4	3	4	4	4	4	4	4	4	4	5	4	5	4	4	5
chr9:63799001	chr9	63,799,001	63,800,000	5	5	5	4	4	4	3	4	4	4	4	4	4	4	4	5	4	5	4	4	5
chr9:63802001	chr9	63,802,001	63,803,000	5	5	5	4	4	4	3	4	4	4	4	4	4	4	4	5	4	5	4	4	5
chr9:63803001	chr9	63,803,001	63,804,000	5	5	5	4	4	4	3	4	4	4	4	4	4	4	4	5	4	5	4	4	5
chr9:63804001	chr9	63,804,001	63,805,000	5	5	5	4	4	4	3	4	4	4	4	4	4	4	4	5	4	5	4	4	5
chr9:63805001	chr9	63,805,001	63,806,000	5	5	5	4	4	4	3	4	4	4	4	4	4	4	4	5	4	5	4	4	5
chr9:63806001	chr9	63,806,001	63,807,000	5	5	5	4	4	4	3	4	4	4	4	4	4	4	4	5	4	5	4	4	5
chr9:63807001	chr9	63,807,001	63,808,000	5	5	5	4	4	4	3	4	4	4	4	4	4	4	4	5	4	5	4	4	5
chr9:63808001	chr9	63,808,001	63,809,000	5	5	5	4	4	4	3	4	4	4	4	4	4	4	4	5	4	5	4	4	5
chr9:63809001	chr9	63,809,001	63,810,000	5	5	5	4	4	4	3	4	4	4	4	4	4	4	4	5	4	5	4	4	5
chr9:63810001	chr9	63,810,001	63,811,000	5	5	5	4	4	4	3	4	4	4	4	4	4	4	4	5	4	5	4	4	5
chr10:38926001	chr10	38,926,001	38,927,000	5	5	5	4	4	3	3	3	4	5	4	4	4	4	3	4	4	5	5	4	5
chr10:38927001	chr10	38,927,001	38,928,000	5	5	5	4	4	3	3	3	4	5	4	4	4	4	3	4	4	5	5	4	5
chr15:17021001	chr15	17,021,001	17,022,000	5	5	5	4	4	3	3	3	4	5	5	4	4	3	4	5	4	5	4	4	4
chr17:21851001	chr17	21,851,001	21,852,000	5	5	5	4	4	3	2	3	3	5	4	5	3	3	3	5	4	5	5	5	4
chr17:21852001	chr17	21,852,001	21,853,000	5	5	5	4	4	3	2	3	3	5	4	5	3	3	3	5	4	5	5	5	4
chr17:26782001	chr17	26,782,001	26,783,000	5	5	5	4	3	3	2	3	3	5	4	5	3	4	3	5	3	5	5	5	4
chr17:26791001	chr17	26,791,001	26,792,000	5	5	5	4	3	3	2	3	3	5	4	5	3	3	3	5	4	5	5	5	4
chr17:26792001	chr17	26,792,001	26,793,000	5	5	5	4	3	3	2	3	3	5	4	5	3	3	3	5	4	5	5	5	4
chr17:26793001	chr17	26,793,001	26,794,000	5	5	5	4	3	3	2	3	3	5	4	5	3	3	3	5	4	5	5	5	4
chr17:26794001	chr17	26,794,001	26,795,000	5	5	5	4	3	3	2	3	3	5	4	5	3	3	3	5	4	5	5	5	4
chr17:26795001	chr17	26,795,001	26,796,000	5	5	5	4	3	3	2	3	3	5	4	5	3	3	3	5	4	5	5	5	4
chr17:26796001	chr17	26,796,001	26,797,000	5	5	5	4	3	3	2	3	3	5	4	5	3	3	3	5	4	5	5	5	4
chr17:26797001	chr17	26,797,001	26,798,000	5	5	5	4	3	3	2	3	3	5	4	5	3	3	3	5	4	5	5	5	4
chr17:26833001	chr17	26,833,001	26,834,000	5	5	5	4	3	3	2	3	3	5	4	5	3	4	3	5	4	4	5	5	4
chr15:101753001	chr15	101,753,001	101,754,000	3	2	1	4	5	4	4	5	5	4	5	5	4	4	5	5	5	4	2	3	5
chr15:101754001	chr15	101,754,001	101,755,000	3	2	1	4	5	4	4	5	5	4	5	5	4	4	5	5	5	4	2	3	5
chr4:49161001	chr4	49,161,001	49,162,000	5	5	5	4	4	3	2	3	3	4	4	4	3	5	3	5	3	5	5	4	5
chr4:49246001	chr4	49,246,001	49,247,000	5	5	5	4	3	3	1	3	3	4	5	3	3	5	3	5	3	5	5	5	4
chr4:49247001	chr4	49,247,001	49,248,000	5	5	5	4	3	3	1	3	3	4	5	3	4	5	2	5	3	5	5	5	4
chr4:49541001	chr4	49,541,001	49,542,000	5	5	5	4	3	3	2	3	4	4	5	3	4	4	3	5	4	5	5	4	4
chr4:49542001	chr4	49,542,001	49,543,000	5	5	5	4	3	3	2	3	4	4	5	3	4	4	3	5	4	5	5	4	4
chr4:49543001	chr4	49,543,001	49,544,000	5	5	5	4	3	3	2	3	4	4	5	3	4	4	3	5	4	5	5	4	4
chr4:49544001	chr4	49,544,001	49,545,000	5	5	5	4	3	3	2	3	4	4	5	3	4	4	3	5	4	5	5	4	4
chr7:62401001	chr7	62,401,001	62,402,000	5	5	5	4	4	3	3	4	4	5	5	4	4	4	3	5	4	4	4	4	4
chr9:63780001	chr9	63,780,001	63,781,000	5	5	5	4	4	3	3	3	4	4	4	5	4	4	3	5	4	5	4	4	4
chr9:63781001	chr9	63,781,001	63,782,000	5	5	5	4	4	3	3	3	4	4	4	5	4	4	3	5	4	5	4	4	4
chr9:63800001	chr9	63,800,001	63,801,000	5	5	5	4	4	4	3	4	4	4	4	4	4	4	4	5	4	5	4	4	4
chr9:63801001	chr9	63,801,001	63,802,000	5	5	5	4	4	4	3	4	4	4	4	4	4	4	4	5	4	5	4	4	4
chr17:21968001	chr17	21,968,001	21,969,000	5	5	5	4	4	3	2	3	3	5	5	5	4	4	3	5	3	5	4	4	3
chr17:26798001	chr17	26,798,001	26,799,000	5	5	5	4	3	3	2	3	3	5	4	5	3	3	3	5	3	5	5	5	4
chr1:16866001	chr1	16,866,001	16,867,000	4	3	2	4	5	4	4	4	4	5	4	4	4	4	4	5	4	4	4	4	4
chr1:16867001	chr1	16,867,001	16,868,000	4	3	2	5	5	4	4	4	5	5	4	4	4	4	4	5	4	4	4	4	4
chr15:101755001	chr15	101,755,001	101,756,000	3	2	1	4	5	4	4	5	5	4	5	5	4	4	5	5	5	4	2	2	5
chr15:101756001	chr15	101,756,001	101,757,000	3	2	1	4	4	4	4	5	5	4	5	5	4	4	5	5	5	4	2	2	5
chr15:101757001	chr15	101,757,001	101,758,000	3	2	1	4	4	4	4	5	5	4	5	5	4	4	5	5	5	4	2	2	5
chr4:49538001	chr4	49,538,001	49,539,000	5	5	5	4	3	3	2	3	4	4	5	3	4	4	3	5	4	5	4	4	4
chr4:49539001	chr4	49,539,001	49,540,000	5	5	5	4	3	3	2	3	4	4	5	3	4	4	3	5	4	5	4	4	4
chr4:49540001	chr4	49,540,001	49,541,000	5	5	5	4	3	3	2	3	4	4	5	3	4	4	3	5	4	5	4	4	4
chr4:49545001	chr4	49,545,001	49,546,000	5	5	5	4	3	3	2	3	4	4	5	3	3	4	3	5	4	5	5	4	4
chr4:49546001	chr4	49,546,001	49,547,000	5	5	5	4	3	3	2	3	4	4	5	3	3	4	3	5	4	5	5	4	4
chr9:41280001	chr9	41,280,001	41,281,000	5	5	5	4	4	4	3	3	4	4	4	4	4	4	3	5	5	4	4	4	4
chr9:41282001	chr9	41,282,001	41,283,000	5	5	5	4	4	4	3	3	4	4	4	4	4	4	3	5	5	4	4	4	4
chr9:41283001	chr9	41,283,001	41,284,000	5	5	5	4	4	4	3	3	4	4	4	4	4	4	3	5	5	4	4	4	4
chr9:41284001	chr9	41,284,001	41,285,000	5	5	5	4	4	4	3	3	4	4	4	4	4	4	3	5	5	4	4	4	4
chr9:41285001	chr9	41,285,001	41,286,000	5	5	5	4	4	4	3	3	4	4	4	4	4	4	3	5	5	4	4	4	4
chr9:41286001	chr9	41,286,001	41,287,000	5	5	5	4	4	4	3	3	4	4	4	4	4	4	3	5	5	4	4	4	4
chr9:41287001	chr9	41,287,001	41,288,000	5	5	5	4	4	4	3	3	4	4	4	4	4	4	3	5	5	4	4	4	4
chr9:41288001	chr9	41,288,001	41,289,000	5	5	5	4	4	4	3	3	4	4	4	4	4	4	3	5	5	4	4	4	4
chr9:63779001	chr9	63,779,001	63,780,000	5	5	5	4	4	3	3	3	4	4	4	5	4	4	3	5	4	4	4	4	4
chr10:38932001	chr10	38,932,001	38,933,000	5	5	5	4	4	3	3	3	4	5	4	4	4	3	3	4	4	5	5	4	4
chr10:38933001	chr10	38,933,001	38,934,000	5	5	5	4	4	3	3	3	4	5	4	4	4	3	3	4	4	5	5	4	4
chr10:38934001	chr10	38,934,001	38,935,000	5	5	5	4	4	3	3	3	4	5	4	4	4	3	3	4	4	5	5	4	4
chr10:38935001	chr10	38,935,001	38,936,000	5	5	5	4	4	3	3	3	4	5	4	4	4	3	3	4	4	5	5	4	4
chr16:34065001	chr16	34,065,001	34,066,000	5	5	5	3	3	3	2	3	3	5	4	5	3	4	3	5	2	5	5	4	4
chr16:34066001	chr16	34,066,001	34,067,000	5	5	5	4	3	3	2	3	3	5	4	5	3	4	3	5	2	5	5	4	4
chr16:34067001	chr16	34,067,001	34,068,000	5	5	5	4	3	3	2	3	3	5	4	5	3	4	3	5	2	5	5	4	4
chr17:26799001	chr17	26,799,001	26,800,000	5	5	5	4	3	3	2	3	3	5	4	5	3	3	3	5	3	5	5	4	4
chr17:26800001	chr17	26,800,001	26,801,000	5	5	5	4	3	3	2	3	3	5	4	5	3	3	3	5	3	5	5	4	4
chr17:26801001	chr17	26,801,001	26,802,000	5	5	5	4	3	3	2	3	3	5	4	5	3	3	3	5	3	5	5	4	4
chr17:26802001	chr17	26,802,001	26,803,000	5	5	5	4	3	3	2	3	3	5	4	5	3	3	3	5	3	5	5	4	4
chr17:26803001	chr17	26,803,001	26,804,000	5	5	5	4	3	3	2	3	3	5	4	5	3	3	3	5	3	5	5	4	4
chr17:26804001	chr17	26,804,001	26,805,000	5	5	5	4	3	3	2	3	3	5	4	5	3	3	3	5	3	5	5	4	4
chr17:26806001	chr17	26,806,001	26,807,000	5	5	5	4	3	3	2	3	3	5	4	5	3	3	3	5	3	5	5	4	4
chr17:26829001	chr17	26,829,001	26,830,000	5	5	5	4	3	3	2	3	3	5	4	5	3	3	3	5	4	4	5	4	4

Region	CHR	start	end	HD2	HD3	HD1	BN1	BN2	BN3	BN4	BN5	BN6	LC1	LC2	LC3	LC4	LC5	LC6	LC7	LC8	LC9	LC10	LC10*	LC11
chr17:26830001	chr17	26,830,001	26,831,000	5	5	5	4	3	3	2	3	3	5	4	5	3	3	3	5	4	4	5	4	4
chr17:26831001	chr17	26,831,001	26,832,000	5	5	5	4	3	3	2	3	3	5	4	5	3	3	3	5	4	4	5	4	4
chr17:26832001	chr17	26,832,001	26,833,000	5	5	5	4	3	3	2	3	3	5	4	5	3	3	3	5	4	4	5	4	4
chr17:26936001	chr17	26,936,001	26,937,000	5	5	5	3	2	2	1	2	3	5	5	5	3	3	2	5	2	5	5	5	4
chr17:26937001	chr17	26,937,001	26,938,000	5	5	5	3	2	2	1	2	3	5	5	5	3	3	2	5	2	5	5	5	4
chr17:26938001	chr17	26,938,001	26,939,000	5	5	5	3	2	2	1	2	3	5	5	5	3	3	2	5	2	5	5	5	4
chr17:26939001	chr17	26,939,001	26,940,000	5	5	5	3	2	2	1	2	3	5	5	5	3	3	2	5	2	5	5	5	4
chr17:26940001	chr17	26,940,001	26,941,000	5	5	5	3	2	2	1	2	3	5	5	5	3	3	2	5	2	5	5	5	4
chr4:49245001	chr4	49,245,001	49,246,000	5	4	4	3	3	3	2	3	3	4	4	3	3	5	3	5	3	5	5	5	4
chr1:262001	chr1	262,001	263,000	3	3	3	5	4	5	5	4	4	4	4	4	5	4	5	4	4	4	3	4	4
chr1:263001	chr1	263,001	264,000	3	3	3	5	4	5	5	4	4	4	4	4	5	4	5	4	4	4	3	4	4
chr2:94286001	chr2	94,286,001	94,287,000	5	5	5	4	4	3	3	3	4	4	4	4	4	4	3	4	4	4	5	4	4
chr2:94287001	chr2	94,287,001	94,288,000	5	5	5	4	4	3	3	3	4	4	4	4	4	4	3	4	4	4	5	4	4
chr2:94288001	chr2	94,288,001	94,289,000	5	5	5	4	4	3	3	3	4	4	4	4	4	4	3	4	4	4	5	4	4
chr2:94289001	chr2	94,289,001	94,290,000	5	5	5	4	4	4	3	3	4	4	4	4	4	4	3	4	4	4	5	4	4
chr2:94290001	chr2	94,290,001	94,291,000	5	5	5	4	4	4	4	3	4	4	4	4	4	4	3	4	4	4	5	4	4
chr2:94291001	chr2	94,291,001	94,292,000	5	5	5	4	4	4	4	3	4	4	4	4	4	4	3	4	4	4	5	4	4
chr2:94292001	chr2	94,292,001	94,293,000	5	5	5	4	4	4	4	3	4	4	4	4	4	4	3	4	4	4	5	4	4
chr3:75794001	chr3	75,794,001	75,795,000	4	5	5	4	4	3	3	3	4	4	5	4	4	4	3	4	4	4	4	4	4
chr4:49162001	chr4	49,162,001	49,163,000	5	5	5	4	4	3	2	3	3	4	4	4	3	4	3	5	3	5	5	4	4
chr4:49163001	chr4	49,163,001	49,164,000	5	5	5	4	4	3	2	3	3	4	4	4	3	4	3	5	3	5	5	4	4
chr4:49164001	chr4	49,164,001	49,165,000	5	5	5	4	4	3	2	3	3	4	4	4	3	4	3	5	3	5	5	4	4
chr4:190065001	chr4	190,065,001	190,066,000	5	5	5	3	3	3	2	2	3	5	5	4	4	5	3	5	3	5	3	3	3
chr4:190066001	chr4	190,066,001	190,067,000	5	5	5	3	3	2	1	2	3	5	5	4	4	5	2	5	4	5	3	3	3
chr9:63770001	chr9	63,770,001	63,771,000	5	5	5	4	4	3	3	4	4	4	4	4	4	4	3	5	4	4	4	4	4
chr9:63771001	chr9	63,771,001	63,772,000	5	5	5	4	4	3	3	4	4	4	4	4	4	4	3	5	4	4	4	4	4
chr9:63772001	chr9	63,772,001	63,773,000	5	5	5	4	4	3	3	4	4	4	4	4	4	4	3	5	4	4	4	4	4
chr9:63773001	chr9	63,773,001	63,774,000	5	5	5	4	4	3	3	4	4	4	4	4	4	4	3	5	4	4	4	4	4
chr9:63774001	chr9	63,774,001	63,775,000	5	5	5	4	4	3	3	4	4	4	4	4	4	4	3	5	4	4	4	4	4
chr9:63775001	chr9	63,775,001	63,776,000	5	5	5	4	4	3	3	4	4	4	4	4	4	4	3	5	4	4	4	4	4
chr9:63776001	chr9	63,776,001	63,777,000	5	5	5	4	4	3	3	4	4	4	4	4	4	4	3	5	4	4	4	4	4
chr9:63777001	chr9	63,777,001	63,778,000	5	5	5	4	4	3	3	4	4	4	4	4	4	4	3	5	4	4	4	4	4
chr9:63778001	chr9	63,778,001	63,779,000	5	5	5	4	4	3	3	4	4	4	4	4	4	4	3	5	4	4	4	4	4
chr10:38936001	chr10	38,936,001	38,937,000	5	5	5	4	4	3	3	3	4	5	4	4	4	3	3	4	4	4	5	4	4
chr10:38937001	chr10	38,937,001	38,938,000	5	5	5	4	4	3	3	3	4	5	4	4	4	3	3	4	4	4	5	4	4
chr15:17027001	chr15	17,027,001	17,028,000	5	5	5	4	4	3	3	4	4	4	4	4	4	3	4	5	3	5	4	4	4
chr15:17028001	chr15	17,028,001	17,029,000	5	5	5	4	4	3	3	4	4	4	4	4	4	3	4	5	3	5	4	4	4
chr16:34062001	chr16	34,062,001	34,063,000	5	5	5	3	2	2	1	2	3	5	4	5	3	4	2	5	2	5	5	4	4
chr16:34063001	chr16	34,063,001	34,064,000	5	5	5	3	3	2	1	2	3	5	4	5	3	4	2	5	2	5	5	4	4
chr16:34064001	chr16	34,064,001	34,065,000	5	5	5	3	3	2	1	2	3	5	4	5	3	4	2	5	2	5	5	4	4
chr17:26805001	chr17	26,805,001	26,806,000	5	5	5	4	3	3	2	3	3	5	4	5	3	3	3	5	3	4	5	4	4
chr17:26807001	chr17	26,807,001	26,808,000	5	5	5	4	3	3	2	3	3	5	4	5	3	3	3	5	3	4	5	4	4
chr17:26808001	chr17	26,808,001	26,809,000	5	5	5	4	3	3	2	3	3	5	4	5	3	3	3	5	3	4	5	4	4
chr17:26809001	chr17	26,809,001	26,810,000	5	5	5	4	3	3	2	3	3	5	4	5	3	3	3	5	3	4	5	4	4
chr17:26810001	chr17	26,810,001	26,811,000	5	5	5	4	3	3	2	3	3	5	4	5	3	3	3	5	3	4	5	4	4
chr17:26811001	chr17	26,811,001	26,812,000	5	5	5	4	3	3	2	3	3	5	4	5	3	3	3	5	3	4	5	4	4
chr17:26812001	chr17	26,812,001	26,813,000	5	5	5	4	3	3	2	3	3	5	4	5	3	3	3	5	3	4	5	4	4
chr17:26813001	chr17	26,813,001	26,814,000	5	5	5	4	3	3	2	3	3	5	4	5	3	3	3	5	3	4	5	4	4
chr17:26814001	chr17	26,814,001	26,815,000	5	5	5	4	3	3	2	3	3	5	4	5	3	3	3	5	3	4	5	4	4
chr17:26815001	chr17	26,815,001	26,816,000	5	5	5	4	3	3	2	3	3	5	4	5	3	3	3	5	3	4	5	4	4
chr17:26816001	chr17	26,816,001	26,817,000	5	5	5	4	3	3	2	3	3	5	4	5	3	3	3	5	3	4	5	4	4
chr17:26817001	chr17	26,817,001	26,818,000	5	5	5	4	3	3	2	3	3	5	4	5	3	3	3	5	3	4	5	4	4
chr17:26818001	chr17	26,818,001	26,819,000	5	5	5	4	3	3	2	3	3	5	4	5	3	3	3	5	3	4	5	4	4
chr17:26819001	chr17	26,819,001	26,820,000	5	5	5	4	3	3	2	3	3	5	4	5	3	3	3	5	3	4	5	4	4
chr17:26820001	chr17	26,820,001	26,821,000	5	5	5	4	3	3	2	3	3	5	4	5	3	3	3	5	3	4	5	4	4
chr4:49165001	chr4	49,165,001	49,166,000	5	5	5	4	4	3	2	3	3	4	4	4	3	4	3	5	3	4	5	4	4
chr9:63762001	chr9	63,762,001	63,763,000	5	5	5	4	4	3	3	3	4	4	4	4	4	4	3	4	4	4	4	4	4
chr9:63763001	chr9	63,763,001	63,764,000	5	5	5	4	4	3	3	3	4	4	4	4	4	4	3	4	4	4	4	4	4
chr9:63764001	chr9	63,764,001	63,765,000	5	5	5	4	4	3	3	3	4	4	4	4	4	4	3	4	4	4	4	4	4
chr9:63765001	chr9	63,765,001	63,766,000	5	5	5	4	4	3	3	3	4	4	4	4	4	4	3	4	4	4	4	4	4
chr9:63766001	chr9	63,766,001	63,767,000	5	5	5	4	4	3	3	3	4	4	4	4	4	4	3	4	4	4	4	4	4
chr9:63767001	chr9	63,767,001	63,768,000	5	5	5	4	4	3	3	3	4	4	4	4	4	4	3	4	4	4	4	4	4
chr9:63768001	chr9	63,768,001	63,769,000	5	5	5	4	4	3	3	4	4	4	4	4	4	4	3	4	4	4	4	4	4
chr9:63769001	chr9	63,769,001	63,770,000	5	5	5	4	4	3	3	4	4	4	4	4	4	4	3	4	4	4	4	4	4
chr10:38938001	chr10	38,938,001	38,939,000	5	5	5	4	4	3	3	3	4	5	4	4	4	3	3	4	4	4	4	4	4
chr10:38939001	chr10	38,939,001	38,940,000	5	5	5	4	4	3	3	3	4	5	4	4	4	3	3	4	4	4	4	4	4
chr10:38940001	chr10	38,940,001	38,941,000	5	5	5	4	3	3	3	3	4	5	4	4	4	3	3	4	4	4	4	4	4
chr10:38941001	chr10	38,941,001	38,942,000	5	5	5	4	3	3	3	3	4	5	4	4	4	3	3	4	4	4	4	4	4
chr15:17020001	chr15	17,020,001	17,021,000	5	5	5	3	4	3	3	3	4	5	5	4	4	3	4	5	4	3	4	3	3
chr17:26821001	chr17	26,821,001	26,822,000	5	5	5	4	3	3	2	3	3	5	4	5	3	3	3	5	3	4	4	4	4
chr17:26822001	chr17	26,822,001	26,823,000	5	5	5	4	3	3	2	3	3	5	4	5	3	3	3	5	3	4	4	4	4
chr17:26823001	chr17	26,823,001	26,824,000	5	5	5	4	3	3	2	3	3	5	4	5	3	3	3	5	3	4	4	4	4
chr17:26824001	chr17	26,824,001	26,825,000	5	5	5	4	3	3	2	3	3	5	4	5	3	3	3	5	3	4	4	4	4
chr17:26825001	chr17	26,825,001	26,826,000	5	5	5	4	3	3	2	3	3	5	4	5	3	3	3	5	3	4	4	4	4
chr17:26826001	chr17	26,826,001	26,827,000	5	5	5	4	3	3	2	3	3	5	4	5	3	3	3	5	3	4	4	4	4
chr17:26827001	chr17	26,827,001	26,828,000	5	5	5	4	3	3	2	3	3	5	4	5	3	3	3	5	3	4	4	4	4
chr17:26828001	chr17	26,828,001	26,829,000	5	5	5	4	3	3	2	3	3	5	4	5	3	3	3	5	3	4	4	4	4
chr2:94266001	chr2	94,266,001	94,267,000	5	5	5	4	4	3	3	3	4	4	4	4	4	4	3	4	3	4	4	4	4
chr2:94267001	chr2	94,267,001	94,268,000	5	5	5	4	4	3	3	3	4	4	4	4	4	4	3	4	3	4	4	4	4
chr2:94268001	chr2	94,268,001	94,269,000	5	5	5	4	4	3	3	3	4	4	4	4	4	4	3	4	3	4	4	4	4
chr2:94269001	chr2	94,269,001	94,270,000	5	5	5	4	4	3	3	3	4	4	4	4	4	4	3	4	3	4	4	4	4
chr2:94270001	chr2	94,270,001	94,271,000	5	5	5	4	4	3	3	3	4	4	4	4	4	4	3	4	3	4	4	4	4
chr2:94271001	chr2	94,271,001	94,272,000	5	5	5	4	4	3	3	3	4	4	4	4	4	4	3	4	3	4	4	4	4
chr2:94272001	chr2	94,272,001	94,273,000	5	5	5	4	4	3	3	3	4	4	4	4	4	4	3	4	3	4	4	4	4
chr2:94273001	chr2	94,273,001	94,274,000	5	5	5	4	4	3	3	3	4	4	4	4	4	4	3	4	3	4	4	4	4
chr2:94274001	chr2	94,274,001	94,275,000	5	5	5	4	4	3	3	3	4	4	4	4	4	4	3	4	3	4	4	4	4
chr2:94275001	chr2	94,275,001	94,276,000	5	5	5	4	4	3	3	3	4	4	4	4	4	4	3	4	3	4	4	4	4
chr2:94276001	chr2	94,276,001	94,277,000	5	5	5	4	4	3	3	3	4	4	4	4	4	4	3	4	3	4	4	4	4
chr2:94277001	chr2	94,277,001	94,278,000	5	5	5	4	4	3	3	3	4	4	4	4	4	4	3	4	3	4	4	4	4
chr2:94278001	chr2	94,278,001	94,279,000	5	5	5	4	4	3	3	3	4	4	4	4	4	4	3	4	3	4	4	4	4
chr2:94279001	chr2	94,279,001	94,280,000	5	5	5	4	4	3	3	3	4	4	4	4	4	4	3	4	3	4	4	4	4
chr2:94280001	chr2	94,280,001	94,281,000	5	5	5	4	4	3	3	3	4	4	4	4	4	4	3	4	3	4	4	4	4
chr2:94281001	chr2	94,281,001	94,282,000	5	5	5	4	4	3	3	3	4	4	4	4	4	4	3	4	3	4	4	4	4
chr2:94282001	chr2	94,282,001	94,283,000	5	5	5	4	4	3	3	3	4	4	4	4	4	4	3	4	3	4	4	4	4
chr2:94283001	chr2	94,283,001	94,284,000	5	5	5	4	4	3	3	3	4	4	4	4	4	4	3	4	3	4	4	4	4
chr2:94284001	chr2	94,284,001	94,285,000	5	5	5	4	4	3	3	3	4	4	4	4	4	4	3	4	3	4	4	4	4
chr2:94285001	chr2	94,285,001	94,286,000	5	5	5	4	4	3	3	3	4	4	4	4	4	4	3	4	3	4	4	4	4
chr7:62397001	chr7	62,397,001	62,398,000	5	4	5	4	3	3	3	4	4	4	4	4	4	4	3	4	3	4	4	4	4
chr9:63759001	chr9	63,759,001	63,760,000	5	5	5	4	4	3	3	3	4	4	4	4	4	3	3	4	4	4	4	4	4
chr9:63760001	chr9	63,760,001	63,761,000	5	5	5	4	4	3	3	3	4	4	4	4	4	3	3	4	4	4	4	4	4
chr9:63761001	chr9	63,761,001	63,762,000	5	5	5	4	4	3	3	3	4	4	4	4	4	3	3	4	4	4	4	4	4
chr10:38942001	chr10	38,942,001	38,943,000	5	5	5	4	3	3	3	3	4	5	4	4	4	3	3	4	3	4	4	4	4
chr10:38943001	chr10	38,943,001	38,944,000	5	5	5	4	3	3	3	3	4	5	4	4	4	3	3	4	3	4	4	4	4
chr13:63066001	chr13	63,066,001	63,067,000	5	5	5	4	3	3	2	2	4	4	4	4	3	3	3	5	4	5	4	3	4
chr13:63067001	chr13	63,067,001	63,068,000	5	5	5	4	3	3	2	2	4	4	4	4	3	3	3	5	4	5	4	3	4
chr13:63068001	chr13	63,068,001	63,069,000	5	5	5	4	3	3	2	2	4	4	4	4	3	3	3	5	4	5	4	3	4
chr13:63069001	chr13	63,069,001	63,070,000	5	5	5	4	3	3	2	2	4	4	4	4	3	3	3	5	4	5	4	3	4
chr13:63070001	chr13	63,070,001	63,071,000	5	5	5	4	3	3	2	2	4	4	4	4	3	3	3	5	4	5	4	3	4
chr13:63071001	chr13	63,071,001	63,072,000	5	5	5	4	3	3	2	2	4	4	4	4	3	3	3	5	4	5	4	3	4
chr13:63072001	chr13	63,072,001	63,073,000	5	5	5	4	3	3	2	2	4	4	4	4	3	3	3	5	4	5	4	3	4
chr13:63073001	chr13	63,073,001	63,074,000	5	5	5	4	3	3	2	2	4	4	4	4	3	3	3	5	4	5	4	3	4
chr13:63074001	chr13	63,074,001	63,075,000	5	5	5	4	3	2	2	2	4	4	4	4	3	3	3	5	4	5	4	3	4
chr15:17019001	chr15	17,019,001	17,020,000	5	5	5	3	4	3	3	3	4	4	5	4	4	3	4	5	4	3	4	3	3

Region	CHR	start	end	HD2	HD3	HD1	BN1	BN2	BN3	BN4	BN5	BN6	LC1	LC2	LC3	LC4	LC5	LC6	LC7	LC8	LC9	LC10	LC10*	LC11
chr15:17058001	chr15	17,058,001	17,059,000	5	5	5	3	3	4	3	4	3	4	4	4	3	3	4	5	3	4	4	4	4
chr15:17059001	chr15	17,059,001	17,060,000	5	5	5	3	3	4	3	4	3	4	4	4	3	3	4	5	3	4	4	4	4
chr15:17060001	chr15	17,060,001	17,061,000	5	5	5	3	3	4	3	4	3	4	4	4	3	3	4	5	3	4	4	4	4
chr15:17061001	chr15	17,061,001	17,062,000	5	5	5	3	3	4	3	4	3	4	4	4	3	3	4	5	3	4	4	4	4
chr16:34097001	chr16	34,097,001	34,098,000	5	5	5	3	3	2	2	2	3	5	4	5	3	3	2	5	2	4	5	4	4
chr16:34098001	chr16	34,098,001	34,099,000	5	5	5	3	3	2	2	2	3	5	4	5	3	3	2	5	2	4	5	4	4
chr16:34099001	chr16	34,099,001	34,100,000	5	5	5	3	3	2	2	2	3	5	4	5	3	3	2	5	2	4	5	4	4
chr17:26781001	chr17	26,781,001	26,782,000	5	5	5	3	3	3	2	2	3	5	4	5	3	3	3	5	3	5	3	3	4
chr2:87419001	chr2	87,419,001	87,420,000	5	5	5	3	3	4	2	3	3	5	4	3	4	3	3	4	5	4	3	3	4
chr2:94263001	chr2	94,263,001	94,264,000	5	5	5	4	4	3	3	3	4	4	4	4	3	4	3	4	3	4	4	4	4
chr2:94264001	chr2	94,264,001	94,265,000	5	5	5	4	4	3	3	3	4	4	4	4	3	4	3	4	3	4	4	4	4
chr2:94265001	chr2	94,265,001	94,266,000	5	5	5	4	4	3	3	3	4	4	4	4	3	4	3	4	3	4	4	4	4
chr2:95860001	chr2	95,860,001	95,861,000	5	5	5	4	3	2	2	3	4	4	4	4	3	3	3	4	3	4	5	4	4
chr2:95861001	chr2	95,861,001	95,862,000	5	5	5	4	3	2	2	3	4	4	4	4	3	3	3	4	3	4	5	4	4
chr3:90541001	chr3	90,541,001	90,542,000	5	5	5	4	3	3	2	3	3	4	4	4	4	4	2	4	3	4	5	3	4
chr3:90542001	chr3	90,542,001	90,543,000	5	5	5	4	3	3	2	3	3	4	4	4	4	4	2	4	3	4	5	3	4
chr4:49572001	chr4	49,572,001	49,573,000	5	5	5	4	2	2	1	2	3	4	4	2	3	4	2	5	3	5	5	4	4
chr4:49573001	chr4	49,573,001	49,574,000	5	5	5	4	2	2	1	2	3	4	4	2	3	4	2	5	3	5	5	4	4
chr4:49574001	chr4	49,574,001	49,575,000	5	5	5	4	2	2	1	2	3	4	4	2	3	4	2	5	3	5	5	4	4
chr7:62393001	chr7	62,393,001	62,394,000	5	4	5	4	3	3	3	3	3	4	4	4	3	4	3	4	3	4	4	4	4
chr9:42386001	chr9	42,386,001	42,387,000	4	4	5	3	3	3	2	3	3	4	5	4	3	4	2	4	4	4	4	4	3
chr9:42387001	chr9	42,387,001	42,388,000	4	4	5	3	3	3	2	3	3	4	5	4	3	4	2	4	4	4	4	4	3
chr9:42388001	chr9	42,388,001	42,389,000	4	4	5	2	3	3	2	3	3	4	5	4	3	4	2	4	4	4	4	4	3
chr9:43330001	chr9	43,330,001	43,331,000	5	5	5	4	4	4	4	4	4	4	4	4	3	4	3	5	3	4	4	3	4
chr9:43331001	chr9	43,331,001	43,332,000	5	5	5	4	4	4	4	4	4	4	4	4	3	4	3	5	3	4	4	3	4
chr9:63757001	chr9	63,757,001	63,758,000	5	5	5	4	4	3	3	3	4	4	4	4	4	3	3	4	4	4	4	4	3
chr9:63758001	chr9	63,758,001	63,759,000	5	5	5	4	4	3	3	3	4	4	4	4	4	3	3	4	4	4	4	4	3
chr10:38944001	chr10	38,944,001	38,945,000	5	5	5	4	3	3	3	3	4	4	4	4	4	3	3	4	3	4	4	4	4
chr13:63065001	chr13	63,065,001	63,066,000	5	5	5	4	3	3	2	2	4	4	4	4	3	3	3	5	4	4	4	3	4
chr15:17062001	chr15	17,062,001	17,063,000	5	5	5	3	3	4	3	4	3	4	3	4	3	3	4	5	3	4	4	4	4
chr16:34096001	chr16	34,096,001	34,097,000	5	5	5	3	3	2	2	2	3	5	4	5	3	3	2	5	2	4	5	4	3
chr2:87428001	chr2	87,428,001	87,429,000	4	5	4	3	3	2	2	3	3	5	5	3	3	3	3	4	3	3	5	4	4
chr2:87429001	chr2	87,429,001	87,430,000	4	5	4	3	3	2	2	3	3	5	5	3	3	3	3	4	3	3	5	4	4
chr2:87430001	chr2	87,430,001	87,431,000	4	5	4	3	3	2	2	3	3	5	5	3	3	3	3	4	3	3	5	4	4
chr4:49243001	chr4	49,243,001	49,244,000	5	4	4	3	3	3	2	3	3	4	4	3	3	5	3	4	3	4	4	4	4
chr4:49244001	chr4	49,244,001	49,245,000	5	4	4	3	3	3	2	3	3	4	4	3	3	5	3	4	3	4	4	4	4
chr9:67318001	chr9	67,318,001	67,319,000	5	4	4	3	4	3	2	3	3	4	3	4	4	4	3	4	4	3	4	4	4
chr19:105001	chr19	105,001	106,000	2	2	2	3	3	4	5	4	4	3	3	4	4	3	5	3	5	5	3	4	3
chr15:101760001	chr15	101,760,001	101,761,000	2	2	1	3	4	4	4	5	5	4	5	5	3	3	5	3	5	3	2	2	5
chr15:101761001	chr15	101,761,001	101,762,000	2	2	1	3	4	4	4	5	5	4	5	5	3	3	5	3	5	3	2	2	5
chr15:101762001	chr15	101,762,001	101,763,000	2	2	1	3	4	4	4	5	5	4	5	5	3	3	5	3	5	3	2	2	5
chr15:101763001	chr15	101,763,001	101,764,000	2	2	1	3	4	4	4	5	5	4	5	5	3	3	5	3	5	3	2	2	5
chr2:95858001	chr2	95,858,001	95,859,000	5	5	5	4	3	2	2	3	4	4	4	4	3	3	3	4	3	4	5	3	4
chr2:95859001	chr2	95,859,001	95,860,000	5	5	5	4	3	2	2	3	4	4	4	4	3	3	3	4	3	4	5	3	4
chr2:95928001	chr2	95,928,001	95,929,000	4	5	5	4	3	3	3	3	4	4	4	4	3	3	3	4	3	4	4	4	4
chr2:95929001	chr2	95,929,001	95,930,000	4	5	5	4	3	3	3	3	4	4	4	4	3	3	3	4	3	4	4	4	4
chr2:95930001	chr2	95,930,001	95,931,000	4	5	5	4	3	3	3	3	4	4	4	4	3	3	3	4	3	4	4	4	4
chr2:95931001	chr2	95,931,001	95,932,000	4	5	5	4	3	3	3	3	4	4	4	4	3	3	3	4	3	4	4	4	4
chr2:95932001	chr2	95,932,001	95,933,000	4	5	5	4	3	3	3	3	4	4	4	4	3	3	3	4	3	4	4	4	4
chr2:95933001	chr2	95,933,001	95,934,000	4	5	5	4	3	3	3	3	4	4	4	4	3	3	3	4	3	4	4	4	4
chr2:95934001	chr2	95,934,001	95,935,000	4	5	5	4	3	3	3	3	4	4	4	4	3	3	3	4	3	4	4	4	4
chr2:95935001	chr2	95,935,001	95,936,000	4	5	5	4	3	3	3	3	4	4	4	4	3	3	3	4	3	4	4	4	4
chr2:95936001	chr2	95,936,001	95,937,000	4	5	5	4	3	3	3	3	4	4	4	4	3	3	3	4	3	4	4	4	4
chr2:95937001	chr2	95,937,001	95,938,000	4	5	5	4	3	3	3	3	4	4	4	4	3	3	3	4	3	4	4	4	4
chr2:95938001	chr2	95,938,001	95,939,000	4	5	5	4	3	3	3	3	4	4	4	4	3	3	3	4	3	4	4	4	4
chr2:95939001	chr2	95,939,001	95,940,000	4	5	5	4	3	3	3	3	4	4	4	4	3	3	3	4	3	4	4	4	4
chr3:90510001	chr3	90,510,001	90,511,000	5	5	5	4	2	2	2	2	3	4	4	3	3	3	2	4	3	4	5	5	4
chr3:90540001	chr3	90,540,001	90,541,000	5	5	5	4	3	3	2	3	3	4	4	4	3	4	2	4	3	4	5	3	4
chr4:49193001	chr4	49,193,001	49,194,000	4	5	5	4	3	3	2	3	4	4	4	3	3	4	3	4	3	4	4	4	4
chr4:49194001	chr4	49,194,001	49,195,000	4	5	5	4	3	3	2	3	3	4	4	3	3	4	3	4	3	4	5	4	3
chr4:49195001	chr4	49,195,001	49,196,000	4	5	5	4	3	3	2	3	3	4	4	3	3	4	3	4	3	4	5	4	3
chr4:49196001	chr4	49,196,001	49,197,000	4	5	5	4	3	3	2	3	3	4	4	3	3	4	3	4	3	4	5	4	3
chr4:49575001	chr4	49,575,001	49,576,000	5	5	5	4	2	2	1	2	3	4	4	2	3	4	2	5	2	5	5	4	4
chr4:49576001	chr4	49,576,001	49,577,000	5	5	5	4	2	2	1	2	3	4	4	2	3	4	2	5	2	5	5	4	4
chr4:49577001	chr4	49,577,001	49,578,000	5	5	5	4	2	2	1	2	3	4	4	2	3	4	2	5	2	5	5	4	4
chr4:49578001	chr4	49,578,001	49,579,000	5	5	5	4	2	2	1	2	3	4	4	2	3	4	2	5	2	5	5	4	4
chr7:62387001	chr7	62,387,001	62,388,000	5	4	5	4	3	3	3	3	3	4	4	4	3	3	3	4	3	4	4	4	4
chr7:62388001	chr7	62,388,001	62,389,000	5	4	5	4	3	3	3	3	3	4	4	4	3	3	3	4	3	4	4	4	4
chr7:62389001	chr7	62,389,001	62,390,000	5	4	5	4	3	3	3	3	3	4	4	4	3	3	3	4	3	4	4	4	4
chr7:62390001	chr7	62,390,001	62,391,000	5	4	5	4	3	3	3	3	3	4	4	4	3	3	3	4	3	4	4	4	4
chr7:62391001	chr7	62,391,001	62,392,000	5	4	5	4	3	3	3	3	3	4	4	4	3	3	3	4	3	4	4	4	4
chr7:62392001	chr7	62,392,001	62,393,000	5	4	5	4	3	3	3	3	3	4	4	4	3	3	3	4	3	4	4	4	4
chr9:63755001	chr9	63,755,001	63,756,000	4	5	5	4	4	3	3	3	3	4	4	3	4	3	3	4	4	4	4	4	3
chr10:38945001	chr10	38,945,001	38,946,000	5	5	5	4	3	3	3	3	3	4	4	4	3	3	3	4	3	4	4	4	4
chr10:38946001	chr10	38,946,001	38,947,000	5	5	5	4	3	3	3	3	3	4	4	4	3	3	3	4	3	4	4	4	4
chr10:38947001	chr10	38,947,001	38,948,000	5	5	5	4	3	3	3	3	3	4	4	4	3	3	3	4	3	4	4	4	4
chr10:38948001	chr10	38,948,001	38,949,000	5	5	5	4	3	3	3	3	3	4	4	4	3	3	3	4	3	4	4	4	4
chr10:38949001	chr10	38,949,001	38,950,000	5	5	5	4	3	3	3	3	3	4	4	4	3	3	3	4	3	4	4	4	4
chr10:38950001	chr10	38,950,001	38,951,000	5	5	5	4	3	3	3	3	3	4	4	4	3	3	3	4	3	4	4	4	4
chr10:38951001	chr10	38,951,001	38,952,000	5	5	5	4	3	3	3	3	3	4	4	4	3	3	3	4	3	4	4	4	4
chr10:38952001	chr10	38,952,001	38,953,000	5	5	5	4	3	3	3	3	3	4	4	4	3	3	3	4	3	4	4	4	4
chr10:38953001	chr10	38,953,001	38,954,000	5	5	5	4	3	3	3	3	3	4	4	4	3	3	3	4	3	4	4	4	4
chr10:38954001	chr10	38,954,001	38,955,000	5	5	5	4	3	3	3	3	3	4	4	4	3	3	3	4	3	4	4	4	4
chr10:38955001	chr10	38,955,001	38,956,000	5	5	5	4	3	2	3	3	3	4	4	4	3	3	3	4	3	4	4	4	4
chr13:63063001	chr13	63,063,001	63,064,000	5	5	5	4	3	3	2	2	3	4	4	4	3	3	3	4	4	4	4	3	4
chr13:63064001	chr13	63,064,001	63,065,000	5	5	5	4	3	3	2	2	4	4	4	4	3	3	3	4	4	4	4	3	4
chr4:49242001	chr4	49,242,001	49,243,000	5	4	4	3	3	3	2	3	3	4	4	3	3	5	3	4	3	4	4	3	4
chr6:272001	chr6	272,001	273,000	2	3	3	3	4	5	5	4	5	3	3	4	3	4	4	4	4	4	4	4	3
chr6:273001	chr6	273,001	274,000	2	3	3	3	4	5	5	4	5	3	3	4	3	4	4	4	4	4	4	4	3
chr6:274001	chr6	274,001	275,000	2	3	3	3	4	5	5	4	5	3	3	4	3	4	4	4	4	4	4	4	3
chr6:275001	chr6	275,001	276,000	2	3	3	3	4	5	5	4	5	3	3	4	3	4	4	4	4	4	4	4	3
chr6:276001	chr6	276,001	277,000	2	3	3	3	4	5	5	4	4	3	3	4	3	4	4	4	4	4	4	4	3
chr6:277001	chr6	277,001	278,000	2	3	3	3	4	5	5	4	4	3	3	4	3	4	4	4	4	4	4	4	3
chr6:278001	chr6	278,001	279,000	2	3	3	3	4	5	5	4	4	3	3	4	3	4	4	4	4	4	4	4	3
chr6:279001	chr6	279,001	280,000	2	3	3	3	4	5	5	4	4	3	3	4	3	4	4	4	4	4	4	4	3
chr19:106001	chr19	106,001	107,000	2	2	2	3	3	4	5	4	4	3	3	3	4	3	5	3	5	5	3	4	3
chr19:107001	chr19	107,001	108,000	2	2	2	3	3	4	5	4	4	3	3	3	4	3	5	3	5	5	3	4	3
chr19:109001	chr19	109,001	110,000	2	2	2	3	3	4	5	4	4	3	3	3	4	3	5	3	5	5	3	4	3
chr19:110001	chr19	110,001	111,000	2	2	2	3	3	4	5	4	4	3	3	3	4	3	5	3	5	5	3	4	3
chr3:90538001	chr3	90,538,001	90,539,000	5	5	5	4	3	3	2	3	3	4	4	4	3	3	2	4	3	4	5	3	4
chr3:90539001	chr3	90,539,001	90,540,000	5	5	5	4	3	3	2	3	3	4	4	4	3	3	2	4	3	4	5	3	4
chr4:49199001	chr4	49,199,001	49,200,000	4	5	5	3	3	3	1	2	3	4	4	3	3	4	2	4	3	4	5	4	3
chr4:49580001	chr4	49,580,001	49,581,000	5	4	5	3	2	2	2	2	3	4	4	2	3	4	2	5	2	5	5	4	3
chr5:21480001	chr5	21,480,001	21,481,000	4	5	5	3	1	3	1	3	2	5	5	5	3	5	3	3	4	3	3	2	2
chr10:38956001	chr10	38,956,001	38,957,000	5	5	5	4	3	2	2	3	3	4	3	4	3	3	3	4	3	4	4	4	4
chr10:38957001	chr10	38,957,001	38,958,000	5	5	5	4	3	2	2	3	3	4	3	4	3	3	3	4	3	4	4	4	4
chr10:38958001	chr10	38,958,001	38,959,000	5	5	5	4	3	2	2	3	3	4	3	4	3	3	3	4	3	4	4	4	4
chr10:38959001	chr10	38,959,001	38,960,000	5	5	5	4	3	2	2	3	3	4	3	4	3	3	3	4	3	4	4	4	4
chr10:38960001	chr10	38,960,001	38,961,000	5	5	5	4	3	2	2	3	3	4	3	4	3	3	3	4	3	4	4	4	4
chr13:63062001	chr13	63,062,001	63,063,000	5	5	5	4	3	3	2	2	3	4	4	4	2	3	3	4	4	4	4	3	4
chr4:49239001	chr4	49,239,001	49,240,000	5	4	4	3	3	3	2	3	3	4	4	3	3	5	3	4	3	4	4	3	3
chr4:49240001	chr4	49,240,001	49,241,000	5	4	4	3	3	3	2	3	3	4	4	3	3	5	3	4	3	4	4	3	3
chr4:49241001	chr4	49,241,001	49,242,000	5	4	4	3	3	3	2	3	3	4	4	3	3	5	3	4	3	4	4	3	3

Region	CHR	start	end	HD2	HD3	HD1	BN1	BN2	BN3	BN4	BN5	BN6	LC1	LC2	LC3	LC4	LC5	LC6	LC7	LC8	LC9	LC10	LC10*	LC11
chr14:19006001	chr14	19,006,001	19,007,000	4	4	4	3	3	2	2	3	3	4	4	4	3	4	2	4	4	4	3	3	4
chr6:266001	chr6	266,001	267,000	1	2	3	3	4	5	5	4	5	3	3	4	3	4	4	3	4	4	4	4	3
chr6:267001	chr6	267,001	268,000	1	2	3	3	4	5	5	4	5	3	3	4	3	4	4	3	4	4	4	4	3
chr6:268001	chr6	268,001	269,000	2	2	3	3	4	5	5	4	5	3	3	4	3	4	4	3	4	4	4	4	3
chr6:269001	chr6	269,001	270,000	2	2	3	3	4	5	5	4	5	3	3	4	3	4	4	3	4	4	4	4	3
chr6:270001	chr6	270,001	271,000	2	3	3	3	4	5	5	4	5	3	3	4	3	4	4	3	4	4	4	4	3
chr6:271001	chr6	271,001	272,000	2	3	3	3	4	5	5	4	5	3	3	4	3	4	4	3	4	4	4	4	3
chr6:280001	chr6	280,001	281,000	2	3	3	3	4	5	5	4	4	3	3	4	3	3	4	4	4	4	4	4	3
chr6:281001	chr6	281,001	282,000	2	3	3	3	4	5	5	4	4	3	3	4	3	3	4	4	4	4	4	4	3
chr6:282001	chr6	282,001	283,000	2	3	3	3	4	5	5	4	4	3	3	4	3	3	4	4	4	4	4	4	3
chr6:283001	chr6	283,001	284,000	2	3	3	3	4	5	5	4	4	3	3	4	3	3	4	4	4	4	4	4	3
chr6:284001	chr6	284,001	285,000	2	3	3	3	4	5	5	4	4	3	3	4	3	3	4	4	4	4	4	4	3
chr6:285001	chr6	285,001	286,000	2	3	3	3	4	5	5	4	4	3	3	4	3	3	4	4	4	4	4	4	3
chr6:286001	chr6	286,001	287,000	2	3	3	3	4	5	5	4	4	3	3	4	3	3	4	4	4	4	4	4	3
chr6:287001	chr6	287,001	288,000	2	3	3	3	4	5	5	4	4	3	3	4	3	3	4	4	4	4	4	4	3
chr6:288001	chr6	288,001	289,000	2	3	3	3	4	5	5	4	4	3	3	4	3	3	4	4	4	4	4	4	3
chr6:289001	chr6	289,001	290,000	2	3	3	3	4	5	5	4	4	3	3	4	3	3	4	4	4	4	4	4	3
chr19:111001	chr19	111,001	112,000	2	2	2	3	3	4	5	4	4	3	3	3	4	3	5	3	5	4	3	4	3
chr1:121743001	chr1	121,743,001	121,744,000	5	5	5	3	4	2	2	3	2	4	5	3	3	3	2	3	3	4	5	4	3
chr3:90511001	chr3	90,511,001	90,512,000	5	5	5	4	2	2	2	2	3	4	4	3	3	3	2	4	3	4	5	3	4
chr4:49201001	chr4	49,201,001	49,202,000	4	5	5	3	3	3	1	2	3	3	4	3	3	4	3	3	3	4	5	4	3
chr4:49202001	chr4	49,202,001	49,203,000	4	5	5	3	3	3	1	2	3	3	4	3	3	4	3	3	3	4	5	4	3
chr5:34179001	chr5	34,179,001	34,180,000	5	5	5	4	2	2	1	2	2	5	4	3	4	4	3	4	4	4	3	2	2
chr5:34180001	chr5	34,180,001	34,181,000	5	5	5	4	2	2	1	3	2	5	4	3	4	4	3	4	4	4	3	2	2
chr5:34181001	chr5	34,181,001	34,182,000	5	5	5	4	2	2	1	3	2	5	4	3	4	4	3	4	4	4	3	2	2
chr5:34182001	chr5	34,182,001	34,183,000	5	5	5	4	2	2	1	3	2	5	4	3	4	4	3	4	4	4	3	2	2
chr5:34183001	chr5	34,183,001	34,184,000	5	5	5	4	2	2	1	3	2	5	4	3	4	4	3	4	4	4	3	2	2
chr5:34184001	chr5	34,184,001	34,185,000	5	5	5	4	2	2	1	3	2	5	4	3	4	4	3	4	4	4	3	2	2
chr5:34185001	chr5	34,185,001	34,186,000	5	5	5	4	2	2	1	3	2	5	4	3	4	4	3	4	4	4	3	2	2
chr5:34186001	chr5	34,186,001	34,187,000	5	5	5	4	2	2	1	3	2	5	4	3	4	4	3	4	4	4	3	2	2
chr5:34187001	chr5	34,187,001	34,188,000	5	5	5	4	2	2	1	3	2	5	4	3	4	4	3	4	4	4	3	2	2
chr5:34188001	chr5	34,188,001	34,189,000	5	5	5	4	2	2	1	3	2	5	4	3	4	4	3	4	4	4	3	2	2
chr5:34189001	chr5	34,189,001	34,190,000	5	5	5	4	2	2	1	3	2	5	4	3	4	4	3	4	4	4	3	2	2
chr5:34190001	chr5	34,190,001	34,191,000	5	5	5	4	2	2	1	3	2	5	4	3	4	4	3	4	4	4	3	2	2
chr5:34191001	chr5	34,191,001	34,192,000	5	5	5	4	2	2	1	3	2	5	4	3	4	4	3	4	4	4	3	2	2
chr5:34192001	chr5	34,192,001	34,193,000	5	5	5	4	2	2	1	3	2	5	4	3	4	4	3	4	4	4	3	2	2
chr8:144125001	chr8	144,125,001	144,126,000	5	5	5	4	2	3	2	2	3	2	4	3	3	3	2	4	3	4	5	4	5
chr10:38961001	chr10	38,961,001	38,962,000	5	5	5	4	3	2	2	3	3	4	3	4	3	3	2	4	3	4	4	4	4
chr10:38962001	chr10	38,962,001	38,963,000	5	5	5	4	3	2	2	3	3	4	3	4	3	3	2	4	3	4	4	4	4
chr13:63060001	chr13	63,060,001	63,061,000	5	4	5	4	2	3	2	2	3	4	4	4	2	3	3	4	4	4	4	2	4
chr17:21850001	chr17	21,850,001	21,851,000	5	5	5	3	2	3	2	3	3	3	3	3	3	3	3	5	3	3	5	4	4
chr1:121774001	chr1	121,774,001	121,775,000	5	5	4	3	4	2	1	2	2	3	5	3	4	3	2	4	3	5	4	3	3
chr1:121775001	chr1	121,775,001	121,776,000	5	5	4	3	4	2	1	2	2	3	5	3	4	3	2	4	3	5	4	3	3
chr4:49220001	chr4	49,220,001	49,221,000	5	3	4	3	3	3	2	2	3	3	4	3	3	4	3	4	3	4	4	4	3
chr14:18999001	chr14	18,999,001	19,000,000	4	4	4	3	3	2	2	3	3	4	4	4	3	4	2	4	4	3	3	3	4
chr14:19000001	chr14	19,000,001	19,001,000	4	4	4	3	3	2	2	3	3	4	4	4	3	4	2	4	4	3	3	3	4
chr14:19001001	chr14	19,001,001	19,002,000	4	4	4	3	3	2	2	3	3	4	4	4	3	4	2	4	4	3	3	3	4
chr14:19002001	chr14	19,002,001	19,003,000	4	4	4	3	3	2	2	3	3	4	4	4	3	4	2	4	4	3	3	3	4
chr14:19003001	chr14	19,003,001	19,004,000	4	4	4	3	3	2	2	3	3	4	4	4	3	4	2	4	4	3	3	3	4
chr14:19004001	chr14	19,004,001	19,005,000	4	4	4	3	3	2	2	3	3	4	4	4	3	4	2	4	4	3	3	3	4
chr14:19005001	chr14	19,005,001	19,006,000	4	4	4	3	3	2	2	3	3	4	4	4	3	4	2	4	4	3	3	3	4
chr14:19007001	chr14	19,007,001	19,008,000	4	4	4	3	3	2	2	3	3	4	4	4	3	4	2	4	3	4	3	3	4
chr14:19008001	chr14	19,008,001	19,009,000	4	4	4	3	3	2	2	3	3	4	4	4	3	4	2	4	3	4	3	3	4
chr14:19009001	chr14	19,009,001	19,010,000	4	4	4	3	3	2	2	3	3	4	4	4	3	4	2	4	3	4	3	3	4
chr14:19010001	chr14	19,010,001	19,011,000	4	4	4	3	3	2	2	3	3	4	4	4	3	4	2	4	3	4	3	3	4
chr14:19011001	chr14	19,011,001	19,012,000	4	4	4	3	3	2	2	3	3	4	4	4	3	4	2	4	3	4	3	3	4
chr14:19012001	chr14	19,012,001	19,013,000	4	4	4	3	3	2	2	3	3	4	4	4	3	4	2	4	3	4	3	3	4
chr14:19013001	chr14	19,013,001	19,014,000	4	4	4	3	3	2	2	3	3	4	4	4	3	4	2	4	3	4	3	3	4
chr14:19014001	chr14	19,014,001	19,015,000	4	4	4	3	3	2	2	3	3	4	4	4	3	4	2	4	3	4	3	3	4
chr19:24357001	chr19	24,357,001	24,358,000	4	4	4	3	3	2	3	3	3	4	4	4	3	3	4	4	3	4	3	3	3
chr19:24358001	chr19	24,358,001	24,359,000	4	4	4	3	3	3	3	3	3	4	4	4	3	3	4	4	3	4	3	3	3
chr19:24359001	chr19	24,359,001	24,360,000	4	4	4	3	3	3	3	3	3	4	4	4	3	3	4	4	3	4	3	3	3
chr19:24360001	chr19	24,360,001	24,361,000	4	4	4	3	3	3	3	3	3	4	4	4	3	3	4	4	3	4	3	3	3
chr19:24361001	chr19	24,361,001	24,362,000	4	4	4	3	3	3	3	3	3	4	4	4	3	3	4	4	3	4	3	3	3
chr6:265001	chr6	265,001	266,000	1	2	3	3	4	5	5	4	5	3	3	4	3	4	4	3	4	4	3	4	3
chr1:133001	chr1	133,001	134,000	3	4	2	4	4	5	4	4	5	3	3	4	4	4	4	3	3	3	3	4	4
chr19:112001	chr19	112,001	113,000	2	2	2	3	3	4	5	4	4	3	3	3	4	3	5	3	5	4	3	3	3
chr19:113001	chr19	113,001	114,000	2	2	2	3	3	4	5	4	4	3	3	3	4	3	5	3	5	4	3	3	3
chr1:121744001	chr1	121,744,001	121,745,000	5	5	5	3	4	2	2	3	2	4	4	3	3	3	2	3	3	4	5	4	3
chr1:144101001	chr1	144,101,001	144,102,000	4	4	5	3	3	2	2	2	2	4	4	3	2	3	2	5	3	5	4	3	3
chr1:144102001	chr1	144,102,001	144,103,000	4	4	5	3	3	2	1	2	2	4	4	3	2	3	2	5	3	5	4	3	3
chr1:144103001	chr1	144,103,001	144,104,000	4	4	5	3	3	2	1	2	2	4	4	3	2	3	2	5	3	5	4	3	3
chr1:144104001	chr1	144,104,001	144,105,000	4	4	5	3	3	2	1	2	2	4	4	3	2	3	2	5	3	5	4	3	3
chr4:49200001	chr4	49,200,001	49,201,000	4	5	5	3	3	3	1	2	3	3	4	3	3	4	2	3	3	4	5	4	3
chr4:49708001	chr4	49,708,001	49,709,000	5	5	5	2	3	1	1	2	1	5	5	5	2	1	2	3	5	5	2	1	5
chr4:190022001	chr4	190,022,001	190,023,000	5	5	5	3	3	2	1	2	3	3	4	3	3	3	2	4	3	5	5	3	3
chr5:34178001	chr5	34,178,001	34,179,000	5	5	5	4	2	2	2	2	2	5	4	3	4	4	3	4	4	4	2	2	2
chr5:34193001	chr5	34,193,001	34,194,000	5	5	5	4	2	3	1	3	2	4	4	3	4	4	3	4	4	4	3	2	2
chr5:34194001	chr5	34,194,001	34,195,000	5	5	5	4	2	3	2	3	2	4	4	3	4	4	3	4	4	4	3	2	2
chr5:34195001	chr5	34,195,001	34,196,000	5	5	5	4	2	3	2	3	2	4	4	3	4	4	3	4	4	4	3	2	2
chr5:34196001	chr5	34,196,001	34,197,000	5	5	5	4	2	3	2	3	2	4	4	3	4	4	3	4	4	4	3	2	2
chr9:38989001	chr9	38,989,001	38,990,000	5	4	5	4	3	3	2	3	3	4	4	3	3	3	3	4	3	3	4	3	4
chr9:63881001	chr9	63,881,001	63,882,000	4	4	5	3	3	3	3	3	3	3	4	4	3	3	3	4	3	4	4	3	3
chr9:63882001	chr9	63,882,001	63,883,000	4	4	5	3	3	3	3	3	3	3	4	4	3	3	3	4	3	4	4	3	3
chr9:63883001	chr9	63,883,001	63,884,000	4	4	5	3	3	3	3	3	3	3	4	4	3	3	3	4	3	4	4	3	3
chr9:63884001	chr9	63,884,001	63,885,000	4	4	5	3	3	3	3	3	3	3	4	4	3	3	3	4	3	4	4	3	3
chr9:63885001	chr9	63,885,001	63,886,000	4	4	5	3	3	3	3	3	3	3	4	4	3	3	3	4	3	4	4	3	3
chr9:63886001	chr9	63,886,001	63,887,000	4	4	5	3	3	3	3	3	3	3	4	4	3	3	3	4	3	4	4	3	3
chr9:63887001	chr9	63,887,001	63,888,000	4	4	5	3	3	3	3	3	3	3	4	4	3	3	3	4	3	4	4	3	3
chr9:63888001	chr9	63,888,001	63,889,000	4	4	5	3	3	3	3	3	3	3	4	4	3	3	3	4	3	4	4	3	3
chr9:63889001	chr9	63,889,001	63,890,000	4	4	5	3	3	3	3	3	3	3	4	4	3	3	3	4	3	4	4	3	3
chr3:90533001	chr3	90,533,001	90,534,000	5	5	4	4	3	3	2	3	3	4	4	4	3	3	2	4	3	4	3	3	4
chr3:90534001	chr3	90,534,001	90,535,000	5	5	4	4	3	3	2	3	3	4	4	4	3	3	2	4	3	4	3	3	4
chr3:90535001	chr3	90,535,001	90,536,000	5	5	4	4	3	3	2	3	3	4	4	4	3	3	2	4	3	4	3	3	4
chr9:66816001	chr9	66,816,001	66,817,000	5	4	4	2	3	3	2	3	2	4	4	3	3	4	3	4	4	3	3	3	3
chr9:66817001	chr9	66,817,001	66,818,000	5	4	4	2	3	3	2	3	2	4	4	3	3	4	3	4	4	3	3	3	3
chr9:66818001	chr9	66,818,001	66,819,000	5	4	4	2	3	3	2	3	2	4	4	3	3	4	3	4	4	3	3	3	3
chr9:66819001	chr9	66,819,001	66,820,000	5	4	4	2	3	3	2	3	2	4	4	3	3	4	3	4	4	3	3	3	3
chr9:66820001	chr9	66,820,001	66,821,000	5	4	4	2	3	3	2	3	2	4	4	3	3	4	3	4	4	3	3	3	3
chr9:66821001	chr9	66,821,001	66,822,000	5	4	4	2	3	3	2	3	2	4	4	3	3	4	3	4	4	3	3	3	3
chr9:66822001	chr9	66,822,001	66,823,000	5	4	4	2	3	3	2	3	2	4	4	3	3	4	3	4	4	3	3	3	3
chr9:66823001	chr9	66,823,001	66,824,000	5	4	4	2	3	3	2	3	2	4	4	3	3	4	3	4	4	3	3	3	3
chr9:66824001	chr9	66,824,001	66,825,000	5	4	4	2	3	4	2	3	2	4	4	3	3	4	3	4	4	3	3	3	3
chr9:66825001	chr9	66,825,001	66,826,000	5	4	4	2	3	4	2	3	2	4	4	3	3	4	3	4	4	3	3	3	3
chr9:66826001	chr9	66,826,001	66,827,000	5	4	4	2	3	4	2	3	2	4	4	3	3	4	3	4	4	3	3	3	3
chr19:24362001	chr19	24,362,001	24,363,000	4	4	4	3	3	3	3	3	3	4	4	4	3	3	4	3	3	4	3	3	3
chr6:263001	chr6	263,001	264,000	2	2	3	3	4	5	5	4	5	3	3	4	2	4	4	3	4	4	3	4	3
chr6:264001	chr6	264,001	265,000	2	2	3	3	4	5	5	4	5	3	3	4	2	4	4	3	4	4	3	4	3
chr1:16676001	chr1	16,676,001	16,677,000	3	3	2	3	5	5	4	4	4	4	4	3	3	3	4	4	3	3	3	4	3
chr4:49203001	chr4	49,203,001	49,204,000	2	3	2	3	3	3	2	2	3	3	4	3	3	4	3	3	3	4	4	4	3
chr4:49204001	chr4	49,204,001	49,205,000	2	3	2	3	3	3	2	2	3	3	4	3	3	4	3	3	3	4	4	4	3
chr4:49205001	chr4	49,205,001	49,206,000	2	3	2	3	3	3	2	2	3	3	4	3	3	4	3	3	3	4	4	4	3
chr6:258001	chr6	258,001	259,000	2	2	2	2	4	5	5	4	4	3	3	4	2	4	4	3	4	4	3	4	3

Region	CHR	start	end	HD2	HD3	HD1	BN1	BN2	BN3	BN4	BN5	BN6	LC1	LC2	LC3	LC4	LC5	LC6	LC7	LC8	LC9	LC10	LC10*	LC11
chr6:259001	chr6	259,001	260,000	2	2	2	2	4	5	5	4	4	3	3	4	2	4	4	3	4	4	3	4	3
chr6:260001	chr6	260,001	261,000	2	2	2	3	4	5	5	4	4	3	3	4	2	4	4	3	4	4	3	4	3
chr6:261001	chr6	261,001	262,000	2	2	2	3	4	5	5	4	5	3	3	4	2	4	4	3	4	4	3	4	3
chr6:262001	chr6	262,001	263,000	2	2	2	3	4	5	5	4	5	3	3	4	2	4	4	3	4	4	3	4	3
chr1:125079001	chr1	125,079,001	125,080,000	4	4	5	3	3	2	2	2	3	4	4	4	3	3	2	4	3	3	4	3	3
chr1:125080001	chr1	125,080,001	125,081,000	4	4	5	3	3	2	2	2	3	4	4	4	3	3	2	4	3	3	4	3	3
chr1:125081001	chr1	125,081,001	125,082,000	4	4	5	3	3	2	2	2	3	4	4	4	3	3	2	4	3	3	4	3	3
chr1:125082001	chr1	125,082,001	125,083,000	4	4	5	3	3	2	2	2	3	4	4	4	3	3	2	4	3	3	4	3	3
chr1:125083001	chr1	125,083,001	125,084,000	4	4	5	3	3	2	2	2	3	4	4	4	3	3	2	4	3	3	4	3	3
chr1:125084001	chr1	125,084,001	125,085,000	4	4	5	3	3	2	2	2	3	4	4	4	3	3	2	4	3	3	4	3	3
chr1:125085001	chr1	125,085,001	125,086,000	4	4	5	3	3	2	2	2	3	4	4	4	3	3	2	4	3	3	4	3	3
chr2:87420001	chr2	87,420,001	87,421,000	5	5	5	3	3	4	2	3	3	5	4	3	4	3	3	4	3	2	3	3	3
chr4:190021001	chr4	190,021,001	190,022,000	5	5	5	3	3	2	1	2	2	2	4	3	3	3	2	4	3	5	5	3	3
chr9:38990001	chr9	38,990,001	38,991,000	5	4	5	4	3	3	2	3	3	4	4	3	3	3	3	4	3	3	4	3	3
chr9:38991001	chr9	38,991,001	38,992,000	5	4	5	4	3	3	2	3	3	4	4	3	3	3	3	4	3	3	4	3	3
chr9:38992001	chr9	38,992,001	38,993,000	5	4	5	4	3	3	2	3	3	4	4	3	3	3	3	4	3	3	4	3	3
chr9:42366001	chr9	42,366,001	42,367,000	4	4	5	2	3	3	1	1	3	4	5	3	3	3	2	4	3	3	4	3	3
chr9:60621001	chr9	60,621,001	60,622,000	5	5	5	3	4	3	2	4	4	4	3	3	3	2	3	5	3	4	4	3	3
chr9:60668001	chr9	60,668,001	60,669,000	5	4	5	4	3	3	2	3	3	3	3	3	3	3	3	5	3	3	4	3	4
chr9:60669001	chr9	60,669,001	60,670,000	5	4	5	4	3	3	2	3	3	3	3	3	3	3	3	5	3	3	4	3	4
chr9:60670001	chr9	60,670,001	60,671,000	5	4	5	4	3	3	2	3	3	3	3	3	3	3	3	5	3	3	4	3	4
chr9:60671001	chr9	60,671,001	60,672,000	5	4	5	4	3	3	2	3	3	3	3	3	3	3	3	5	3	3	4	3	4
chr9:63878001	chr9	63,878,001	63,879,000	4	4	5	3	3	3	3	3	3	3	4	4	3	3	3	4	3	4	3	3	3
chr9:63879001	chr9	63,879,001	63,880,000	4	4	5	3	3	3	3	3	3	3	4	4	3	3	3	4	3	4	3	3	3
chr9:63880001	chr9	63,880,001	63,881,000	4	4	5	3	3	3	3	3	3	3	4	4	3	3	3	4	3	4	3	3	3
chr9:63890001	chr9	63,890,001	63,891,000	4	4	5	3	3	3	3	3	3	3	4	4	3	3	3	3	3	4	4	3	3
chr9:63891001	chr9	63,891,001	63,892,000	4	4	5	3	3	3	3	3	3	3	4	4	3	3	3	3	3	4	4	3	3
chr9:63892001	chr9	63,892,001	63,893,000	4	4	5	3	3	3	3	3	3	3	4	4	3	3	3	3	3	4	4	3	3
chr9:63893001	chr9	63,893,001	63,894,000	4	4	5	3	3	3	3	3	3	3	4	4	3	3	3	3	3	4	4	3	3
chr3:90519001	chr3	90,519,001	90,520,000	5	5	4	4	3	2	2	2	3	4	4	3	3	3	2	4	3	4	3	3	4
chr3:90520001	chr3	90,520,001	90,521,000	5	5	4	4	3	2	2	3	3	4	4	3	3	3	2	4	3	4	3	3	4
chr3:90521001	chr3	90,521,001	90,522,000	5	5	4	4	3	2	2	3	3	4	4	3	3	3	2	4	3	4	3	3	4
chr3:90522001	chr3	90,522,001	90,523,000	5	5	4	4	3	2	2	3	3	4	4	3	3	3	2	4	3	4	3	3	4
chr3:90523001	chr3	90,523,001	90,524,000	5	5	4	4	3	2	2	3	3	4	4	3	3	3	2	4	3	4	3	3	4
chr3:90524001	chr3	90,524,001	90,525,000	5	5	4	3	3	2	2	3	3	4	4	3	3	3	2	4	3	4	3	3	4
chr3:90525001	chr3	90,525,001	90,526,000	5	5	4	3	3	2	2	3	3	4	4	3	3	3	2	4	3	4	3	3	4
chr3:90526001	chr3	90,526,001	90,527,000	5	5	4	3	3	2	2	3	3	4	4	3	3	3	2	4	3	4	3	3	4
chr3:90527001	chr3	90,527,001	90,528,000	5	5	4	3	3	2	2	3	3	4	4	3	3	3	2	4	3	4	3	3	4
chr3:90528001	chr3	90,528,001	90,529,000	5	5	4	4	3	2	2	3	3	4	4	3	3	3	2	4	3	4	3	3	4
chr3:90529001	chr3	90,529,001	90,530,000	5	5	4	4	3	3	2	3	3	4	4	3	3	3	2	4	3	4	3	3	4
chr3:90530001	chr3	90,530,001	90,531,000	5	5	4	4	3	3	2	3	3	4	4	3	3	3	2	4	3	4	3	3	4
chr3:90531001	chr3	90,531,001	90,532,000	5	5	4	4	3	3	2	3	3	4	4	3	3	3	2	4	3	4	3	3	4
chr3:90532001	chr3	90,532,001	90,533,000	5	5	4	4	3	3	2	3	3	4	4	3	3	3	2	4	3	4	3	3	4
chr9:39060001	chr9	39,060,001	39,061,000	5	4	4	3	4	3	2	3	3	3	4	3	3	4	3	3	4	3	4	3	3
chr9:39061001	chr9	39,061,001	39,062,000	5	4	4	3	4	3	2	3	3	3	4	3	3	4	3	3	4	3	4	3	3
chr9:39062001	chr9	39,062,001	39,063,000	5	4	4	3	4	3	2	3	3	3	4	3	3	4	3	3	4	3	4	3	3
chr9:39063001	chr9	39,063,001	39,064,000	5	4	4	3	4	3	2	3	3	3	4	3	3	4	3	3	4	3	4	3	3
chr9:39064001	chr9	39,064,001	39,065,000	5	4	4	3	4	3	2	3	3	3	4	3	3	4	3	3	4	3	4	3	3
chr9:63876001	chr9	63,876,001	63,877,000	4	4	4	3	3	3	3	3	3	3	4	4	3	3	3	4	3	4	3	3	3
chr9:63877001	chr9	63,877,001	63,878,000	4	4	4	3	3	3	3	3	3	3	4	4	3	3	3	4	3	4	3	3	3
chr9:66032001	chr9	66,032,001	66,033,000	5	4	4	4	3	3	2	3	3	3	4	3	3	4	3	4	4	3	3	3	3
chr9:66033001	chr9	66,033,001	66,034,000	5	4	4	4	3	3	2	3	3	3	4	3	3	4	3	4	4	3	3	3	3
chr9:66814001	chr9	66,814,001	66,815,000	5	4	4	2	3	3	2	3	2	3	4	3	4	4	3	4	3	3	3	3	3
chr9:66815001	chr9	66,815,001	66,816,000	5	4	4	2	3	3	2	3	2	4	4	3	3	4	3	4	3	3	3	3	3
chr1:134001	chr1	134,001	135,000	3	4	2	4	4	5	4	4	5	3	3	4	3	4	4	3	3	3	3	4	3
chr1:135001	chr1	135,001	136,000	3	4	2	4	4	5	4	4	5	3	3	4	3	4	4	3	3	3	3	4	3
chr1:136001	chr1	136,001	137,000	3	4	2	4	4	5	4	4	5	3	3	4	3	4	4	3	3	3	3	4	3
chr1:137001	chr1	137,001	138,000	2	4	2	4	4	5	4	4	5	3	3	4	3	4	4	3	3	3	3	4	3
chr1:16677001	chr1	16,677,001	16,678,000	3	3	2	3	5	5	4	4	4	4	3	3	3	3	4	4	3	3	3	4	3
chr19:114001	chr19	114,001	115,000	2	2	2	3	3	4	5	4	4	3	3	3	4	3	3	3	5	4	3	3	3
chr2:94562001	chr2	94,562,001	94,563,000	4	5	5	3	3	3	2	3	3	4	4	3	3	3	2	4	3	3	3	3	4
chr2:94563001	chr2	94,563,001	94,564,000	4	5	5	3	3	3	2	3	3	4	4	3	3	3	2	4	3	3	3	3	4
chr2:94564001	chr2	94,564,001	94,565,000	4	5	5	3	3	3	2	3	3	4	4	3	3	3	2	4	3	3	3	3	4
chr2:94565001	chr2	94,565,001	94,566,000	5	5	5	3	3	3	2	3	3	4	4	3	3	3	2	4	3	3	3	3	4
chr2:94566001	chr2	94,566,001	94,567,000	5	5	5	3	3	3	2	3	3	4	4	3	3	3	2	4	3	3	3	3	4
chr2:94567001	chr2	94,567,001	94,568,000	5	5	5	3	3	2	2	3	3	4	4	3	3	3	2	4	3	3	3	3	4
chr3:90612001	chr3	90,612,001	90,613,000	5	5	5	4	3	2	2	3	3	4	4	3	3	3	2	4	3	3	4	3	3
chr3:90613001	chr3	90,613,001	90,614,000	5	5	5	4	3	2	2	3	3	4	4	3	3	3	2	4	3	3	4	3	3
chr3:90614001	chr3	90,614,001	90,615,000	5	5	5	4	3	2	2	3	3	4	4	3	3	3	2	4	3	3	4	3	3
chr3:90615001	chr3	90,615,001	90,616,000	5	5	5	4	3	2	2	3	3	4	4	3	3	3	2	4	3	3	4	3	3
chr3:90616001	chr3	90,616,001	90,617,000	5	5	5	4	3	2	2	3	3	4	4	3	3	3	2	4	3	3	4	3	3
chr3:90617001	chr3	90,617,001	90,618,000	5	5	5	4	3	2	2	3	3	4	4	3	3	3	2	4	3	3	4	3	3
chr9:39048001	chr9	39,048,001	39,049,000	3	5	5	3	2	3	2	3	3	3	4	4	4	3	3	3	3	3	3	3	3
chr16:34071001	chr16	34,071,001	34,072,000	5	5	5	3	3	2	2	3	3	3	3	3	3	3	3	5	2	5	3	3	3
chr17:21849001	chr17	21,849,001	21,850,000	5	4	5	3	2	3	2	2	3	3	3	3	3	3	3	5	3	3	3	3	4
chr2:94560001	chr2	94,560,001	94,561,000	4	5	4	3	3	2	2	3	3	4	4	3	3	3	2	4	3	3	3	3	4
chr2:94561001	chr2	94,561,001	94,562,000	4	5	4	3	3	2	2	3	3	4	4	3	3	3	2	4	3	3	3	3	4
chr3:90683001	chr3	90,683,001	90,684,000	5	4	4	4	3	3	2	3	3	4	3	3	3	3	3	4	3	3	4	3	3
chr3:90684001	chr3	90,684,001	90,685,000	5	4	4	3	3	3	2	3	3	4	3	3	3	3	3	4	3	3	4	3	3
chr3:90685001	chr3	90,685,001	90,686,000	5	4	4	3	3	3	2	3	3	4	3	3	3	3	3	4	3	3	4	3	3
chr3:90686001	chr3	90,686,001	90,687,000	5	4	4	3	3	3	2	3	3	4	3	3	3	3	3	4	3	3	4	3	3
chr9:38998001	chr9	38,998,001	38,999,000	4	4	4	3	3	3	2	3	3	3	4	3	3	4	3	4	3	3	3	3	3
chr9:38999001	chr9	38,999,001	39,000,000	4	4	4	3	3	3	2	3	3	3	4	3	3	4	3	4	3	3	3	3	3
chr9:39000001	chr9	39,000,001	39,001,000	4	4	4	3	3	3	2	3	3	3	4	3	3	4	3	4	3	3	3	3	3
chr9:39001001	chr9	39,001,001	39,002,000	4	4	4	3	3	3	2	3	3	3	4	3	3	4	3	4	3	3	3	3	3
chr9:39059001	chr9	39,059,001	39,060,000	5	4	4	3	4	3	2	3	3	3	3	3	3	4	3	3	4	3	4	3	3
chr9:39068001	chr9	39,068,001	39,069,000	5	4	4	3	3	3	2	3	3	3	4	3	3	4	3	3	4	3	3	3	3
chr9:63868001	chr9	63,868,001	63,869,000	4	4	4	3	3	3	3	3	3	3	4	4	3	3	3	3	3	3	4	3	3
chr9:63875001	chr9	63,875,001	63,876,000	4	4	4	3	3	3	3	3	3	3	3	4	3	3	3	4	3	4	3	3	3
chr9:66828001	chr9	66,828,001	66,829,000	5	4	4	2	3	4	2	3	2	3	4	3	3	4	2	4	4	3	3	3	3
chr9:66829001	chr9	66,829,001	66,830,000	5	4	4	2	3	4	2	3	2	3	4	3	3	4	2	4	4	3	3	3	3
chr12:37726001	chr12	37,726,001	37,727,000	4	5	4	3	3	2	2	3	3	3	4	4	3	3	3	4	3	4	2	3	3
chr12:37727001	chr12	37,727,001	37,728,000	4	5	4	3	3	2	2	3	3	3	4	4	3	3	3	4	3	4	2	3	3
chr12:37728001	chr12	37,728,001	37,729,000	4	5	4	3	3	2	2	3	3	3	4	4	3	3	3	4	3	4	2	3	3
chr12:37729001	chr12	37,729,001	37,730,000	4	5	4	3	3	2	2	3	3	3	4	4	3	3	3	4	3	4	2	3	3
chr12:37730001	chr12	37,730,001	37,731,000	4	5	4	3	3	2	2	3	3	3	4	4	3	3	3	4	3	4	2	3	3
chr12:37731001	chr12	37,731,001	37,732,000	4	5	4	3	3	2	2	3	3	3	4	4	3	3	3	4	3	4	2	3	3
chr6:382001	chr6	382,001	383,000	2	2	2	4	4	4	4	3	3	3	3	3	3	3	4	3	3	3	4	4	3
chr19:119001	chr19	119,001	120,000	1	2	2	3	3	4	5	3	3	3	3	3	4	3	3	3	5	4	2	3	3
chr1:144105001	chr1	144,105,001	144,106,000	4	4	5	3	3	2	1	2	2	4	4	3	2	3	3	3	3	3	4	3	3
chr2:94568001	chr2	94,568,001	94,569,000	5	5	5	3	3	2	2	3	3	4	3	3	3	3	2	4	3	3	3	3	4
chr3:90512001	chr3	90,512,001	90,513,000	5	5	5	4	2	2	2	2	3	4	4	3	3	3	2	4	3	2	3	3	4
chr3:90513001	chr3	90,513,001	90,514,000	5	5	5	4	2	2	2	2	3	4	4	3	3	3	2	4	3	2	3	3	4
chr3:90610001	chr3	90,610,001	90,611,000	5	5	5	4	3	2	2	3	3	4	3	3	3	3	2	4	3	3	4	3	3
chr3:90611001	chr3	90,611,001	90,612,000	5	5	5	4	3	2	2	3	3	4	3	3	3	3	2	4	3	3	4	3	3
chr6:168840001	chr6	168,840,001	168,841,000	5	5	5	2	2	2	2	2	1	5	5	4	4	2	2	3	2	5	2	2	2
chr9:38993001	chr9	38,993,001	38,994,000	5	4	5	4	3	3	2	3	3	3	4	3	3	3	3	4	3	3	3	3	3
chr9:39025001	chr9	39,025,001	39,026,000	5	5	5	4	2	3	2	3	3	3	4	3	3	3	3	4	3	3	3	3	3
chr9:39026001	chr9	39,026,001	39,027,000	5	5	5	4	2	3	2	3	3	3	4	3	3	3	3	4	3	3	3	3	3
chr9:39027001	chr9	39,027,001	39,028,000	5	5	5	4	2	3	2	3	3	3	4	3	3	3	3	4	3	3	3	3	3
chr9:39028001	chr9	39,028,001	39,029,000	5	5	5	4	2	3	2	3	3	3	4	3	3	3	3	4	3	3	3	3	3
chr9:39029001	chr9	39,029,001	39,030,000	5	5	5	4	3	3	2	3	3	3	4	3	3	3	3	4	3	3	3	3	3
chr9:39030001	chr9	39,030,001	39,031,000	5	5	5	4	3	3	2	3	3	3	4	3	3	3	3	4	3	3	3	3	3

Region	CHR	start	end	HD2	HD3	HD1	BN1	BN2	BN3	BN4	BN5	BN6	LC1	LC2	LC3	LC4	LC5	LC6	LC7	LC8	LC9	LC10	LC10*	LC11
chr9:39038001	chr9	39,038,001	39,039,000	3	5	5	3	2	3	2	3	3	3	4	4	3	3	3	4	3	3	3	2	3
chr9:60620001	chr9	60,620,001	60,621,000	5	5	5	3	4	3	2	4	4	4	3	3	3	2	3	5	3	4	2	3	3
chr9:60665001	chr9	60,665,001	60,666,000	5	4	5	4	3	3	2	3	3	3	3	3	3	2	3	5	3	3	3	3	4
chr9:60666001	chr9	60,666,001	60,667,000	5	4	5	4	3	3	2	3	3	3	3	3	3	2	3	5	3	3	3	3	4
chr9:60672001	chr9	60,672,001	60,673,000	5	4	5	4	3	3	2	3	3	3	3	3	3	3	3	5	3	3	4	3	2
chr17:21848001	chr17	21,848,001	21,849,000	5	4	5	3	2	2	2	2	3	3	3	3	3	3	3	5	3	3	3	3	3
chr2:94205001	chr2	94,205,001	94,206,000	4	4	4	3	3	3	3	3	3	3	4	4	3	3	3	3	3	3	3	3	3
chr2:94206001	chr2	94,206,001	94,207,000	4	4	4	3	4	3	3	3	3	3	4	4	3	3	3	3	3	3	3	3	3
chr2:94559001	chr2	94,559,001	94,560,000	4	4	4	3	3	2	2	3	3	4	4	3	3	3	2	4	3	3	3	3	3
chr2:153222001	chr2	153,222,001	153,223,000	5	5	4	2	2	2	2	2	2	4	4	4	2	4	2	4	2	4	2	2	4
chr3:90514001	chr3	90,514,001	90,515,000	5	5	4	4	2	2	2	2	3	4	4	3	3	3	2	4	3	2	3	3	4
chr3:90515001	chr3	90,515,001	90,516,000	5	5	4	4	2	2	2	2	3	4	4	3	3	3	2	4	3	2	3	3	4
chr3:90516001	chr3	90,516,001	90,517,000	5	5	4	4	2	2	2	2	3	4	4	3	3	3	2	4	3	2	3	3	4
chr3:90517001	chr3	90,517,001	90,518,000	5	5	4	4	2	2	2	2	3	4	4	3	3	3	2	4	3	2	3	3	4
chr3:90518001	chr3	90,518,001	90,519,000	5	5	4	4	2	2	2	2	3	4	4	3	3	3	2	4	3	2	3	3	4
chr3:90682001	chr3	90,682,001	90,683,000	5	4	4	3	3	3	2	3	3	4	3	2	3	3	3	4	3	3	4	3	3
chr3:90688001	chr3	90,688,001	90,689,000	4	4	4	3	3	3	2	3	3	4	3	3	3	3	3	4	3	3	3	3	3
chr7:57932001	chr7	57,932,001	57,933,000	4	4	4	3	3	3	2	3	3	3	3	3	3	3	3	3	3	4	3	3	4
chr7:57933001	chr7	57,933,001	57,934,000	4	4	4	3	3	3	2	3	3	3	3	3	3	3	3	3	3	4	3	3	4
chr7:57934001	chr7	57,934,001	57,935,000	4	4	4	3	3	3	2	3	3	3	3	3	3	3	3	3	3	4	3	3	4
chr7:57935001	chr7	57,935,001	57,936,000	4	4	4	3	3	3	2	3	3	3	3	3	3	3	3	3	3	4	3	3	4
chr7:57936001	chr7	57,936,001	57,937,000	4	4	4	3	3	3	2	3	3	3	3	3	3	3	3	3	3	4	3	3	4
chr7:57937001	chr7	57,937,001	57,938,000	4	4	4	3	3	3	2	3	3	3	3	3	3	3	3	3	3	4	3	3	4
chr9:38976001	chr9	38,976,001	38,977,000	5	4	4	4	3	4	2	3	3	3	4	3	3	3	3	4	3	3	3	3	3
chr9:38977001	chr9	38,977,001	38,978,000	5	4	4	4	3	4	2	3	3	3	4	3	3	3	3	4	3	3	3	3	3
chr9:38978001	chr9	38,978,001	38,979,000	5	4	4	4	3	4	2	3	3	3	4	3	3	3	3	4	3	3	3	3	3
chr9:38979001	chr9	38,979,001	38,980,000	5	4	4	4	3	4	2	3	3	3	4	3	3	3	3	4	3	3	3	3	3
chr9:39069001	chr9	39,069,001	39,070,000	5	4	4	3	3	3	2	3	3	3	4	3	3	4	3	3	3	3	3	3	3
chr9:39070001	chr9	39,070,001	39,071,000	5	4	4	3	3	3	2	3	3	3	4	3	3	4	3	3	3	3	3	3	3
chr9:41093001	chr9	41,093,001	41,094,000	5	4	4	3	3	3	2	3	3	3	4	3	3	4	2	4	3	3	3	3	3
chr9:41094001	chr9	41,094,001	41,095,000	5	4	4	3	3	3	2	3	3	3	4	3	3	4	2	4	3	3	3	3	3
chr9:41095001	chr9	41,095,001	41,096,000	5	4	4	3	3	3	2	3	3	3	4	3	3	4	2	4	3	3	3	3	3
chr9:41096001	chr9	41,096,001	41,097,000	5	4	4	3	3	3	2	3	3	3	4	3	3	4	2	4	3	3	3	3	3
chr9:41097001	chr9	41,097,001	41,098,000	5	4	4	3	3	3	2	3	3	3	4	3	3	4	2	4	3	3	3	3	3
chr9:63864001	chr9	63,864,001	63,865,000	4	4	4	3	3	3	3	3	3	3	4	3	3	3	3	3	3	3	4	3	3
chr9:63865001	chr9	63,865,001	63,866,000	4	4	4	3	3	3	3	3	3	3	4	3	3	3	3	3	3	3	4	3	3
chr9:63866001	chr9	63,866,001	63,867,000	4	4	4	3	3	3	3	3	3	3	4	3	3	3	3	3	3	3	4	3	3
chr9:63867001	chr9	63,867,001	63,868,000	4	4	4	3	3	3	3	3	3	3	4	3	3	3	3	3	3	3	4	3	3
chr9:63869001	chr9	63,869,001	63,870,000	4	4	4	3	3	3	3	3	3	3	4	4	3	3	3	3	3	3	3	3	3
chr9:63870001	chr9	63,870,001	63,871,000	4	4	4	3	3	3	3	4	3	3	4	4	3	3	3	3	3	3	3	3	3
chr9:63874001	chr9	63,874,001	63,875,000	4	4	4	3	3	3	3	3	3	3	3	4	3	3	3	4	3	3	3	3	3
chr9:66804001	chr9	66,804,001	66,805,000	5	4	3	2	3	3	2	3	2	3	4	3	3	3	3	3	3	4	3	3	3
chr9:66805001	chr9	66,805,001	66,806,000	5	4	3	2	3	3	2	3	2	3	4	3	3	3	3	3	3	4	3	3	3
chr9:66811001	chr9	66,811,001	66,812,000	5	4	3	2	3	3	2	3	2	3	4	3	4	3	3	3	3	3	3	3	3
chr9:66812001	chr9	66,812,001	66,813,000	5	4	3	2	3	3	2	3	2	3	4	3	4	3	3	3	3	3	3	3	3
chr9:66813001	chr9	66,813,001	66,814,000	5	4	3	2	3	3	2	3	2	3	4	3	4	3	3	3	3	3	3	3	3
chr16:32833001	chr16	32,833,001	32,834,000	3	3	3	3	2	2	2	2	2	3	3	3	3	3	2	3	3	4	4	4	3
chr16:32834001	chr16	32,834,001	32,835,000	3	3	3	3	2	2	2	2	2	3	3	3	3	3	2	3	3	4	4	4	3
chr16:32835001	chr16	32,835,001	32,836,000	3	3	3	3	2	2	2	2	2	3	3	3	3	3	2	3	3	4	4	4	3
chr16:32836001	chr16	32,836,001	32,837,000	3	3	3	3	2	2	2	2	2	3	3	3	3	3	2	3	3	4	4	4	3
chr16:32837001	chr16	32,837,001	32,838,000	3	3	3	3	2	2	2	2	2	3	3	3	3	3	2	3	3	4	4	4	3
chr16:32838001	chr16	32,838,001	32,839,000	3	3	3	3	2	2	2	2	2	3	3	3	3	3	2	3	3	4	4	4	3
chr16:32839001	chr16	32,839,001	32,840,000	3	3	3	3	2	2	2	2	2	3	3	3	3	3	2	3	3	4	4	4	3
chr16:32840001	chr16	32,840,001	32,841,000	2	3	3	3	2	2	2	2	2	3	3	3	3	3	2	3	3	4	4	4	3
chr6:383001	chr6	383,001	384,000	2	2	2	3	4	4	4	3	3	3	2	3	3	3	4	3	3	3	4	4	3
chr1:121747001	chr1	121,747,001	121,748,000	5	5	5	3	4	2	2	3	2	4	4	3	3	3	2	3	3	4	3	3	2
chr2:132361001	chr2	132,361,001	132,362,000	5	5	5	2	3	2	2	2	3	3	4	3	2	2	2	4	3	3	4	3	4
chr2:132362001	chr2	132,362,001	132,363,000	5	5	5	2	3	2	2	2	3	3	4	3	2	2	2	4	3	3	4	3	4
chr9:39031001	chr9	39,031,001	39,032,000	5	5	5	3	3	3	2	3	3	3	4	3	3	3	3	4	3	3	3	2	3
chr9:39032001	chr9	39,032,001	39,033,000	5	5	5	3	3	3	2	3	3	3	4	3	3	3	3	4	3	3	3	2	3
chr9:60664001	chr9	60,664,001	60,665,000	5	4	5	4	3	3	2	3	3	3	3	3	3	2	3	5	3	3	3	2	4
chr9:60673001	chr9	60,673,001	60,674,000	5	4	5	4	3	3	2	3	3	3	3	5	3	3	3	3	2	3	4	3	2
chr9:63831001	chr9	63,831,001	63,832,000	5	5	5	3	3	2	3	3	3	5	3	3	3	3	2	3	3	3	3	3	3
chr2:94201001	chr2	94,201,001	94,202,000	4	4	4	3	3	3	3	3	3	3	4	3	3	3	3	3	3	3	3	3	3
chr2:94202001	chr2	94,202,001	94,203,000	4	4	4	3	3	3	3	3	3	3	4	3	3	3	3	3	3	3	3	3	3
chr2:94203001	chr2	94,203,001	94,204,000	4	4	4	3	3	3	3	3	3	3	4	3	3	3	3	3	3	3	3	3	3
chr2:94204001	chr2	94,204,001	94,205,000	4	4	4	3	3	3	3	3	3	3	4	3	3	3	3	3	3	3	3	3	3
chr3:90689001	chr3	90,689,001	90,690,000	4	4	4	3	3	3	2	3	3	4	3	2	3	3	3	4	3	3	3	3	3
chr3:90690001	chr3	90,690,001	90,691,000	4	4	4	3	3	3	2	3	3	4	3	2	3	3	3	4	3	3	3	3	3
chr3:90691001	chr3	90,691,001	90,692,000	4	4	4	3	3	3	2	3	3	4	3	2	3	3	3	4	3	3	3	3	3
chr3:90692001	chr3	90,692,001	90,693,000	4	4	4	3	3	3	2	3	3	4	3	2	3	3	3	4	3	3	3	3	3
chr3:90693001	chr3	90,693,001	90,694,000	4	4	4	3	3	3	2	3	3	4	3	2	3	3	3	4	3	3	3	3	3
chr3:90694001	chr3	90,694,001	90,695,000	4	4	4	3	3	3	2	3	3	4	3	2	3	3	3	4	3	3	3	3	3
chr3:90695001	chr3	90,695,001	90,696,000	4	4	4	3	3	3	2	3	3	4	3	2	3	3	3	4	3	3	3	3	3
chr3:90696001	chr3	90,696,001	90,697,000	4	4	4	3	3	3	2	3	3	4	3	2	3	3	3	4	3	3	3	3	3
chr3:90697001	chr3	90,697,001	90,698,000	4	4	4	3	3	3	2	3	3	4	3	2	3	3	3	4	3	3	3	3	3
chr9:38974001	chr9	38,974,001	38,975,000	5	4	4	3	3	4	2	3	3	3	3	3	3	3	3	4	3	3	3	3	3
chr9:38975001	chr9	38,975,001	38,976,000	5	4	4	4	3	4	2	3	3	3	3	3	3	3	3	4	3	3	3	3	3
chr9:41098001	chr9	41,098,001	41,099,000	5	4	4	3	3	3	2	3	3	3	4	3	3	3	2	4	3	3	3	3	3
chr9:41099001	chr9	41,099,001	41,100,000	4	4	4	3	3	3	2	3	3	3	4	3	3	3	2	4	3	3	3	3	3
chr9:41100001	chr9	41,100,001	41,101,000	4	4	4	3	3	3	2	3	3	3	4	3	3	3	2	4	3	3	3	3	3
chr9:41101001	chr9	41,101,001	41,102,000	4	4	4	3	3	3	2	3	3	3	4	3	3	3	2	4	3	3	3	3	3
chr9:41102001	chr9	41,102,001	41,103,000	4	4	4	3	3	3	2	3	3	3	4	3	3	3	2	4	3	3	3	3	3
chr9:41103001	chr9	41,103,001	41,104,000	4	4	4	3	3	3	2	3	3	3	4	3	3	3	2	4	3	3	3	3	3
chr9:41104001	chr9	41,104,001	41,105,000	4	4	4	3	3	3	2	3	3	3	4	3	3	3	2	4	3	3	3	3	3
chr9:41105001	chr9	41,105,001	41,106,000	4	4	4	3	3	3	2	3	3	3	4	3	3	3	2	4	3	3	3	3	3
chr9:41106001	chr9	41,106,001	41,107,000	4	4	4	3	3	3	2	3	3	3	4	3	3	3	2	4	3	3	3	3	3
chr9:41107001	chr9	41,107,001	41,108,000	4	4	4	3	3	3	2	3	3	3	4	3	3	3	2	4	3	3	3	3	3
chr9:41108001	chr9	41,108,001	41,109,000	4	4	4	3	3	3	2	3	3	3	4	3	3	3	2	4	3	3	3	3	3
chr9:41109001	chr9	41,109,001	41,110,000	4	4	4	3	3	3	2	3	3	3	4	3	3	3	2	4	3	3	3	3	3
chr9:63871001	chr9	63,871,001	63,872,000	4	4	4	3	3	3	3	4	3	3	3	4	3	3	3	3	3	3	3	3	3
chr9:63872001	chr9	63,872,001	63,873,000	4	4	4	3	3	3	3	4	3	3	3	4	3	3	3	3	3	3	3	3	3
chr9:63873001	chr9	63,873,001	63,874,000	4	4	4	3	3	3	3	4	3	3	3	4	3	3	3	3	3	3	3	3	3
chr9:63897001	chr9	63,897,001	63,898,000	4	4	4	3	3	3	2	3	3	3	4	3	3	3	3	3	2	4	3	3	3
chr9:63898001	chr9	63,898,001	63,899,000	4	4	4	3	3	3	2	3	3	3	4	3	3	3	3	3	2	4	3	3	3
chr9:65966001	chr9	65,966,001	65,967,000	5	4	4	4	3	3	2	3	3	3	3	3	3	4	2	3	4	3	3	3	3
chr9:65967001	chr9	65,967,001	65,968,000	5	4	4	4	3	3	2	3	3	3	3	3	3	4	2	3	4	3	3	3	3
chr17:21847001	chr17	21,847,001	21,848,000	3	4	4	3	2	2	2	2	2	3	3	3	3	3	2	5	3	3	3	3	3
chr9:66806001	chr9	66,806,001	66,807,000	5	4	3	2	3	3	2	3	2	3	4	3	3	3	3	3	3	3	3	3	3
chr9:66807001	chr9	66,807,001	66,808,000	5	4	3	2	3	3	2	3	2	3	4	3	3	3	3	3	3	3	3	3	3
chr9:66808001	chr9	66,808,001	66,809,000	5	4	3	2	3	3	2	3	2	3	4	3	3	3	3	3	3	3	3	3	3
chr9:66809001	chr9	66,809,001	66,810,000	5	4	3	2	3	3	2	3	2	3	4	3	3	3	3	3	3	3	3	3	3
chr9:66810001	chr9	66,810,001	66,811,000	5	4	3	2	3	3	2	3	2	3	4	3	3	3	3	3	3	3	3	3	3
chr1:138001	chr1	138,001	139,000	2	4	2	4	4	5	4	4	5	3	2	4	3	3	4	3	3	2	3	4	3
chr1:200001	chr1	200,001	201,000	3	3	2	3	4	4	4	4	4	4	3	3	3	3	3	3	4	3	2	3	3
chr1:13224001	chr1	13,224,001	13,225,000	2	1	2	3	4	4	4	3	4	2	3	3	4	2	4	2	4	3	4	4	2
chr22:11431001	chr22	11,431,001	11,432,000	1	1	2	3	3	2	3	2	2	3	3	2	3	2	3	4	4	4	3	3	3
chr22:11432001	chr22	11,432,001	11,433,000	1	2	2	3	3	2	3	2	2	3	3	2	3	2	3	4	4	4	3	3	3
chr22:11433001	chr22	11,433,001	11,434,000	1	2	2	3	3	2	3	2	2	3	3	2	3	2	3	4	4	4	3	3	3
chr22:11434001	chr22	11,434,001	11,435,000	1	2	2	3	3	2	3	2	2	3	3	2	3	2	3	4	4	4	3	3	3
chr22:11435001	chr22	11,435,001	11,436,000	1	2	2	3	3	2	3	2	2	3	3	2	3	2	3	4	4	4	3	3	3
chr2:94569001	chr2	94,569,001	94,570,000	4	5	5	3	3	2	2	3	3	4	3	3	3	3	2	2	3	3	3	3	4
chr2:132360001	chr2	132,360,001	132,361,000	5	5	5	2	3	2	2	2	3	3	4	3	2	2	2	4	3	3	4	2	4

Region	CHR	start	end	HD2	HD3	HD1	BN1	BN2	BN3	BN4	BN5	BN6	LC1	LC2	LC3	LC4	LC5	LC6	LC7	LC8	LC9	LC10	LC10*	LC11
chr2:132363001	chr2	132,363,001	132,364,000	5	5	5	2	3	2	2	2	3	3	3	3	2	2	2	4	3	3	4	3	4
chr5:34197001	chr5	34,197,001	34,198,000	5	5	5	4	2	3	2	3	2	4	4	3	2	4	3	4	4	2	2	2	2
chr9:38994001	chr9	38,994,001	38,995,000	5	4	5	4	3	3	2	3	3	3	2	3	3	3	3	4	3	3	3	3	3
chr9:39044001	chr9	39,044,001	39,045,000	3	5	5	3	2	3	2	3	3	3	4	4	3	3	3	3	3	3	2	2	3
chr9:39045001	chr9	39,045,001	39,046,000	3	5	5	3	2	3	2	3	3	3	4	4	3	3	3	3	3	3	2	2	3
chr9:39046001	chr9	39,046,001	39,047,000	3	5	5	3	2	3	2	3	3	3	4	4	3	3	3	3	3	3	2	2	3
chr9:60674001	chr9	60,674,001	60,675,000	5	4	5	4	3	2	2	3	3	3	3	5	3	3	2	3	2	3	4	3	2
chr16:34068001	chr16	34,068,001	34,069,000	5	5	5	4	3	3	4	3	3	3	3	3	3	4	3	1	2	5	3	3	3
chr16:34069001	chr16	34,069,001	34,070,000	5	5	5	4	3	2	4	3	3	3	3	3	3	4	3	1	2	5	3	3	3
chr2:89628001	chr2	89,628,001	89,629,000	3	4	4	3	3	3	3	3	3	3	3	3	3	3	3	3	3	3	3	3	3
chr2:89629001	chr2	89,629,001	89,630,000	3	4	4	3	3	3	3	3	3	3	3	3	3	3	3	3	3	3	3	3	3
chr2:89630001	chr2	89,630,001	89,631,000	3	4	4	3	3	3	3	3	3	3	3	3	3	3	3	3	3	3	3	3	3
chr2:89631001	chr2	89,631,001	89,632,000	3	4	4	3	3	3	3	3	3	3	3	3	3	3	3	3	3	3	3	3	3
chr2:89632001	chr2	89,632,001	89,633,000	3	4	4	3	3	3	3	3	3	3	3	3	3	3	3	3	3	3	3	3	3
chr2:89633001	chr2	89,633,001	89,634,000	3	4	4	3	3	3	3	3	3	3	3	3	3	3	3	3	3	3	3	3	3
chr2:89634001	chr2	89,634,001	89,635,000	3	4	4	3	3	3	3	3	3	3	3	3	3	3	3	3	3	3	3	3	3
chr2:89635001	chr2	89,635,001	89,636,000	3	4	4	3	3	3	3	3	3	3	3	3	3	3	3	3	3	3	3	3	3
chr2:89636001	chr2	89,636,001	89,637,000	3	4	4	3	3	3	3	3	3	3	3	3	3	3	3	3	3	3	3	3	3
chr2:89637001	chr2	89,637,001	89,638,000	3	4	4	3	3	3	3	3	3	3	3	3	3	3	3	3	3	3	3	3	3
chr2:89638001	chr2	89,638,001	89,639,000	3	4	4	3	3	3	3	3	3	3	3	3	3	3	3	3	3	3	3	3	3
chr2:94189001	chr2	94,189,001	94,190,000	3	4	4	3	3	2	2	2	3	3	3	3	3	3	3	3	3	3	3	3	3
chr2:94190001	chr2	94,190,001	94,191,000	3	4	4	3	3	2	2	2	3	3	3	3	3	3	3	3	3	3	3	3	3
chr2:94191001	chr2	94,191,001	94,192,000	3	4	4	3	3	2	2	2	3	3	3	3	3	3	3	3	3	3	3	3	3
chr2:94192001	chr2	94,192,001	94,193,000	3	4	4	3	3	2	2	2	3	3	3	3	3	3	3	3	3	3	3	3	3
chr2:94193001	chr2	94,193,001	94,194,000	3	4	4	3	3	2	2	2	3	3	3	3	3	3	3	3	3	3	3	3	3
chr2:94194001	chr2	94,194,001	94,195,000	3	4	4	3	3	3	2	3	3	3	3	3	3	3	3	3	3	3	3	3	3
chr2:94195001	chr2	94,195,001	94,196,000	3	4	4	3	3	3	2	3	3	3	3	3	3	3	3	3	3	3	3	3	3
chr2:94196001	chr2	94,196,001	94,197,000	3	4	4	3	3	3	3	3	3	3	3	3	3	3	3	3	3	3	3	3	3
chr2:94197001	chr2	94,197,001	94,198,000	3	4	4	3	3	3	3	3	3	3	3	3	3	3	3	3	3	3	3	3	3
chr2:94198001	chr2	94,198,001	94,199,000	3	4	4	3	3	3	3	3	3	3	3	3	3	3	3	3	3	3	3	3	3
chr2:94199001	chr2	94,199,001	94,200,000	3	4	4	3	3	3	3	3	3	3	3	3	3	3	3	3	3	3	3	3	3
chr2:94200001	chr2	94,200,001	94,201,000	3	4	4	3	3	3	3	3	3	3	3	3	3	3	3	3	3	3	3	3	3
chr2:94557001	chr2	94,557,001	94,558,000	4	4	4	3	3	2	2	3	3	4	4	3	3	3	2	3	3	3	3	2	3
chr2:94558001	chr2	94,558,001	94,559,000	4	4	4	3	3	2	2	3	3	4	4	3	3	3	2	3	3	3	3	2	3
chr3:90687001	chr3	90,687,001	90,688,000	5	4	4	3	3	3	2	3	3	4	1	3	3	3	3	4	3	3	3	3	3
chr9:41110001	chr9	41,110,001	41,111,000	4	4	4	3	3	3	2	2	3	3	4	3	3	3	2	3	3	3	3	3	3
chr9:63855001	chr9	63,855,001	63,856,000	4	3	4	3	3	2	2	3	3	3	3	3	3	3	3	3	3	3	3	3	3
chr9:63856001	chr9	63,856,001	63,857,000	4	3	4	3	3	2	2	3	3	3	3	3	3	3	3	3	3	3	3	3	3
chr9:63857001	chr9	63,857,001	63,858,000	4	3	4	3	3	2	2	3	3	3	3	3	3	3	3	3	3	3	3	3	3
chr9:63858001	chr9	63,858,001	63,859,000	4	3	4	3	3	3	2	3	3	3	3	3	3	3	3	3	3	3	3	3	3
chr9:63859001	chr9	63,859,001	63,860,000	4	3	4	3	3	3	2	3	3	3	3	3	3	3	3	3	3	3	3	3	3
chr9:63860001	chr9	63,860,001	63,861,000	4	3	4	3	3	3	2	3	3	3	3	3	3	3	3	3	3	3	3	3	3
chr9:66211001	chr9	66,211,001	66,212,000	5	4	4	4	4	4	2	3	3	3	3	3	3	3	2	4	3	3	3	3	3
chr9:66212001	chr9	66,212,001	66,213,000	5	4	4	4	4	4	2	3	3	3	3	3	3	3	2	4	3	3	3	3	3
chr10:42145001	chr10	42,145,001	42,146,000	3	4	4	3	3	2	2	2	3	3	3	3	3	3	2	3	3	3	4	3	3
chr10:42146001	chr10	42,146,001	42,147,000	3	4	4	3	3	2	2	2	3	3	3	3	3	3	2	3	3	3	4	3	3
chr10:42152001	chr10	42,152,001	42,153,000	4	4	4	3	3	2	2	2	3	3	3	3	3	3	2	3	3	3	4	3	3
chr10:42153001	chr10	42,153,001	42,154,000	4	4	4	3	3	2	2	2	3	3	3	3	3	3	2	3	3	3	4	3	3
chr10:42154001	chr10	42,154,001	42,155,000	4	4	4	3	3	2	2	2	3	3	3	3	3	3	2	3	3	3	4	3	3
chr10:42155001	chr10	42,155,001	42,156,000	4	4	4	3	3	2	2	2	3	3	3	3	3	3	2	3	3	3	4	3	3
chr10:42156001	chr10	42,156,001	42,157,000	4	4	4	3	3	2	2	2	3	3	3	3	3	3	2	3	3	3	4	3	3
chr10:42157001	chr10	42,157,001	42,158,000	4	4	4	3	3	2	2	2	3	3	3	3	3	3	2	3	3	3	4	3	3
chr10:42158001	chr10	42,158,001	42,159,000	4	4	4	3	3	2	2	2	3	3	3	3	3	3	2	3	3	3	4	3	3
chr10:42159001	chr10	42,159,001	42,160,000	4	4	4	4	3	2	2	2	3	3	3	3	3	3	2	3	3	3	4	3	3
chr10:42160001	chr10	42,160,001	42,161,000	4	4	4	4	3	2	2	2	3	3	3	3	3	3	2	3	3	3	4	3	3
chr10:42168001	chr10	42,168,001	42,169,000	4	4	4	4	3	2	2	2	3	3	3	4	3	3	2	3	3	3	3	3	3
chr12:37732001	chr12	37,732,001	37,733,000	4	5	4	3	3	2	2	2	3	3	4	3	3	3	2	3	3	4	2	3	3
chr1:144118001	chr1	144,118,001	144,119,000	3	3	3	2	3	3	2	2	3	3	3	3	3	3	3	3	3	3	3	3	3
chr1:144119001	chr1	144,119,001	144,120,000	3	3	3	2	3	3	2	2	3	3	3	3	3	3	3	3	3	3	3	3	3
chr1:144120001	chr1	144,120,001	144,121,000	3	3	3	2	3	3	2	2	3	3	3	3	3	3	3	3	3	3	3	3	3
chr1:144121001	chr1	144,121,001	144,122,000	3	3	3	2	3	3	2	2	3	3	3	3	3	3	3	3	3	3	3	3	3
chr1:144122001	chr1	144,122,001	144,123,000	3	3	3	2	3	3	2	2	3	3	3	3	3	3	3	3	3	3	3	3	3
chr9:66802001	chr9	66,802,001	66,803,000	5	4	3	2	3	3	2	3	2	3	4	3	3	3	3	3	3	3	3	2	3
chr9:66803001	chr9	66,803,001	66,804,000	5	4	3	2	3	3	2	3	2	3	4	3	3	3	3	3	3	3	3	2	3
chr1:201001	chr1	201,001	202,000	3	3	2	3	4	4	4	4	4	3	3	3	3	3	3	3	4	3	2	3	3
chr22:11428001	chr22	11,428,001	11,429,000	0	1	2	3	3	2	3	2	2	3	3	2	3	2	3	4	4	4	3	3	2
chr22:11429001	chr22	11,429,001	11,430,000	1	1	2	3	3	2	3	2	2	3	3	2	3	2	3	4	4	4	3	3	2
chr22:11430001	chr22	11,430,001	11,431,000	1	1	2	3	3	2	3	2	2	3	3	2	3	2	3	4	4	4	3	3	2
chr22:11440001	chr22	11,440,001	11,441,000	1	2	2	3	3	2	3	2	2	3	3	2	3	3	3	3	3	4	3	3	3
chr22:11441001	chr22	11,441,001	11,442,000	1	2	2	3	3	2	3	2	2	3	3	2	3	3	3	3	3	4	3	3	3
chr22:11442001	chr22	11,442,001	11,443,000	1	2	2	3	3	2	3	2	2	3	3	2	3	3	3	3	3	4	3	3	3
chr2:95857001	chr2	95,857,001	95,858,000	5	5	5	4	3	2	2	3	2	4	2	4	3	2	2	4	3	2	2	3	4
chr4:49621001	chr4	49,621,001	49,622,000	4	3	5	3	3	2	2	2	3	3	3	3	2	3	2	4	3	3	3	3	3
chr4:49622001	chr4	49,622,001	49,623,000	4	3	5	3	3	2	2	2	3	3	3	3	2	3	2	4	3	3	3	3	3
chr4:49623001	chr4	49,623,001	49,624,000	4	3	5	3	3	2	2	2	3	3	3	3	2	3	2	4	3	3	3	3	3
chr4:49624001	chr4	49,624,001	49,625,000	4	3	5	3	3	2	2	2	3	3	3	3	2	3	2	4	3	3	3	3	3
chr4:49625001	chr4	49,625,001	49,626,000	4	3	5	3	3	2	2	2	3	3	3	3	2	3	2	4	3	3	3	3	3
chr4:49626001	chr4	49,626,001	49,627,000	4	3	5	3	3	2	2	2	3	3	3	3	2	3	2	4	3	3	3	3	3
chr4:49627001	chr4	49,627,001	49,628,000	4	3	5	3	3	2	2	2	3	3	3	3	2	3	2	4	3	3	3	3	3
chr4:49628001	chr4	49,628,001	49,629,000	4	3	5	3	3	2	2	2	3	3	3	3	2	3	2	4	3	3	3	3	3
chr16:34070001	chr16	34,070,001	34,071,000	5	5	5	4	3	2	4	3	3	3	3	3	3	3	3	1	2	5	3	3	3
chr17:26780001	chr17	26,780,001	26,781,000	5	3	5	3	3	3	2	2	3	3	3	3	3	2	3	3	3	3	3	3	3
chr21:9811001	chr21	9,811,001	9,812,000	4	4	5	2	3	2	1	2	2	3	3	3	3	2	2	4	3	3	4	2	3
chr2:94556001	chr2	94,556,001	94,557,000	4	4	4	3	3	2	2	3	3	4	3	3	3	3	2	3	3	3	3	2	3
chr9:41111001	chr9	41,111,001	41,112,000	4	4	4	3	3	3	2	2	3	3	4	3	3	3	2	3	3	2	3	3	3
chr9:63854001	chr9	63,854,001	63,855,000	4	3	4	3	3	2	2	3	3	3	3	3	2	3	3	3	3	3	3	3	3
chr9:65960001	chr9	65,960,001	65,961,000	4	3	4	3	3	3	2	2	3	3	3	3	3	3	2	3	3	3	3	3	3
chr10:42148001	chr10	42,148,001	42,149,000	4	4	4	3	3	2	2	2	3	3	3	3	3	2	2	3	3	3	4	3	3
chr10:42149001	chr10	42,149,001	42,150,000	4	4	4	3	3	2	2	2	3	3	3	3	3	2	2	3	3	3	4	3	3
chr10:42150001	chr10	42,150,001	42,151,000	4	4	4	3	3	2	2	2	3	3	3	3	3	2	2	3	3	3	4	3	3
chr10:42151001	chr10	42,151,001	42,152,000	4	4	4	3	3	2	2	2	3	3	3	3	3	2	2	3	3	3	4	3	3
chr16:34085001	chr16	34,085,001	34,086,000	4	4	4	3	2	2	1	2	3	3	3	3	3	3	2	3	2	3	4	3	3
chr16:34086001	chr16	34,086,001	34,087,000	4	3	4	3	2	2	1	2	3	3	3	3	3	3	2	3	2	3	4	3	3
chr16:36115001	chr16	36,115,001	36,116,000	3	3	4	3	2	2	2	2	2	3	3	3	3	3	2	3	3	3	3	3	3
chr16:36116001	chr16	36,116,001	36,117,000	3	3	4	3	2	2	2	2	2	3	3	3	3	3	2	3	3	3	3	3	3
chr16:36117001	chr16	36,117,001	36,118,000	3	3	4	3	2	2	2	2	2	3	3	3	3	3	2	3	3	3	3	3	3
chr16:36118001	chr16	36,118,001	36,119,000	3	3	4	3	2	2	2	2	3	3	3	3	3	3	2	3	3	3	3	3	3
chr16:36119001	chr16	36,119,001	36,120,000	3	3	4	3	2	2	2	2	3	3	3	3	3	3	2	3	3	3	3	3	3
chr16:36120001	chr16	36,120,001	36,121,000	3	3	4	3	2	2	2	2	3	3	3	3	3	3	2	3	3	3	3	3	3
chr16:36121001	chr16	36,121,001	36,122,000	3	3	4	3	2	2	2	2	3	3	3	3	3	3	2	3	3	3	3	3	3
chr16:36122001	chr16	36,122,001	36,123,000	3	3	4	3	2	2	2	2	3	3	3	3	3	3	2	3	3	3	3	3	3
chr17:26943001	chr17	26,943,001	26,944,000	3	4	4	3	2	2	2	2	3	3	3	3	3	3	2	3	3	3	3	3	3
chr3:90650001	chr3	90,650,001	90,651,000	4	4	3	3	3	2	2	3	3	3	3	3	3	3	2	3	3	3	3	3	3
chr3:90651001	chr3	90,651,001	90,652,000	4	4	3	3	3	2	2	3	3	3	3	3	3	3	2	3	3	3	3	3	3
chr3:90652001	chr3	90,652,001	90,653,000	4	4	3	3	3	2	2	3	3	3	3	3	3	3	2	3	3	3	3	3	3
chr3:90653001	chr3	90,653,001	90,654,000	4	4	3	3	3	2	2	3	3	3	3	3	3	3	2	3	3	3	3	3	3
chr3:90654001	chr3	90,654,001	90,655,000	4	4	3	3	3	2	2	3	3	3	3	3	3	3	2	3	3	3	3	3	3
chr3:90655001	chr3	90,655,001	90,656,000	4	4	3	3	3	2	2	3	3	3	3	3	3	3	2	3	3	3	3	3	3
chr3:90656001	chr3	90,656,001	90,657,000	4	4	3	3	3	2	2	3	3	3	3	3	3	3	2	3	3	3	3	3	3
chr3:90657001	chr3	90,657,001	90,658,000	4	4	3	3	3	2	2	3	3	3	3	3	3	3	2	3	3	3	3	3	3
chr3:90658001	chr3	90,658,001	90,659,000	4	4	3	3	3	2	2	3	3	3	3	3	3	3	2	3	3	3	3	3	3
chr3:90659001	chr3	90,659,001	90,660,000	4	4	3	3	3	2	2	3	3	3	3	3	3	3	2	3	3	3	3	3	3
chr3:90660001	chr3	90,660,001	90,661,000	4	4	3	3	3	2	2	3	3	3	3	3	3	3	2	3	3	3	3	3	3

Region	CHR	start	end	HD2	HD3	HD1	BN1	BN2	BN3	BN4	BN5	BN6	LC1	LC2	LC3	LC4	LC5	LC6	LC7	LC8	LC9	LC10	LC10*	LC11
chr3:90661001	chr3	90,661,001	90,662,000	4	4	3	3	3	2	2	3	3	3	3	3	3	3	2	3	3	3	3	3	3
chr3:90662001	chr3	90,662,001	90,663,000	4	4	3	3	3	2	2	3	3	3	3	3	3	3	2	3	3	3	3	3	3
chr3:90663001	chr3	90,663,001	90,664,000	4	4	3	3	3	2	2	3	3	3	3	3	3	3	2	3	3	3	3	3	3
chr7:62269001	chr7	62,269,001	62,270,000	3	3	3	3	2	2	2	2	2	3	3	3	3	3	2	3	3	3	3	3	3
chr7:62270001	chr7	62,270,001	62,271,000	3	3	3	3	2	2	2	2	3	3	3	3	3	3	2	3	3	3	3	3	3
chr7:62271001	chr7	62,271,001	62,272,000	3	3	3	3	2	2	2	2	3	3	3	3	3	3	2	3	3	3	3	3	3
chr7:62272001	chr7	62,272,001	62,273,000	3	3	3	3	2	2	2	2	3	3	3	3	3	3	2	3	3	3	3	3	3
chr7:62273001	chr7	62,273,001	62,274,000	3	3	3	3	2	2	2	2	3	3	3	3	3	3	2	3	3	3	3	3	3
chr7:62274001	chr7	62,274,001	62,275,000	3	3	3	3	2	2	2	2	2	3	3	3	3	3	2	3	3	3	3	3	3
chr7:62275001	chr7	62,275,001	62,276,000	3	3	3	3	2	3	2	2	2	3	3	3	3	3	2	3	3	3	3	3	3
chr7:62276001	chr7	62,276,001	62,277,000	3	3	3	3	2	3	2	2	2	3	3	3	3	3	2	3	3	3	3	3	3
chr7:62277001	chr7	62,277,001	62,278,000	3	3	3	3	2	3	2	2	2	3	3	3	3	3	2	3	3	3	3	3	3
chr7:62278001	chr7	62,278,001	62,279,000	3	3	3	3	2	3	2	2	2	3	3	3	3	3	2	3	3	3	3	3	3
chr7:62279001	chr7	62,279,001	62,280,000	3	3	3	3	2	3	2	2	2	3	3	3	3	3	2	3	3	3	3	3	3
chr9:66008001	chr9	66,008,001	66,009,000	4	4	3	3	3	3	2	3	2	3	3	3	3	3	2	3	3	3	3	3	3
chr9:66009001	chr9	66,009,001	66,010,000	4	4	3	3	3	3	2	3	2	3	3	3	3	3	2	3	3	3	3	3	3
chr9:66010001	chr9	66,010,001	66,011,000	4	4	3	3	3	3	2	3	2	3	3	3	3	3	2	3	3	3	3	3	3
chr16:32091001	chr16	32,091,001	32,092,000	3	3	3	3	2	2	2	2	2	3	3	3	3	3	2	3	3	3	3	3	3
chr16:32092001	chr16	32,092,001	32,093,000	3	3	3	3	2	2	2	2	2	3	3	3	3	3	2	3	3	3	3	3	3
chr16:32093001	chr16	32,093,001	32,094,000	3	3	3	3	2	2	2	2	2	3	3	3	3	3	2	3	3	3	3	3	3
chr16:32094001	chr16	32,094,001	32,095,000	3	3	3	3	2	2	2	2	2	3	3	3	3	3	2	3	3	3	3	3	3
chr16:32095001	chr16	32,095,001	32,096,000	3	3	3	3	2	2	2	2	2	3	3	3	3	3	2	3	3	3	3	3	3
chr16:32096001	chr16	32,096,001	32,097,000	3	3	3	3	2	2	2	2	2	3	3	3	3	3	2	3	3	3	3	3	3
chr16:32097001	chr16	32,097,001	32,098,000	3	3	3	3	2	2	2	2	2	3	3	3	3	3	2	3	3	3	3	3	3
chr16:32098001	chr16	32,098,001	32,099,000	3	3	3	3	2	2	2	2	2	3	3	3	3	3	2	3	3	3	3	3	3
chr16:32099001	chr16	32,099,001	32,100,000	3	3	3	3	2	2	2	2	3	3	3	3	3	3	2	3	3	3	3	3	3
chr16:32100001	chr16	32,100,001	32,101,000	3	3	3	3	2	2	2	2	3	3	3	3	3	3	2	3	3	3	3	3	3
chr16:32101001	chr16	32,101,001	32,102,000	3	3	3	3	2	2	2	2	3	3	3	3	3	3	2	3	3	3	3	3	3
chr16:32102001	chr16	32,102,001	32,103,000	3	3	3	3	2	2	2	2	3	3	3	3	3	3	2	3	3	3	3	3	3
chr16:32103001	chr16	32,103,001	32,104,000	3	3	3	3	2	2	2	2	3	3	3	3	3	3	2	3	3	3	3	3	3
chr16:32104001	chr16	32,104,001	32,105,000	3	3	3	3	2	2	2	2	3	3	3	3	3	3	2	3	3	3	3	3	3
chr16:32105001	chr16	32,105,001	32,106,000	3	3	3	3	2	2	2	2	3	3	3	3	3	3	2	3	3	3	3	3	3
chr16:32106001	chr16	32,106,001	32,107,000	3	3	3	3	2	2	2	2	3	3	3	3	3	3	2	3	3	3	3	3	3
chr16:32107001	chr16	32,107,001	32,108,000	3	3	3	3	2	2	2	2	3	3	3	3	3	3	2	3	3	3	3	3	3
chr16:32108001	chr16	32,108,001	32,109,000	3	3	3	3	2	2	2	2	3	3	3	3	3	3	2	3	3	3	3	3	3
chr16:32109001	chr16	32,109,001	32,110,000	3	3	3	3	2	2	2	2	3	3	3	3	3	3	2	3	3	3	3	3	3
chr16:32110001	chr16	32,110,001	32,111,000	3	3	3	3	2	2	2	2	3	3	3	3	3	3	2	3	3	3	3	3	3
chr4:190029001	chr4	190,029,001	190,030,000	2	2	2	3	3	3	3	3	3	2	3	3	3	3	3	3	3	3	3	3	3
chr7:144214001	chr7	144,214,001	144,215,000	2	2	2	3	3	3	2	3	4	3	3	2	3	3	3	3	3	3	3	3	3
chr7:144215001	chr7	144,215,001	144,216,000	2	2	2	3	3	3	2	3	4	3	3	2	3	3	3	3	3	3	3	3	3
chr7:144216001	chr7	144,216,001	144,217,000	2	2	2	3	3	3	2	3	4	3	3	2	3	3	3	3	3	3	3	3	3
chr7:144217001	chr7	144,217,001	144,218,000	2	2	2	3	3	3	2	3	4	3	3	2	3	3	3	3	3	3	3	3	3
chr22:11436001	chr22	11,436,001	11,437,000	1	2	2	3	3	2	3	2	2	3	3	2	3	2	3	3	3	4	3	3	3
chr22:11437001	chr22	11,437,001	11,438,000	1	2	2	3	3	2	3	2	2	3	3	2	3	2	3	3	3	4	3	3	3
chr22:11438001	chr22	11,438,001	11,439,000	1	2	2	3	3	2	3	2	2	3	3	2	3	2	3	3	3	4	3	3	3
chr22:11439001	chr22	11,439,001	11,440,000	1	2	2	3	3	2	3	2	2	3	3	2	3	2	3	3	3	4	3	3	3
chr22:11443001	chr22	11,443,001	11,444,000	1	2	2	3	3	2	3	2	2	3	3	2	3	3	3	3	3	3	3	3	3
chr15:101779001	chr15	101,779,001	101,780,000	2	2	1	3	3	4	4	3	3	3	2	3	3	3	4	3	3	3	2	3	3
chr15:101780001	chr15	101,780,001	101,781,000	2	2	1	3	3	4	4	3	3	3	2	3	3	3	4	3	3	3	2	3	3
chr15:101781001	chr15	101,781,001	101,782,000	2	2	1	3	3	4	4	3	3	3	2	3	3	3	4	3	3	3	2	3	3
chr2:110520001	chr2	110,520,001	110,521,000	4	5	5	2	2	2	2	2	2	2	4	2	2	2	2	2	2	4	5	5	2
chr9:41209001	chr9	41,209,001	41,210,000	3	4	5	3	3	3	2	2	3	3	3	3	3	3	2	3	3	2	3	3	3
chr12:131576001	chr12	131,576,001	131,577,000	5	5	5	2	2	1	2	2	1	1	5	5	2	5	2	2	5	3	2	1	1
chr12:131577001	chr12	131,577,001	131,578,000	5	5	5	2	2	1	2	2	1	1	5	5	2	5	2	2	5	3	2	1	1
chr16:34072001	chr16	34,072,001	34,073,000	5	5	5	3	3	2	2	2	3	3	3	3	3	3	2	3	2	3	3	3	3
chr16:34073001	chr16	34,073,001	34,074,000	5	5	5	3	3	2	2	2	3	3	3	3	3	3	2	3	2	3	3	3	3
chr9:39791001	chr9	39,791,001	39,792,000	5	4	4	3	3	3	2	3	3	3	3	3	2	3	3	3	3	2	3	3	3
chr9:39792001	chr9	39,792,001	39,793,000	4	4	4	3	3	3	2	3	3	3	3	3	2	3	3	3	3	2	3	3	3
chr9:41633001	chr9	41,633,001	41,634,000	3	3	4	2	2	2	2	2	2	3	3	3	2	3	2	4	3	3	3	3	2
chr9:63901001	chr9	63,901,001	63,902,000	3	4	4	3	3	2	2	3	2	2	3	3	3	3	3	3	2	3	3	3	3
chr16:36103001	chr16	36,103,001	36,104,000	3	3	4	3	2	2	2	2	2	3	3	3	3	3	2	3	2	3	3	3	3
chr16:36104001	chr16	36,104,001	36,105,000	3	3	4	3	2	2	2	2	2	3	3	3	3	3	2	3	2	3	3	3	3
chr16:36105001	chr16	36,105,001	36,106,000	3	3	4	3	2	2	2	2	2	3	3	3	3	3	2	3	2	3	3	3	3
chr16:36106001	chr16	36,106,001	36,107,000	3	3	4	3	2	2	2	2	2	3	3	3	3	3	2	3	2	3	3	3	3
chr16:36107001	chr16	36,107,001	36,108,000	3	3	4	3	2	2	2	2	2	3	3	3	3	3	2	3	2	3	3	3	3
chr16:36108001	chr16	36,108,001	36,109,000	3	3	4	3	2	2	2	2	2	3	3	3	3	3	2	3	2	3	3	3	3
chr16:36109001	chr16	36,109,001	36,110,000	3	3	4	3	2	2	2	2	2	3	3	3	3	3	2	3	2	3	3	3	3
chr16:36110001	chr16	36,110,001	36,111,000	3	3	4	3	2	2	2	2	2	3	3	3	3	3	2	3	2	3	3	3	3
chr16:36111001	chr16	36,111,001	36,112,000	3	3	4	3	2	2	2	2	2	3	3	3	3	3	2	3	2	3	3	3	3
chr16:36112001	chr16	36,112,001	36,113,000	3	3	4	3	2	2	2	2	2	3	3	3	3	3	2	3	2	3	3	3	3
chr16:36113001	chr16	36,113,001	36,114,000	3	3	4	3	2	2	2	2	2	3	3	3	3	3	2	3	2	3	3	3	3
chr16:36114001	chr16	36,114,001	36,115,000	3	3	4	3	2	2	2	2	2	3	3	3	3	3	2	3	2	3	3	3	3
chr16:36125001	chr16	36,125,001	36,126,000	3	3	4	2	2	2	2	2	3	3	3	3	3	2	2	3	3	3	3	3	3
chr17:26942001	chr17	26,942,001	26,943,000	3	4	4	3	2	2	2	2	3	3	3	3	3	3	2	3	2	3	3	3	3
chr17:26944001	chr17	26,944,001	26,945,000	3	4	4	3	2	2	2	2	3	3	3	3	3	2	2	3	3	3	3	3	3
chr17:26945001	chr17	26,945,001	26,946,000	3	4	4	3	2	2	2	2	3	3	3	3	3	2	2	3	3	3	3	3	3
chr17:26946001	chr17	26,946,001	26,947,000	3	4	4	3	2	2	2	2	3	3	3	3	3	2	2	3	3	3	3	3	3
chr21:9674001	chr21	9,674,001	9,675,000	4	4	4	2	2	2	2	2	3	3	2	3	3	2	3	3	3	3	3	3	3
chr21:9675001	chr21	9,675,001	9,676,000	4	4	4	2	2	2	2	2	3	3	2	3	3	2	3	3	3	3	3	3	3
chr21:9697001	chr21	9,697,001	9,698,000	4	4	4	3	2	2	2	2	3	3	2	3	3	2	3	3	3	3	3	3	3
chr21:9698001	chr21	9,698,001	9,699,000	4	4	4	3	2	2	2	2	3	3	2	3	3	2	3	3	3	3	3	3	3
chr21:9699001	chr21	9,699,001	9,700,000	4	4	4	3	2	2	2	2	3	3	2	3	3	2	3	3	3	3	3	3	3
chr21:9700001	chr21	9,700,001	9,701,000	4	4	4	3	2	2	2	2	3	3	2	3	3	2	3	3	3	3	3	3	3
chr21:9701001	chr21	9,701,001	9,702,000	4	4	4	3	2	2	2	2	3	3	2	3	3	2	3	3	3	3	3	3	3
chr21:9702001	chr21	9,702,001	9,703,000	4	4	4	3	2	2	2	2	3	3	2	3	3	2	3	3	3	3	3	3	3
chr21:9703001	chr21	9,703,001	9,704,000	4	4	4	3	2	2	2	2	3	3	2	3	3	2	3	3	3	3	3	3	3
chr21:9704001	chr21	9,704,001	9,705,000	4	4	4	3	2	2	2	2	3	3	2	3	3	2	3	3	3	3	3	3	3
chr21:9705001	chr21	9,705,001	9,706,000	4	4	4	3	2	2	2	2	3	3	2	3	3	2	3	3	3	3	3	3	3
chr21:9706001	chr21	9,706,001	9,707,000	4	4	4	3	2	2	2	2	3	3	2	3	3	2	3	3	3	3	3	3	3
chr3:90630001	chr3	90,630,001	90,631,000	4	4	3	3	2	2	2	3	3	3	3	3	3	3	2	3	2	3	3	3	3
chr3:90631001	chr3	90,631,001	90,632,000	4	4	3	3	2	2	2	3	3	3	3	3	3	3	2	3	2	3	3	3	3
chr3:90632001	chr3	90,632,001	90,633,000	4	4	3	3	2	2	2	3	3	3	3	3	3	3	2	3	2	3	3	3	3
chr3:90633001	chr3	90,633,001	90,634,000	4	4	3	3	2	2	2	3	3	3	3	3	3	3	2	3	2	3	3	3	3
chr3:197647001	chr3	197,647,001	197,648,000	3	3	3	2	2	2	2	2	3	3	3	2	2	3	3	3	3	3	3	3	3
chr3:197648001	chr3	197,648,001	197,649,000	3	3	3	2	2	2	2	2	3	3	3	2	2	3	3	3	3	3	3	3	3
chr9:40282001	chr9	40,282,001	40,283,000	5	4	3	3	3	3	2	3	2	3	3	3	3	3	2	3	3	2	3	3	3
chr9:40283001	chr9	40,283,001	40,284,000	5	4	3	3	3	3	2	3	2	3	3	3	3	3	2	3	3	2	3	3	3
chr9:40284001	chr9	40,284,001	40,285,000	5	4	3	3	3	3	2	3	2	3	3	3	3	3	2	3	3	2	3	3	3
chr9:40285001	chr9	40,285,001	40,286,000	5	4	3	3	3	3	2	3	2	3	3	3	3	3	2	3	3	2	3	3	3
chr9:40286001	chr9	40,286,001	40,287,000	5	4	3	3	3	3	2	3	2	3	3	3	3	3	2	3	3	2	3	3	3
chr9:40287001	chr9	40,287,001	40,288,000	5	4	3	3	3	3	2	3	2	3	3	3	3	3	2	3	3	2	3	3	3
chr9:40288001	chr9	40,288,001	40,289,000	5	4	3	3	3	3	2	3	2	3	3	3	3	3	2	3	3	2	3	3	3
chr9:40289001	chr9	40,289,001	40,290,000	5	4	3	3	3	3	2	3	2	3	3	3	3	3	2	3	3	2	3	3	3
chr9:40290001	chr9	40,290,001	40,291,000	5	4	3	3	3	3	2	3	2	3	3	3	3	3	2	3	3	2	3	3	3
chr16:32087001	chr16	32,087,001	32,088,000	3	3	3	3	2	2	2	2	2	3	3	3	3	3	2	3	2	3	3	3	3
chr16:32088001	chr16	32,088,001	32,089,000	3	3	3	3	2	2	2	2	2	3	3	3	3	3	2	3	2	3	3	3	3
chr16:32089001	chr16	32,089,001	32,090,000	3	3	3	3	2	2	2	2	2	3	3	3	3	3	2	3	2	3	3	3	3
chr16:32090001	chr16	32,090,001	32,091,000	3	3	3	3	2	2	2	2	2	3	3	3	3	3	2	3	2	3	3	3	3
chr16:34200001	chr16	34,200,001	34,201,000	4	4	3	3	3	2	2	2	3	3	3	3	3	3	2	3	2	3	3	3	3
chr22:21150001	chr22	21,150,001	21,151,000	3	3	3	2	2	2	2	2	2	3	3	3	3	3	3	4	3	2	2	2	3
chr22:21151001	chr22	21,151,001	21,152,000	3	3	3	2	2	2	2	2	2	3	3	3	3	3	3	4	3	2	2	2	3
chr22:21152001	chr22	21,152,001	21,153,000	3	3	3	2	2	2	2	2	2	3	3	3	3	3	3	4	3	2	2	2	3
chr22:21155001	chr22	21,155,001	21,156,000	3	3	3	2	2	2	2	2	2	3	3	3	3	3	3	4	3	2	2	2	3

Region	CHR	start	end	HD2	HD3	HD1	BN1	BN2	BN3	BN4	BN5	BN6	LC1	LC2	LC3	LC4	LC5	LC6	LC7	LC8	LC9	LC10	LC10*	LC11
chr22:21156001	chr22	21,156,001	21,157,000	3	3	3	2	2	2	2	2	2	3	3	3	3	3	3	4	3	2	2	2	3
chr4:190024001	chr4	190,024,001	190,025,000	2	2	2	3	3	3	3	3	3	2	3	2	3	3	3	3	3	3	3	3	3
chr4:190025001	chr4	190,025,001	190,026,000	2	2	2	3	3	3	3	3	3	2	3	2	3	3	3	3	3	3	3	3	3
chr4:190026001	chr4	190,026,001	190,027,000	2	2	2	3	3	3	3	3	3	2	3	2	3	3	3	3	3	3	3	3	3
chr4:190027001	chr4	190,027,001	190,028,000	2	2	2	3	3	3	3	3	3	2	3	2	3	3	3	3	3	3	3	3	3
chr4:190028001	chr4	190,028,001	190,029,000	2	2	2	3	3	3	3	3	3	2	3	2	3	3	3	3	3	3	3	3	3
chr22:11444001	chr22	11,444,001	11,445,000	1	2	2	3	3	2	3	2	2	2	3	2	3	3	3	3	3	3	3	3	3
chr22:11447001	chr22	11,447,001	11,448,000	2	2	2	3	3	2	2	2	2	2	3	2	3	3	3	3	3	3	3	3	3
chr22:11448001	chr22	11,448,001	11,449,000	2	2	2	3	3	2	2	2	2	2	3	2	3	3	3	3	3	3	3	3	3
chr22:11449001	chr22	11,449,001	11,450,000	2	2	2	3	3	2	2	2	2	2	3	2	3	3	3	3	3	3	3	3	3
chr15:101765001	chr15	101,765,001	101,766,000	2	2	1	2	4	4	4	3	3	3	2	3	3	3	4	3	3	3	2	3	2
chr15:101766001	chr15	101,766,001	101,767,000	2	2	1	2	4	4	4	3	3	3	2	3	3	3	4	3	3	3	2	3	2
chr15:101767001	chr15	101,767,001	101,768,000	2	2	1	2	4	4	4	3	3	3	2	3	3	3	4	3	3	3	2	3	2
chr15:101768001	chr15	101,768,001	101,769,000	2	2	1	2	4	4	4	3	3	3	2	3	3	3	4	3	3	3	2	3	2
chr15:101782001	chr15	101,782,001	101,783,000	2	2	1	3	3	4	4	3	3	3	2	3	3	3	4	3	3	2	2	3	3
chr15:101783001	chr15	101,783,001	101,784,000	2	2	1	3	3	4	4	3	3	3	2	3	3	3	4	3	3	2	2	3	3
chr21:13134001	chr21	13,134,001	13,135,000	2	1	1	3	3	2	3	2	2	3	2	3	3	3	2	4	3	3	3	3	2
chr21:13135001	chr21	13,135,001	13,136,000	2	1	1	3	3	2	3	2	2	3	2	3	3	3	2	4	3	3	3	3	2
chr21:13148001	chr21	13,148,001	13,149,000	2	1	1	3	3	2	3	2	2	3	2	3	3	3	2	4	3	3	3	3	2
chr21:13133001	chr21	13,133,001	13,134,000	2	1	0	3	3	2	2	2	2	3	2	3	3	3	2	4	3	3	3	3	2
chr4:189651001	chr4	189,651,001	189,652,000	5	5	5	3	2	2	2	2	3	2	2	2	2	2	2	4	4	3	4	2	4
chr4:189652001	chr4	189,652,001	189,653,000	5	5	5	3	2	2	2	2	3	2	2	2	2	2	2	4	4	3	4	2	4
chr7:56825001	chr7	56,825,001	56,826,000	4	4	5	3	4	3	2	3	3	3	4	3	3	2	3	3	2	2	3	3	2
chr7:118703001	chr7	118,703,001	118,704,000	5	5	5	2	2	2	2	2	2	2	4	2	2	2	2	2	2	4	5	4	2
chr16:34159001	chr16	34,159,001	34,160,000	4	4	5	3	3	2	2	2	3	3	3	3	3	2	2	3	2	3	3	3	3
chr16:34160001	chr16	34,160,001	34,161,000	4	4	5	3	3	2	2	2	3	3	3	3	3	2	2	3	2	3	3	3	3
chr16:34161001	chr16	34,161,001	34,162,000	4	4	5	3	3	2	2	2	3	3	3	3	3	2	2	3	2	3	3	3	3
chr16:34162001	chr16	34,162,001	34,163,000	4	4	5	3	3	2	2	2	3	3	3	3	3	2	2	3	2	3	3	3	3
chr16:34163001	chr16	34,163,001	34,164,000	4	4	5	3	3	2	2	2	3	3	3	3	3	2	2	3	2	3	3	3	3
chr16:34164001	chr16	34,164,001	34,165,000	4	4	5	3	3	2	2	2	3	3	3	3	3	2	2	3	2	3	3	3	3
chr16:34165001	chr16	34,165,001	34,166,000	4	4	5	3	3	2	2	2	3	3	3	3	3	2	2	3	2	3	3	3	3
chr16:34166001	chr16	34,166,001	34,167,000	4	4	5	3	3	2	2	2	3	3	3	3	3	2	2	3	2	3	3	3	3
chr16:34167001	chr16	34,167,001	34,168,000	4	4	5	3	3	2	2	2	3	3	3	3	3	2	2	3	2	3	3	3	3
chr16:34168001	chr16	34,168,001	34,169,000	4	4	5	3	3	2	2	2	3	3	3	3	3	2	2	3	2	3	3	3	3
chr16:34169001	chr16	34,169,001	34,170,000	4	4	5	3	3	2	2	2	3	3	3	3	3	2	2	3	2	3	3	3	3
chr16:34170001	chr16	34,170,001	34,171,000	4	4	5	3	3	2	2	2	3	3	3	3	3	2	2	3	2	3	3	3	3
chr16:34171001	chr16	34,171,001	34,172,000	4	4	5	3	3	2	2	2	3	3	3	3	3	2	2	3	2	3	3	3	3
chr16:34172001	chr16	34,172,001	34,173,000	4	4	5	3	3	2	2	2	3	3	3	3	3	2	2	3	2	3	3	3	3
chr16:34173001	chr16	34,173,001	34,174,000	4	4	5	3	3	2	2	2	3	3	3	3	3	2	2	3	2	3	3	3	3
chr21:9810001	chr21	9,810,001	9,811,000	4	4	5	2	3	2	2	2	3	3	2	3	3	2	2	4	3	3	3	2	3
chr3:195480001	chr3	195,480,001	195,481,000	5	4	4	3	3	3	2	4	3	3	3	3	4	2	3	2	3	3	2	2	3
chr9:39786001	chr9	39,786,001	39,787,000	5	3	4	3	3	3	2	3	3	3	3	3	2	3	3	3	3	2	3	3	2
chr9:39787001	chr9	39,787,001	39,788,000	5	3	4	3	3	3	2	3	3	3	3	3	2	3	3	3	3	2	3	3	2
chr9:39788001	chr9	39,788,001	39,789,000	5	4	4	3	3	3	2	3	3	3	3	3	2	3	3	3	3	2	3	3	2
chr9:39789001	chr9	39,789,001	39,790,000	5	4	4	3	3	3	2	3	3	3	3	3	2	3	3	3	3	2	3	3	2
chr9:39790001	chr9	39,790,001	39,791,000	5	4	4	3	3	3	2	3	3	3	3	3	2	3	3	3	3	2	3	3	2
chr16:34174001	chr16	34,174,001	34,175,000	4	4	4	3	3	2	2	2	3	3	3	3	3	2	2	3	2	3	3	3	3
chr16:34194001	chr16	34,194,001	34,195,000	4	4	4	3	3	2	2	2	3	3	3	3	3	2	2	3	2	3	3	3	3
chr16:34195001	chr16	34,195,001	34,196,000	4	4	4	3	3	2	2	2	3	3	3	3	3	2	2	3	2	3	3	3	3
chr16:36097001	chr16	36,097,001	36,098,000	3	3	4	3	2	2	2	2	2	3	3	3	3	2	2	3	2	3	3	3	3
chr16:36098001	chr16	36,098,001	36,099,000	3	3	4	3	2	2	2	2	2	3	3	3	3	2	2	3	2	3	3	3	3
chr16:36099001	chr16	36,099,001	36,100,000	3	3	4	3	2	2	2	2	2	3	3	3	3	2	2	3	2	3	3	3	3
chr16:36100001	chr16	36,100,001	36,101,000	3	3	4	3	2	2	2	2	2	3	3	3	3	2	2	3	2	3	3	3	3
chr16:36101001	chr16	36,101,001	36,102,000	3	3	4	3	2	2	2	2	2	3	3	3	3	2	2	3	2	3	3	3	3
chr16:36102001	chr16	36,102,001	36,103,000	3	3	4	3	2	2	2	2	2	3	3	3	3	2	2	3	2	3	3	3	3
chr17:26947001	chr17	26,947,001	26,948,000	3	4	4	3	2	2	2	2	3	3	3	3	3	2	2	3	2	3	3	3	3
chr17:26948001	chr17	26,948,001	26,949,000	3	4	4	3	2	2	2	2	3	3	3	3	3	2	2	3	2	3	3	3	3
chr17:26949001	chr17	26,949,001	26,950,000	3	4	4	3	2	2	2	2	3	3	3	3	3	2	2	3	2	3	3	3	3
chr17:26950001	chr17	26,950,001	26,951,000	3	4	4	3	2	2	2	2	3	3	3	3	3	2	2	3	2	3	3	3	3
chr17:26951001	chr17	26,951,001	26,952,000	3	4	4	3	2	2	2	2	3	3	3	3	3	2	2	3	2	3	3	3	3
chr17:26952001	chr17	26,952,001	26,953,000	3	4	4	3	2	2	2	2	3	3	3	3	3	2	2	3	2	3	3	3	3
chr17:26953001	chr17	26,953,001	26,954,000	3	4	4	3	2	2	2	2	3	3	3	3	3	2	2	3	2	3	3	3	3
chr17:26954001	chr17	26,954,001	26,955,000	3	4	4	3	2	2	2	2	3	3	3	3	3	2	2	3	2	3	3	3	3
chr17:26955001	chr17	26,955,001	26,956,000	3	4	4	3	2	2	2	2	3	3	3	3	3	2	2	3	2	3	3	3	3
chr17:26956001	chr17	26,956,001	26,957,000	3	4	4	3	2	2	2	2	3	3	3	3	3	2	2	3	2	3	3	3	3
chr17:26957001	chr17	26,957,001	26,958,000	3	4	4	3	2	2	2	2	3	3	3	3	3	2	2	3	2	3	3	3	3
chr17:26958001	chr17	26,958,001	26,959,000	3	4	4	3	2	2	2	2	3	3	3	3	3	2	2	3	2	3	3	3	3
chr17:26959001	chr17	26,959,001	26,960,000	3	4	4	3	2	2	2	2	3	3	3	3	3	2	2	3	2	3	3	3	3
chr17:26960001	chr17	26,960,001	26,961,000	3	4	4	3	2	2	2	2	3	3	3	3	3	2	2	3	2	3	3	3	3
chr21:9672001	chr21	9,672,001	9,673,000	4	4	4	2	2	2	2	2	3	3	2	3	3	2	3	3	3	3	3	2	3
chr21:9673001	chr21	9,673,001	9,674,000	4	4	4	2	2	2	2	2	3	3	2	3	3	2	3	3	3	3	3	2	3
chr21:9676001	chr21	9,676,001	9,677,000	4	4	4	2	2	2	2	2	3	3	2	3	2	2	3	3	3	3	3	3	3
chr21:9677001	chr21	9,677,001	9,678,000	4	4	4	2	2	2	2	2	3	3	2	3	2	2	3	3	3	3	3	3	3
chr21:9678001	chr21	9,678,001	9,679,000	4	4	4	2	2	2	2	2	3	3	2	3	2	2	3	3	3	3	3	3	3
chr21:9679001	chr21	9,679,001	9,680,000	4	4	4	2	2	2	2	2	3	3	2	3	2	2	3	3	3	3	3	3	3
chr21:9680001	chr21	9,680,001	9,681,000	4	4	4	2	2	2	2	2	3	3	2	3	2	2	3	3	3	3	3	3	3
chr21:9681001	chr21	9,681,001	9,682,000	4	4	4	2	2	2	2	2	3	3	2	3	2	2	3	3	3	3	3	3	3
chr21:9682001	chr21	9,682,001	9,683,000	4	4	4	2	2	2	2	2	3	3	2	3	2	2	3	3	3	3	3	3	3
chr21:9683001	chr21	9,683,001	9,684,000	4	4	4	2	2	2	2	2	3	3	2	3	2	2	3	3	3	3	3	3	3
chr21:9684001	chr21	9,684,001	9,685,000	4	4	4	2	2	2	2	2	3	3	2	3	2	2	3	3	3	3	3	3	3
chr21:9685001	chr21	9,685,001	9,686,000	4	4	4	2	2	2	2	2	3	3	2	3	2	2	3	3	3	3	3	3	3
chr21:9686001	chr21	9,686,001	9,687,000	4	4	4	2	2	2	2	2	3	3	2	3	2	2	3	3	3	3	3	3	3
chr21:9687001	chr21	9,687,001	9,688,000	4	4	4	2	2	2	2	2	3	3	2	3	2	2	3	3	3	3	3	3	3
chr21:9688001	chr21	9,688,001	9,689,000	4	4	4	3	2	2	2	2	3	3	2	3	2	2	3	3	3	3	3	3	3
chr21:9689001	chr21	9,689,001	9,690,000	4	4	4	3	2	2	2	2	3	3	2	3	2	2	3	3	3	3	3	3	3
chr21:9690001	chr21	9,690,001	9,691,000	4	4	4	3	2	2	2	2	3	3	2	3	2	2	3	3	3	3	3	3	3
chr21:9691001	chr21	9,691,001	9,692,000	4	4	4	3	2	2	2	2	3	3	2	3	2	2	3	3	3	3	3	3	3
chr21:9692001	chr21	9,692,001	9,693,000	4	4	4	3	2	2	2	2	3	3	2	3	2	2	3	3	3	3	3	3	3
chr21:9693001	chr21	9,693,001	9,694,000	4	4	4	3	2	2	2	2	3	3	2	3	2	2	3	3	3	3	3	3	3
chr21:9694001	chr21	9,694,001	9,695,000	4	4	4	3	2	2	2	2	3	3	2	3	2	2	3	3	3	3	3	3	3
chr21:9695001	chr21	9,695,001	9,696,000	4	4	4	3	2	2	2	2	3	3	2	3	2	2	3	3	3	3	3	3	3
chr21:9696001	chr21	9,696,001	9,697,000	4	4	4	3	2	2	2	2	3	3	2	3	2	2	3	3	3	3	3	3	3
chr21:9707001	chr21	9,707,001	9,708,000	4	4	4	2	2	2	2	2	3	3	2	3	3	2	2	3	3	3	3	3	3
chr21:9708001	chr21	9,708,001	9,709,000	4	4	4	2	2	2	2	2	3	3	2	3	3	2	2	3	3	3	3	3	3
chr21:9709001	chr21	9,709,001	9,710,000	4	4	4	2	2	2	2	2	3	3	2	3	3	2	2	3	3	3	3	3	3
chr21:10592001	chr21	10,592,001	10,593,000	4	3	4	2	3	2	2	2	3	2	2	3	3	2	3	3	3	3	3	3	3
chr21:10593001	chr21	10,593,001	10,594,000	4	3	4	2	3	2	2	2	3	2	2	3	3	2	3	3	3	3	3	3	3
chr21:10594001	chr21	10,594,001	10,595,000	4	3	4	2	3	2	2	2	3	2	2	3	3	2	3	3	3	3	3	3	3
chr21:10595001	chr21	10,595,001	10,596,000	4	3	4	2	3	2	2	2	3	2	2	3	3	2	3	3	3	3	3	3	3
chr21:10596001	chr21	10,596,001	10,597,000	4	3	4	2	3	2	2	2	3	2	2	3	3	2	3	3	3	3	3	3	3
chr21:10597001	chr21	10,597,001	10,598,000	4	3	4	2	3	2	2	2	3	2	2	3	3	2	3	3	3	3	3	3	3
chr21:10598001	chr21	10,598,001	10,599,000	4	3	4	2	3	2	2	2	3	2	2	3	3	2	3	3	3	3	3	3	3
chr21:10599001	chr21	10,599,001	10,600,000	4	3	4	2	3	2	2	2	3	2	2	3	3	2	3	3	3	3	3	3	3
chr21:10600001	chr21	10,600,001	10,601,000	4	3	4	2	3	2	2	2	3	2	2	3	3	2	3	3	3	3	3	3	3
chr21:10613001	chr21	10,613,001	10,614,000	4	3	4	2	3	2	2	2	3	2	2	3	3	2	3	3	3	3	3	3	3
chr21:10614001	chr21	10,614,001	10,615,000	4	3	4	2	3	2	2	2	3	2	2	3	3	2	3	3	3	3	3	3	3
chr21:10615001	chr21	10,615,001	10,616,000	4	3	4	2	3	2	2	2	3	2	2	3	3	2	3	3	3	3	3	3	3
chr21:10616001	chr21	10,616,001	10,617,000	4	3	4	2	3	2	2	2	3	2	2	3	3	2	3	3	3	3	3	3	3
chr21:10617001	chr21	10,617,001	10,618,000	4	3	4	2	3	2	2	2	3	2	2	3	3	2	3	3	3	3	3	3	3
chr21:10618001	chr21	10,618,001	10,619,000	4	3	4	2	3	2	2	2	3	2	2	3	3	2	3	3	3	3	3	3	3
chr21:10619001	chr21	10,619,001	10,620,000	4	3	4	2	3	2	2	2	3	2	2	3	3	2	3	3	3	3	3	3	3
chr21:10620001	chr21	10,620,001	10,621,000	4	3	4	2	3	2	2	2	3	2	2	3	3	2	3	3	3	3	3	3	3
chr21:10621001	chr21	10,621,001	10,622,000	4	3	4	2	3	2	2	2	3	2	2	3	3	2	3	3	3	3	3	3	3

Region	CHR	start	end	HD2	HD3	HD1	BN1	BN2	BN3	BN4	BN5	BN6	LC1	LC2	LC3	LC4	LC5	LC6	LC7	LC8	LC9	LC10	LC10*	LC11
chr21:10622001	chr21	10,622,001	10,623,000	4	3	4	2	3	2	2	2	3	2	2	3	3	2	3	3	3	3	3	3	3
chr21:10623001	chr21	10,623,001	10,624,000	4	3	4	2	3	2	2	2	3	2	2	3	3	2	3	3	3	3	3	3	3
chr21:10624001	chr21	10,624,001	10,625,000	4	3	4	2	3	2	2	2	3	2	2	3	3	2	3	3	3	3	3	3	3
chr21:10625001	chr21	10,625,001	10,626,000	4	3	4	2	3	2	2	2	3	2	2	3	3	2	3	3	3	3	3	3	3
chr21:10626001	chr21	10,626,001	10,627,000	4	3	4	2	2	2	2	2	3	2	2	3	3	2	3	3	3	3	3	3	3
chr21:10627001	chr21	10,627,001	10,628,000	4	3	4	2	2	2	2	2	3	2	2	3	3	2	3	3	3	3	3	3	3
chr9:40281001	chr9	40,281,001	40,282,000	5	4	3	3	3	3	2	3	3	3	3	3	3	3	2	3	3	2	3	3	2
chr9:40291001	chr9	40,291,001	40,292,000	5	4	3	3	3	3	2	3	2	3	3	2	3	3	2	3	3	2	3	3	3
chr9:40292001	chr9	40,292,001	40,293,000	5	4	3	3	3	3	2	3	2	3	3	2	3	3	2	3	3	2	3	3	3
chr9:40293001	chr9	40,293,001	40,294,000	5	4	3	3	3	3	2	3	2	3	3	2	3	3	2	3	3	2	3	3	3
chr9:40294001	chr9	40,294,001	40,295,000	5	4	3	3	3	3	2	3	2	3	3	2	3	3	2	3	3	2	3	3	3
chr9:67907001	chr9	67,907,001	67,908,000	5	4	3	3	3	2	2	2	2	3	3	2	3	3	2	3	3	3	3	3	2
chr9:67908001	chr9	67,908,001	67,909,000	5	4	3	3	3	2	2	2	2	3	3	2	3	3	2	3	3	3	3	3	2
chr9:67909001	chr9	67,909,001	67,910,000	5	4	3	3	3	2	2	2	2	3	3	2	3	3	2	3	3	3	3	3	2
chr16:33038001	chr16	33,038,001	33,039,000	3	3	3	2	2	2	2	2	2	3	3	3	3	3	2	3	2	3	3	3	2
chr16:34192001	chr16	34,192,001	34,193,000	4	4	3	3	3	2	2	2	2	3	3	3	3	2	2	3	2	3	3	3	3
chr16:34193001	chr16	34,193,001	34,194,000	4	4	3	3	3	2	2	2	2	3	3	3	3	2	2	3	2	3	3	3	3
chr19:39890001	chr19	39,890,001	39,891,000	3	4	3	3	2	2	2	2	2	2	3	2	3	2	3	3	3	3	3	3	3
chr19:39891001	chr19	39,891,001	39,892,000	3	4	3	3	2	2	2	2	2	2	3	2	3	2	3	3	3	3	3	3	3
chr19:39892001	chr19	39,892,001	39,893,000	3	4	3	3	2	2	2	2	2	2	3	2	3	2	3	3	3	3	3	3	3
chr19:39893001	chr19	39,893,001	39,894,000	3	4	3	3	2	2	2	2	2	2	3	2	3	2	3	3	3	3	3	3	3
chr22:21147001	chr22	21,147,001	21,148,000	3	3	3	2	2	2	2	2	2	3	3	3	3	3	3	3	3	2	2	2	3
chr22:21148001	chr22	21,148,001	21,149,000	3	3	3	2	2	2	2	2	2	3	3	3	3	3	3	3	3	2	2	2	3
chr22:21149001	chr22	21,149,001	21,150,000	3	3	3	2	2	2	2	2	2	3	3	3	3	3	3	3	3	2	2	2	3
chr22:21157001	chr22	21,157,001	21,158,000	3	3	3	2	2	2	2	2	2	3	3	3	3	3	3	4	3	2	2	2	2
chr22:21158001	chr22	21,158,001	21,159,000	3	3	3	2	2	2	2	2	2	3	3	3	3	3	3	4	3	2	2	2	2
chr22:21159001	chr22	21,159,001	21,160,000	3	3	3	2	2	2	2	2	2	3	3	3	3	3	3	4	3	2	2	2	2
chr22:21160001	chr22	21,160,001	21,161,000	3	3	3	2	2	2	2	2	2	3	3	3	3	3	3	4	3	2	2	2	2
chr22:21161001	chr22	21,161,001	21,162,000	3	3	3	2	2	2	2	2	2	3	3	3	3	3	3	4	3	2	2	2	2
chr22:21162001	chr22	21,162,001	21,163,000	3	3	3	2	2	2	2	2	2	3	3	3	3	3	3	4	3	2	2	2	2
chr22:21163001	chr22	21,163,001	21,164,000	3	3	3	2	2	2	2	2	2	3	3	3	3	3	3	4	3	2	2	2	2
chr1:719001	chr1	719,001	720,000	2	2	2	3	3	3	3	3	3	3	3	2	3	3	3	3	3	2	2	3	3
chr1:720001	chr1	720,001	721,000	2	2	2	3	3	3	3	3	3	3	3	2	3	3	3	3	3	2	2	3	3
chr1:721001	chr1	721,001	722,000	2	2	2	3	3	3	3	3	3	3	3	2	3	3	3	3	3	2	2	3	3
chr1:13241001	chr1	13,241,001	13,242,000	2	2	2	3	4	4	4	3	4	3	3	3	3	2	3	3	3	2	3	3	2
chr1:13242001	chr1	13,242,001	13,243,000	2	2	2	3	4	4	4	3	4	3	3	3	3	2	3	3	3	2	3	3	2
chr1:13243001	chr1	13,243,001	13,244,000	2	2	2	3	4	4	4	3	4	3	3	3	3	2	3	3	3	2	3	3	2
chr7:144224001	chr7	144,224,001	144,225,000	2	2	2	3	3	3	3	3	4	3	3	2	3	3	3	3	3	3	2	2	3
chr7:144225001	chr7	144,225,001	144,226,000	2	2	2	3	3	3	3	3	4	3	3	2	3	3	3	3	3	3	2	2	3
chr16:32215001	chr16	32,215,001	32,216,000	2	2	2	2	2	2	2	2	2	3	3	2	2	2	3	4	3	3	3	3	2
chr6:170645001	chr6	170,645,001	170,646,000	1	2	1	2	2	2	3	3	2	2	2	3	3	3	3	3	4	3	2	3	2
chr6:170646001	chr6	170,646,001	170,647,000	1	2	1	2	2	2	3	3	2	2	2	3	3	3	3	3	4	3	2	3	2
chr6:170647001	chr6	170,647,001	170,648,000	1	2	1	2	2	2	3	3	2	2	2	3	3	3	3	3	4	3	2	3	2
chr6:170672001	chr6	170,672,001	170,673,000	2	2	1	2	2	2	3	3	2	2	3	3	3	3	3	3	3	3	2	3	2
chr7:158333001	chr7	158,333,001	158,334,000	1	2	1	2	4	3	3	3	3	3	3	3	3	3	3	3	3	2	2	2	3
chr15:101681001	chr15	101,681,001	101,682,000	2	2	1	2	3	3	3	3	3	3	2	2	3	3	3	3	3	3	2	3	3
chr15:101684001	chr15	101,684,001	101,685,000	2	2	1	2	3	3	3	3	3	2	2	3	3	3	3	3	3	3	2	3	3
chr15:101685001	chr15	101,685,001	101,686,000	2	2	1	2	3	3	3	3	3	2	2	3	3	3	3	3	3	3	2	3	3
chr21:13074001	chr21	13,074,001	13,075,000	2	1	1	3	3	1	3	1	2	3	2	3	3	3	2	3	3	3	3	3	2
chr21:13075001	chr21	13,075,001	13,076,000	2	1	1	3	3	1	3	2	2	3	2	3	3	3	2	3	3	3	3	3	2
chr21:13076001	chr21	13,076,001	13,077,000	2	1	1	3	3	1	3	2	2	3	2	3	3	3	2	3	3	3	3	3	2
chr21:13077001	chr21	13,077,001	13,078,000	2	1	1	3	3	1	3	2	2	3	2	3	3	3	2	3	3	3	3	3	2
chr21:13078001	chr21	13,078,001	13,079,000	2	1	1	3	3	1	3	2	2	3	2	3	3	3	2	3	3	3	3	3	2
chr21:13079001	chr21	13,079,001	13,080,000	2	1	1	3	3	1	3	2	2	3	2	3	3	3	2	3	3	3	3	3	2
chr21:13080001	chr21	13,080,001	13,081,000	2	0	1	3	3	2	3	2	2	3	2	3	3	3	2	3	3	3	3	3	2
chr21:13083001	chr21	13,083,001	13,084,000	2	1	1	3	3	2	3	2	2	3	2	3	3	3	2	3	3	3	3	3	2
chr21:13084001	chr21	13,084,001	13,085,000	2	1	1	3	3	2	3	2	2	3	2	3	3	3	2	3	3	3	3	3	2
chr21:13085001	chr21	13,085,001	13,086,000	2	1	1	3	3	2	3	2	2	3	2	3	3	3	2	3	3	3	3	3	2
chr21:13086001	chr21	13,086,001	13,087,000	2	1	1	3	3	2	3	2	2	3	2	3	3	3	2	3	3	3	3	3	2
chr21:13105001	chr21	13,105,001	13,106,000	2	1	1	3	3	2	3	2	2	3	2	3	3	3	2	3	3	3	3	3	2
chr21:13106001	chr21	13,106,001	13,107,000	2	1	1	3	3	2	3	2	2	3	2	3	3	3	2	3	3	3	3	3	2
chr21:13107001	chr21	13,107,001	13,108,000	2	1	1	3	3	2	3	2	2	3	2	3	3	3	2	3	3	3	3	3	2
chr21:13108001	chr21	13,108,001	13,109,000	2	1	1	3	3	2	3	2	2	3	2	3	3	3	2	3	3	3	3	3	2
chr21:13109001	chr21	13,109,001	13,110,000	2	1	1	3	3	2	3	2	2	3	2	3	3	3	2	3	3	3	3	3	2
chr21:13110001	chr21	13,110,001	13,111,000	2	1	1	3	3	2	3	2	2	3	2	3	3	3	2	3	3	3	3	3	2
chr21:13111001	chr21	13,111,001	13,112,000	2	1	1	3	3	2	3	2	2	3	2	3	3	3	2	3	3	3	3	3	2
chr21:13112001	chr21	13,112,001	13,113,000	2	1	1	3	3	2	3	2	2	3	2	3	3	3	2	3	3	3	3	3	2
chr21:13136001	chr21	13,136,001	13,137,000	2	1	1	3	3	2	3	2	2	3	2	3	3	3	2	4	3	3	3	2	2
chr21:13137001	chr21	13,137,001	13,138,000	2	1	1	3	3	2	3	2	2	3	2	3	3	3	2	4	3	3	3	2	2
chr21:13138001	chr21	13,138,001	13,139,000	2	1	1	3	3	2	3	2	2	3	2	3	3	3	2	4	3	3	3	2	2
chr21:13139001	chr21	13,139,001	13,140,000	2	1	1	3	3	2	3	2	2	3	2	3	3	3	2	4	3	3	3	2	2
chr21:13140001	chr21	13,140,001	13,141,000	2	1	1	3	3	2	3	2	2	3	2	3	3	3	2	4	3	3	3	2	2
chr21:13141001	chr21	13,141,001	13,142,000	2	1	1	3	3	2	3	2	2	3	2	3	3	3	2	4	3	3	3	2	2
chr21:13142001	chr21	13,142,001	13,143,000	2	1	1	3	3	2	3	2	2	3	2	3	3	3	2	4	3	3	3	2	2
chr21:13143001	chr21	13,143,001	13,144,000	2	1	1	3	3	2	3	2	2	3	2	3	3	3	2	4	3	3	3	2	2
chr21:13149001	chr21	13,149,001	13,150,000	2	1	1	3	3	2	3	2	2	3	2	3	3	3	2	3	3	3	3	3	2
chr21:13150001	chr21	13,150,001	13,151,000	2	1	1	3	3	2	3	2	2	3	2	3	3	3	2	3	3	3	3	3	2
chr21:13151001	chr21	13,151,001	13,152,000	2	1	1	3	3	2	3	2	2	3	2	3	3	3	2	3	3	3	3	3	2
chr21:13155001	chr21	13,155,001	13,156,000	2	1	1	3	3	2	3	2	2	3	2	3	3	3	2	3	3	3	3	3	2
chr21:13156001	chr21	13,156,001	13,157,000	2	1	1	3	3	2	3	2	2	3	2	3	3	3	2	3	3	3	3	3	2
chr21:13157001	chr21	13,157,001	13,158,000	2	1	1	3	3	2	3	2	2	3	2	3	3	3	2	3	3	3	3	3	2
chr21:8203001	chr21	8,203,001	8,204,000	5	3	5	2	4	2	2	4	2	2	3	3	2	3	2	3	3	3	3	2	3
chr21:8204001	chr21	8,204,001	8,205,000	5	3	5	2	4	2	2	4	2	2	3	3	2	3	2	3	3	3	3	2	3
chr21:8205001	chr21	8,205,001	8,206,000	5	3	5	2	4	2	2	4	2	2	3	3	2	3	2	3	3	3	3	2	3
chr21:8206001	chr21	8,206,001	8,207,000	5	3	5	2	4	2	2	4	2	2	3	3	2	3	2	3	3	3	3	2	3
chr21:8207001	chr21	8,207,001	8,208,000	5	3	5	2	4	2	2	4	2	2	3	3	2	3	2	3	3	3	3	2	3
chr21:8208001	chr21	8,208,001	8,209,000	5	3	5	2	4	2	2	4	2	2	3	3	2	3	2	3	3	3	3	2	3
chr21:8209001	chr21	8,209,001	8,210,000	5	3	5	2	4	2	2	4	2	2	3	3	2	3	2	3	3	3	3	2	3
chr21:8210001	chr21	8,210,001	8,211,000	5	3	5	2	4	2	2	4	2	2	3	3	2	3	2	3	3	3	3	2	3
chr21:8211001	chr21	8,211,001	8,212,000	5	3	5	2	4	2	2	4	2	2	3	3	2	3	2	3	3	3	3	2	3
chr21:8212001	chr21	8,212,001	8,213,000	5	3	5	2	4	2	2	4	2	2	3	3	2	3	2	3	3	3	3	2	3
chr21:8213001	chr21	8,213,001	8,214,000	5	3	5	2	4	2	2	4	2	2	3	3	2	3	2	3	3	3	3	2	3
chr21:8214001	chr21	8,214,001	8,215,000	5	3	5	2	4	2	2	4	2	2	3	3	2	3	2	3	3	3	3	2	3
chr21:8215001	chr21	8,215,001	8,216,000	5	3	5	2	4	2	2	4	2	2	3	3	2	3	2	3	3	3	3	2	3
chr21:8216001	chr21	8,216,001	8,217,000	5	3	5	2	4	2	2	4	2	2	3	3	2	3	2	3	3	3	3	2	3
chr21:8217001	chr21	8,217,001	8,218,000	5	3	5	2	4	2	2	4	2	2	3	3	2	3	2	3	3	3	3	2	3
chr21:8218001	chr21	8,218,001	8,219,000	5	3	5	2	4	2	2	4	2	2	3	3	2	3	2	3	3	3	3	2	3
chr21:8219001	chr21	8,219,001	8,220,000	5	3	5	2	4	2	2	4	2	2	3	3	2	3	2	3	3	3	3	2	3
chr21:8220001	chr21	8,220,001	8,221,000	5	3	5	2	4	2	2	4	2	2	3	3	2	3	2	3	3	3	3	2	3
chr21:8221001	chr21	8,221,001	8,222,000	5	3	5	2	4	2	2	4	2	2	3	3	2	3	2	3	3	3	3	2	3
chr21:8222001	chr21	8,222,001	8,223,000	5	3	5	2	4	2	2	4	2	2	3	3	2	3	2	3	3	3	3	2	3
chr21:8223001	chr21	8,223,001	8,224,000	5	3	5	2	4	2	2	4	2	2	3	3	2	3	2	3	3	3	3	2	3
chr21:8224001	chr21	8,224,001	8,225,000	5	3	5	2	4	2	2	4	2	2	3	3	2	3	2	3	3	3	3	2	3
chr21:8225001	chr21	8,225,001	8,226,000	5	3	5	2	4	2	2	4	2	2	3	3	2	3	2	3	3	3	3	2	3
chr21:8226001	chr21	8,226,001	8,227,000	5	3	5	2	4	2	2	4	2	2	3	3	2	3	2	3	3	3	3	2	3
chr21:8227001	chr21	8,227,001	8,228,000	5	3	5	2	4	2	2	4	2	2	3	3	2	3	2	3	3	3	3	2	3
chr21:8228001	chr21	8,228,001	8,229,000	5	3	5	2	4	2	2	4	2	2	3	3	2	3	2	3	3	3	3	2	3
chr21:8229001	chr21	8,229,001	8,230,000	5	3	5	2	4	2	2	4	2	2	3	3	2	3	2	3	3	3	3	2	3
chr21:8230001	chr21	8,230,001	8,231,000	5	3	5	2	4	2	2	4	2	2	3	3	2	3	2	3	3	3	3	2	3
chr21:8231001	chr21	8,231,001	8,232,000	5	3	5	2	4	2	2	4	2	2	3	3	2	3	2	3	3	3	3	2	3
chr21:8232001	chr21	8,232,001	8,233,000	5	3	5	2	4	2	2	4	2	2	3	3	2	3	2	3	3	3	3	2	3
chr21:8233001	chr21	8,233,001	8,234,000	5	3	5	2	4	2	2	4	2	2	3	3	2	3	2	3	3	3	3	2	3
chr21:8234001	chr21	8,234,001	8,235,000	5	3	5	2	4	2	2	4	2	2	3	3	2	3	2	3	3	3	3	2	3

Region	CHR	start	end	HD2	HD3	HD1	BN1	BN2	BN3	BN4	BN5	BN6	LC1	LC2	LC3	LC4	LC5	LC6	LC7	LC8	LC9	LC10	LC10*	LC11
chr21:8235001	chr21	8,235,001	8,236,000	5	3	5	2	4	2	2	4	2	2	3	3	2	3	2	3	3	3	3	2	3
chr21:8236001	chr21	8,236,001	8,237,000	5	3	5	2	4	2	2	4	2	2	3	3	2	3	2	3	3	3	3	2	3
chr21:8237001	chr21	8,237,001	8,238,000	5	3	5	2	4	2	2	4	2	2	3	3	2	3	2	3	3	3	3	2	3
chr21:8238001	chr21	8,238,001	8,239,000	5	3	5	2	4	2	2	4	2	2	3	3	2	3	2	3	3	3	3	2	3
chr21:8239001	chr21	8,239,001	8,240,000	5	3	5	2	4	2	2	4	2	2	3	3	2	3	2	3	3	3	3	2	3
chr21:8240001	chr21	8,240,001	8,241,000	5	3	5	2	4	2	2	4	2	2	3	3	2	3	2	3	3	3	3	2	3
chr21:8241001	chr21	8,241,001	8,242,000	5	3	5	2	4	2	2	4	2	2	3	3	2	3	2	3	3	3	3	2	3
chr21:8242001	chr21	8,242,001	8,243,000	5	3	5	2	4	2	2	4	2	2	3	3	2	3	2	3	3	3	3	2	3
chr21:8243001	chr21	8,243,001	8,244,000	5	3	5	2	4	2	2	4	2	2	3	3	2	3	2	3	3	3	3	2	3
chr21:8244001	chr21	8,244,001	8,245,000	5	3	5	2	4	2	2	4	2	2	3	3	2	3	2	3	3	3	3	2	3
chr21:8245001	chr21	8,245,001	8,246,000	5	3	5	2	4	2	2	4	2	2	3	3	2	3	2	3	3	3	3	2	3
chr21:8246001	chr21	8,246,001	8,247,000	5	3	5	2	4	2	2	4	2	2	3	3	2	3	2	3	3	3	3	2	3
chr21:8247001	chr21	8,247,001	8,248,000	5	3	5	2	4	2	2	4	2	2	3	3	2	3	2	3	3	3	3	2	3
chr21:8248001	chr21	8,248,001	8,249,000	5	3	5	2	4	2	2	4	2	2	3	3	2	3	2	3	3	3	3	2	3
chr21:8249001	chr21	8,249,001	8,250,000	5	3	5	2	4	2	2	4	2	2	3	3	2	3	2	3	3	3	3	2	3
chr21:8250001	chr21	8,250,001	8,251,000	5	3	5	2	4	2	2	4	2	2	3	3	2	3	2	3	3	3	3	2	3
chr21:8251001	chr21	8,251,001	8,252,000	5	3	5	2	4	2	2	4	2	2	3	3	2	3	2	3	3	3	3	2	3
chr21:8252001	chr21	8,252,001	8,253,000	5	3	5	2	4	2	2	4	2	2	3	3	2	3	2	3	3	3	3	2	3
chr21:8253001	chr21	8,253,001	8,254,000	5	3	5	2	4	2	2	4	2	2	3	3	2	3	2	3	3	3	3	2	3
chr21:8254001	chr21	8,254,001	8,255,000	5	3	5	2	4	2	2	4	2	2	3	3	2	3	2	3	3	3	3	2	3
chr21:8255001	chr21	8,255,001	8,256,000	5	3	5	2	4	2	2	4	2	2	3	3	2	3	2	3	3	3	3	2	3
chr21:8256001	chr21	8,256,001	8,257,000	5	3	5	2	4	2	2	4	2	2	3	3	2	3	2	3	3	3	3	2	3
chr21:8257001	chr21	8,257,001	8,258,000	5	3	5	2	4	2	2	4	2	2	3	3	2	3	2	3	3	3	3	2	3
chr21:8258001	chr21	8,258,001	8,259,000	5	3	5	2	4	2	2	4	2	2	3	3	2	3	2	3	3	3	3	2	3
chr21:8259001	chr21	8,259,001	8,260,000	5	3	5	2	4	2	2	4	2	2	3	3	2	3	2	3	3	3	3	2	3
chr21:8260001	chr21	8,260,001	8,261,000	5	3	5	2	4	2	2	4	2	2	3	3	2	3	2	3	3	3	3	2	3
chr21:8385001	chr21	8,385,001	8,386,000	5	3	5	2	4	2	2	4	2	2	3	3	2	3	2	3	3	3	3	2	3
chr21:8386001	chr21	8,386,001	8,387,000	5	3	5	2	4	2	2	4	2	2	3	3	2	3	2	3	3	3	3	2	3
chr21:8387001	chr21	8,387,001	8,388,000	5	3	5	2	4	2	2	4	2	2	3	3	2	3	2	3	3	3	3	2	3
chr21:8388001	chr21	8,388,001	8,389,000	5	3	5	2	4	2	2	4	2	2	3	3	2	3	2	3	3	3	3	2	3
chr21:8389001	chr21	8,389,001	8,390,000	5	3	5	2	4	2	2	4	2	2	3	3	2	3	2	3	3	3	3	2	3
chr21:8390001	chr21	8,390,001	8,391,000	5	3	5	2	4	2	2	4	2	2	3	3	2	3	2	3	3	3	3	2	3
chr21:8391001	chr21	8,391,001	8,392,000	5	3	5	2	4	2	2	4	2	2	3	3	2	3	2	3	3	3	3	2	3
chr21:8392001	chr21	8,392,001	8,393,000	5	3	5	2	4	2	2	4	2	2	3	3	2	3	2	3	3	3	3	2	3
chr21:8393001	chr21	8,393,001	8,394,000	5	3	5	2	4	2	2	4	2	2	3	3	2	3	2	3	3	3	3	2	3
chr21:8394001	chr21	8,394,001	8,395,000	5	3	5	2	4	2	2	4	2	2	3	3	2	3	2	3	3	3	3	2	3
chr21:8395001	chr21	8,395,001	8,396,000	5	3	5	2	4	2	2	4	2	2	3	3	2	3	2	3	3	3	3	2	3
chr21:8396001	chr21	8,396,001	8,397,000	5	3	5	2	4	2	2	4	2	2	3	3	2	3	2	3	3	3	3	2	3
chr21:8397001	chr21	8,397,001	8,398,000	5	3	5	2	4	2	2	4	2	2	3	3	2	3	2	3	3	3	3	2	3
chr21:8398001	chr21	8,398,001	8,399,000	5	3	5	2	4	2	2	4	2	2	3	3	2	3	2	3	3	3	3	2	3
chr21:8399001	chr21	8,399,001	8,400,000	5	3	5	2	4	2	2	4	2	2	3	3	2	3	2	3	3	3	3	2	3
chr21:8400001	chr21	8,400,001	8,401,000	5	3	5	2	4	2	2	4	2	2	3	3	2	3	2	3	3	3	3	2	3
chr21:8401001	chr21	8,401,001	8,402,000	5	3	5	2	4	2	2	4	2	2	3	3	2	3	2	3	3	3	3	2	3
chr21:8402001	chr21	8,402,001	8,403,000	5	3	5	2	4	2	2	4	2	2	3	3	2	3	2	3	3	3	3	2	3
chr21:8403001	chr21	8,403,001	8,404,000	5	3	5	2	4	2	2	4	2	2	3	3	2	3	2	3	3	3	3	2	3
chr21:8404001	chr21	8,404,001	8,405,000	5	3	5	2	4	2	2	4	2	2	3	3	2	3	2	3	3	3	3	2	3
chr21:8405001	chr21	8,405,001	8,406,000	5	3	5	2	4	2	2	4	2	2	3	3	2	3	2	3	3	3	3	2	3
chr21:8406001	chr21	8,406,001	8,407,000	5	3	5	2	4	2	2	4	2	2	3	3	2	3	2	3	3	3	3	2	3
chr21:8407001	chr21	8,407,001	8,408,000	5	3	5	2	4	2	2	4	2	2	3	3	2	3	2	3	3	3	3	2	3
chr21:8408001	chr21	8,408,001	8,409,000	5	3	5	2	4	2	2	4	2	2	3	3	2	3	2	3	3	3	3	2	3
chr21:8409001	chr21	8,409,001	8,410,000	5	3	5	2	4	2	2	4	2	2	3	3	2	3	2	3	3	3	3	2	3
chr21:8410001	chr21	8,410,001	8,411,000	5	3	5	2	4	2	2	4	2	2	3	3	2	3	2	3	3	3	3	2	3
chr21:8411001	chr21	8,411,001	8,412,000	5	3	5	2	4	2	2	4	2	2	3	3	2	3	2	3	3	3	3	2	3
chr21:8412001	chr21	8,412,001	8,413,000	5	3	5	2	4	2	2	4	2	2	3	3	2	3	2	3	3	3	3	2	3
chr21:8413001	chr21	8,413,001	8,414,000	5	3	5	2	4	2	2	4	2	2	3	3	2	3	2	3	3	3	3	2	3
chr21:8414001	chr21	8,414,001	8,415,000	5	3	5	2	4	2	2	4	2	2	3	3	2	3	2	3	3	3	3	2	3
chr21:8415001	chr21	8,415,001	8,416,000	5	3	5	2	4	2	2	4	2	2	3	3	2	3	2	3	3	3	3	2	3
chr21:8416001	chr21	8,416,001	8,417,000	5	3	5	2	4	2	2	4	2	2	3	3	2	3	2	3	3	3	3	2	3
chr21:8417001	chr21	8,417,001	8,418,000	5	3	5	2	4	2	2	4	2	2	3	3	2	3	2	3	3	3	3	2	3
chr21:8418001	chr21	8,418,001	8,419,000	5	3	5	2	4	2	2	4	2	2	3	3	2	3	2	3	3	3	3	2	3
chr21:8419001	chr21	8,419,001	8,420,000	5	3	5	2	4	2	2	4	2	2	3	3	2	3	2	3	3	3	3	2	3
chr21:8420001	chr21	8,420,001	8,421,000	5	3	5	2	4	2	2	4	2	2	3	3	2	3	2	3	3	3	3	2	3
chr21:8421001	chr21	8,421,001	8,422,000	5	3	5	2	4	2	2	4	2	2	3	3	2	3	2	3	3	3	3	2	3
chr21:8422001	chr21	8,422,001	8,423,000	5	3	5	2	4	2	2	4	2	2	3	3	2	3	2	3	3	3	3	2	3
chr21:8423001	chr21	8,423,001	8,424,000	5	3	5	2	4	2	2	4	2	2	3	3	2	3	2	3	3	3	3	2	3
chr21:8424001	chr21	8,424,001	8,425,000	5	3	5	2	4	2	2	4	2	2	3	3	2	3	2	3	3	3	3	2	3
chr21:8425001	chr21	8,425,001	8,426,000	5	3	5	2	4	2	2	4	2	2	3	3	2	3	2	3	3	3	3	2	3
chr21:8426001	chr21	8,426,001	8,427,000	5	3	5	2	4	2	2	4	2	2	3	3	2	3	2	3	3	3	3	2	3
chr21:8427001	chr21	8,427,001	8,428,000	5	3	5	2	4	2	2	4	2	2	3	3	2	3	2	3	3	3	3	2	3
chr21:8428001	chr21	8,428,001	8,429,000	5	3	5	2	4	2	2	4	2	2	3	3	2	3	2	3	3	3	3	2	3
chr21:8429001	chr21	8,429,001	8,430,000	5	3	5	2	4	2	2	4	2	2	3	3	2	3	2	3	3	3	3	2	3
chr21:8430001	chr21	8,430,001	8,431,000	5	3	5	2	4	2	2	4	2	2	3	3	2	3	2	3	3	3	3	2	3
chr21:8431001	chr21	8,431,001	8,432,000	5	3	5	2	4	2	2	4	2	2	3	3	2	3	2	3	3	3	3	2	3
chr21:8432001	chr21	8,432,001	8,433,000	5	3	5	2	4	2	2	4	2	2	3	3	2	3	2	3	3	3	3	2	3
chr21:8433001	chr21	8,433,001	8,434,000	5	3	5	2	4	2	2	4	2	2	3	3	2	3	2	3	3	3	3	2	3
chr21:8434001	chr21	8,434,001	8,435,000	5	3	5	2	4	2	2	4	2	2	3	3	2	3	2	3	3	3	3	2	3
chr21:8435001	chr21	8,435,001	8,436,000	5	3	5	2	4	2	2	4	2	2	3	3	2	3	2	3	3	3	3	2	3
chr21:8436001	chr21	8,436,001	8,437,000	5	3	5	2	4	2	2	4	2	2	3	3	2	3	2	3	3	3	3	2	3
chr21:8437001	chr21	8,437,001	8,438,000	5	3	5	2	4	2	2	4	2	2	3	3	2	3	2	3	3	3	3	2	3
chr21:8438001	chr21	8,438,001	8,439,000	5	3	5	2	4	2	2	4	2	2	3	3	2	3	2	3	3	3	3	2	3
chr21:8439001	chr21	8,439,001	8,440,000	5	3	5	2	4	2	2	4	2	2	3	3	2	3	2	3	3	3	3	2	3
chr21:8440001	chr21	8,440,001	8,441,000	5	3	5	2	4	2	2	4	2	2	3	3	2	3	2	3	3	3	3	2	3
chr21:8441001	chr21	8,441,001	8,442,000	5	3	5	2	4	2	2	4	2	2	3	3	2	3	2	3	3	3	3	2	3
chr21:8442001	chr21	8,442,001	8,443,000	5	3	5	2	4	2	2	4	2	2	3	3	2	3	2	3	3	3	3	2	3
chr21:8443001	chr21	8,443,001	8,444,000	5	3	5	2	4	2	2	4	2	2	3	3	2	3	2	3	3	3	3	2	3
chr21:8444001	chr21	8,444,001	8,445,000	5	3	5	2	4	2	2	4	2	2	3	3	2	3	2	3	3	3	3	2	3
chr21:8445001	chr21	8,445,001	8,446,000	5	3	5	2	4	2	2	4	2	2	3	3	2	3	2	3	3	3	3	2	3
chr21:8446001	chr21	8,446,001	8,447,000	5	3	5	2	4	2	2	4	2	2	3	3	2	3	2	3	3	3	3	2	3
chr21:8447001	chr21	8,447,001	8,448,000	5	3	5	2	4	2	2	4	2	2	3	3	2	3	2	3	3	3	3	2	3
chr21:8448001	chr21	8,448,001	8,449,000	5	3	5	2	4	2	2	4	2	2	3	3	2	3	2	3	3	3	3	2	3
chr21:8449001	chr21	8,449,001	8,450,000	5	3	5	2	4	2	2	4	2	2	3	3	2	3	2	3	3	3	3	2	3
chr21:8450001	chr21	8,450,001	8,451,000	5	3	5	2	4	2	2	4	2	2	3	3	2	3	2	3	3	3	3	2	3
chr21:8451001	chr21	8,451,001	8,452,000	5	3	5	2	4	2	2	4	2	2	3	3	2	3	2	3	3	3	3	2	3
chr21:8452001	chr21	8,452,001	8,453,000	5	3	5	2	4	2	2	4	2	2	3	3	2	3	2	3	3	3	3	2	3
chr21:8453001	chr21	8,453,001	8,454,000	5	3	5	2	4	2	2	4	2	2	3	3	2	3	2	3	3	3	3	2	3
chr21:8454001	chr21	8,454,001	8,455,000	5	3	5	2	4	2	2	4	2	2	3	3	2	3	2	3	3	3	3	2	3
chr21:9805001	chr21	9,805,001	9,806,000	4	4	5	2	3	2	2	2	3	3	2	3	2	2	2	3	3	3	3	3	3
chr21:9806001	chr21	9,806,001	9,807,000	4	4	5	2	3	2	2	2	3	3	2	3	2	2	2	3	3	3	3	3	3
chr21:9807001	chr21	9,807,001	9,808,000	4	4	5	2	3	2	2	2	3	3	2	3	2	2	2	3	3	3	3	3	3
chr21:9808001	chr21	9,808,001	9,809,000	4	4	5	2	3	2	2	2	3	3	2	3	2	2	2	3	3	3	3	3	3
chr21:9809001	chr21	9,809,001	9,810,000	4	4	5	2	3	2	2	2	3	3	2	3	2	2	2	4	3	3	3	2	3
chr2:91862001	chr2	91,862,001	91,863,000	3	3	4	2	2	2	2	2	3	3	3	3	3	2	2	3	2	3	3	2	3
chr5:69899001	chr5	69,899,001	69,900,000	4	4	4	4	4	3	3	3	4	3	3	2	2	4	2	2	3	3	2	3	3
chr7:57938001	chr7	57,938,001	57,939,000	4	4	4	3	3	3	2	3	3	3	3	3	3	2	3	3	3	2	2	2	3
chr9:41634001	chr9	41,634,001	41,635,000	3	3	4	2	2	2	2	2	2	3	3	3	2	3	2	2	3	3	3	3	2
chr9:63916001	chr9	63,916,001	63,917,000	3	3	4	2	2	2	2	2	2	3	3	3	2	2	2	3	2	3	3	3	3
chr9:63917001	chr9	63,917,001	63,918,000	3	3	4	2	2	2	2	2	2	3	3	3	2	2	2	3	2	3	3	3	3
chr9:63918001	chr9	63,918,001	63,919,000	3	3	4	2	2	2	2	2	2	3	3	3	2	2	2	3	2	3	3	3	3
chr9:67380001	chr9	67,380,001	67,381,000	4	3	4	3	2	3	2	2	2	3	3	3	2	3	2	3	3	2	3	3	2
chr12:37733001	chr12	37,733,001	37,734,000	4	5	4	3	3	2	2	2	3	3	4	2	2	2	2	3	3	4	2	2	3
chr16:36126001	chr16	36,126,001	36,127,000	3	3	4	2	2	2	2	2	3	3	3	3	2	2	2	3	2	3	3	3	3
chr16:36127001	chr16	36,127,001	36,128,000	3	3	4	2	2	2	2	2	3	3	3	3	2	2	2	3	2	3	3	3	3
chr17:21846001	chr17	21,846,001	21,847,000	3	4	4	3	2	2	2	2	2	3	2	3	2	3	2	3	3	3	3	2	3

Region	CHR	start	end	HD2	HD3	HD1	BN1	BN2	BN3	BN4	BN5	BN6	LC1	LC2	LC3	LC4	LC5	LC6	LC7	LC8	LC9	LC10	LC10*	LC11
chr17:26775001	chr17	26,775,001	26,776,000	3	3	4	2	2	2	2	2	3	3	3	3	3	2	2	3	2	3	3	3	2
chr17:26971001	chr17	26,971,001	26,972,000	3	4	4	3	2	2	2	2	3	3	3	3	2	2	2	3	2	3	3	3	3
chr17:26972001	chr17	26,972,001	26,973,000	3	4	4	3	2	2	2	2	3	3	3	3	2	2	2	3	2	3	3	3	3
chr21:9670001	chr21	9,670,001	9,671,000	4	4	4	2	2	2	2	2	3	3	2	3	3	2	2	3	3	3	3	2	3
chr21:9671001	chr21	9,671,001	9,672,000	4	4	4	2	2	2	2	2	3	3	2	3	3	2	2	3	3	3	3	2	3
chr21:9710001	chr21	9,710,001	9,711,000	4	4	4	2	2	2	2	2	3	3	2	3	3	2	2	3	3	3	3	2	3
chr21:9711001	chr21	9,711,001	9,712,000	4	4	4	2	2	2	2	2	3	3	2	3	3	2	2	3	3	3	3	2	3
chr21:9712001	chr21	9,712,001	9,713,000	4	4	4	2	2	2	2	2	3	3	2	3	3	2	2	3	3	3	3	2	3
chr21:9713001	chr21	9,713,001	9,714,000	4	4	4	2	2	2	2	2	3	3	2	3	3	2	2	3	3	3	3	2	3
chr21:9714001	chr21	9,714,001	9,715,000	4	4	4	2	2	2	2	2	3	3	2	3	3	2	2	3	3	3	3	2	3
chr21:9715001	chr21	9,715,001	9,716,000	4	4	4	2	2	2	2	2	3	3	2	3	3	2	2	3	3	3	3	2	3
chr21:9716001	chr21	9,716,001	9,717,000	4	4	4	2	2	2	2	2	3	3	2	3	3	2	2	3	3	3	3	2	3
chr21:9717001	chr21	9,717,001	9,718,000	4	4	4	2	2	2	2	2	3	3	2	3	3	2	2	3	3	3	3	2	3
chr21:9718001	chr21	9,718,001	9,719,000	4	4	4	3	2	2	2	2	3	3	2	3	3	2	2	3	3	3	3	2	3
chr21:9797001	chr21	9,797,001	9,798,000	4	4	4	2	3	2	2	2	3	3	2	3	2	2	2	3	3	3	3	3	3
chr21:9798001	chr21	9,798,001	9,799,000	4	4	4	2	3	2	2	2	3	3	2	3	2	2	2	3	3	3	3	3	3
chr21:9799001	chr21	9,799,001	9,800,000	4	4	4	2	3	2	2	2	3	3	2	3	2	2	2	3	3	3	3	3	3
chr21:9800001	chr21	9,800,001	9,801,000	4	4	4	2	3	2	2	2	3	3	2	3	2	2	2	3	3	3	3	3	3
chr21:9801001	chr21	9,801,001	9,802,000	4	4	4	2	3	2	2	2	3	3	2	3	2	2	2	3	3	3	3	3	3
chr21:9802001	chr21	9,802,001	9,803,000	4	4	4	2	3	2	2	2	3	3	2	3	2	2	2	3	3	3	3	3	3
chr21:9803001	chr21	9,803,001	9,804,000	4	4	4	2	3	2	2	2	3	3	2	3	2	2	2	3	3	3	3	3	3
chr21:9804001	chr21	9,804,001	9,805,000	4	4	4	2	3	2	2	2	3	3	2	3	2	2	2	3	3	3	3	3	3
chr21:10120001	chr21	10,120,001	10,121,000	3	3	4	2	2	2	2	2	3	3	2	3	2	2	2	3	3	3	3	3	3
chr21:10121001	chr21	10,121,001	10,122,000	4	3	4	2	2	2	2	2	3	3	2	3	2	2	2	3	3	3	3	3	3
chr21:10575001	chr21	10,575,001	10,576,000	4	3	4	2	2	2	2	2	3	2	2	3	2	2	3	3	3	3	3	3	3
chr21:10576001	chr21	10,576,001	10,577,000	4	3	4	2	2	2	2	2	3	2	2	3	2	2	3	3	3	3	3	3	3
chr21:10577001	chr21	10,577,001	10,578,000	4	3	4	2	2	2	2	2	3	2	2	3	2	2	3	3	3	3	3	3	3
chr21:10578001	chr21	10,578,001	10,579,000	4	3	4	2	2	2	2	2	3	2	2	3	2	2	3	3	3	3	3	3	3
chr21:10579001	chr21	10,579,001	10,580,000	4	3	4	2	2	2	2	2	3	2	2	3	2	2	3	3	3	3	3	3	3
chr21:10580001	chr21	10,580,001	10,581,000	4	3	4	2	2	2	2	2	3	2	2	3	2	2	3	3	3	3	3	3	3
chr21:10581001	chr21	10,581,001	10,582,000	4	3	4	2	2	2	2	2	3	2	2	3	2	2	3	3	3	3	3	3	3
chr21:10582001	chr21	10,582,001	10,583,000	4	3	4	2	2	2	2	2	3	2	2	3	2	2	3	3	3	3	3	3	3
chr21:10583001	chr21	10,583,001	10,584,000	4	3	4	2	2	2	2	2	3	2	2	3	2	2	3	3	3	3	3	3	3
chr21:10584001	chr21	10,584,001	10,585,000	4	3	4	2	2	2	2	2	3	2	2	3	2	2	3	3	3	3	3	3	3
chr21:10585001	chr21	10,585,001	10,586,000	4	3	4	2	2	2	2	2	3	2	2	3	2	2	3	3	3	3	3	3	3
chr21:10586001	chr21	10,586,001	10,587,000	4	3	4	2	3	2	2	2	3	2	2	3	2	2	3	3	3	3	3	3	3
chr21:10587001	chr21	10,587,001	10,588,000	4	3	4	2	3	2	2	2	3	2	2	3	2	2	3	3	3	3	3	3	3
chr21:10588001	chr21	10,588,001	10,589,000	4	3	4	2	3	2	2	2	3	2	2	3	2	2	3	3	3	3	3	3	3
chr21:10589001	chr21	10,589,001	10,590,000	4	3	4	2	3	2	2	2	3	2	2	3	2	2	3	3	3	3	3	3	3
chr21:10590001	chr21	10,590,001	10,591,000	4	3	4	2	3	2	2	2	3	2	2	3	2	2	3	3	3	3	3	3	3
chr21:10591001	chr21	10,591,001	10,592,000	4	3	4	2	3	2	2	2	3	2	2	3	2	2	3	3	3	3	3	3	3
chr21:10628001	chr21	10,628,001	10,629,000	4	3	4	2	2	2	2	2	3	2	2	3	2	2	3	3	3	3	3	3	3
chr21:10629001	chr21	10,629,001	10,630,000	4	3	4	2	2	2	2	2	3	2	2	3	2	2	3	3	3	3	3	3	3
chr21:10630001	chr21	10,630,001	10,631,000	4	3	4	2	2	2	2	2	3	2	2	3	2	2	3	3	3	3	3	3	3
chr21:10631001	chr21	10,631,001	10,632,000	4	3	4	2	2	2	2	2	3	2	2	3	2	2	3	3	3	3	3	3	3
chr21:10632001	chr21	10,632,001	10,633,000	4	3	4	2	2	2	2	2	3	2	2	3	2	2	3	3	3	3	3	3	3
chr1:145364001	chr1	145,364,001	145,365,000	2	2	3	2	2	2	1	2	2	2	2	3	3	3	2	3	2	3	3	3	3
chr1:145365001	chr1	145,365,001	145,366,000	2	2	3	2	2	2	1	2	2	2	2	3	3	3	2	3	2	3	3	3	3
chr3:197651001	chr3	197,651,001	197,652,000	3	3	3	2	2	2	2	2	2	2	3	2	2	3	2	3	3	3	3	3	3
chr9:39570001	chr9	39,570,001	39,571,000	4	3	3	2	3	2	2	2	2	2	3	2	3	2	3	3	3	3	3	3	2
chr9:39770001	chr9	39,770,001	39,771,000	3	3	3	2	2	2	2	2	2	3	3	3	2	3	2	3	2	2	3	3	3
chr9:39771001	chr9	39,771,001	39,772,000	3	3	3	2	2	2	2	2	2	3	3	3	2	3	2	3	2	2	3	3	3
chr9:39772001	chr9	39,772,001	39,773,000	3	3	3	2	2	2	2	2	2	3	3	3	2	3	2	3	2	2	3	3	3
chr9:67870001	chr9	67,870,001	67,871,000	3	3	3	2	2	2	2	2	2	3	3	3	2	3	2	3	3	2	3	3	2
chr9:67871001	chr9	67,871,001	67,872,000	3	3	3	2	2	2	2	2	2	3	3	3	2	3	2	3	3	2	3	3	2
chr9:67910001	chr9	67,910,001	67,911,000	5	4	3	3	3	2	2	2	2	3	3	2	2	3	2	3	3	3	3	3	2
chr9:67911001	chr9	67,911,001	67,912,000	5	4	3	3	2	2	2	2	2	3	3	2	2	3	2	3	3	3	3	3	2
chr16:33039001	chr16	33,039,001	33,040,000	3	3	3	2	2	2	2	2	2	3	2	3	3	3	2	3	2	3	3	3	2
chr16:34186001	chr16	34,186,001	34,187,000	4	4	3	3	3	2	2	2	2	3	3	3	3	2	2	3	2	3	3	2	3
chr16:34204001	chr16	34,204,001	34,205,000	4	4	3	3	2	2	2	2	3	3	3	3	2	2	2	3	2	3	3	3	3
chr16:34205001	chr16	34,205,001	34,206,000	4	4	3	3	2	2	2	2	3	3	3	3	2	2	2	3	2	3	3	3	3
chr16:34212001	chr16	34,212,001	34,213,000	3	4	3	3	2	2	2	2	2	3	3	3	2	2	2	3	2	3	3	3	3
chr16:34213001	chr16	34,213,001	34,214,000	3	4	3	3	2	2	2	2	2	3	3	3	2	2	2	3	2	3	3	3	3
chr19:39889001	chr19	39,889,001	39,890,000	3	4	3	3	2	2	2	2	2	2	3	2	3	2	3	2	3	3	3	3	3
chr22:18766001	chr22	18,766,001	18,767,000	3	3	3	1	1	2	2	2	2	2	3	3	4	3	3	2	3	2	2	2	3
chr22:21146001	chr22	21,146,001	21,147,000	3	3	3	2	2	2	2	2	2	3	3	3	3	3	3	3	2	2	2	2	3
chr22:21164001	chr22	21,164,001	21,165,000	3	3	3	2	2	2	2	2	2	3	3	3	3	3	3	3	3	2	2	2	2
chr1:722001	chr1	722,001	723,000	2	2	2	3	3	3	3	3	3	2	3	2	3	3	3	3	3	2	2	3	3
chr1:723001	chr1	723,001	724,000	2	2	2	3	3	3	3	3	3	2	3	2	3	3	3	3	3	2	2	3	3
chr1:724001	chr1	724,001	725,000	2	2	2	3	3	3	3	3	3	2	3	2	3	3	3	3	3	2	2	3	3
chr1:725001	chr1	725,001	726,000	2	2	2	3	3	3	3	3	3	2	3	2	3	3	3	3	3	2	2	3	3
chr1:726001	chr1	726,001	727,000	2	2	2	3	3	3	3	3	3	2	3	2	3	3	3	3	3	2	2	3	3
chr1:741001	chr1	741,001	742,000	2	2	2	3	4	3	3	3	3	2	3	2	3	3	3	3	3	3	2	2	3
chr1:13236001	chr1	13,236,001	13,237,000	3	2	2	3	4	4	4	3	4	3	3	3	3	2	3	4	3	2	2	2	2
chr1:13237001	chr1	13,237,001	13,238,000	3	2	2	3	4	4	4	3	4	3	3	3	3	2	3	4	3	2	2	2	2
chr1:13238001	chr1	13,238,001	13,239,000	3	2	2	3	4	4	4	3	4	3	3	3	3	2	3	4	3	2	2	2	2
chr1:13239001	chr1	13,239,001	13,240,000	3	2	2	3	4	4	4	3	4	3	3	3	3	2	3	4	3	2	2	2	2
chr7:144226001	chr7	144,226,001	144,227,000	2	2	2	3	3	3	3	3	4	3	2	2	3	3	3	3	3	3	2	2	3
chr16:122001	chr16	122,001	123,000	2	2	2	3	3	3	3	3	3	2	2	2	3	3	3	3	3	3	2	3	3
chr16:123001	chr16	123,001	124,000	2	2	2	3	3	3	3	3	3	2	2	2	3	3	3	3	3	3	2	3	3
chr16:124001	chr16	124,001	125,000	2	2	2	3	3	3	3	3	3	2	2	2	3	3	3	3	3	3	2	3	3
chr16:125001	chr16	125,001	126,000	2	2	2	3	3	3	3	3	3	2	2	2	3	3	3	3	3	3	2	3	3
chr16:126001	chr16	126,001	127,000	2	2	2	3	3	3	3	3	3	2	2	2	3	3	3	3	3	3	2	3	3
chr16:127001	chr16	127,001	128,000	2	2	2	3	3	3	3	3	3	2	2	2	3	3	3	3	3	3	2	3	3
chr16:128001	chr16	128,001	129,000	2	2	2	3	3	3	3	3	3	2	2	2	3	3	3	3	3	3	2	3	3
chr16:32221001	chr16	32,221,001	32,222,000	2	2	2	2	2	2	2	2	2	3	3	2	2	2	2	3	3	3	3	3	3
chr16:32222001	chr16	32,222,001	32,223,000	2	2	2	2	2	2	2	2	2	3	3	2	2	2	2	3	3	3	3	3	3
chr16:32223001	chr16	32,223,001	32,224,000	2	2	2	2	2	2	2	2	2	3	3	2	2	2	2	3	3	3	3	3	3
chr16:32224001	chr16	32,224,001	32,225,000	2	2	2	2	2	2	2	2	2	3	3	2	2	2	2	3	3	3	3	3	3
chr16:32225001	chr16	32,225,001	32,226,000	2	2	2	2	2	2	2	2	2	3	3	2	2	2	2	3	3	3	3	3	3
chr16:32226001	chr16	32,226,001	32,227,000	2	2	2	2	2	2	2	2	2	3	3	2	2	2	2	3	3	3	3	3	3
chr16:32227001	chr16	32,227,001	32,228,000	2	2	2	2	2	2	2	2	2	3	3	2	2	2	2	3	3	3	3	3	3
chr16:32228001	chr16	32,228,001	32,229,000	2	2	2	2	2	2	2	2	2	3	3	2	2	2	2	3	3	3	3	3	3
chr15:101682001	chr15	101,682,001	101,683,000	2	2	1	2	3	3	3	3	3	2	2	2	3	3	3	3	3	3	2	3	3
chr15:101683001	chr15	101,683,001	101,684,000	2	2	1	2	3	3	3	3	3	2	2	2	3	3	3	3	3	3	2	3	3
chr15:101686001	chr15	101,686,001	101,687,000	2	2	1	2	3	3	3	3	3	2	2	3	3	3	3	2	3	3	2	3	3
chr15:101687001	chr15	101,687,001	101,688,000	2	2	1	2	3	3	3	3	3	2	2	3	3	3	3	2	3	3	2	3	3
chr15:101709001	chr15	101,709,001	101,710,000	2	2	1	2	3	4	3	3	3	3	3	3	3	3	3	3	3	2	2	2	2
chr15:101710001	chr15	101,710,001	101,711,000	2	2	1	2	3	4	3	3	3	3	3	3	3	3	3	3	3	2	2	2	2
chr15:101711001	chr15	101,711,001	101,712,000	2	2	1	2	3	4	3	3	3	3	3	3	3	3	3	3	3	2	2	2	2
chr15:101719001	chr15	101,719,001	101,720,000	2	2	1	2	3	4	3	3	3	3	2	3	3	3	3	3	3	2	2	3	2
chr15:101722001	chr15	101,722,001	101,723,000	2	2	1	2	3	3	3	3	3	3	3	3	3	3	3	3	3	2	2	2	2
chr15:101808001	chr15	101,808,001	101,809,000	2	2	1	2	3	4	4	3	3	2	2	3	3	3	3	3	3	2	2	3	3
chr15:101809001	chr15	101,809,001	101,810,000	2	2	1	2	3	4	4	3	3	2	2	3	3	3	3	3	3	2	2	3	3
chr15:101810001	chr15	101,810,001	101,811,000	2	2	1	2	3	4	4	3	3	2	2	3	3	3	3	3	3	2	2	3	3
chr15:101819001	chr15	101,819,001	101,820,000	2	2	1	2	3	3	4	3	3	2	2	3	3	3	3	3	3	2	2	3	3
chr15:101820001	chr15	101,820,001	101,821,000	2	2	1	2	3	3	4	3	3	2	2	3	3	3	3	3	3	2	2	3	3
chr15:101821001	chr15	101,821,001	101,822,000	2	2	1	2	3	3	4	3	3	2	2	3	3	3	3	3	3	2	2	3	3
chr15:101822001	chr15	101,822,001	101,823,000	2	2	1	2	3	3	4	3	3	2	2	3	3	3	3	3	3	2	2	3	3
chr15:101823001	chr15	101,823,001	101,824,000	2	2	1	2	3	3	4	3	3	2	2	3	3	3	3	3	3	2	2	3	3
chr21:13113001	chr21	13,113,001	13,114,000	2	1	1	3	3	2	3	2	2	3	2	3	3	3	2	3	2	3	3	3	2
chr21:13114001	chr21	13,114,001	13,115,000	2	1	1	3	3	2	3	2	2	3	2	3	3	3	2	3	2	3	3	3	2
chr21:13115001	chr21	13,115,001	13,116,000	2	1	1	3	3	2	3	2	2	3	2	3	3	3	2	3	2	3	3	3	2

Region	CHR	start	end	HD2	HD3	HD1	BN1	BN2	BN3	BN4	BN5	BN6	LC1	LC2	LC3	LC4	LC5	LC6	LC7	LC8	LC9	LC10	LC10*	LC11
chr21:13116001	chr21	13,116,001	13,117,000	2	1	1	3	3	2	3	2	2	3	2	3	3	3	2	3	2	3	3	3	2
chr21:13117001	chr21	13,117,001	13,118,000	2	1	1	3	3	2	3	2	2	3	2	3	3	3	2	3	2	3	3	3	2
chr21:13118001	chr21	13,118,001	13,119,000	2	1	1	3	3	2	3	2	2	3	2	3	3	3	2	3	2	3	3	3	2
chr21:13158001	chr21	13,158,001	13,159,000	2	1	1	3	2	2	3	2	2	3	2	3	3	2	2	3	3	3	3	3	2
chr21:13159001	chr21	13,159,001	13,160,000	2	1	1	3	2	2	3	2	2	3	2	3	3	2	2	3	3	3	3	3	2
chr21:13073001	chr21	13,073,001	13,074,000	2	0	0	3	3	1	3	1	2	3	2	3	3	2	2	3	3	3	3	3	2
chr2:87907001	chr2	87,907,001	87,908,000	4	5	5	2	2	2	2	2	2	2	2	2	2	2	2	3	2	3	5	4	2
chr2:109851001	chr2	109,851,001	109,852,000	4	5	5	2	2	2	2	2	2	2	2	2	2	2	2	2	2	4	5	4	2
chr4:19077001	chr4	19,077,001	19,078,000	5	5	5	2	2	2	2	2	2	2	2	2	3	2	2	2	2	2	5	5	2
chr5:69895001	chr5	69,895,001	69,896,000	4	4	5	4	4	3	3	2	4	3	3	2	2	4	2	2	3	3	2	2	3
chr5:69896001	chr5	69,896,001	69,897,000	4	4	5	4	4	3	3	2	4	3	3	2	2	4	2	2	3	3	2	2	3
chr5:69897001	chr5	69,897,001	69,898,000	4	4	5	4	4	3	3	2	4	3	3	2	2	4	2	2	3	3	2	2	3
chr6:160100001	chr6	160,100,001	160,101,000	5	5	5	2	2	2	2	2	2	2	3	2	2	2	2	2	2	2	5	5	2
chr7:107770001	chr7	107,770,001	107,771,000	5	5	5	2	2	2	2	2	2	2	2	2	2	2	2	2	2	3	5	5	2
chr9:43181001	chr9	43,181,001	43,182,000	3	5	5	3	2	2	2	2	3	3	3	3	2	2	2	3	2	3	3	2	3
chr9:43182001	chr9	43,182,001	43,183,000	3	5	5	3	2	2	2	2	3	3	3	3	2	2	2	3	2	3	3	2	3
chr9:43183001	chr9	43,183,001	43,184,000	4	5	5	3	2	2	2	2	3	3	3	3	2	2	2	3	2	3	3	2	3
chr9:43184001	chr9	43,184,001	43,185,000	4	5	5	3	2	2	2	2	3	3	3	3	2	2	2	3	2	3	3	2	3
chr9:43185001	chr9	43,185,001	43,186,000	4	5	5	3	2	2	2	2	3	3	3	3	2	2	2	3	2	3	3	2	3
chr9:43186001	chr9	43,186,001	43,187,000	4	5	5	3	2	2	2	2	3	3	3	3	2	2	2	3	2	3	3	2	3
chr9:43187001	chr9	43,187,001	43,188,000	4	5	5	3	2	2	2	2	3	3	3	3	2	2	2	3	2	3	3	2	3
chr9:60655001	chr9	60,655,001	60,656,000	5	4	5	4	3	3	2	3	3	3	4	5	2	2	2	2	3	2	1	2	3
chr11:16565001	chr11	16,565,001	16,566,000	4	4	5	2	2	2	2	2	2	2	2	2	2	2	2	2	2	3	5	5	2
chr12:131588001	chr12	131,588,001	131,589,000	5	5	5	2	2	2	2	2	2	4	4	2	2	2	2	4	2	3	2	2	2
chr15:19903001	chr15	19,903,001	19,904,000	3	3	5	3	2	2	2	2	2	3	3	3	3	2	2	3	3	2	3	2	2
chr15:19904001	chr15	19,904,001	19,905,000	3	3	5	3	2	2	2	2	2	3	3	3	3	2	2	3	3	2	3	2	2
chr15:20632001	chr15	20,632,001	20,633,000	4	3	5	3	4	3	3	3	4	3	3	2	3	2	3	4	3	2	2	2	2
chr15:20633001	chr15	20,633,001	20,634,000	4	3	5	3	4	3	3	3	4	3	3	2	3	2	3	4	3	2	2	2	2
chr15:20634001	chr15	20,634,001	20,635,000	4	3	5	3	4	3	3	3	4	3	3	2	3	2	3	4	3	2	2	2	2
chr15:20635001	chr15	20,635,001	20,636,000	4	3	5	3	4	3	3	3	4	3	3	2	3	2	3	4	3	2	2	2	2
chr21:8202001	chr21	8,202,001	8,203,000	5	3	5	2	4	2	2	4	2	2	3	3	2	3	2	3	3	2	3	2	3
chr5:70783001	chr5	70,783,001	70,784,000	4	5	4	3	4	2	2	2	4	3	3	2	2	4	2	2	3	3	2	2	3
chr5:71203001	chr5	71,203,001	71,204,000	4	4	4	3	3	3	3	2	4	3	3	2	2	4	3	2	3	3	2	2	2
chr5:71204001	chr5	71,204,001	71,205,000	4	4	4	3	3	3	3	2	4	3	3	2	2	4	3	2	3	3	2	2	2
chr5:71205001	chr5	71,205,001	71,206,000	4	4	4	3	4	3	3	2	4	3	3	2	2	4	3	2	3	3	2	2	2
chr5:71206001	chr5	71,206,001	71,207,000	4	4	4	3	4	3	3	2	4	3	3	2	2	4	3	2	3	3	2	2	2
chr5:71207001	chr5	71,207,001	71,208,000	4	4	4	3	4	4	3	2	4	3	3	2	2	4	3	2	3	3	2	2	2
chr9:67381001	chr9	67,381,001	67,382,000	4	3	4	3	2	3	2	2	2	3	3	3	2	3	2	3	2	2	3	3	2
chr10:39486001	chr10	39,486,001	39,487,000	4	3	4	2	2	2	2	2	2	3	3	3	2	2	2	3	2	3	3	2	3
chr10:39487001	chr10	39,487,001	39,488,000	4	3	4	2	2	2	2	2	2	3	3	3	2	2	2	3	2	3	3	2	3
chr17:21845001	chr17	21,845,001	21,846,000	3	4	4	3	2	2	2	2	2	3	2	3	2	3	2	3	2	3	3	2	3
chr17:22123001	chr17	22,123,001	22,124,000	3	4	4	3	2	2	2	2	3	3	2	3	2	2	2	3	2	3	3	3	3
chr17:22124001	chr17	22,124,001	22,125,000	4	4	4	3	2	2	2	2	3	3	2	3	2	2	2	3	2	3	3	3	3
chr17:22125001	chr17	22,125,001	22,126,000	4	4	4	3	2	2	2	2	3	3	2	3	2	2	2	3	2	3	3	3	3
chr17:22126001	chr17	22,126,001	22,127,000	4	4	4	3	2	2	2	2	3	3	2	3	2	2	2	3	2	3	3	3	3
chr17:22127001	chr17	22,127,001	22,128,000	4	4	4	3	2	2	2	2	3	3	2	3	2	2	2	3	2	3	3	3	3
chr17:22128001	chr17	22,128,001	22,129,000	4	4	4	3	2	2	2	2	3	3	2	3	2	2	2	3	2	3	3	3	3
chr17:22129001	chr17	22,129,001	22,130,000	4	4	4	3	2	2	2	2	3	3	2	3	2	2	2	3	2	3	3	3	3
chr17:22130001	chr17	22,130,001	22,131,000	4	4	4	3	2	2	2	2	3	3	2	3	2	2	2	3	2	3	3	3	3
chr17:22131001	chr17	22,131,001	22,132,000	4	4	4	3	2	2	2	2	3	3	2	3	2	2	2	3	2	3	3	3	3
chr17:22132001	chr17	22,132,001	22,133,000	4	4	4	3	2	2	2	2	3	3	2	3	2	2	2	3	2	3	3	3	3
chr17:22133001	chr17	22,133,001	22,134,000	4	4	4	3	2	2	2	2	3	3	2	3	2	2	2	3	2	3	3	3	3
chr17:22134001	chr17	22,134,001	22,135,000	4	4	4	2	2	2	2	2	3	3	2	3	2	2	2	3	2	3	3	3	3
chr17:22135001	chr17	22,135,001	22,136,000	4	4	4	2	2	2	2	2	3	3	2	3	2	2	2	3	2	3	3	3	3
chr17:22136001	chr17	22,136,001	22,137,000	4	4	4	2	2	2	2	2	3	3	2	3	2	2	2	3	2	3	3	3	3
chr17:22137001	chr17	22,137,001	22,138,000	4	4	4	2	2	2	2	2	3	3	2	3	2	2	2	3	2	3	3	3	3
chr17:22138001	chr17	22,138,001	22,139,000	4	4	4	2	2	2	2	2	3	3	2	3	2	2	2	3	2	3	3	3	3
chr17:22139001	chr17	22,139,001	22,140,000	4	4	4	2	2	2	2	2	3	3	2	3	2	2	2	3	2	3	3	3	3
chr17:22140001	chr17	22,140,001	22,141,000	4	4	4	2	2	2	2	2	3	3	2	3	2	2	2	3	2	3	3	3	3
chr17:22141001	chr17	22,141,001	22,142,000	4	4	4	2	2	2	2	2	3	3	2	3	2	2	2	3	2	3	3	3	3
chr17:22142001	chr17	22,142,001	22,143,000	4	4	4	2	2	2	2	2	3	3	2	3	2	2	2	3	2	3	3	3	3
chr17:22143001	chr17	22,143,001	22,144,000	4	4	4	2	2	2	2	2	3	3	2	3	2	2	2	3	2	3	3	3	3
chr17:22144001	chr17	22,144,001	22,145,000	4	4	4	2	2	2	2	2	3	3	2	3	2	2	2	3	2	3	3	3	3
chr17:22145001	chr17	22,145,001	22,146,000	4	4	4	2	2	2	2	2	3	3	2	3	2	2	2	3	2	3	3	3	3
chr17:22146001	chr17	22,146,001	22,147,000	4	4	4	2	2	2	2	2	3	3	2	3	2	2	2	3	2	3	3	3	3
chr21:5312001	chr21	5,312,001	5,313,000	3	4	4	2	2	2	2	2	3	3	2	3	2	2	3	4	2	3	2	2	3
chr21:5313001	chr21	5,313,001	5,314,000	3	4	4	2	2	2	2	2	3	3	2	3	2	2	3	4	2	3	2	2	3
chr21:5314001	chr21	5,314,001	5,315,000	3	4	4	2	2	2	2	2	3	3	2	3	2	2	3	4	2	3	2	2	3
chr21:5315001	chr21	5,315,001	5,316,000	3	4	4	2	2	2	2	2	3	3	2	3	2	2	3	4	2	3	2	2	3
chr21:5316001	chr21	5,316,001	5,317,000	3	4	4	2	2	2	2	2	3	3	2	3	2	2	3	4	2	3	2	2	3
chr21:5317001	chr21	5,317,001	5,318,000	3	4	4	2	2	2	2	2	3	3	2	3	2	2	3	4	2	3	2	2	3
chr21:5318001	chr21	5,318,001	5,319,000	3	4	4	2	2	2	2	2	3	3	2	3	2	2	3	4	2	3	2	2	3
chr21:9667001	chr21	9,667,001	9,668,000	4	3	4	2	2	2	2	2	3	3	2	3	2	2	2	3	3	3	3	2	3
chr21:9668001	chr21	9,668,001	9,669,000	4	4	4	2	2	2	2	2	3	3	2	3	2	2	2	3	3	3	3	2	3
chr21:9669001	chr21	9,669,001	9,670,000	4	4	4	2	2	2	2	2	3	3	2	3	2	2	2	3	3	3	3	2	3
chr21:9719001	chr21	9,719,001	9,720,000	4	4	4	3	2	2	2	2	3	3	2	3	2	2	2	3	3	3	3	2	3
chr21:9720001	chr21	9,720,001	9,721,000	4	4	4	3	2	2	2	2	2	3	2	3	2	2	2	3	3	3	3	2	3
chr21:9796001	chr21	9,796,001	9,797,000	4	4	4	2	3	2	2	2	3	3	2	3	2	2	2	3	3	3	2	3	3
chr21:10122001	chr21	10,122,001	10,123,000	4	3	4	2	2	2	2	2	3	3	2	3	2	2	2	3	3	3	3	2	3
chr21:10571001	chr21	10,571,001	10,572,000	4	3	4	2	2	2	2	2	3	2	2	3	2	2	3	3	3	3	3	2	3
chr21:10572001	chr21	10,572,001	10,573,000	4	3	4	2	2	2	2	2	3	2	2	3	2	2	3	3	3	3	3	2	3
chr21:10573001	chr21	10,573,001	10,574,000	4	3	4	2	2	2	2	2	3	2	2	3	2	2	3	3	3	3	3	2	3
chr21:10574001	chr21	10,574,001	10,575,000	4	3	4	2	2	2	2	2	3	2	2	3	2	2	3	3	3	3	3	2	3
chr21:10633001	chr21	10,633,001	10,634,000	4	3	4	2	2	2	2	2	3	2	2	3	2	2	2	3	3	3	3	3	3
chr21:10634001	chr21	10,634,001	10,635,000	4	3	4	2	2	2	2	2	3	2	2	3	2	2	2	3	3	3	3	3	3
chr21:10635001	chr21	10,635,001	10,636,000	4	3	4	2	2	2	2	2	3	2	2	3	2	2	2	3	3	3	3	3	3
chr21:10636001	chr21	10,636,001	10,637,000	4	3	4	2	2	2	2	2	3	2	2	3	2	2	2	3	3	3	3	3	3
chr21:10637001	chr21	10,637,001	10,638,000	4	3	4	2	2	2	2	2	3	2	2	3	2	2	2	3	3	3	3	3	3
chr21:10638001	chr21	10,638,001	10,639,000	4	3	4	2	2	2	2	2	3	2	2	3	2	2	2	3	3	3	3	3	3
chr21:10639001	chr21	10,639,001	10,640,000	4	3	4	2	2	2	2	2	3	2	2	3	2	2	2	3	3	3	3	3	3
chr21:10640001	chr21	10,640,001	10,641,000	4	3	4	2	2	2	2	2	3	2	2	3	2	2	2	3	3	3	3	3	3
chr21:10641001	chr21	10,641,001	10,642,000	4	3	4	2	2	2	2	2	3	2	2	3	2	2	2	3	3	3	3	3	3
chr21:10642001	chr21	10,642,001	10,643,000	4	3	4	2	2	2	2	2	3	2	2	3	2	2	2	3	3	3	3	3	3
chr21:10643001	chr21	10,643,001	10,644,000	4	3	4	2	2	2	2	2	3	2	2	3	2	2	2	3	3	3	3	3	3
chr21:10644001	chr21	10,644,001	10,645,000	4	3	4	2	2	2	2	2	3	2	2	3	2	2	2	3	3	3	3	3	3
chr21:10645001	chr21	10,645,001	10,646,000	4	3	4	2	2	2	2	2	3	2	2	3	2	2	2	3	3	3	3	3	3
chr21:10646001	chr21	10,646,001	10,647,000	4	3	4	2	2	2	2	2	3	2	2	3	2	2	2	3	3	3	3	3	3
chr21:10647001	chr21	10,647,001	10,648,000	4	3	4	2	2	2	2	2	2	2	2	3	2	2	2	3	3	3	3	3	3
chr21:10648001	chr21	10,648,001	10,649,000	4	3	4	2	2	2	2	2	2	2	2	3	2	2	2	3	3	3	3	3	3
chr21:10649001	chr21	10,649,001	10,650,000	3	3	4	2	2	2	2	2	2	2	2	3	2	2	2	3	3	3	3	3	3
chr1:103598001	chr1	103,598,001	103,599,000	4	4	3	2	3	2	2	2	3	2	3	3	2	3	2	3	3	3	2	2	3
chr1:103599001	chr1	103,599,001	103,600,000	4	4	3	2	3	2	2	2	3	2	3	3	2	3	2	3	3	3	2	2	3
chr1:103600001	chr1	103,600,001	103,601,000	4	4	3	2	3	2	2	2	3	2	3	3	2	3	2	3	3	3	2	2	3
chr1:103601001	chr1	103,601,001	103,602,000	4	4	3	2	3	2	2	2	3	2	3	3	2	3	2	3	3	3	2	2	3
chr1:103652001	chr1	103,652,001	103,653,000	4	5	3	2	3	2	1	2	3	2	3	4	2	3	2	2	3	3	2	2	3
chr1:103721001	chr1	103,721,001	103,722,000	4	4	3	2	3	2	2	2	3	2	3	3	2	3	3	2	3	3	2	2	3
chr1:103722001	chr1	103,722,001	103,723,000	4	4	3	2	3	2	2	2	3	2	3	3	2	3	3	2	3	3	2	2	3
chr6:168841001	chr6	168,841,001	168,842,000	5	5	3	2	2	2	2	2	1	5	3	4	2	2	2	3	2	2	2	2	2
chr9:39767001	chr9	39,767,001	39,768,000	3	3	3	2	2	2	2	2	2	3	3	3	2	3	2	3	2	2	3	2	3
chr9:39768001	chr9	39,768,001	39,769,000	3	3	3	2	2	2	2	2	2	3	3	3	2	3	2	3	2	2	3	2	3
chr9:39769001	chr9	39,769,001	39,770,000	3	3	3	2	2	2	2	2	2	3	3	3	2	3	2	3	2	2	3	2	3
chr9:39773001	chr9	39,773,001	39,774,000	3	3	3	2	2	2	2	2	2	3	3	3	2	3	2	3	2	2	3	3	2
chr9:39774001	chr9	39,774,001	39,775,000	3	3	3	2	2	2	2	2	2	3	3	3	2	3	2	3	2	2	3	3	2

Region	CHR	start	end	HD2	HD3	HD1	BN1	BN2	BN3	BN4	BN5	BN6	LC1	LC2	LC3	LC4	LC5	LC6	LC7	LC8	LC9	LC10	LC10*	LC11
chr9:67868001	chr9	67,868,001	67,869,000	3	3	3	2	2	2	2	2	2	3	3	3	2	3	2	3	3	2	3	2	2
chr10:39488001	chr10	39,488,001	39,489,000	4	3	3	2	2	2	2	2	2	3	3	3	2	2	2	3	2	3	3	2	3
chr10:39489001	chr10	39,489,001	39,490,000	4	3	3	2	2	2	2	2	2	3	3	3	2	2	2	3	2	3	3	2	3
chr10:39490001	chr10	39,490,001	39,491,000	3	3	3	2	2	2	2	2	2	3	3	3	2	2	2	3	2	3	3	2	3
chr10:39491001	chr10	39,491,001	39,492,000	3	3	3	2	2	2	2	2	2	3	3	3	2	2	2	3	2	3	3	2	3
chr10:39492001	chr10	39,492,001	39,493,000	3	3	3	2	2	2	2	2	2	3	3	3	2	2	2	3	2	3	3	2	3
chr10:39493001	chr10	39,493,001	39,494,000	3	3	3	2	2	2	2	2	2	3	3	3	2	2	2	3	2	3	3	2	3
chr10:39494001	chr10	39,494,001	39,495,000	3	3	3	2	2	2	2	2	2	3	3	3	2	2	2	3	2	3	3	2	3
chr10:39495001	chr10	39,495,001	39,496,000	3	3	3	2	2	2	2	2	2	3	3	3	2	2	2	3	2	3	3	2	3
chr10:39496001	chr10	39,496,001	39,497,000	3	3	3	2	2	2	2	2	2	3	3	3	2	2	2	3	2	3	3	2	3
chr10:39497001	chr10	39,497,001	39,498,000	3	3	3	2	2	2	2	2	2	3	3	3	2	2	2	3	2	3	3	2	3
chr16:33040001	chr16	33,040,001	33,041,000	3	3	3	2	2	2	2	2	2	2	2	3	3	3	2	3	2	3	3	3	2
chr16:34187001	chr16	34,187,001	34,188,000	4	4	3	3	3	2	2	2	2	3	3	3	3	2	2	3	2	3	2	2	3
chr16:34188001	chr16	34,188,001	34,189,000	4	4	3	3	3	2	2	2	2	3	3	3	3	2	2	3	2	3	2	2	3
chr16:34189001	chr16	34,189,001	34,190,000	4	4	3	3	3	2	2	2	2	3	3	3	3	2	2	3	2	3	2	2	3
chr16:34190001	chr16	34,190,001	34,191,000	4	4	3	3	3	2	2	2	2	3	3	3	3	2	2	3	2	3	2	2	3
chr16:34191001	chr16	34,191,001	34,192,000	4	4	3	3	3	2	2	2	2	3	3	3	3	2	2	3	2	3	2	2	3
chr16:34206001	chr16	34,206,001	34,207,000	4	4	3	3	2	2	2	2	3	3	3	3	2	2	2	3	2	3	3	2	3
chr16:34207001	chr16	34,207,001	34,208,000	4	4	3	3	2	2	2	2	3	3	3	3	2	2	2	3	2	3	3	2	3
chr16:34208001	chr16	34,208,001	34,209,000	4	4	3	3	2	2	2	2	3	3	3	3	2	2	2	3	2	3	3	2	3
chr16:34209001	chr16	34,209,001	34,210,000	4	4	3	3	2	2	2	2	3	3	3	3	2	2	2	3	2	3	3	2	3
chr16:34210001	chr16	34,210,001	34,211,000	4	4	3	3	2	2	2	2	3	3	3	3	2	2	2	3	2	3	3	2	3
chr17:22120001	chr17	22,120,001	22,121,000	3	3	3	2	2	2	2	2	2	3	2	3	2	2	2	3	2	3	3	3	3
chr17:26769001	chr17	26,769,001	26,770,000	3	3	3	2	2	2	2	2	2	3	3	3	2	2	2	3	2	3	3	3	2
chr17:26770001	chr17	26,770,001	26,771,000	3	3	3	2	2	2	2	2	2	3	3	3	2	2	2	3	2	3	3	3	2
chr17:26771001	chr17	26,771,001	26,772,000	3	3	3	2	2	2	2	2	2	3	3	3	2	2	2	3	2	3	3	3	2
chr22:11816001	chr22	11,816,001	11,817,000	3	3	3	2	2	2	2	2	2	3	3	3	2	2	2	2	3	3	3	3	2
chr22:12172001	chr22	12,172,001	12,173,000	3	3	3	2	2	2	2	2	2	3	2	3	2	3	2	2	3	3	3	3	2
chr22:12173001	chr22	12,173,001	12,174,000	3	3	3	2	2	2	2	2	2	3	2	3	2	3	2	2	3	3	3	3	2
chr22:12174001	chr22	12,174,001	12,175,000	3	3	3	2	2	2	2	2	2	3	2	3	2	3	2	2	3	3	3	3	2
chr22:12175001	chr22	12,175,001	12,176,000	3	3	3	2	2	2	2	2	2	3	2	3	2	3	2	2	3	3	3	3	2
chr22:12176001	chr22	12,176,001	12,177,000	3	3	3	2	2	2	2	2	2	3	2	3	2	3	2	2	3	3	3	3	2
chr22:12177001	chr22	12,177,001	12,178,000	3	3	3	2	2	2	2	2	2	3	2	3	2	3	2	2	3	3	3	3	2
chr22:12178001	chr22	12,178,001	12,179,000	3	3	3	2	2	2	2	2	2	3	2	3	2	3	2	2	3	3	3	3	2
chr22:15824001	chr22	15,824,001	15,825,000	3	3	3	2	2	2	2	2	2	3	3	3	2	3	2	3	3	2	2	2	3
chr22:21141001	chr22	21,141,001	21,142,000	3	3	3	2	2	2	2	2	2	2	3	3	3	3	3	3	2	2	2	2	3
chr22:21142001	chr22	21,142,001	21,143,000	3	3	3	2	2	2	2	2	2	2	3	3	3	3	3	3	2	2	2	2	3
chr22:21143001	chr22	21,143,001	21,144,000	3	3	3	2	2	2	2	2	2	2	3	3	3	3	3	3	2	2	2	2	3
chr22:21144001	chr22	21,144,001	21,145,000	3	3	3	2	2	2	2	2	2	2	3	3	3	3	3	3	2	2	2	2	3
chr22:21145001	chr22	21,145,001	21,146,000	3	3	3	2	2	2	2	2	2	2	3	3	3	3	3	3	2	2	2	2	3
chr1:717001	chr1	717,001	718,000	2	2	2	3	3	3	3	3	3	3	2	2	3	3	3	3	3	2	2	3	2
chr1:718001	chr1	718,001	719,000	2	2	2	3	3	3	3	3	3	3	2	2	3	3	3	3	3	2	2	3	2
chr1:727001	chr1	727,001	728,000	2	2	2	3	3	3	3	3	3	2	2	2	3	3	3	3	3	2	2	3	3
chr1:728001	chr1	728,001	729,000	2	2	2	3	3	3	3	3	3	2	2	2	3	3	3	3	3	2	2	3	3
chr1:729001	chr1	729,001	730,000	2	2	2	3	3	3	3	3	3	2	2	2	3	3	3	3	3	2	2	3	3
chr1:742001	chr1	742,001	743,000	2	2	2	3	4	3	3	3	3	2	3	2	3	2	3	3	3	3	2	2	3
chr1:743001	chr1	743,001	744,000	2	2	2	3	4	3	3	3	3	2	3	2	3	2	3	3	3	3	2	2	3
chr1:13240001	chr1	13,240,001	13,241,000	3	2	2	3	4	4	4	3	4	3	3	3	3	2	3	3	3	2	2	2	2
chr16:129001	chr16	129,001	130,000	2	2	2	3	3	3	3	3	3	2	2	2	3	3	3	2	3	3	2	3	3
chr6:170664001	chr6	170,664,001	170,665,000	1	2	1	2	2	2	3	3	2	2	3	2	3	3	3	2	3	3	2	3	2
chr6:170665001	chr6	170,665,001	170,666,000	1	2	1	2	2	2	3	3	2	2	3	2	3	3	3	2	3	3	2	3	2
chr6:170666001	chr6	170,666,001	170,667,000	1	2	1	2	2	2	3	3	2	2	3	2	3	3	3	2	3	3	2	3	2
chr6:170667001	chr6	170,667,001	170,668,000	1	2	1	2	2	2	3	3	2	2	3	2	3	3	3	2	3	3	2	3	2
chr15:101811001	chr15	101,811,001	101,812,000	2	2	1	2	3	4	4	3	3	2	2	3	3	3	3	3	3	2	2	3	2
chr15:101812001	chr15	101,812,001	101,813,000	2	2	1	2	3	4	4	3	3	2	2	3	3	3	3	3	3	2	2	3	2
chr15:101813001	chr15	101,813,001	101,814,000	2	2	1	2	3	4	4	3	3	2	2	3	3	3	3	3	3	2	2	3	2
chr15:101814001	chr15	101,814,001	101,815,000	2	2	1	2	3	4	4	3	3	2	2	3	3	3	3	3	3	2	2	3	2
chr15:101815001	chr15	101,815,001	101,816,000	2	2	1	2	3	4	4	3	3	2	2	3	3	3	3	3	3	2	2	3	2
chr15:101816001	chr15	101,816,001	101,817,000	2	2	1	2	3	4	4	3	3	2	2	3	3	3	3	3	3	2	2	3	2
chr15:101817001	chr15	101,817,001	101,818,000	2	2	1	2	3	4	4	3	3	2	2	3	3	3	3	3	3	2	2	3	2
chr21:13119001	chr21	13,119,001	13,120,000	2	1	1	3	3	2	3	2	2	3	2	3	3	3	2	3	2	3	3	2	2
chr22:15948001	chr22	15,948,001	15,949,000	2	2	1	4	3	4	4	3	3	3	2	4	3	2	2	3	2	2	2	2	4
chr9:43188001	chr9	43,188,001	43,189,000	4	4	5	3	2	2	2	2	3	3	3	3	2	2	2	3	2	3	2	2	3
chr11:124913001	chr11	124,913,001	124,914,000	5	5	5	2	2	2	2	2	2	5	2	2	1	2	2	2	2	2	5	1	4
chr15:19902001	chr15	19,902,001	19,903,000	3	3	5	3	2	2	2	2	2	3	3	3	2	2	2	3	3	2	3	2	2
chr1:125073001	chr1	125,073,001	125,074,000	4	3	4	2	3	2	2	2	3	3	3	3	3	2	2	3	2	3	2	2	2
chr1:125074001	chr1	125,074,001	125,075,000	4	3	4	3	3	2	2	2	3	3	3	3	3	2	2	3	2	3	2	2	2
chr9:63748001	chr9	63,748,001	63,749,000	4	3	4	3	2	2	2	2	3	2	2	3	2	2	2	3	3	3	3	2	3
chr9:63906001	chr9	63,906,001	63,907,000	3	3	4	2	2	2	2	2	2	2	3	3	2	2	2	3	2	3	3	2	3
chr9:63915001	chr9	63,915,001	63,916,000	3	3	4	2	2	2	2	2	2	3	3	2	2	2	2	3	2	3	3	2	3
chr10:39485001	chr10	39,485,001	39,486,000	4	3	4	2	2	2	2	2	2	3	3	3	2	2	2	3	2	3	2	2	3
chr17:21844001	chr17	21,844,001	21,845,000	3	4	4	3	2	2	2	2	2	3	2	3	2	2	2	3	2	3	3	2	3
chr17:22147001	chr17	22,147,001	22,148,000	4	4	4	2	2	2	2	2	3	3	2	3	2	2	2	3	2	3	3	2	3
chr17:22148001	chr17	22,148,001	22,149,000	4	4	4	2	2	2	2	2	3	3	2	3	2	2	2	3	2	3	3	2	3
chr17:22149001	chr17	22,149,001	22,150,000	4	4	4	2	2	2	2	2	3	3	2	3	2	2	2	3	2	3	3	2	3
chr17:22150001	chr17	22,150,001	22,151,000	4	4	4	2	2	2	2	2	3	3	2	3	2	2	2	3	2	3	3	2	3
chr17:22151001	chr17	22,151,001	22,152,000	4	4	4	2	2	2	2	2	3	3	2	3	2	2	2	3	2	3	3	2	3
chr17:22152001	chr17	22,152,001	22,153,000	4	4	4	2	2	2	2	2	3	3	2	3	2	2	2	3	2	3	3	2	3
chr17:22153001	chr17	22,153,001	22,154,000	4	4	4	2	2	2	2	2	3	3	2	3	2	2	2	3	2	3	3	2	3
chr17:22154001	chr17	22,154,001	22,155,000	4	4	4	2	2	2	2	2	3	3	2	3	2	2	2	3	2	3	3	2	3
chr17:22155001	chr17	22,155,001	22,156,000	4	4	4	2	2	2	2	2	3	3	2	3	2	2	2	3	2	3	3	2	3
chr17:22156001	chr17	22,156,001	22,157,000	4	4	4	2	2	2	2	2	3	3	2	3	2	2	2	3	2	3	3	2	3
chr18:15237001	chr18	15,237,001	15,238,000	3	3	4	2	2	2	2	2	2	3	3	3	2	2	2	3	2	3	3	2	2
chr18:15238001	chr18	15,238,001	15,239,000	3	3	4	2	2	2	2	2	2	3	3	3	2	2	2	3	2	3	3	2	2
chr18:15239001	chr18	15,239,001	15,240,000	3	3	4	2	2	2	2	2	2	3	3	3	2	2	2	3	2	3	3	2	2
chr18:15240001	chr18	15,240,001	15,241,000	3	3	4	2	2	2	2	2	2	3	3	3	2	2	2	3	2	3	3	2	2
chr18:15241001	chr18	15,241,001	15,242,000	3	3	4	2	2	2	2	2	2	3	3	3	2	2	2	3	2	3	3	2	2
chr18:15242001	chr18	15,242,001	15,243,000	3	3	4	2	2	2	2	2	2	3	3	3	2	2	2	3	2	3	3	2	2
chr18:15243001	chr18	15,243,001	15,244,000	3	3	4	2	2	2	2	2	2	3	3	3	2	2	2	3	2	3	3	2	2
chr18:15244001	chr18	15,244,001	15,245,000	3	3	4	2	2	2	2	2	2	3	3	3	2	2	2	3	2	3	3	2	2
chr21:5319001	chr21	5,319,001	5,320,000	3	4	4	2	2	2	2	2	3	3	2	3	2	2	2	4	2	3	2	2	3
chr21:5320001	chr21	5,320,001	5,321,000	3	4	4	2	2	2	2	2	3	3	2	3	2	2	2	4	2	3	2	2	3
chr21:5321001	chr21	5,321,001	5,322,000	3	4	4	2	2	2	2	2	3	3	2	3	2	2	2	4	2	3	2	2	3
chr21:5322001	chr21	5,322,001	5,323,000	3	4	4	2	2	2	2	2	3	3	2	3	2	2	2	4	2	3	2	2	3
chr21:5323001	chr21	5,323,001	5,324,000	3	4	4	2	2	2	2	2	3	3	2	3	2	2	2	4	2	3	2	2	3
chr21:9540001	chr21	9,540,001	9,541,000	3	3	4	2	2	2	1	2	2	2	2	3	3	1	2	3	2	3	3	3	3
chr21:9541001	chr21	9,541,001	9,542,000	3	3	4	2	2	2	1	2	2	2	2	3	3	1	2	3	2	3	3	3	3
chr21:9542001	chr21	9,542,001	9,543,000	3	3	4	2	2	2	1	2	2	2	2	3	3	1	2	3	2	3	3	3	3
chr21:9543001	chr21	9,543,001	9,544,000	3	3	4	2	2	2	1	2	2	2	2	3	3	1	2	3	2	3	3	3	3
chr21:9544001	chr21	9,544,001	9,545,000	3	3	4	2	2	2	1	2	2	2	2	3	3	1	2	3	2	3	3	3	3
chr21:9545001	chr21	9,545,001	9,546,000	3	3	4	2	2	2	1	2	2	2	2	3	3	1	2	3	2	3	3	3	3
chr21:9546001	chr21	9,546,001	9,547,000	3	3	4	2	2	2	1	2	2	2	2	3	3	1	2	3	2	3	3	3	3
chr21:9547001	chr21	9,547,001	9,548,000	3	3	4	2	2	2	1	2	2	2	2	3	3	1	2	3	2	3	3	3	3
chr21:9548001	chr21	9,548,001	9,549,000	3	3	4	2	2	2	1	2	2	2	2	3	3	1	2	3	2	3	3	3	3
chr21:9549001	chr21	9,549,001	9,550,000	3	3	4	2	2	2	1	2	2	2	2	3	3	1	2	3	2	3	3	3	3
chr21:9550001	chr21	9,550,001	9,551,000	3	3	4	2	2	2	1	2	2	2	2	3	3	1	2	3	2	3	3	3	3
chr21:9551001	chr21	9,551,001	9,552,000	3	3	4	2	2	2	1	2	2	2	2	3	3	1	2	3	2	3	3	3	3
chr21:9552001	chr21	9,552,001	9,553,000	3	3	4	2	2	2	1	2	2	2	2	3	3	1	2	3	2	3	3	3	3
chr21:9553001	chr21	9,553,001	9,554,000	3	3	4	2	2	2	1	2	2	2	2	3	3	1	2	3	2	3	3	3	3
chr21:9554001	chr21	9,554,001	9,555,000	3	3	4	2	2	2	1	2	2	2	2	3	3	1	2	3	2	3	3	3	3
chr21:9721001	chr21	9,721,001	9,722,000	4	4	4	3	2	2	2	2	2	2	2	3	2	2	2	3	3	3	3	2	3
chr21:9722001	chr21	9,722,001	9,723,000	4	3	4	3	2	2	2	2	2	2	2	3	2	2	2	3	3	3	3	2	3
chr21:10123001	chr21	10,123,001	10,124,000	4	3	4	2	2	2	2	2	3	3	2	3	2	2	2	3	2	3	3	2	3

Region	CHR	start	end	HD2	HD3	HD1	BN1	BN2	BN3	BN4	BN5	BN6	LC1	LC2	LC3	LC4	LC5	LC6	LC7	LC8	LC9	LC10	LC10*	LC11
chr21:10124001	chr21	10,124,001	10,125,000	4	3	4	2	2	2	2	2	3	3	2	3	2	2	2	3	2	3	3	2	3
chr21:10125001	chr21	10,125,001	10,126,000	4	3	4	2	2	2	2	2	3	3	2	3	2	2	2	3	2	3	3	2	3
chr21:10126001	chr21	10,126,001	10,127,000	4	3	4	2	2	2	2	2	3	3	2	3	2	2	2	3	2	3	3	2	3
chr21:10127001	chr21	10,127,001	10,128,000	4	3	4	2	2	2	2	2	3	3	2	3	2	2	2	3	2	3	3	2	3
chr21:10128001	chr21	10,128,001	10,129,000	4	3	4	2	2	2	2	2	3	3	2	3	2	2	2	3	2	3	3	2	3
chr21:10129001	chr21	10,129,001	10,130,000	4	3	4	2	2	2	2	2	3	3	2	3	2	2	2	3	2	3	3	2	3
chr21:10136001	chr21	10,136,001	10,137,000	4	3	4	2	2	2	2	2	2	2	2	3	2	2	2	3	3	3	3	2	3
chr21:10137001	chr21	10,137,001	10,138,000	4	3	4	2	2	2	2	2	2	2	2	3	2	2	2	3	3	3	3	2	3
chr21:10138001	chr21	10,138,001	10,139,000	4	3	4	2	2	2	2	2	2	2	2	3	2	2	2	3	3	3	3	2	3
chr21:10139001	chr21	10,139,001	10,140,000	3	3	4	2	2	2	2	2	2	2	2	3	2	2	2	3	3	3	3	2	3
chr21:10570001	chr21	10,570,001	10,571,000	4	3	4	2	2	2	2	2	3	2	2	3	2	2	3	3	3	3	2	2	3
chr1:103595001	chr1	103,595,001	103,596,000	4	4	3	2	3	2	2	2	2	2	3	3	2	3	2	2	3	3	2	2	3
chr1:103596001	chr1	103,596,001	103,597,000	4	4	3	2	3	2	2	2	3	2	3	3	2	3	2	2	3	3	2	2	3
chr1:103597001	chr1	103,597,001	103,598,000	4	4	3	2	3	2	2	2	3	2	3	3	2	3	2	2	3	3	2	2	3
chr1:103626001	chr1	103,626,001	103,627,000	4	4	3	2	3	2	2	2	3	2	3	3	2	3	2	2	3	3	2	2	3
chr1:103627001	chr1	103,627,001	103,628,000	4	4	3	2	3	2	2	2	3	2	3	3	2	3	2	2	3	3	2	2	3
chr1:103628001	chr1	103,628,001	103,629,000	4	4	3	2	3	2	2	2	3	2	3	3	2	3	2	2	3	3	2	2	3
chr1:103629001	chr1	103,629,001	103,630,000	4	4	3	2	3	2	2	2	3	2	3	3	2	3	2	2	3	3	2	2	3
chr1:103630001	chr1	103,630,001	103,631,000	4	4	3	2	3	2	1	2	3	2	3	3	2	3	2	2	3	3	2	2	3
chr1:103631001	chr1	103,631,001	103,632,000	4	4	3	2	2	2	1	2	3	2	3	3	2	3	2	2	3	3	2	2	3
chr1:103632001	chr1	103,632,001	103,633,000	4	4	3	2	2	2	1	2	3	2	3	3	2	3	2	2	3	3	2	2	3
chr1:103633001	chr1	103,633,001	103,634,000	4	4	3	2	2	2	1	2	3	2	3	3	2	3	2	2	3	3	2	2	3
chr1:103634001	chr1	103,634,001	103,635,000	4	4	3	2	2	2	1	2	3	2	3	3	2	3	2	2	3	3	2	2	3
chr1:103635001	chr1	103,635,001	103,636,000	4	4	3	2	2	2	1	2	3	2	3	3	2	3	2	2	3	3	2	2	3
chr1:103636001	chr1	103,636,001	103,637,000	4	5	3	2	3	2	1	2	3	2	3	3	2	3	2	2	3	3	2	2	3
chr1:103637001	chr1	103,637,001	103,638,000	4	5	3	2	3	2	1	2	3	2	3	3	2	3	2	2	3	3	2	2	3
chr1:103638001	chr1	103,638,001	103,639,000	4	5	3	2	3	2	1	2	3	2	3	3	2	3	2	2	3	3	2	2	3
chr1:103639001	chr1	103,639,001	103,640,000	4	5	3	2	3	2	1	2	3	2	3	3	2	3	2	2	3	3	2	2	3
chr1:103640001	chr1	103,640,001	103,641,000	4	5	3	2	3	2	1	2	3	2	3	4	2	3	2	2	3	2	2	2	3
chr1:103641001	chr1	103,641,001	103,642,000	4	5	3	2	3	2	1	2	3	2	3	4	2	3	2	2	3	2	2	2	3
chr1:103642001	chr1	103,642,001	103,643,000	4	5	3	2	3	2	1	2	3	2	3	4	2	3	2	2	3	2	2	2	3
chr1:103645001	chr1	103,645,001	103,646,000	4	5	3	2	3	2	1	2	3	2	3	4	2	3	2	2	3	2	2	2	3
chr1:103646001	chr1	103,646,001	103,647,000	4	5	3	2	3	2	1	2	3	2	3	4	2	3	2	2	3	2	2	2	3
chr1:103647001	chr1	103,647,001	103,648,000	4	5	3	2	3	2	1	2	3	2	3	4	2	3	2	2	3	2	2	2	3
chr1:103648001	chr1	103,648,001	103,649,000	4	5	3	2	3	2	1	2	3	2	3	4	2	3	2	2	3	2	2	2	3
chr1:103649001	chr1	103,649,001	103,650,000	4	5	3	2	3	2	1	2	3	2	3	4	2	3	2	2	3	2	2	2	3
chr1:103650001	chr1	103,650,001	103,651,000	4	5	3	2	3	2	1	2	3	2	3	4	2	3	2	2	3	2	2	2	3
chr1:103651001	chr1	103,651,001	103,652,000	4	5	3	2	3	2	1	2	3	2	3	4	2	3	2	2	3	2	2	2	3
chr1:103704001	chr1	103,704,001	103,705,000	4	5	3	2	3	2	1	2	3	2	3	4	2	3	2	2	3	2	2	2	3
chr1:103705001	chr1	103,705,001	103,706,000	4	5	3	2	3	2	1	2	3	2	3	4	2	3	2	2	3	2	2	2	3
chr1:103706001	chr1	103,706,001	103,707,000	4	5	3	2	3	2	1	2	3	2	3	4	2	3	2	2	3	2	2	2	3
chr1:103707001	chr1	103,707,001	103,708,000	4	5	3	2	3	2	1	2	3	2	3	4	2	3	2	2	3	2	2	2	3
chr1:103708001	chr1	103,708,001	103,709,000	4	5	3	2	3	2	1	2	3	2	3	4	2	3	2	2	3	2	2	2	3
chr1:103709001	chr1	103,709,001	103,710,000	4	5	3	2	3	2	1	2	3	2	3	4	2	3	2	2	3	2	2	2	3
chr1:103720001	chr1	103,720,001	103,721,000	4	4	3	2	3	2	2	2	3	2	3	3	2	3	2	2	3	3	2	2	3
chr1:103737001	chr1	103,737,001	103,738,000	4	4	3	2	3	2	2	2	3	2	3	3	2	3	2	2	3	3	2	2	3
chr1:103748001	chr1	103,748,001	103,749,000	4	4	3	2	3	2	2	2	3	2	3	3	2	3	2	2	3	3	2	2	3
chr1:103749001	chr1	103,749,001	103,750,000	4	4	3	2	3	2	2	2	3	2	3	3	2	3	2	2	3	3	2	2	3
chr1:103750001	chr1	103,750,001	103,751,000	4	4	3	2	3	2	2	2	3	2	3	3	2	3	2	2	3	3	2	2	3
chr1:103751001	chr1	103,751,001	103,752,000	4	4	3	2	3	2	2	2	3	2	3	3	2	3	2	2	3	3	2	2	3
chr1:103752001	chr1	103,752,001	103,753,000	4	4	3	2	3	2	2	2	3	2	3	3	2	3	2	2	3	3	2	2	3
chr1:103759001	chr1	103,759,001	103,760,000	4	4	3	2	2	2	2	2	3	2	3	3	2	3	2	2	3	3	2	2	3
chr1:103760001	chr1	103,760,001	103,761,000	4	4	3	2	2	2	2	2	2	2	3	3	2	3	2	2	3	3	2	2	3
chr1:125109001	chr1	125,109,001	125,110,000	3	3	3	2	2	2	2	2	2	3	3	3	2	2	2	3	2	3	3	2	2
chr1:125110001	chr1	125,110,001	125,111,000	3	3	3	2	2	2	2	2	2	3	3	3	2	2	2	3	2	3	3	2	2
chr1:125111001	chr1	125,111,001	125,112,000	3	3	3	2	2	2	2	2	2	3	3	3	2	2	2	3	2	3	3	2	2
chr9:41635001	chr9	41,635,001	41,636,000	3	3	3	2	2	2	2	2	2	3	3	3	2	3	2	2	2	2	3	3	2
chr9:63256001	chr9	63,256,001	63,257,000	5	4	3	3	3	3	2	3	3	3	3	2	2	3	2	3	4	2	2	2	2
chr9:63264001	chr9	63,264,001	63,265,000	5	4	3	3	3	3	2	3	3	3	3	2	2	3	2	3	4	2	2	2	2
chr9:63265001	chr9	63,265,001	63,266,000	5	4	3	3	3	3	2	3	3	3	3	2	2	3	2	3	4	2	2	2	2
chr9:63266001	chr9	63,266,001	63,267,000	5	4	3	3	3	3	2	3	3	3	3	2	2	3	2	3	4	2	2	2	2
chr9:63270001	chr9	63,270,001	63,271,000	5	4	3	3	3	3	2	3	3	3	3	2	2	3	2	3	4	2	2	2	2
chr9:67845001	chr9	67,845,001	67,846,000	3	3	3	2	2	2	2	2	2	3	3	3	2	3	2	3	3	2	2	2	2
chr10:39498001	chr10	39,498,001	39,499,000	3	3	3	2	2	2	2	2	2	3	3	2	2	2	2	3	2	3	3	2	3
chr16:32876001	chr16	32,876,001	32,877,000	3	3	3	2	2	1	2	2	2	3	3	2	2	2	2	3	2	3	3	3	2
chr16:32877001	chr16	32,877,001	32,878,000	3	3	3	2	2	1	2	2	2	3	3	2	2	2	2	3	2	3	3	3	2
chr16:34158001	chr16	34,158,001	34,159,000	4	4	3	3	2	2	2	2	3	3	3	3	2	2	2	3	2	3	2	2	3
chr16:36086001	chr16	36,086,001	36,087,000	3	3	3	2	2	2	2	2	2	3	3	3	2	2	2	3	2	3	3	2	2
chr16:36087001	chr16	36,087,001	36,088,000	3	3	3	2	2	2	2	2	2	3	3	3	2	2	2	3	2	3	3	2	2
chr16:36088001	chr16	36,088,001	36,089,000	3	3	3	2	2	2	2	2	2	3	3	3	2	2	2	3	2	3	3	2	2
chr16:36089001	chr16	36,089,001	36,090,000	3	3	3	2	2	2	2	2	2	3	3	3	2	2	2	3	2	3	3	2	2
chr16:36090001	chr16	36,090,001	36,091,000	3	3	3	2	2	2	2	2	2	3	3	3	2	2	2	3	2	3	3	2	2
chr17:22119001	chr17	22,119,001	22,120,000	3	3	3	2	2	2	2	2	2	3	2	3	2	2	2	3	2	3	3	2	3
chr17:26768001	chr17	26,768,001	26,769,000	3	3	3	2	2	2	2	2	2	3	3	3	2	2	2	3	2	2	3	3	2
chr21:10043001	chr21	10,043,001	10,044,000	4	4	3	2	2	2	2	2	3	2	2	3	2	1	3	3	3	3	3	2	3
chr21:10044001	chr21	10,044,001	10,045,000	4	4	3	2	2	2	2	2	3	2	2	3	2	1	3	3	3	3	3	2	3
chr21:10045001	chr21	10,045,001	10,046,000	4	4	3	2	2	2	2	2	3	2	2	3	2	1	3	3	3	3	3	2	3
chr21:10046001	chr21	10,046,001	10,047,000	4	4	3	2	2	2	2	2	3	2	2	3	2	1	3	3	3	3	3	2	3
chr21:10047001	chr21	10,047,001	10,048,000	4	4	3	2	2	2	2	2	3	2	2	3	2	1	3	3	3	3	3	2	3
chr21:10048001	chr21	10,048,001	10,049,000	4	4	3	2	2	2	2	2	3	2	2	3	2	1	3	3	3	3	3	2	3
chr21:10049001	chr21	10,049,001	10,050,000	4	4	3	2	2	2	2	2	3	2	2	3	2	1	3	3	3	3	3	2	3
chr21:10050001	chr21	10,050,001	10,051,000	4	4	3	2	2	2	2	2	3	2	2	3	2	1	3	3	3	3	3	2	3
chr21:10140001	chr21	10,140,001	10,141,000	3	3	3	2	2	2	2	2	2	2	2	3	2	2	2	3	3	3	3	2	3
chr21:10141001	chr21	10,141,001	10,142,000	3	3	3	2	2	2	2	2	2	2	2	3	2	2	2	3	3	3	3	2	3
chr21:10142001	chr21	10,142,001	10,143,000	3	3	3	2	2	2	2	2	2	2	2	3	2	2	2	3	3	3	3	2	3
chr21:10143001	chr21	10,143,001	10,144,000	3	3	3	2	2	2	2	2	2	2	2	3	2	2	2	3	3	3	3	2	3
chr21:10144001	chr21	10,144,001	10,145,000	3	3	3	2	2	2	2	2	2	2	2	3	2	2	2	3	3	3	3	2	3
chr21:10145001	chr21	10,145,001	10,146,000	3	3	3	2	2	2	2	1	2	2	2	3	2	2	2	3	3	3	3	2	3
chr21:10146001	chr21	10,146,001	10,147,000	3	3	3	2	2	2	2	1	2	2	2	3	2	2	2	3	3	3	3	2	3
chr21:10147001	chr21	10,147,001	10,148,000	3	3	3	2	2	2	2	1	2	2	2	3	2	2	2	3	3	3	3	2	3
chr21:10148001	chr21	10,148,001	10,149,000	3	3	3	2	2	2	2	1	2	2	2	3	2	2	2	3	3	3	3	2	3
chr21:10149001	chr21	10,149,001	10,150,000	3	3	3	2	2	2	2	2	2	2	2	3	2	2	2	3	3	3	3	2	3
chr21:10150001	chr21	10,150,001	10,151,000	3	3	3	2	2	2	2	2	2	2	2	3	2	2	2	3	3	3	3	2	3
chr21:10151001	chr21	10,151,001	10,152,000	3	3	3	2	2	2	2	2	2	2	2	3	2	2	2	3	3	3	3	2	3
chr21:10152001	chr21	10,152,001	10,153,000	3	3	3	2	2	2	2	2	2	2	2	3	2	2	2	3	3	3	3	2	3
chr21:10153001	chr21	10,153,001	10,154,000	3	3	3	2	2	2	2	2	2	2	2	3	2	2	2	3	3	3	3	2	3
chr22:11813001	chr22	11,813,001	11,814,000	3	3	3	2	2	2	2	2	2	3	3	2	2	2	2	2	3	3	3	3	2
chr22:11814001	chr22	11,814,001	11,815,000	3	3	3	2	2	2	2	2	2	3	3	2	2	2	2	2	3	3	3	3	2
chr22:11815001	chr22	11,815,001	11,816,000	3	3	3	2	2	2	2	2	2	3	3	2	2	2	2	2	3	3	3	3	2
chr22:15825001	chr22	15,825,001	15,826,000	3	3	3	2	2	2	2	2	2	3	3	2	2	3	2	3	3	2	2	2	3
chr22:15826001	chr22	15,826,001	15,827,000	3	3	3	2	2	2	2	2	2	3	3	2	2	3	2	3	3	2	2	2	3
chr1:744001	chr1	744,001	745,000	2	2	2	3	4	3	3	3	3	2	3	2	3	2	3	3	3	2	2	2	3
chr1:749001	chr1	749,001	750,000	2	2	2	3	3	3	3	3	3	2	2	2	3	3	3	3	3	2	2	2	3
chr18:80260001	chr18	80,260,001	80,261,000	2	3	2	2	5	4	4	4	4	3	3	2	2	3	2	3	3	3	2	2	2
chr15:101788001	chr15	101,788,001	101,789,000	2	2	1	2	3	4	4	3	3	2	2	3	3	3	3	3	3	2	2	2	2
chr15:101789001	chr15	101,789,001	101,790,000	2	2	1	2	3	4	4	3	3	2	2	3	3	3	3	3	3	2	2	2	2
chr4:130425001	chr4	130,425,001	130,426,000	4	5	5	2	2	2	1	2	2	2	2	2	2	2	2	2	2	2	5	4	2
chr4:189881001	chr4	189,881,001	189,882,000	5	5	5	2	2	2	1	1	3	2	2	2	2	2	2	2	2	2	5	2	4
chr8:143669001	chr8	143,669,001	143,670,000	5	3	5	2	2	2	2	2	2	2	2	2	2	2	2	2	2	3	4	2	4
chr9:43189001	chr9	43,189,001	43,190,000	4	4	5	3	2	2	2	2	3	3	3	3	1	2	2	3	2	3	2	2	3
chr11:50757001	chr11	50,757,001	50,758,000	3	4	5	2	2	2	2	2	3	3	3	3	2	2	2	3	2	3	2	2	2
chr11:50758001	chr11	50,758,001	50,759,000	3	4	5	2	2	2	2	2	3	3	3	3	2	2	2	3	2	3	2	2	2
chr15:19901001	chr15	19,901,001	19,902,000	3	3	5	3	2	2	2	2	2	3	3	3	2	2	2	2	3	2	3	2	2

Region	CHR	start	end	HD2	HD3	HD1	BN1	BN2	BN3	BN4	BN5	BN6	LC1	LC2	LC3	LC4	LC5	LC6	LC7	LC8	LC9	LC10	LC10*	LC11
chr2:91784001	chr2	91,784,001	91,785,000	3	4	4	3	2	2	2	2	2	3	3	3	2	2	2	3	2	3	2	2	2
chr2:91858001	chr2	91,858,001	91,859,000	3	3	4	2	2	2	2	2	2	3	2	3	3	2	2	3	2	3	2	2	2
chr2:91859001	chr2	91,859,001	91,860,000	3	3	4	2	2	2	2	2	2	3	2	3	3	2	2	3	2	3	2	2	2
chr9:60608001	chr9	60,608,001	60,609,000	5	5	4	3	3	2	2	4	4	3	3	4	1	2	2	3	1	2	3	2	3
chr9:63913001	chr9	63,913,001	63,914,000	3	3	4	2	2	2	2	2	2	3	3	2	2	2	2	3	2	3	3	2	2
chr9:63914001	chr9	63,914,001	63,915,000	3	3	4	2	2	2	2	2	2	3	3	2	2	2	2	3	2	3	3	2	2
chr16:33919001	chr16	33,919,001	33,920,000	3	3	4	2	2	2	2	2	2	3	3	2	2	2	2	3	2	3	3	2	2
chr16:33920001	chr16	33,920,001	33,921,000	3	3	4	2	2	2	2	2	2	3	3	2	2	2	2	3	2	3	3	2	2
chr16:33921001	chr16	33,921,001	33,922,000	3	3	4	2	2	2	2	2	2	3	3	2	2	2	2	3	2	3	3	2	2
chr16:33922001	chr16	33,922,001	33,923,000	3	3	4	2	2	2	2	2	2	3	3	2	2	2	2	3	2	3	3	2	2
chr16:33923001	chr16	33,923,001	33,924,000	3	3	4	2	2	2	2	2	2	3	3	2	2	2	2	3	2	3	3	2	2
chr17:21661001	chr17	21,661,001	21,662,000	3	3	4	2	2	2	2	2	2	3	2	3	2	2	2	3	2	3	3	2	2
chr17:21662001	chr17	21,662,001	21,663,000	3	3	4	2	2	2	2	2	2	3	2	3	2	2	2	3	2	3	3	2	2
chr17:21663001	chr17	21,663,001	21,664,000	3	3	4	2	2	2	2	2	2	3	2	3	2	2	2	3	2	3	3	2	2
chr17:21664001	chr17	21,664,001	21,665,000	3	3	4	2	2	2	2	2	2	3	2	3	2	2	2	3	2	3	3	2	2
chr17:21665001	chr17	21,665,001	21,666,000	3	3	4	2	2	2	2	2	2	3	2	3	2	2	2	3	2	3	3	2	2
chr17:21666001	chr17	21,666,001	21,667,000	3	3	4	2	2	2	2	2	2	3	2	3	2	2	2	3	2	3	3	2	2
chr17:22157001	chr17	22,157,001	22,158,000	4	4	4	2	2	2	2	2	3	3	2	3	2	2	2	3	2	3	2	2	3
chr21:5324001	chr21	5,324,001	5,325,000	3	4	4	2	2	2	2	2	3	3	2	2	2	2	2	4	2	3	2	2	3
chr21:9534001	chr21	9,534,001	9,535,000	3	3	4	2	2	2	1	2	2	2	2	3	3	1	2	3	2	3	3	2	3
chr21:9535001	chr21	9,535,001	9,536,000	3	3	4	2	2	2	1	2	2	2	2	3	3	1	2	3	2	3	3	2	3
chr21:9536001	chr21	9,536,001	9,537,000	3	3	4	2	2	2	1	2	2	2	2	3	3	1	2	3	2	3	3	2	3
chr21:9537001	chr21	9,537,001	9,538,000	3	3	4	2	2	2	1	2	2	2	2	3	3	1	2	3	2	3	3	2	3
chr21:9538001	chr21	9,538,001	9,539,000	3	3	4	2	2	2	1	2	2	2	2	3	3	1	2	3	2	3	3	2	3
chr21:9539001	chr21	9,539,001	9,540,000	3	3	4	2	2	2	1	2	2	2	2	3	3	1	2	3	2	3	3	2	3
chr21:9555001	chr21	9,555,001	9,556,000	3	3	4	2	2	2	1	2	2	2	2	3	3	1	2	3	2	3	3	2	3
chr21:9556001	chr21	9,556,001	9,557,000	3	3	4	2	2	2	1	2	2	2	2	3	3	1	2	3	2	3	3	2	3
chr21:9557001	chr21	9,557,001	9,558,000	3	3	4	2	2	2	1	2	2	2	2	3	3	1	2	3	2	3	3	2	3
chr21:9741001	chr21	9,741,001	9,742,000	3	3	4	2	2	2	2	2	2	2	2	3	2	2	2	3	3	3	2	2	3
chr21:9779001	chr21	9,779,001	9,780,000	3	3	4	2	2	2	2	2	2	2	2	3	2	2	2	3	3	3	2	2	3
chr21:9781001	chr21	9,781,001	9,782,000	4	3	4	2	2	2	2	2	3	2	2	3	2	2	2	3	3	3	2	2	3
chr21:9782001	chr21	9,782,001	9,783,000	4	4	4	2	2	2	2	2	3	2	2	3	2	2	2	3	3	3	2	2	3
chr21:9783001	chr21	9,783,001	9,784,000	4	4	4	2	2	2	2	2	3	2	2	3	2	2	2	3	3	3	2	2	3
chr21:9784001	chr21	9,784,001	9,785,000	4	4	4	2	2	2	2	2	3	2	2	3	2	2	2	3	3	3	2	2	3
chr21:9785001	chr21	9,785,001	9,786,000	4	4	4	2	2	2	2	2	3	2	2	3	2	2	2	3	3	3	2	2	3
chr21:9786001	chr21	9,786,001	9,787,000	4	4	4	2	2	2	2	2	3	2	2	3	2	2	2	3	3	3	2	2	3
chr21:9787001	chr21	9,787,001	9,788,000	4	4	4	2	2	2	2	2	3	2	2	3	2	2	2	3	3	3	2	2	3
chr21:9788001	chr21	9,788,001	9,789,000	4	4	4	2	2	2	2	2	3	2	2	3	2	2	2	3	3	3	2	2	3
chr21:9789001	chr21	9,789,001	9,790,000	4	4	4	2	2	2	2	2	3	2	2	3	2	2	2	3	3	3	2	2	3
chr21:9790001	chr21	9,790,001	9,791,000	4	4	4	2	2	2	2	2	3	2	2	3	2	2	2	3	3	3	2	2	3
chr21:9791001	chr21	9,791,001	9,792,000	4	4	4	2	2	2	2	2	3	2	2	3	2	2	2	3	3	3	2	2	3
chr21:9792001	chr21	9,792,001	9,793,000	4	4	4	2	2	2	2	2	3	2	2	3	2	2	2	3	3	3	2	2	3
chr21:9793001	chr21	9,793,001	9,794,000	4	4	4	2	3	2	2	2	3	2	2	3	2	2	2	3	3	3	2	2	3
chr21:9794001	chr21	9,794,001	9,795,000	4	4	4	2	3	2	2	2	3	2	2	3	2	2	2	3	3	3	2	2	3
chr21:9795001	chr21	9,795,001	9,796,000	4	4	4	2	3	2	2	2	3	2	2	3	2	2	2	3	3	3	2	2	3
chr21:10130001	chr21	10,130,001	10,131,000	4	3	4	2	2	2	2	2	3	2	2	3	2	2	2	3	2	3	3	2	3
chr21:10131001	chr21	10,131,001	10,132,000	4	3	4	2	2	2	2	2	3	2	2	3	2	2	2	3	2	3	3	2	3
chr21:10132001	chr21	10,132,001	10,133,000	4	3	4	2	2	2	2	2	2	2	2	3	2	2	2	3	2	3	3	2	3
chr21:10133001	chr21	10,133,001	10,134,000	4	3	4	2	2	2	2	2	2	2	2	3	2	2	2	3	2	3	3	2	3
chr21:10134001	chr21	10,134,001	10,135,000	4	3	4	2	2	2	2	2	2	2	2	3	2	2	2	3	2	3	3	2	3
chr21:10135001	chr21	10,135,001	10,136,000	4	3	4	2	2	2	2	2	2	2	2	3	2	2	2	3	2	3	3	2	3
chr1:103594001	chr1	103,594,001	103,595,000	4	4	3	2	3	2	2	2	2	2	3	3	2	3	2	2	3	2	2	2	3
chr1:103662001	chr1	103,662,001	103,663,000	4	5	3	3	3	2	2	2	3	1	3	3	2	3	2	2	3	3	2	2	3
chr1:103663001	chr1	103,663,001	103,664,000	4	5	3	3	3	2	2	2	3	1	3	3	2	3	2	2	3	3	2	2	3
chr1:103718001	chr1	103,718,001	103,719,000	4	4	3	2	3	2	1	2	3	2	3	3	2	3	2	2	3	2	2	2	3
chr1:103719001	chr1	103,719,001	103,720,000	4	4	3	2	3	2	2	2	3	2	3	3	2	3	2	2	3	2	2	2	3
chr1:103738001	chr1	103,738,001	103,739,000	4	4	3	2	3	2	2	2	3	2	3	3	2	3	2	2	3	2	2	2	3
chr1:103739001	chr1	103,739,001	103,740,000	4	4	3	2	3	2	2	2	3	2	3	3	2	3	2	2	3	2	2	2	3
chr1:103740001	chr1	103,740,001	103,741,000	4	4	3	2	3	2	2	2	3	2	3	3	2	3	2	2	3	2	2	2	3
chr1:103741001	chr1	103,741,001	103,742,000	4	4	3	2	3	2	1	2	3	2	3	3	2	3	2	2	3	2	2	2	3
chr1:103742001	chr1	103,742,001	103,743,000	4	4	3	2	3	2	1	2	3	2	3	3	2	3	2	2	3	2	2	2	3
chr1:103743001	chr1	103,743,001	103,744,000	4	4	3	2	3	2	1	2	3	2	3	3	2	3	2	2	3	2	2	2	3
chr1:103746001	chr1	103,746,001	103,747,000	4	4	3	2	3	2	1	2	3	2	3	3	2	3	2	2	3	2	2	2	3
chr1:103747001	chr1	103,747,001	103,748,000	4	4	3	2	3	2	1	2	3	2	3	3	2	3	2	2	3	2	2	2	3
chr1:125119001	chr1	125,119,001	125,120,000	3	3	3	2	2	2	2	2	2	3	3	3	2	2	2	3	2	2	3	2	2
chr1:125120001	chr1	125,120,001	125,121,000	3	3	3	2	2	2	2	2	2	3	3	3	2	2	2	3	2	2	3	2	2
chr1:125121001	chr1	125,121,001	125,122,000	3	3	3	2	2	2	2	2	2	3	3	3	2	2	2	3	2	2	3	2	2
chr1:125122001	chr1	125,122,001	125,123,000	3	3	3	2	2	2	2	2	2	3	3	3	2	2	2	3	2	2	3	2	2
chr1:125123001	chr1	125,123,001	125,124,000	3	3	3	2	2	2	2	2	2	3	3	3	2	2	2	3	2	2	3	2	2
chr1:125124001	chr1	125,124,001	125,125,000	3	3	3	2	2	2	2	2	2	3	3	3	2	2	2	3	2	2	3	2	2
chr1:125125001	chr1	125,125,001	125,126,000	3	3	3	2	2	2	2	2	2	3	3	3	2	2	2	3	2	2	3	2	2
chr1:125126001	chr1	125,126,001	125,127,000	3	3	3	2	2	2	2	2	2	3	3	3	2	2	2	3	2	2	3	2	2
chr1:125127001	chr1	125,127,001	125,128,000	3	3	3	2	2	2	2	2	2	3	3	3	2	2	2	3	2	2	3	2	2
chr9:39766001	chr9	39,766,001	39,767,000	3	3	3	2	2	2	2	2	2	3	3	3	2	3	2	2	2	2	3	2	2
chr9:63254001	chr9	63,254,001	63,255,000	5	4	3	2	3	3	2	3	3	2	3	2	2	3	2	3	4	2	2	2	2
chr9:63255001	chr9	63,255,001	63,256,000	5	4	3	3	3	3	2	3	3	2	3	2	2	3	2	3	4	2	2	2	2
chr9:67846001	chr9	67,846,001	67,847,000	3	3	3	2	2	2	2	2	2	2	3	3	2	3	2	3	3	2	2	2	2
chr9:67847001	chr9	67,847,001	67,848,000	3	3	3	2	2	2	2	2	2	2	3	3	2	3	2	3	3	2	2	2	2
chr9:67848001	chr9	67,848,001	67,849,000	3	3	3	1	1	2	2	2	2	2	3	3	2	3	2	3	3	2	2	2	2
chr9:67867001	chr9	67,867,001	67,868,000	3	3	3	2	2	2	2	2	2	3	3	2	2	3	2	3	3	2	2	2	2
chr10:39499001	chr10	39,499,001	39,500,000	3	3	3	2	2	2	2	2	2	3	3	2	2	2	2	3	2	3	3	2	2
chr11:50763001	chr11	50,763,001	50,764,000	3	4	3	2	2	2	2	2	2	3	3	3	2	2	2	3	2	3	2	2	2
chr11:50764001	chr11	50,764,001	50,765,000	3	4	3	2	2	2	2	2	2	3	3	3	2	2	2	3	2	3	2	2	2
chr16:32878001	chr16	32,878,001	32,879,000	3	3	3	2	2	2	2	2	2	3	3	2	2	2	2	3	2	2	3	3	2
chr16:32879001	chr16	32,879,001	32,880,000	3	3	3	2	2	2	2	2	2	3	3	2	2	2	2	3	2	2	3	3	2
chr16:33041001	chr16	33,041,001	33,042,000	3	3	3	2	2	2	2	2	2	2	2	3	3	2	2	3	2	3	3	2	2
chr16:33992001	chr16	33,992,001	33,993,000	3	3	3	2	2	2	2	2	1	3	3	2	2	2	2	3	2	3	3	2	2
chr16:33993001	chr16	33,993,001	33,994,000	3	3	3	2	2	2	2	2	2	3	3	2	2	2	2	3	2	3	3	2	2
chr16:33994001	chr16	33,994,001	33,995,000	3	3	3	2	2	2	2	2	2	3	3	2	2	2	2	3	2	3	3	2	2
chr16:33995001	chr16	33,995,001	33,996,000	3	3	3	2	2	2	2	2	2	3	3	2	2	2	2	3	2	3	3	2	2
chr16:36082001	chr16	36,082,001	36,083,000	3	3	3	2	2	2	2	2	2	3	3	3	2	2	2	3	2	3	2	2	2
chr16:36083001	chr16	36,083,001	36,084,000	3	3	3	2	2	2	2	2	2	3	3	3	2	2	2	3	2	3	2	2	2
chr16:36084001	chr16	36,084,001	36,085,000	3	3	3	2	2	2	2	2	2	3	3	3	2	2	2	3	2	3	2	2	2
chr16:36085001	chr16	36,085,001	36,086,000	3	3	3	2	2	2	2	2	2	3	3	3	2	2	2	3	2	3	2	2	2
chr16:36231001	chr16	36,231,001	36,232,000	3	3	3	2	2	2	2	2	2	3	3	3	2	2	2	3	2	3	2	2	2
chr16:36232001	chr16	36,232,001	36,233,000	3	3	3	2	2	2	2	2	2	3	3	3	2	2	2	3	2	3	2	2	2
chr16:36233001	chr16	36,233,001	36,234,000	3	3	3	2	2	2	2	2	2	3	3	3	2	2	2	3	2	3	2	2	2
chr16:36234001	chr16	36,234,001	36,235,000	3	3	3	2	2	2	2	2	2	3	3	3	2	2	2	3	2	3	2	2	2
chr16:36235001	chr16	36,235,001	36,236,000	3	3	3	2	2	2	2	2	2	3	3	3	2	2	2	3	2	3	2	2	2
chr16:36236001	chr16	36,236,001	36,237,000	3	3	3	2	2	2	2	2	2	3	3	3	2	2	2	3	2	3	2	2	2
chr16:36237001	chr16	36,237,001	36,238,000	3	3	3	2	2	2	2	2	2	3	3	3	2	2	2	3	2	3	2	2	2
chr16:36238001	chr16	36,238,001	36,239,000	3	3	3	2	2	2	2	2	2	3	3	3	2	2	2	3	2	3	2	2	2
chr16:36239001	chr16	36,239,001	36,240,000	3	3	3	2	2	2	2	2	2	3	3	3	2	2	2	3	2	3	2	2	2
chr16:36240001	chr16	36,240,001	36,241,000	3	3	3	2	2	2	2	2	2	3	3	3	2	2	2	3	2	3	2	2	2
chr16:36241001	chr16	36,241,001	36,242,000	3	3	3	2	2	2	2	2	2	3	3	3	2	2	2	3	2	3	2	2	2
chr16:36242001	chr16	36,242,001	36,243,000	3	3	3	2	2	2	2	2	2	3	3	3	2	2	2	3	2	3	2	2	2
chr16:36243001	chr16	36,243,001	36,244,000	3	3	3	2	2	2	2	2	2	3	3	3	2	2	2	3	2	3	2	2	2
chr16:36244001	chr16	36,244,001	36,245,000	3	3	3	2	2	2	2	2	2	3	3	3	2	2	2	3	2	3	2	2	2
chr16:36245001	chr16	36,245,001	36,246,000	3	3	3	2	2	2	2	2	2	3	3	3	2	2	2	3	2	3	2	2	2
chr16:36246001	chr16	36,246,001	36,247,000	3	3	3	2	2	2	2	2	2	3	3	3	2	2	2	3	2	3	2	2	2
chr16:36247001	chr16	36,247,001	36,248,000	3	3	3	2	2	2	2	2	2	3	3	3	2	2	2	3	2	3	2	2	2
chr16:36248001	chr16	36,248,001	36,249,000	3	3	3	2	2	2	2	2	2	3	3	3	2	2	2	3	2	3	2	2	2
chr17:21658001	chr17	21,658,001	21,659,000	3	3	3	2	2	2	2	2	2	3	2	3	2	2	2	3	2	3	3	2	2

Region	CHR	start	end	HD2	HD3	HD1	BN1	BN2	BN3	BN4	BN5	BN6	LC1	LC2	LC3	LC4	LC5	LC6	LC7	LC8	LC9	LC10	LC10*	LC11
chr17:21659001	chr17	21,659,001	21,660,000	3	3	3	2	2	2	2	2	2	3	2	3	2	2	2	3	2	3	3	2	2
chr17:21660001	chr17	21,660,001	21,661,000	3	3	3	2	2	2	2	2	2	3	2	3	2	2	2	3	2	3	3	2	2
chr17:21667001	chr17	21,667,001	21,668,000	3	3	3	2	2	2	2	2	2	3	2	3	2	2	2	3	2	3	3	2	2
chr17:21668001	chr17	21,668,001	21,669,000	3	3	3	2	2	2	2	2	2	3	2	3	2	2	2	3	2	3	3	2	2
chr17:21669001	chr17	21,669,001	21,670,000	3	3	3	2	2	2	2	2	2	2	3	3	2	2	2	3	2	3	3	2	2
chr17:26764001	chr17	26,764,001	26,765,000	3	3	3	2	2	2	2	2	2	3	2	3	2	2	2	3	2	2	3	3	2
chr17:26765001	chr17	26,765,001	26,766,000	3	3	3	2	2	2	2	2	2	3	2	3	2	2	2	3	2	2	3	3	2
chr17:26766001	chr17	26,766,001	26,767,000	3	3	3	2	2	2	2	2	2	3	2	3	2	2	2	3	2	2	3	3	2
chr17:26767001	chr17	26,767,001	26,768,000	3	3	3	2	2	2	2	2	2	3	2	3	2	2	2	3	2	2	3	3	2
chr21:9727001	chr21	9,727,001	9,728,000	3	3	3	2	2	2	2	2	2	2	2	3	2	2	2	3	3	3	2	2	3
chr21:9728001	chr21	9,728,001	9,729,000	3	3	3	2	2	2	2	2	2	2	2	3	2	2	2	3	3	3	2	2	3
chr21:9729001	chr21	9,729,001	9,730,000	3	3	3	2	2	2	2	2	2	2	2	3	2	2	2	3	3	3	2	2	3
chr21:9730001	chr21	9,730,001	9,731,000	3	3	3	2	2	2	2	2	2	2	2	3	2	2	2	3	3	3	2	2	3
chr21:9731001	chr21	9,731,001	9,732,000	3	3	3	2	2	2	2	2	2	2	2	3	2	2	2	3	3	3	2	2	3
chr21:9732001	chr21	9,732,001	9,733,000	3	3	3	2	2	2	2	2	2	2	2	3	2	2	2	3	3	3	2	2	3
chr21:9733001	chr21	9,733,001	9,734,000	3	3	3	2	2	2	2	2	2	2	2	3	2	2	2	3	3	3	2	2	3
chr21:9734001	chr21	9,734,001	9,735,000	3	3	3	2	2	2	2	2	2	2	2	3	2	2	2	3	3	3	2	2	3
chr21:9735001	chr21	9,735,001	9,736,000	3	3	3	2	2	2	2	2	2	2	2	3	2	2	2	3	3	3	2	2	3
chr21:9736001	chr21	9,736,001	9,737,000	3	3	3	2	2	2	2	2	2	2	2	3	2	2	2	3	3	3	2	2	3
chr21:9737001	chr21	9,737,001	9,738,000	3	3	3	2	2	2	2	2	2	2	2	3	2	2	2	3	3	3	2	2	3
chr21:9738001	chr21	9,738,001	9,739,000	3	3	3	2	2	2	2	2	2	2	2	3	2	2	2	3	3	3	2	2	3
chr21:9739001	chr21	9,739,001	9,740,000	3	3	3	2	2	2	2	2	2	2	2	3	2	2	2	3	3	3	2	2	3
chr21:9740001	chr21	9,740,001	9,741,000	3	3	3	2	2	2	2	2	2	2	2	3	2	2	2	3	3	3	2	2	3
chr21:9989001	chr21	9,989,001	9,990,000	3	3	3	2	2	2	2	1	2	2	2	3	2	2	2	3	3	3	2	2	3
chr21:9990001	chr21	9,990,001	9,991,000	3	3	3	2	2	2	2	1	2	2	2	3	2	2	2	3	3	3	2	2	3
chr21:9991001	chr21	9,991,001	9,992,000	3	3	3	2	2	2	2	1	2	2	2	3	2	2	2	3	3	3	2	2	3
chr21:9992001	chr21	9,992,001	9,993,000	4	3	3	2	2	2	2	1	2	2	2	3	2	2	2	3	3	3	2	2	3
chr21:10077001	chr21	10,077,001	10,078,000	3	3	3	2	2	2	2	2	2	2	2	3	2	2	2	3	3	3	2	2	3
chr21:10078001	chr21	10,078,001	10,079,000	3	3	3	2	2	2	2	2	2	2	2	3	2	2	2	3	3	3	2	2	3
chr21:10079001	chr21	10,079,001	10,080,000	3	3	3	2	2	2	2	2	2	2	2	3	2	2	2	3	3	3	2	2	3
chr21:10080001	chr21	10,080,001	10,081,000	3	3	3	2	2	2	2	2	2	2	2	3	2	2	2	3	3	3	2	2	3
chr21:10094001	chr21	10,094,001	10,095,000	3	3	3	2	2	2	2	2	2	2	2	3	2	2	2	3	3	3	2	2	3
chr21:10095001	chr21	10,095,001	10,096,000	3	3	3	2	2	2	2	2	2	2	2	3	2	2	2	3	3	3	2	2	3
chr21:10096001	chr21	10,096,001	10,097,000	3	3	3	2	2	2	2	2	2	2	2	3	2	2	2	3	3	3	2	2	3
chr21:10097001	chr21	10,097,001	10,098,000	3	3	3	2	2	2	2	2	2	2	2	3	2	2	2	3	3	3	2	2	3
chr21:10098001	chr21	10,098,001	10,099,000	3	3	3	2	2	2	2	2	2	2	2	3	2	2	2	3	3	3	2	2	3
chr21:10099001	chr21	10,099,001	10,100,000	3	3	3	2	2	2	2	2	2	2	2	3	2	2	2	3	3	3	2	2	3
chr22:12179001	chr22	12,179,001	12,180,000	3	3	3	2	2	2	1	2	2	2	2	3	2	3	2	2	2	3	3	3	2
chr22:12180001	chr22	12,180,001	12,181,000	3	3	3	2	2	2	1	2	2	2	2	3	2	3	2	2	2	3	3	3	2
chr1:745001	chr1	745,001	746,000	2	2	2	3	3	3	3	3	3	2	2	2	3	2	3	3	3	2	2	2	3
chr1:746001	chr1	746,001	747,000	2	2	2	3	3	3	3	3	3	2	2	2	3	2	3	3	3	2	2	2	3
chr1:747001	chr1	747,001	748,000	2	2	2	3	3	3	3	3	3	2	2	2	3	2	3	3	3	2	2	2	3
chr1:748001	chr1	748,001	749,000	2	2	2	3	3	3	3	3	3	2	2	2	3	2	3	3	3	2	2	2	3
chr1:750001	chr1	750,001	751,000	2	2	2	3	3	3	3	3	3	2	2	2	3	3	2	3	3	2	2	2	3
chr1:751001	chr1	751,001	752,000	2	2	2	3	3	3	3	3	3	2	2	2	3	3	2	3	3	2	2	2	3
chr1:752001	chr1	752,001	753,000	2	2	2	3	3	3	3	3	3	2	2	2	3	3	2	3	3	2	2	2	3
chr5:181319001	chr5	181,319,001	181,320,000	2	2	2	3	3	3	3	3	3	2	2	2	3	3	2	3	3	2	2	2	3
chr5:181320001	chr5	181,320,001	181,321,000	2	2	2	3	3	3	3	3	3	2	2	2	3	3	2	3	3	2	2	2	3
chr5:181321001	chr5	181,321,001	181,322,000	2	2	2	3	3	3	3	3	3	2	2	2	3	3	2	3	3	2	2	2	3
chr5:181322001	chr5	181,322,001	181,323,000	2	2	2	3	3	3	3	3	3	2	2	2	3	3	2	3	3	2	2	2	3
chr5:181323001	chr5	181,323,001	181,324,000	2	2	2	3	3	3	3	3	3	2	2	2	3	3	2	3	3	2	2	2	3
chr5:181324001	chr5	181,324,001	181,325,000	2	2	2	3	3	3	3	3	3	2	2	2	3	3	2	3	3	2	2	2	3
chr5:181325001	chr5	181,325,001	181,326,000	2	2	2	3	3	3	3	3	3	2	2	2	3	3	2	3	3	2	2	2	3
chr8:7396001	chr8	7,396,001	7,397,000	1	1	2	3	2	3	3	2	3	2	3	3	3	2	2	2	2	2	3	3	2
chr8:7397001	chr8	7,397,001	7,398,000	1	1	2	3	2	3	3	2	3	2	3	3	3	2	2	2	2	2	3	3	2
chr6:170636001	chr6	170,636,001	170,637,000	1	1	1	2	2	2	3	2	2	2	2	2	3	3	3	2	2	3	2	3	2
chr6:170637001	chr6	170,637,001	170,638,000	1	1	1	2	2	2	3	2	2	2	2	2	3	3	3	2	2	3	2	3	2
chr15:101792001	chr15	101,792,001	101,793,000	2	2	1	3	3	4	3	3	3	2	2	3	3	3	3	3	2	2	2	2	2
chr5:2145001	chr5	2,145,001	2,146,000	5	4	5	3	2	2	2	2	2	2	3	2	2	2	1	3	3	2	4	2	2
chr5:2146001	chr5	2,146,001	2,147,000	5	4	5	3	2	2	2	2	2	2	3	2	2	2	1	3	3	2	4	2	2
chr7:138612001	chr7	138,612,001	138,613,000	2	5	5	2	2	2	2	2	2	2	2	2	2	2	2	2	2	2	4	4	2
chr11:50756001	chr11	50,756,001	50,757,000	3	4	5	2	2	2	2	2	3	2	3	3	2	2	2	3	2	3	2	2	2
chr19:15933001	chr19	15,933,001	15,934,000	2	5	5	2	2	2	1	2	2	2	2	4	4	2	2	2	2	2	2	2	2
chr1:125068001	chr1	125,068,001	125,069,000	3	3	4	2	2	2	2	2	3	2	3	3	2	2	2	3	2	3	2	2	2
chr2:89042001	chr2	89,042,001	89,043,000	3	3	4	2	2	2	2	2	2	3	3	2	2	2	2	3	2	3	2	2	2
chr2:89043001	chr2	89,043,001	89,044,000	3	3	4	2	2	2	2	2	2	3	3	2	2	2	2	3	2	3	2	2	2
chr2:89044001	chr2	89,044,001	89,045,000	3	3	4	2	2	2	2	2	2	3	3	2	2	2	2	3	2	3	2	2	2
chr2:89045001	chr2	89,045,001	89,046,000	3	3	4	2	2	2	2	2	2	3	3	2	2	2	2	3	2	3	2	2	2
chr4:189307001	chr4	189,307,001	189,308,000	4	5	4	2	2	2	2	2	4	2	2	2	2	2	2	2	2	5	2	2	3
chr7:58104001	chr7	58,104,001	58,105,000	3	3	4	2	2	2	2	2	3	3	3	3	2	2	2	2	2	3	2	2	2
chr9:63747001	chr9	63,747,001	63,748,000	4	2	4	2	2	2	2	2	2	2	2	3	2	2	2	2	3	2	3	2	3
chr9:63907001	chr9	63,907,001	63,908,000	3	3	4	2	2	2	2	2	2	2	3	3	2	2	2	2	2	3	3	2	2
chr9:65386001	chr9	65,386,001	65,387,000	3	3	4	2	2	2	1	2	2	2	2	3	2	3	2	3	2	2	3	2	2
chr9:65387001	chr9	65,387,001	65,388,000	3	3	4	2	2	2	1	2	2	2	2	3	2	3	2	3	2	2	3	2	2
chr9:65388001	chr9	65,388,001	65,389,000	3	3	4	2	2	2	3	2	2	2	2	3	2	3	2	3	2	2	3	2	2
chr9:65389001	chr9	65,389,001	65,390,000	3	3	4	2	2	2	1	2	2	2	2	3	2	3	2	3	2	2	3	2	2
chr9:65390001	chr9	65,390,001	65,391,000	3	3	4	2	2	2	1	2	2	2	2	3	2	3	2	3	2	2	3	2	2
chr9:65391001	chr9	65,391,001	65,392,000	3	3	4	2	2	2	1	2	2	2	2	3	2	3	2	3	2	2	3	2	2
chr9:65392001	chr9	65,392,001	65,393,000	3	3	4	2	2	2	1	2	2	2	2	3	2	3	2	3	2	2	3	2	2
chr9:65393001	chr9	65,393,001	65,394,000	3	3	4	2	2	2	1	2	2	2	2	3	2	3	2	3	2	2	3	2	2
chr9:65394001	chr9	65,394,001	65,395,000	3	3	4	2	2	2	1	2	2	2	2	3	2	3	2	3	2	2	3	2	2
chr9:65395001	chr9	65,395,001	65,396,000	3	3	4	2	2	2	1	2	2	2	2	3	2	3	2	3	2	2	3	2	2
chr9:65396001	chr9	65,396,001	65,397,000	3	3	4	2	2	2	1	2	2	2	2	3	2	3	2	3	2	2	3	2	2
chr10:39426001	chr10	39,426,001	39,427,000	4	3	4	2	2	2	2	2	2	3	3	2	2	2	2	3	2	3	2	2	2
chr10:39427001	chr10	39,427,001	39,428,000	4	3	4	2	2	2	2	2	2	3	3	2	2	2	2	3	2	3	2	2	2
chr13:18511001	chr13	18,511,001	18,512,000	3	4	4	2	2	3	2	2	3	3	3	2	2	2	3	2	2	2	2	2	3
chr13:18512001	chr13	18,512,001	18,513,000	3	4	4	2	2	3	2	2	3	3	3	2	2	2	3	2	2	2	2	2	3
chr13:18513001	chr13	18,513,001	18,514,000	3	4	4	2	2	3	2	3	3	3	3	2	2	2	3	2	2	2	2	2	3
chr13:18514001	chr13	18,514,001	18,515,000	3	4	4	2	2	3	2	3	3	3	3	2	2	2	3	2	2	2	2	2	3
chr13:18515001	chr13	18,515,001	18,516,000	3	4	4	2	2	3	2	3	3	3	3	2	2	2	3	2	2	2	2	2	3
chr13:18516001	chr13	18,516,001	18,517,000	3	4	4	2	2	3	2	3	3	3	3	2	2	2	3	2	2	2	2	2	3
chr13:18517001	chr13	18,517,001	18,518,000	3	4	4	2	2	3	2	3	3	3	3	2	2	2	3	2	2	2	2	2	3
chr13:18518001	chr13	18,518,001	18,519,000	3	4	4	2	2	3	2	3	3	3	3	2	2	2	3	2	2	2	2	2	3
chr13:18519001	chr13	18,519,001	18,520,000	3	4	4	2	2	3	2	3	3	3	3	2	2	2	3	2	2	2	2	2	3
chr13:18520001	chr13	18,520,001	18,521,000	3	4	4	2	2	3	2	3	3	3	3	2	2	2	3	2	2	2	2	2	3
chr15:20030001	chr15	20,030,001	20,031,000	4	3	4	3	3	2	2	3	3	3	2	3	2	2	2	3	3	2	2	2	2
chr15:20031001	chr15	20,031,001	20,032,000	4	3	4	3	3	2	2	3	3	3	2	3	2	2	2	3	3	2	2	2	2
chr15:20032001	chr15	20,032,001	20,033,000	4	3	4	3	3	2	2	3	3	3	2	3	2	2	2	3	3	2	2	2	2
chr15:20033001	chr15	20,033,001	20,034,000	4	3	4	3	3	2	2	3	3	3	2	3	2	2	2	3	3	2	2	2	2
chr15:20034001	chr15	20,034,001	20,035,000	4	3	4	3	3	2	2	3	3	3	2	3	2	2	2	3	3	2	2	2	2
chr15:20035001	chr15	20,035,001	20,036,000	4	3	4	3	3	2	2	3	3	3	2	3	2	2	2	3	3	2	2	2	2
chr15:20036001	chr15	20,036,001	20,037,000	4	3	4	3	3	2	2	3	3	3	2	3	2	2	2	3	3	2	2	2	2
chr15:20037001	chr15	20,037,001	20,038,000	4	3	4	3	3	2	2	2	3	3	2	3	2	2	2	3	3	2	2	2	2
chr15:20038001	chr15	20,038,001	20,039,000	4	3	4	3	3	2	2	2	3	3	2	3	2	2	2	3	3	2	2	2	2
chr15:20039001	chr15	20,039,001	20,040,000	4	3	4	3	3	2	2	2	3	3	2	3	2	2	2	3	3	2	2	2	2
chr16:33914001	chr16	33,914,001	33,915,000	3	3	4	2	2	2	2	2	2	3	2	2	2	2	2	3	2	3	3	2	2
chr16:33915001	chr16	33,915,001	33,916,000	3	3	4	2	2	2	2	2	2	3	2	2	2	2	2	3	2	3	3	2	2
chr16:33916001	chr16	33,916,001	33,917,000	3	3	4	2	2	2	2	2	2	3	2	2	2	2	2	3	2	3	3	2	2
chr16:33917001	chr16	33,917,001	33,918,000	3	3	4	2	2	2	2	2	2	3	2	2	2	2	2	3	2	3	3	2	2
chr16:33918001	chr16	33,918,001	33,919,000	3	3	4	2	2	2	2	2	2	3	2	2	2	2	2	3	2	3	3	2	2
chr16:36130001	chr16	36,130,001	36,131,000	3	3	4	2	2	2	2	2	3	3	3	3	2	2	2	3	2	2	2	2	2
chr17:22158001	chr17	22,158,001	22,159,000	4	4	4	2	2	2	2	2	2	2	2	3	2	2	2	3	2	3	2	2	3
chr18:15236001	chr18	15,236,001	15,237,000	3	3	4	2	2	2	2	2	2	3	3	2	2	2	2	2	2	3	3	2	2

Region	CHR	start	end	HD2	HD3	HD1	BN1	BN2	BN3	BN4	BN5	BN6	LC1	LC2	LC3	LC4	LC5	LC6	LC7	LC8	LC9	LC10	LC10*	LC11
chr21:5325001	chr21	5,325,001	5,326,000	3	4	4	2	2	2	2	2	3	3	2	2	2	2	2	3	2	3	2	2	3
chr21:5326001	chr21	5,326,001	5,327,000	3	4	4	2	2	2	2	2	2	3	2	2	2	2	2	3	2	3	2	2	3
chr21:5327001	chr21	5,327,001	5,328,000	3	3	4	2	2	2	2	2	2	3	2	2	2	2	2	3	2	3	2	2	3
chr21:5339001	chr21	5,339,001	5,340,000	3	3	4	2	2	2	2	2	3	2	2	2	2	2	3	3	2	3	2	2	3
chr21:5340001	chr21	5,340,001	5,341,000	3	3	4	2	2	2	2	2	3	2	2	2	2	2	3	3	2	3	2	2	3
chr21:5341001	chr21	5,341,001	5,342,000	3	3	4	2	2	2	2	2	3	2	2	2	2	2	3	3	2	3	2	2	3
chr21:5342001	chr21	5,342,001	5,343,000	3	3	4	2	2	2	2	2	3	2	2	2	2	2	3	3	2	3	2	2	3
chr21:9558001	chr21	9,558,001	9,559,000	3	3	4	2	2	2	1	2	2	2	2	3	2	1	2	3	2	3	3	2	3
chr21:9559001	chr21	9,559,001	9,560,000	3	3	4	2	2	2	1	2	2	2	2	3	2	1	2	3	2	3	3	2	3
chr21:9560001	chr21	9,560,001	9,561,000	3	3	4	2	2	2	1	2	2	2	2	3	2	1	2	3	2	3	3	2	3
chr21:9561001	chr21	9,561,001	9,562,000	3	3	4	2	2	2	1	2	2	2	2	3	2	1	2	3	2	3	3	2	3
chr21:9562001	chr21	9,562,001	9,563,000	3	3	4	2	2	2	1	2	2	2	2	3	2	1	2	3	2	3	3	2	3
chr21:9563001	chr21	9,563,001	9,564,000	3	3	4	2	2	2	1	2	2	2	2	3	2	1	2	3	2	3	3	2	3
chr21:9564001	chr21	9,564,001	9,565,000	3	3	4	2	2	2	1	2	2	2	2	3	2	1	2	3	2	3	3	2	3
chr21:9565001	chr21	9,565,001	9,566,000	3	3	4	2	2	2	1	2	2	2	2	3	2	1	2	3	2	3	3	2	3
chr21:9566001	chr21	9,566,001	9,567,000	3	3	4	2	2	2	1	2	2	2	2	3	2	1	2	3	2	3	3	2	3
chr21:9567001	chr21	9,567,001	9,568,000	3	3	4	2	2	2	1	2	2	2	2	3	2	1	2	3	2	3	3	2	3
chr21:9568001	chr21	9,568,001	9,569,000	3	3	4	2	2	2	1	2	2	2	2	3	2	1	2	3	2	3	3	2	3
chr21:9569001	chr21	9,569,001	9,570,000	3	3	4	2	2	2	1	2	2	2	2	3	2	1	2	3	2	3	3	2	3
chr21:9570001	chr21	9,570,001	9,571,000	3	3	4	2	2	2	1	2	2	2	2	3	2	1	2	3	2	3	3	2	3
chr21:9571001	chr21	9,571,001	9,572,000	3	3	4	2	2	2	1	2	2	2	2	3	2	1	2	3	2	3	3	2	3
chr21:9572001	chr21	9,572,001	9,573,000	3	3	4	2	2	2	1	2	2	2	2	3	2	1	2	3	2	3	3	2	3
chr21:9573001	chr21	9,573,001	9,574,000	3	3	4	2	2	2	1	2	2	2	2	3	2	1	2	3	2	3	3	2	3
chr21:9574001	chr21	9,574,001	9,575,000	3	3	4	2	2	2	2	2	2	2	2	3	2	1	2	3	2	3	3	2	3
chr21:9575001	chr21	9,575,001	9,576,000	3	3	4	2	2	2	2	2	2	2	2	3	2	1	2	3	2	3	3	2	3
chr21:9576001	chr21	9,576,001	9,577,000	3	3	4	2	2	2	2	2	2	2	2	3	2	1	2	3	2	3	3	2	3
chr21:9577001	chr21	9,577,001	9,578,000	3	3	4	2	2	2	2	2	2	2	2	3	2	1	2	3	2	3	3	2	3
chr21:9578001	chr21	9,578,001	9,579,000	3	3	4	2	2	2	2	2	2	2	2	3	2	1	2	3	2	3	3	2	3
chr21:9585001	chr21	9,585,001	9,586,000	3	3	4	2	2	2	2	2	2	2	2	3	2	1	2	3	2	3	3	2	3
chr21:9586001	chr21	9,586,001	9,587,000	3	3	4	2	2	2	1	2	2	2	2	3	2	1	2	3	2	3	3	2	3
chr21:9587001	chr21	9,587,001	9,588,000	3	3	4	2	2	2	1	2	2	2	2	3	2	1	2	3	2	3	3	2	3
chr21:9588001	chr21	9,588,001	9,589,000	3	3	4	2	2	2	1	2	2	2	2	3	2	1	2	3	2	3	3	2	3
chr21:9589001	chr21	9,589,001	9,590,000	3	3	4	2	2	2	1	2	2	2	2	3	2	1	2	3	2	3	3	2	3
chr21:9590001	chr21	9,590,001	9,591,000	3	3	4	2	2	2	1	2	2	2	2	3	2	1	2	3	2	3	3	2	3
chr21:9591001	chr21	9,591,001	9,592,000	3	3	4	2	2	2	1	2	2	2	2	3	2	1	2	3	2	3	3	2	3
chr21:9592001	chr21	9,592,001	9,593,000	3	3	4	2	2	2	1	2	2	2	2	3	2	1	2	3	2	3	3	2	3
chr21:9593001	chr21	9,593,001	9,594,000	3	3	4	2	2	2	1	2	2	2	2	3	2	1	2	3	2	3	3	2	3
chr21:9594001	chr21	9,594,001	9,595,000	3	3	4	2	2	2	1	2	2	2	2	3	2	1	2	3	2	3	3	2	3
chr21:9595001	chr21	9,595,001	9,596,000	3	3	4	2	2	2	1	2	2	2	2	3	2	1	2	3	2	3	3	2	3
chr21:9596001	chr21	9,596,001	9,597,000	3	3	4	2	2	2	1	2	2	2	2	3	2	1	2	3	2	3	3	2	3
chr21:9597001	chr21	9,597,001	9,598,000	3	3	4	2	2	2	1	2	2	2	2	3	2	1	2	3	2	3	3	2	3
chr21:9598001	chr21	9,598,001	9,599,000	3	3	4	2	2	2	1	2	2	2	2	3	2	1	2	3	2	3	3	2	3
chr21:9599001	chr21	9,599,001	9,600,000	3	3	4	2	2	2	1	2	2	2	2	3	2	1	2	3	2	3	3	2	3
chr21:9600001	chr21	9,600,001	9,601,000	3	3	4	2	2	2	1	2	2	2	2	3	2	1	2	3	2	3	3	2	3
chr21:9601001	chr21	9,601,001	9,602,000	3	3	4	2	2	2	1	2	2	2	2	3	2	1	2	3	2	3	3	2	3
chr21:9602001	chr21	9,602,001	9,603,000	3	3	4	2	2	2	1	2	2	2	2	3	2	1	2	3	2	3	3	2	3
chr21:9603001	chr21	9,603,001	9,604,000	3	3	4	2	2	2	1	2	2	2	2	3	2	1	2	3	2	3	3	2	3
chr21:9604001	chr21	9,604,001	9,605,000	3	3	4	2	2	2	1	2	2	2	2	3	2	1	2	3	2	3	3	2	3
chr21:9605001	chr21	9,605,001	9,606,000	3	3	4	2	2	2	1	2	2	2	2	3	2	1	2	3	2	3	3	2	3
chr21:9606001	chr21	9,606,001	9,607,000	3	3	4	2	2	2	1	2	2	2	2	3	2	1	2	3	2	3	3	2	3
chr21:9607001	chr21	9,607,001	9,608,000	3	3	4	2	2	2	2	2	2	2	2	3	2	1	2	3	2	3	3	2	3
chr21:9773001	chr21	9,773,001	9,774,000	3	3	4	2	2	2	2	2	3	2	2	3	2	2	2	3	2	3	2	2	3
chr21:9774001	chr21	9,774,001	9,775,000	3	3	4	2	2	2	2	2	3	2	2	3	2	2	2	3	2	3	2	2	3
chr21:9775001	chr21	9,775,001	9,776,000	3	3	4	2	2	2	2	2	3	2	2	3	2	2	2	3	2	3	2	2	3
chr21:9776001	chr21	9,776,001	9,777,000	3	3	4	2	2	2	2	2	3	2	2	3	2	2	2	3	2	3	2	2	3
chr21:9777001	chr21	9,777,001	9,778,000	3	3	4	2	2	2	2	2	2	2	2	3	2	2	2	3	2	3	2	2	3
chr21:9778001	chr21	9,778,001	9,779,000	3	3	4	2	2	2	2	2	2	2	2	3	2	2	2	3	2	3	2	2	3
chr1:103677001	chr1	103,677,001	103,678,000	4	4	3	2	3	2	2	2	3	1	3	3	2	3	2	2	3	2	2	2	3
chr1:103678001	chr1	103,678,001	103,679,000	4	4	3	2	3	2	2	2	3	2	3	3	2	3	2	2	3	2	1	2	3
chr1:103682001	chr1	103,682,001	103,683,000	4	4	3	2	3	2	2	2	3	1	3	3	2	3	2	2	3	2	2	2	3
chr1:103683001	chr1	103,683,001	103,684,000	4	4	3	2	3	2	2	2	3	1	3	3	2	3	2	2	3	2	2	2	3
chr1:103684001	chr1	103,684,001	103,685,000	4	4	3	2	3	2	2	2	3	1	3	3	2	3	2	2	3	2	2	2	3
chr1:103685001	chr1	103,685,001	103,686,000	4	4	3	2	3	2	2	2	3	1	3	3	2	3	2	2	3	2	2	2	3
chr1:125128001	chr1	125,128,001	125,129,000	3	3	3	2	2	2	2	2	2	3	3	2	2	2	2	3	2	2	3	2	2
chr5:70532001	chr5	70,532,001	70,533,000	3	4	3	2	2	2	2	2	3	3	3	2	2	3	2	2	2	3	2	2	2
chr7:57923001	chr7	57,923,001	57,924,000	4	3	3	2	2	2	2	2	3	3	3	2	2	2	2	3	2	3	2	2	2
chr7:57924001	chr7	57,924,001	57,925,000	4	3	3	2	2	2	2	2	3	3	3	2	2	2	2	3	2	3	2	2	2
chr7:57953001	chr7	57,953,001	57,954,000	3	3	3	2	2	2	2	2	2	3	3	3	2	2	2	3	2	2	2	2	2
chr7:57954001	chr7	57,954,001	57,955,000	3	3	3	2	2	2	2	2	2	3	3	3	2	2	2	3	2	2	2	2	2
chr7:57955001	chr7	57,955,001	57,956,000	3	3	3	2	2	2	2	2	2	3	3	3	2	2	2	3	2	2	2	2	2
chr7:57956001	chr7	57,956,001	57,957,000	3	3	3	2	2	2	2	2	2	3	3	3	2	2	2	3	2	2	2	2	2
chr7:57957001	chr7	57,957,001	57,958,000	3	3	3	2	2	2	2	2	2	3	3	3	2	2	2	3	2	2	2	2	2
chr7:57958001	chr7	57,958,001	57,959,000	3	3	3	2	2	2	2	2	2	3	3	3	2	2	2	3	2	2	2	2	2
chr7:57959001	chr7	57,959,001	57,960,000	3	3	3	2	2	2	2	2	2	3	3	3	2	2	2	3	2	2	2	2	2
chr7:57960001	chr7	57,960,001	57,961,000	3	3	3	2	2	2	2	2	2	3	3	3	2	2	2	3	2	2	2	2	2
chr7:57961001	chr7	57,961,001	57,962,000	3	3	3	2	2	2	2	2	2	3	3	3	2	2	2	3	2	2	2	2	2
chr7:63528001	chr7	63,528,001	63,529,000	3	3	3	2	2	2	2	2	2	3	3	3	2	2	2	3	2	2	2	2	2
chr9:39762001	chr9	39,762,001	39,763,000	3	3	3	2	2	2	2	2	2	3	3	3	2	3	2	2	2	2	2	2	2
chr9:39763001	chr9	39,763,001	39,764,000	3	3	3	2	2	2	2	2	2	3	3	3	2	3	2	2	2	2	2	2	2
chr9:39764001	chr9	39,764,001	39,765,000	3	3	3	2	2	2	2	2	2	3	3	3	2	3	2	2	2	2	2	2	2
chr9:39765001	chr9	39,765,001	39,766,000	3	3	3	2	2	2	2	2	2	3	3	3	2	3	2	2	2	2	2	2	2
chr9:60536001	chr9	60,536,001	60,537,000	3	3	3	2	2	2	2	2	2	3	3	3	2	2	2	3	2	2	2	2	2
chr9:60537001	chr9	60,537,001	60,538,000	3	3	3	2	2	2	2	2	2	3	3	3	2	2	2	3	2	2	2	2	2
chr9:60538001	chr9	60,538,001	60,539,000	3	3	3	2	2	2	2	2	2	3	3	3	2	2	2	3	2	2	2	2	2
chr9:60539001	chr9	60,539,001	60,540,000	3	3	3	2	2	2	2	2	2	3	3	3	2	2	2	3	2	2	2	2	2
chr9:60540001	chr9	60,540,001	60,541,000	3	3	3	2	2	2	2	2	2	3	3	3	2	2	2	3	2	2	2	2	2
chr9:60541001	chr9	60,541,001	60,542,000	3	3	3	2	2	2	2	2	2	3	3	3	2	2	2	3	2	2	2	2	2
chr9:60542001	chr9	60,542,001	60,543,000	3	3	3	2	2	2	2	2	2	3	3	3	2	2	2	3	2	2	2	2	2
chr9:67866001	chr9	67,866,001	67,867,000	3	3	3	2	2	2	2	2	2	2	3	2	2	3	2	3	3	2	2	2	2
chr10:39428001	chr10	39,428,001	39,429,000	4	3	3	2	2	2	2	2	2	3	3	2	2	2	2	3	2	3	2	2	2
chr10:39432001	chr10	39,432,001	39,433,000	3	3	3	2	2	2	2	2	2	3	3	2	2	2	2	3	2	3	2	2	2
chr10:39433001	chr10	39,433,001	39,434,000	3	3	3	2	2	2	2	2	2	3	3	2	2	2	2	3	2	3	2	2	2
chr10:39434001	chr10	39,434,001	39,435,000	3	3	3	2	2	2	2	2	2	3	3	2	2	2	2	3	2	3	2	2	2
chr10:39435001	chr10	39,435,001	39,436,000	3	3	3	2	2	2	2	2	2	3	3	2	2	2	2	3	2	3	2	2	2
chr10:39436001	chr10	39,436,001	39,437,000	3	3	3	2	2	2	2	2	2	3	3	2	2	2	2	3	2	3	2	2	2
chr10:39437001	chr10	39,437,001	39,438,000	3	3	3	2	2	2	2	2	2	3	3	2	2	2	2	3	2	3	2	2	2
chr10:39438001	chr10	39,438,001	39,439,000	3	3	3	2	2	2	2	2	2	3	3	2	2	2	2	3	2	3	2	2	2
chr10:39439001	chr10	39,439,001	39,440,000	3	3	3	2	2	2	2	2	2	3	3	2	2	2	2	3	2	3	2	2	2
chr10:39500001	chr10	39,500,001	39,501,000	3	3	3	2	2	2	2	2	2	3	3	2	2	2	2	3	2	3	2	2	2
chr10:39501001	chr10	39,501,001	39,502,000	3	3	3	2	2	2	2	2	2	3	3	2	2	2	2	3	2	3	2	2	2
chr10:42175001	chr10	42,175,001	42,176,000	3	3	3	2	2	2	2	2	2	2	3	2	2	2	2	3	2	3	3	2	2
chr11:50770001	chr11	50,770,001	50,771,000	3	3	3	2	2	2	2	2	2	3	3	2	2	2	2	3	2	3	2	2	2
chr11:50771001	chr11	50,771,001	50,772,000	3	3	3	2	2	2	2	2	2	3	3	2	2	2	2	3	2	3	2	2	2
chr11:50772001	chr11	50,772,001	50,773,000	3	3	3	2	2	2	2	2	2	3	3	2	2	2	2	3	2	3	2	2	2
chr11:50777001	chr11	50,777,001	50,778,000	3	3	3	2	2	2	2	2	2	3	3	3	2	2	2	2	2	3	2	2	2
chr11:50778001	chr11	50,778,001	50,779,000	3	3	3	2	2	2	2	2	2	3	3	3	2	2	2	2	2	3	2	2	2
chr16:34154001	chr16	34,154,001	34,155,000	3	3	3	2	2	2	2	2	2	3	3	3	2	2	2	3	2	2	2	2	2
chr16:34155001	chr16	34,155,001	34,156,000	3	3	3	2	2	2	2	2	2	3	3	3	2	2	2	3	2	2	2	2	2
chr16:34156001	chr16	34,156,001	34,157,000	3	3	3	2	2	2	2	2	2	3	3	3	2	2	2	3	2	2	2	2	2
chr16:35993001	chr16	35,993,001	35,994,000	3	3	3	2	2	2	2	2	2	3	3	2	2	2	2	3	2	3	2	2	2
chr16:35994001	chr16	35,994,001	35,995,000	3	3	3	2	2	2	2	2	2	3	3	2	2	2	2	3	2	3	2	2	2
chr16:35995001	chr16	35,995,001	35,996,000	3	3	3	2	2	2	2	2	2	3	3	2	2	2	2	3	2	3	2	2	2
chr16:36032001	chr16	36,032,001	36,033,000	3	3	3	2	2	2	2	2	2	3	3	3	2	2	2	3	2	2	2	2	2

Region	CHR	start	end	HD2	HD3	HD1	BN1	BN2	BN3	BN4	BN5	BN6	LC1	LC2	LC3	LC4	LC5	LC6	LC7	LC8	LC9	LC10	LC10*	LC11
chr16:36033001	chr16	36,033,001	36,034,000	3	3	3	2	2	2	2	2	2	3	3	3	2	2	2	3	2	2	2	2	2
chr16:36079001	chr16	36,079,001	36,080,000	3	3	3	2	2	2	2	2	2	3	3	2	2	2	2	3	2	3	2	2	2
chr16:36080001	chr16	36,080,001	36,081,000	3	3	3	2	2	2	2	2	2	3	3	2	2	2	2	3	2	3	2	2	2
chr16:36081001	chr16	36,081,001	36,082,000	3	3	3	2	2	2	2	2	2	3	3	2	2	2	2	3	2	3	2	2	2
chr16:36132001	chr16	36,132,001	36,133,000	3	3	3	2	2	2	2	2	2	3	3	3	2	2	2	3	2	2	2	2	2
chr16:36133001	chr16	36,133,001	36,134,000	3	3	3	2	2	2	2	2	2	3	3	3	2	2	2	3	2	2	2	2	2
chr16:36134001	chr16	36,134,001	36,135,000	3	3	3	2	2	2	2	2	2	3	3	3	2	2	2	3	2	2	2	2	2
chr16:36135001	chr16	36,135,001	36,136,000	3	3	3	2	2	2	2	2	2	3	3	3	2	2	2	3	2	2	2	2	2
chr16:36136001	chr16	36,136,001	36,137,000	3	3	3	2	2	2	2	2	2	3	3	3	2	2	2	3	2	2	2	2	2
chr16:36137001	chr16	36,137,001	36,138,000	3	3	3	2	2	2	2	2	2	3	3	3	2	2	2	3	2	2	2	2	2
chr16:36169001	chr16	36,169,001	36,170,000	3	3	3	2	2	2	2	2	2	3	3	3	2	2	2	3	2	2	2	2	2
chr16:36170001	chr16	36,170,001	36,171,000	3	3	3	2	2	2	2	2	2	3	3	3	2	2	2	3	2	2	2	2	2
chr16:36171001	chr16	36,171,001	36,172,000	3	3	3	2	2	2	2	2	2	3	3	3	2	2	2	3	2	2	2	2	2
chr16:36176001	chr16	36,176,001	36,177,000	3	3	3	2	2	2	2	2	2	3	3	3	2	2	2	3	2	2	2	2	2
chr16:36177001	chr16	36,177,001	36,178,000	3	3	3	2	2	2	2	2	2	3	3	3	2	2	2	3	2	2	2	2	2
chr16:36178001	chr16	36,178,001	36,179,000	3	3	3	2	2	2	2	2	2	3	3	3	2	2	2	3	2	2	2	2	2
chr16:36179001	chr16	36,179,001	36,180,000	3	3	3	2	2	2	2	2	2	3	3	3	2	2	2	3	2	2	2	2	2
chr16:36183001	chr16	36,183,001	36,184,000	3	3	3	2	2	2	2	2	2	3	2	3	2	2	2	3	2	3	2	2	2
chr16:36184001	chr16	36,184,001	36,185,000	3	3	3	2	2	2	2	2	2	3	2	3	2	2	2	3	2	3	2	2	2
chr16:36185001	chr16	36,185,001	36,186,000	3	3	3	2	2	2	2	2	2	3	2	3	2	2	2	3	2	3	2	2	2
chr16:36186001	chr16	36,186,001	36,187,000	3	3	3	2	2	2	2	2	2	3	2	3	2	2	2	3	2	3	2	2	2
chr16:36187001	chr16	36,187,001	36,188,000	3	3	3	2	2	2	2	2	2	3	2	3	2	2	2	3	2	3	2	2	2
chr16:36188001	chr16	36,188,001	36,189,000	3	3	3	2	2	2	2	2	2	3	2	3	2	2	2	3	2	3	2	2	2
chr16:36189001	chr16	36,189,001	36,190,000	3	3	3	2	2	2	2	2	2	3	2	3	2	2	2	3	2	3	2	2	2
chr16:36190001	chr16	36,190,001	36,191,000	3	3	3	2	2	2	2	2	2	3	2	3	2	2	2	3	2	3	2	2	2
chr16:36191001	chr16	36,191,001	36,192,000	3	3	3	2	2	2	2	2	2	3	2	3	2	2	2	3	2	3	2	2	2
chr16:36192001	chr16	36,192,001	36,193,000	3	3	3	2	2	2	2	2	2	3	2	3	2	2	2	3	2	3	2	2	2
chr16:36193001	chr16	36,193,001	36,194,000	3	3	3	2	2	2	2	2	2	3	2	3	2	2	2	3	2	3	2	2	2
chr16:36194001	chr16	36,194,001	36,195,000	3	3	3	2	2	2	2	2	2	3	2	3	2	2	2	3	2	3	2	2	2
chr16:36195001	chr16	36,195,001	36,196,000	3	3	3	2	2	2	2	2	2	3	2	3	2	2	2	3	2	3	2	2	2
chr16:36229001	chr16	36,229,001	36,230,000	3	3	3	2	2	2	2	2	2	3	2	3	2	2	2	3	2	3	2	2	2
chr16:36230001	chr16	36,230,001	36,231,000	3	3	3	2	2	2	2	2	2	3	2	3	2	2	2	3	2	3	2	2	2
chr16:36249001	chr16	36,249,001	36,250,000	3	3	3	2	2	2	2	2	2	3	3	3	2	2	2	3	2	2	2	2	2
chr16:36250001	chr16	36,250,001	36,251,000	3	3	3	2	2	2	2	2	2	3	3	3	2	2	2	3	2	2	2	2	2
chr17:21654001	chr17	21,654,001	21,655,000	3	3	3	2	2	2	2	2	2	3	2	3	2	2	2	3	2	3	2	2	2
chr17:21655001	chr17	21,655,001	21,656,000	3	3	3	2	2	2	2	2	2	3	2	3	2	2	2	3	2	3	2	2	2
chr17:21656001	chr17	21,656,001	21,657,000	3	3	3	2	2	2	2	2	2	3	2	3	2	2	2	3	2	3	2	2	2
chr17:21657001	chr17	21,657,001	21,658,000	3	3	3	2	2	2	2	2	2	3	2	3	2	2	2	3	2	3	2	2	2
chr17:21673001	chr17	21,673,001	21,674,000	3	3	3	2	2	2	2	2	2	2	3	3	2	2	2	3	2	2	3	2	2
chr18:15385001	chr18	15,385,001	15,386,000	3	4	3	2	2	2	2	2	2	3	3	2	2	2	2	3	2	2	2	2	3
chr18:15386001	chr18	15,386,001	15,387,000	3	4	3	2	2	2	2	2	3	3	3	2	2	2	2	3	2	2	2	2	3
chr19:39878001	chr19	39,878,001	39,879,000	3	3	3	2	2	2	2	2	2	2	3	2	3	2	3	2	3	2	2	2	2
chr21:8595001	chr21	8,595,001	8,596,000	3	3	3	2	2	2	2	2	2	3	2	3	2	2	2	3	3	2	2	2	2
chr21:8596001	chr21	8,596,001	8,597,000	3	3	3	2	2	2	2	2	2	3	2	3	2	2	2	3	3	2	2	2	2
chr21:8597001	chr21	8,597,001	8,598,000	3	3	3	2	2	2	2	2	2	3	2	3	2	2	2	3	3	2	2	2	2
chr21:9869001	chr21	9,869,001	9,870,000	3	3	3	2	2	2	2	2	2	2	2	2	2	2	2	3	3	3	2	2	3
chr21:10081001	chr21	10,081,001	10,082,000	3	3	3	2	2	2	2	2	2	2	2	3	2	2	2	3	2	3	2	2	3
chr21:10082001	chr21	10,082,001	10,083,000	3	3	3	2	2	2	2	2	2	2	2	3	2	2	2	3	2	3	2	2	3
chr21:10083001	chr21	10,083,001	10,084,000	3	3	3	2	2	2	2	2	2	2	2	3	2	2	2	3	2	3	2	2	3
chr21:10084001	chr21	10,084,001	10,085,000	3	3	3	2	2	2	2	2	2	2	2	3	2	2	2	3	2	3	2	2	3
chr21:10085001	chr21	10,085,001	10,086,000	3	3	3	2	2	2	2	2	2	2	2	3	2	2	2	3	2	3	2	2	3
chr21:10086001	chr21	10,086,001	10,087,000	3	3	3	2	2	2	2	2	2	2	2	3	2	2	2	3	2	3	2	2	3
chr21:10087001	chr21	10,087,001	10,088,000	3	3	3	2	2	2	2	2	2	2	2	3	2	2	2	3	2	3	2	2	3
chr21:10088001	chr21	10,088,001	10,089,000	3	3	3	2	2	2	2	2	2	2	2	3	2	2	2	3	2	3	2	2	3
chr21:10089001	chr21	10,089,001	10,090,000	3	3	3	2	2	2	2	2	2	2	2	3	2	2	2	3	2	3	2	2	3
chr21:10090001	chr21	10,090,001	10,091,000	3	3	3	2	2	2	2	2	2	2	2	3	2	2	2	3	2	3	2	2	3
chr21:10091001	chr21	10,091,001	10,092,000	3	3	3	2	2	2	2	2	2	2	2	3	2	2	2	3	2	3	2	2	3
chr21:10092001	chr21	10,092,001	10,093,000	3	3	3	2	2	2	2	2	2	2	2	3	2	2	2	3	2	3	2	2	3
chr21:10093001	chr21	10,093,001	10,094,000	3	3	3	2	2	2	2	2	2	2	2	3	2	2	2	3	2	3	2	2	3
chr22:12181001	chr22	12,181,001	12,182,000	3	3	3	2	2	2	1	2	2	2	2	3	2	3	2	2	2	3	3	2	2
chr22:15762001	chr22	15,762,001	15,763,000	3	3	3	2	2	2	2	2	2	2	3	2	2	3	2	3	2	2	2	2	3
chr15:101729001	chr15	101,729,001	101,730,000	2	2	1	2	3	3	3	3	3	2	2	2	3	3	3	3	2	2	2	2	2
chr15:101730001	chr15	101,730,001	101,731,000	2	2	1	2	3	3	3	3	3	2	2	2	3	3	3	3	2	2	2	2	2
chr15:101731001	chr15	101,731,001	101,732,000	2	2	1	2	3	3	3	3	3	2	2	2	3	3	3	3	2	2	2	2	2
chr15:101732001	chr15	101,732,001	101,733,000	2	2	1	2	3	3	3	3	3	2	2	2	3	3	3	3	2	2	2	2	2
chr15:101733001	chr15	101,733,001	101,734,000	2	2	1	2	3	3	3	3	3	2	2	2	3	3	3	3	2	2	2	2	2
chr15:101737001	chr15	101,737,001	101,738,000	2	2	1	2	3	3	3	3	3	3	2	2	2	3	3	3	2	2	2	2	2
chr15:101738001	chr15	101,738,001	101,739,000	2	2	1	2	3	3	3	3	3	3	2	2	2	3	3	3	2	2	2	2	2
chr15:101739001	chr15	101,739,001	101,740,000	2	2	1	2	3	3	3	3	3	3	2	2	2	3	3	3	2	2	2	2	2
chr15:101740001	chr15	101,740,001	101,741,000	2	2	1	2	3	3	3	3	3	3	2	2	2	3	3	3	2	2	2	2	2
chr15:101741001	chr15	101,741,001	101,742,000	2	2	1	2	3	3	3	3	3	3	2	2	2	3	3	3	2	2	2	2	2
chr15:101742001	chr15	101,742,001	101,743,000	2	2	1	2	3	3	3	3	3	3	2	2	2	3	3	3	2	2	2	2	2
chr15:101743001	chr15	101,743,001	101,744,000	2	2	1	2	3	3	3	3	3	3	2	2	2	3	3	3	2	2	2	2	2
chr15:101744001	chr15	101,744,001	101,745,000	2	2	1	2	3	3	3	3	3	3	2	2	2	3	3	3	2	2	2	2	2
chr15:101747001	chr15	101,747,001	101,748,000	2	2	1	2	3	3	3	3	3	2	2	3	2	3	3	3	2	2	2	2	2
chr15:101748001	chr15	101,748,001	101,749,000	2	2	1	2	3	3	3	3	3	2	2	3	2	3	3	3	2	2	2	2	2
chr15:101749001	chr15	101,749,001	101,750,000	2	2	1	2	3	3	3	3	3	2	2	3	2	3	3	3	2	2	2	2	2
chr15:101793001	chr15	101,793,001	101,794,000	2	2	1	3	3	4	3	3	3	2	2	3	3	3	3	2	2	2	2	2	2
chr15:101794001	chr15	101,794,001	101,795,000	2	2	1	2	3	4	3	3	3	2	2	2	3	3	3	2	2	2	2	3	2
chr15:101795001	chr15	101,795,001	101,796,000	2	2	1	2	3	3	3	3	3	2	2	2	3	3	3	2	2	2	2	3	2
chr15:101827001	chr15	101,827,001	101,828,000	2	2	1	2	3	3	3	3	3	2	2	3	3	2	3	2	2	2	2	2	3
chr15:101837001	chr15	101,837,001	101,838,000	2	2	1	2	3	3	3	3	3	2	2	3	2	3	3	3	2	2	2	2	2
chr15:101838001	chr15	101,838,001	101,839,000	2	2	1	2	3	3	3	3	3	2	2	3	2	3	3	3	2	2	2	2	2
chr3:189001	chr3	189,001	190,000	1	1	0	2	2	2	2	0	2	2	2	3	2	2	2	3	3	2	2	3	2
chr3:190001	chr3	190,001	191,000	1	1	0	2	2	2	2	0	2	2	2	3	2	2	2	3	3	2	2	3	2
chr3:191001	chr3	191,001	192,000	1	1	0	2	2	2	2	0	2	2	2	3	2	2	2	3	3	2	2	3	2
chr1:240184001	chr1	240,184,001	240,185,000	4	2	5	2	2	2	2	2	2	3	2	2	2	2	2	2	2	2	4	2	2
chr2:5022001	chr2	5,022,001	5,023,000	5	5	5	2	2	2	2	2	2	2	5	2	2	2	2	2	2	2	2	2	2
chr7:63519001	chr7	63,519,001	63,520,000	3	3	5	3	2	3	2	2	3	2	3	2	2	2	2	3	2	2	3	2	2
chr7:125702001	chr7	125,702,001	125,703,000	5	5	5	2	2	2	2	2	2	2	2	2	2	2	2	2	2	4	3	2	2
chr11:48867001	chr11	48,867,001	48,868,000	3	3	5	3	3	2	2	2	2	3	3	2	2	2	2	2	2	3	2	2	2
chr11:48868001	chr11	48,868,001	48,869,000	3	3	5	3	3	2	2	2	2	3	3	2	2	2	2	2	2	3	2	2	2
chr11:48869001	chr11	48,869,001	48,870,000	3	3	5	3	3	2	2	2	2	3	3	2	2	2	2	2	2	3	2	2	2
chr11:48870001	chr11	48,870,001	48,871,000	3	3	5	3	3	2	2	2	2	3	3	2	2	2	2	2	2	3	2	2	2
chr11:48871001	chr11	48,871,001	48,872,000	3	3	5	3	3	2	2	2	2	3	3	2	2	2	2	2	2	3	2	2	2
chr11:48872001	chr11	48,872,001	48,873,000	3	3	5	3	3	2	2	2	2	3	3	2	2	2	2	2	2	3	2	2	2
chr11:48873001	chr11	48,873,001	48,874,000	3	3	5	3	3	2	2	2	2	3	3	2	2	2	2	2	2	3	2	2	2
chr11:48874001	chr11	48,874,001	48,875,000	3	3	5	3	3	2	2	2	2	3	3	2	2	2	2	2	2	3	2	2	2
chr14:34350001	chr14	34,350,001	34,351,000	5	5	5	2	2	2	2	2	2	2	2	2	2	2	2	2	2	2	5	2	2
chr2:89034001	chr2	89,034,001	89,035,000	3	3	4	2	2	2	2	2	3	3	3	2	2	2	2	3	2	2	2	2	2
chr2:89035001	chr2	89,035,001	89,036,000	3	3	4	2	2	2	2	2	3	3	3	2	2	2	2	3	2	2	2	2	2
chr2:89036001	chr2	89,036,001	89,037,000	3	3	4	3	2	2	2	2	3	3	3	2	2	2	2	3	2	2	2	2	2
chr2:89046001	chr2	89,046,001	89,047,000	3	3	4	2	2	2	2	2	2	2	3	2	2	2	2	3	2	3	2	2	2
chr2:89047001	chr2	89,047,001	89,048,000	3	3	4	2	2	2	2	2	2	2	3	2	2	2	2	3	2	3	2	2	2
chr4:33844001	chr4	33,844,001	33,845,000	3	3	4	2	2	2	2	2	2	2	2	3	2	2	2	3	2	3	2	2	2
chr4:33845001	chr4	33,845,001	33,846,000	3	3	4	2	2	2	2	2	2	2	2	3	2	2	2	3	2	3	2	2	2
chr4:33846001	chr4	33,846,001	33,847,000	3	3	4	2	2	2	2	2	2	2	2	3	2	2	2	3	2	3	2	2	2
chr9:63908001	chr9	63,908,001	63,909,000	3	3	4	2	2	2	2	2	2	2	3	2	2	2	2	2	2	3	3	2	2
chr9:63909001	chr9	63,909,001	63,910,000	3	3	4	2	2	2	2	2	2	2	3	2	2	2	2	2	2	3	3	2	2
chr9:63910001	chr9	63,910,001	63,911,000	3	3	4	2	2	2	2	2	2	2	3	2	2	2	2	2	2	3	3	2	2
chr9:63911001	chr9	63,911,001	63,912,000	3	3	4	2	2	2	2	2	2	2	3	2	2	2	2	2	2	3	3	2	2
chr9:63912001	chr9	63,912,001	63,913,000	3	3	4	2	2	2	2	2	2	2	3	2	2	2	2	2	2	3	3	2	2
chr10:39425001	chr10	39,425,001	39,426,000	4	3	4	2	2	2	2	2	2	3	2	2	2	2	2	3	2	3	2	2	2

Region	CHR	start	end	HD2	HD3	HD1	BN1	BN2	BN3	BN4	BN5	BN6	LC1	LC2	LC3	LC4	LC5	LC6	LC7	LC8	LC9	LC10	LC10*	LC11
chr11:48875001	chr11	48,875,001	48,876,000	3	3	4	3	3	2	2	2	2	3	3	2	2	2	2	2	2	3	2	2	2
chr13:18436001	chr13	18,436,001	18,437,000	3	3	4	2	2	3	2	2	2	2	3	2	2	2	3	2	2	2	2	2	3
chr13:18437001	chr13	18,437,001	18,438,000	3	4	4	2	2	3	2	2	2	2	3	2	2	2	3	2	2	2	2	2	3
chr13:18438001	chr13	18,438,001	18,439,000	3	4	4	2	2	3	2	2	2	2	3	2	2	2	3	2	2	2	2	2	3
chr13:18439001	chr13	18,439,001	18,440,000	3	4	4	2	2	3	2	2	2	2	3	2	2	2	3	2	2	2	2	2	3
chr13:18440001	chr13	18,440,001	18,441,000	3	4	4	2	2	3	2	2	3	2	3	2	2	2	3	2	2	2	2	2	3
chr13:18441001	chr13	18,441,001	18,442,000	3	4	4	2	2	3	2	2	3	2	3	2	2	2	3	2	2	2	2	2	3
chr13:18442001	chr13	18,442,001	18,443,000	3	4	4	2	2	3	2	2	3	2	3	2	2	2	3	2	2	2	2	2	3
chr13:18443001	chr13	18,443,001	18,444,000	3	4	4	2	2	3	2	3	3	2	3	2	2	2	3	2	2	2	2	2	3
chr13:18444001	chr13	18,444,001	18,445,000	3	4	4	2	2	3	2	3	3	2	3	2	2	2	3	2	2	2	2	2	3
chr13:18445001	chr13	18,445,001	18,446,000	3	4	4	2	2	3	2	3	3	2	3	2	2	2	3	2	2	2	2	2	3
chr13:18446001	chr13	18,446,001	18,447,000	3	4	4	2	2	3	2	3	3	2	3	2	2	2	3	2	2	2	2	2	3
chr13:18447001	chr13	18,447,001	18,448,000	3	4	4	2	2	3	2	2	3	2	3	2	2	2	3	2	2	2	2	2	3
chr13:18448001	chr13	18,448,001	18,449,000	3	4	4	2	2	3	2	2	3	2	3	2	2	2	3	2	2	2	2	2	3
chr13:18449001	chr13	18,449,001	18,450,000	3	4	4	2	2	3	2	2	3	2	3	2	2	2	3	2	2	2	2	2	3
chr13:18450001	chr13	18,450,001	18,451,000	3	4	4	2	2	3	2	2	3	2	3	2	2	2	3	2	2	2	2	2	3
chr13:18451001	chr13	18,451,001	18,452,000	3	4	4	2	2	3	2	2	3	2	3	2	2	2	3	2	2	2	2	2	3
chr13:18452001	chr13	18,452,001	18,453,000	3	4	4	2	2	3	2	2	3	2	3	2	2	2	3	2	2	2	2	2	3
chr13:18453001	chr13	18,453,001	18,454,000	3	4	4	2	2	3	2	2	3	2	3	2	2	2	3	2	2	2	2	2	3
chr13:18454001	chr13	18,454,001	18,455,000	3	4	4	2	2	3	2	2	3	2	3	2	2	2	3	2	2	2	2	2	3
chr13:18455001	chr13	18,455,001	18,456,000	3	4	4	2	2	3	2	2	3	2	3	2	2	2	3	2	2	2	2	2	3
chr13:18456001	chr13	18,456,001	18,457,000	3	4	4	2	2	3	2	3	3	2	3	2	2	2	3	2	2	2	2	2	3
chr13:18457001	chr13	18,457,001	18,458,000	3	4	4	2	2	3	2	3	3	2	3	2	2	2	3	2	2	2	2	2	3
chr13:18458001	chr13	18,458,001	18,459,000	3	4	4	2	2	2	2	3	3	2	3	2	2	2	3	2	2	2	2	2	3
chr13:18459001	chr13	18,459,001	18,460,000	3	4	4	2	2	2	2	3	3	2	3	2	2	2	3	2	2	2	2	2	3
chr13:18474001	chr13	18,474,001	18,475,000	3	4	4	2	2	3	2	3	3	2	3	2	2	2	3	2	2	2	2	2	3
chr13:18475001	chr13	18,475,001	18,476,000	3	4	4	2	2	3	2	3	3	2	3	2	2	2	3	2	2	2	2	2	3
chr13:18476001	chr13	18,476,001	18,477,000	3	4	4	2	2	3	2	3	3	2	3	2	2	2	3	2	2	2	2	2	3
chr13:18477001	chr13	18,477,001	18,478,000	3	4	4	2	2	3	2	3	3	2	3	2	2	2	3	2	2	2	2	2	3
chr13:18499001	chr13	18,499,001	18,500,000	3	4	4	2	2	2	2	2	2	3	3	2	2	2	2	2	2	2	2	2	3
chr13:18500001	chr13	18,500,001	18,501,000	3	4	4	2	2	2	2	2	3	3	3	2	2	2	2	2	2	2	2	2	3
chr13:18501001	chr13	18,501,001	18,502,000	3	4	4	2	2	2	2	2	3	3	3	2	2	2	2	2	2	2	2	2	3
chr13:18502001	chr13	18,502,001	18,503,000	3	4	4	2	2	2	2	2	3	3	3	2	2	2	2	2	2	2	2	2	3
chr13:18503001	chr13	18,503,001	18,504,000	3	4	4	2	2	2	2	2	3	3	3	2	2	2	2	2	2	2	2	2	3
chr13:18504001	chr13	18,504,001	18,505,000	3	4	4	2	2	2	2	2	3	3	3	2	2	2	2	2	2	2	2	2	3
chr13:18505001	chr13	18,505,001	18,506,000	3	4	4	2	2	3	2	2	3	3	3	2	2	2	2	2	2	2	2	2	3
chr13:18506001	chr13	18,506,001	18,507,000	3	4	4	2	2	3	2	2	3	3	3	2	2	2	2	2	2	2	2	2	3
chr13:18507001	chr13	18,507,001	18,508,000	3	4	4	2	2	3	2	2	3	3	3	2	2	2	2	2	2	2	2	2	3
chr13:18508001	chr13	18,508,001	18,509,000	3	4	4	2	2	3	2	2	3	3	3	2	2	2	2	2	2	2	2	2	3
chr13:18509001	chr13	18,509,001	18,510,000	3	4	4	2	2	3	2	2	3	3	3	2	2	2	2	2	2	2	2	2	3
chr13:18510001	chr13	18,510,001	18,511,000	3	4	4	2	2	3	2	2	3	3	3	2	2	2	2	2	2	2	2	2	3
chr13:18521001	chr13	18,521,001	18,522,000	3	4	4	2	2	3	2	3	3	2	3	2	2	2	3	2	2	2	2	2	3
chr13:18522001	chr13	18,522,001	18,523,000	3	4	4	2	2	3	2	3	3	2	3	2	2	2	3	2	2	2	2	2	3
chr13:18523001	chr13	18,523,001	18,524,000	3	4	4	2	2	3	2	3	3	2	3	2	2	2	3	2	2	2	2	2	3
chr13:18524001	chr13	18,524,001	18,525,000	3	4	4	2	2	3	2	3	3	2	3	2	2	2	3	2	2	2	2	2	3
chr13:18525001	chr13	18,525,001	18,526,000	3	4	4	2	2	2	2	3	3	2	3	2	2	2	3	2	2	2	2	2	3
chr13:18526001	chr13	18,526,001	18,527,000	3	4	4	2	2	2	2	3	3	2	3	2	2	2	3	2	2	2	2	2	3
chr13:18527001	chr13	18,527,001	18,528,000	3	4	4	2	2	2	2	3	3	2	3	2	2	2	3	2	2	2	2	2	3
chr15:21037001	chr15	21,037,001	21,038,000	4	3	4	3	4	2	3	3	4	2	2	2	3	2	2	4	3	2	2	2	1
chr15:21070001	chr15	21,070,001	21,071,000	4	3	4	3	4	2	3	3	4	2	2	2	3	2	2	4	3	2	2	2	1
chr15:21071001	chr15	21,071,001	21,072,000	4	3	4	3	4	2	3	3	4	2	2	2	3	2	2	4	3	2	2	2	1
chr15:21072001	chr15	21,072,001	21,073,000	4	3	4	3	4	2	3	3	4	2	2	2	3	2	2	4	3	2	2	2	1
chr21:5328001	chr21	5,328,001	5,329,000	3	3	4	2	2	2	2	2	2	2	2	2	2	2	2	3	2	3	2	2	3
chr21:5329001	chr21	5,329,001	5,330,000	3	3	4	2	2	2	2	2	2	2	2	2	2	2	2	3	2	3	2	2	3
chr21:5330001	chr21	5,330,001	5,331,000	3	3	4	2	2	2	2	2	3	2	2	2	2	2	2	3	2	3	2	2	3
chr21:5331001	chr21	5,331,001	5,332,000	3	3	4	2	2	2	2	2	3	2	2	2	2	2	2	3	2	3	2	2	3
chr21:5332001	chr21	5,332,001	5,333,000	3	3	4	2	2	2	2	2	3	2	2	2	2	2	2	3	2	3	2	2	3
chr21:5333001	chr21	5,333,001	5,334,000	3	3	4	2	2	2	2	2	3	2	2	2	2	2	2	3	2	3	2	2	3
chr21:5334001	chr21	5,334,001	5,335,000	3	3	4	2	2	2	2	2	3	2	2	2	2	2	2	3	2	3	2	2	3
chr21:5335001	chr21	5,335,001	5,336,000	3	3	4	2	2	2	2	2	3	2	2	2	2	2	2	3	2	3	2	2	3
chr21:5336001	chr21	5,336,001	5,337,000	3	3	4	2	2	2	2	2	3	2	2	2	2	2	2	3	2	3	2	2	3
chr21:5337001	chr21	5,337,001	5,338,000	3	3	4	2	2	2	2	2	3	2	2	2	2	2	2	3	2	3	2	2	3
chr21:5338001	chr21	5,338,001	5,339,000	3	3	4	2	2	2	2	2	3	2	2	2	2	2	2	3	2	3	2	2	3
chr21:5343001	chr21	5,343,001	5,344,000	3	3	4	2	2	2	2	2	3	2	2	2	2	2	2	3	2	3	2	2	3
chr21:5344001	chr21	5,344,001	5,345,000	3	3	4	2	2	2	2	2	3	2	2	2	2	2	2	3	2	3	2	2	3
chr21:5345001	chr21	5,345,001	5,346,000	3	3	4	2	2	2	2	2	3	2	2	2	2	2	2	3	2	3	2	2	3
chr21:5346001	chr21	5,346,001	5,347,000	3	3	4	2	2	2	2	2	3	2	2	2	2	2	2	3	2	3	2	2	3
chr21:5347001	chr21	5,347,001	5,348,000	3	3	4	2	2	2	2	2	3	2	2	2	2	2	2	3	2	3	2	2	3
chr21:5362001	chr21	5,362,001	5,363,000	3	3	4	2	2	2	2	2	3	2	2	2	2	2	3	3	2	3	2	2	2
chr21:9533001	chr21	9,533,001	9,534,000	3	3	4	2	2	2	1	2	2	2	2	2	2	1	2	3	2	3	3	2	3
chr21:10650001	chr21	10,650,001	10,651,000	3	3	4	2	2	2	2	2	2	2	2	2	2	2	2	3	3	3	2	2	2
chr3:90582001	chr3	90,582,001	90,583,000	3	3	3	2	2	2	2	2	2	3	3	2	2	2	2	3	2	2	2	2	2
chr3:90583001	chr3	90,583,001	90,584,000	3	3	3	2	2	2	2	2	2	3	3	2	2	2	2	3	2	2	2	2	2
chr3:90584001	chr3	90,584,001	90,585,000	3	3	3	2	2	2	2	2	2	3	3	2	2	2	2	3	2	2	2	2	2
chr3:90585001	chr3	90,585,001	90,586,000	3	3	3	2	2	2	2	2	2	3	3	2	2	2	2	3	2	2	2	2	2
chr3:90586001	chr3	90,586,001	90,587,000	3	3	3	2	2	2	2	2	2	3	3	2	2	2	2	3	2	2	2	2	2
chr3:90587001	chr3	90,587,001	90,588,000	3	3	3	2	2	2	2	2	2	3	3	2	2	2	2	3	2	2	2	2	2
chr7:57707001	chr7	57,707,001	57,708,000	4	3	3	3	2	2	2	2	3	3	2	3	2	2	2	3	2	2	2	2	2
chr7:57708001	chr7	57,708,001	57,709,000	4	3	3	3	2	2	2	2	3	3	2	3	2	2	2	3	2	2	2	2	2
chr7:57962001	chr7	57,962,001	57,963,000	3	3	3	2	2	2	2	2	2	3	2	3	2	2	2	3	2	2	2	2	2
chr7:63527001	chr7	63,527,001	63,528,000	3	3	3	2	2	2	2	2	2	3	3	2	2	2	2	3	2	2	2	2	2
chr7:63581001	chr7	63,581,001	63,582,000	4	3	3	2	3	2	2	2	2	3	3	3	2	2	2	2	2	2	2	2	2
chr7:63582001	chr7	63,582,001	63,583,000	4	3	3	2	3	2	2	2	2	3	3	3	2	2	2	2	2	2	2	2	2
chr9:60543001	chr9	60,543,001	60,544,000	3	3	3	2	2	2	2	2	2	3	3	2	2	2	2	3	2	2	2	2	2
chr9:67428001	chr9	67,428,001	67,429,000	4	3	3	3	2	2	2	2	2	3	3	2	2	2	2	3	2	2	2	2	2
chr9:67429001	chr9	67,429,001	67,430,000	4	3	3	3	2	2	2	2	2	3	3	2	2	2	2	3	2	2	2	2	2
chr9:67627001	chr9	67,627,001	67,628,000	4	3	3	2	3	3	2	2	2	2	3	2	2	2	2	3	3	2	2	2	2
chr9:67628001	chr9	67,628,001	67,629,000	4	3	3	2	3	3	2	2	2	2	3	2	2	2	2	3	3	2	2	2	2
chr9:67629001	chr9	67,629,001	67,630,000	4	3	3	2	3	3	2	2	2	2	3	2	2	2	2	3	3	2	2	2	2
chr9:67630001	chr9	67,630,001	67,631,000	4	3	3	2	3	3	2	2	2	2	3	2	2	2	2	3	3	2	2	2	2
chr9:67631001	chr9	67,631,001	67,632,000	4	3	3	2	3	3	2	2	2	2	3	2	2	2	2	3	3	2	2	2	2
chr10:38967001	chr10	38,967,001	38,968,000	3	3	3	2	2	2	2	2	2	2	2	2	3	2	2	3	2	2	3	2	2
chr10:38968001	chr10	38,968,001	38,969,000	3	3	3	2	2	2	2	2	2	2	2	2	3	2	2	3	2	2	3	2	2
chr10:39440001	chr10	39,440,001	39,441,000	3	3	3	2	2	2	2	2	2	3	3	2	2	2	2	3	2	2	2	2	2
chr10:39441001	chr10	39,441,001	39,442,000	3	3	3	2	2	2	2	2	2	3	3	2	2	2	2	3	2	2	2	2	2
chr10:39442001	chr10	39,442,001	39,443,000	3	3	3	2	2	2	2	2	2	3	3	2	2	2	2	3	2	2	2	2	2
chr10:39443001	chr10	39,443,001	39,444,000	3	3	3	2	2	2	2	2	2	3	3	2	2	2	2	3	2	2	2	2	2
chr10:39444001	chr10	39,444,001	39,445,000	3	3	3	2	2	2	2	2	2	3	3	2	2	2	2	3	2	2	2	2	2
chr10:39445001	chr10	39,445,001	39,446,000	3	3	3	2	2	2	2	2	2	3	3	2	2	2	2	3	2	2	2	2	2
chr10:42176001	chr10	42,176,001	42,177,000	3	3	3	2	2	2	2	2	2	2	3	2	2	2	2	3	2	2	3	2	2
chr11:50773001	chr11	50,773,001	50,774,000	3	3	3	2	2	2	2	2	2	3	3	2	2	2	2	2	2	3	2	2	2
chr11:50774001	chr11	50,774,001	50,775,000	3	3	3	2	2	2	2	2	2	3	3	2	2	2	2	2	2	3	2	2	2
chr11:50775001	chr11	50,775,001	50,776,000	3	3	3	2	2	2	2	2	2	3	3	2	2	2	2	2	2	3	2	2	2
chr11:50776001	chr11	50,776,001	50,777,000	3	3	3	2	2	2	2	2	2	3	3	2	2	2	2	2	2	3	2	2	2
chr13:18528001	chr13	18,528,001	18,529,000	3	4	3	2	2	2	2	3	3	2	3	2	2	2	3	2	2	2	2	2	3
chr13:18529001	chr13	18,529,001	18,530,000	3	4	3	2	2	2	2	3	3	2	3	2	2	2	3	2	2	2	2	2	3
chr13:52288001	chr13	52,288,001	52,289,000	3	3	3	2	2	2	2	2	2	3	2	2	3	2	2	2	2	2	2	2	3
chr15:19818001	chr15	19,818,001	19,819,000	3	3	3	2	2	2	2	2	2	3	2	3	2	2	2	3	2	2	2	2	2
chr15:19819001	chr15	19,819,001	19,820,000	3	3	3	2	2	2	2	2	2	3	2	3	2	2	2	3	2	2	2	2	2
chr15:19820001	chr15	19,820,001	19,821,000	3	3	3	2	2	2	2	2	2	3	2	3	2	2	2	3	2	2	2	2	2
chr16:33042001	chr16	33,042,001	33,043,000	3	3	3	2	2	2	2	2	2	2	2	3	2	2	2	3	2	2	3	2	2
chr16:33043001	chr16	33,043,001	33,044,000	3	3	3	2	2	2	2	2	2	2	2	3	2	2	2	3	2	2	3	2	2
chr16:33044001	chr16	33,044,001	33,045,000	3	3	3	2	2	2	2	2	2	2	2	3	2	2	2	3	2	2	3	2	2

Region	CHR	start	end	HD2	HD3	HD1	BN1	BN2	BN3	BN4	BN5	BN6	LC1	LC2	LC3	LC4	LC5	LC6	LC7	LC8	LC9	LC10	LC10*	LC11
chr16:34152001	chr16	34,152,001	34,153,000	3	3	3	2	2	2	2	2	2	2	3	3	2	2	2	3	2	2	2	2	2
chr16:34153001	chr16	34,153,001	34,154,000	3	3	3	2	2	2	2	2	2	2	3	3	2	2	2	3	2	2	2	2	2
chr16:34712001	chr16	34,712,001	34,713,000	4	3	3	2	2	2	2	2	2	2	3	3	3	2	2	2	2	2	2	2	2
chr16:34713001	chr16	34,713,001	34,714,000	4	3	3	2	2	2	2	2	2	2	3	3	3	2	2	2	2	2	2	2	2
chr16:34714001	chr16	34,714,001	34,715,000	4	3	3	2	2	2	2	2	2	2	3	3	3	2	2	2	2	2	2	2	2
chr16:34715001	chr16	34,715,001	34,716,000	4	3	3	2	2	2	2	2	2	2	3	3	3	2	2	2	2	2	2	2	2
chr16:34716001	chr16	34,716,001	34,717,000	4	3	3	2	2	2	2	2	2	2	3	3	3	2	2	2	2	2	2	2	2
chr16:34894001	chr16	34,894,001	34,895,000	3	3	3	2	2	2	2	2	2	3	2	3	2	2	2	3	2	2	2	2	2
chr16:34895001	chr16	34,895,001	34,896,000	3	3	3	2	2	2	2	2	2	3	2	3	2	2	2	3	2	2	2	2	2
chr16:34896001	chr16	34,896,001	34,897,000	3	3	3	2	2	2	2	2	2	3	2	3	2	2	2	3	2	2	2	2	2
chr16:34897001	chr16	34,897,001	34,898,000	3	3	3	2	2	2	2	2	2	3	2	3	2	2	2	3	2	2	2	2	2
chr16:34898001	chr16	34,898,001	34,899,000	3	3	3	2	2	2	2	2	2	3	2	3	2	2	2	3	2	2	2	2	2
chr16:34899001	chr16	34,899,001	34,900,000	3	3	3	2	2	2	2	2	2	3	2	3	2	2	2	3	2	2	2	2	2
chr16:34900001	chr16	34,900,001	34,901,000	3	3	3	2	2	2	2	2	2	3	2	3	2	2	2	3	2	2	2	2	2
chr16:34912001	chr16	34,912,001	34,913,000	3	3	3	2	2	2	2	2	2	3	2	3	2	2	2	3	2	2	2	2	2
chr16:34913001	chr16	34,913,001	34,914,000	3	3	3	2	2	2	2	2	2	3	2	3	2	2	2	3	2	2	2	2	2
chr16:34914001	chr16	34,914,001	34,915,000	3	3	3	2	2	2	2	2	2	3	2	3	2	2	2	3	2	2	2	2	2
chr16:34915001	chr16	34,915,001	34,916,000	3	3	3	2	2	2	2	2	2	3	2	3	2	2	2	3	2	2	2	2	2
chr16:34916001	chr16	34,916,001	34,917,000	3	3	3	2	2	2	2	2	2	3	2	3	2	2	2	3	2	2	2	2	2
chr16:36020001	chr16	36,020,001	36,021,000	3	3	3	2	2	2	2	2	2	3	3	2	2	2	2	3	2	2	2	2	2
chr16:36031001	chr16	36,031,001	36,032,000	3	3	3	2	2	2	2	2	2	3	3	2	2	2	2	3	2	2	2	2	2
chr16:36046001	chr16	36,046,001	36,047,000	3	3	3	2	2	2	2	2	2	3	3	3	2	2	2	2	2	2	2	2	2
chr16:36047001	chr16	36,047,001	36,048,000	3	3	3	2	2	2	2	2	2	3	3	3	2	2	2	2	2	2	2	2	2
chr16:36048001	chr16	36,048,001	36,049,000	3	3	3	2	2	2	2	2	2	3	3	3	2	2	2	2	2	2	2	2	2
chr16:36049001	chr16	36,049,001	36,050,000	3	3	3	2	2	2	2	2	2	3	3	3	2	2	2	2	2	2	2	2	2
chr16:36050001	chr16	36,050,001	36,051,000	3	3	3	2	2	2	2	2	2	3	3	3	2	2	2	2	2	2	2	2	2
chr16:36074001	chr16	36,074,001	36,075,000	3	3	3	2	2	2	2	2	2	3	3	2	2	2	2	3	2	2	2	2	2
chr16:36075001	chr16	36,075,001	36,076,000	3	3	3	2	2	2	2	2	2	3	3	2	2	2	2	3	2	2	2	2	2
chr16:36076001	chr16	36,076,001	36,077,000	3	3	3	2	2	2	2	2	2	3	3	2	2	2	2	3	2	2	2	2	2
chr16:36077001	chr16	36,077,001	36,078,000	3	3	3	2	2	2	2	2	2	3	3	2	2	2	2	3	2	2	2	2	2
chr16:36078001	chr16	36,078,001	36,079,000	3	3	3	2	2	2	2	2	2	3	3	2	2	2	2	3	2	2	2	2	2
chr16:36182001	chr16	36,182,001	36,183,000	3	3	3	2	2	2	2	2	2	3	2	3	2	2	2	3	2	2	2	2	2
chr16:36196001	chr16	36,196,001	36,197,000	3	3	3	2	2	2	2	2	2	2	2	3	2	2	2	3	2	3	2	2	2
chr16:36197001	chr16	36,197,001	36,198,000	3	3	3	2	2	2	2	2	2	2	2	3	2	2	2	3	2	3	2	2	2
chr16:36198001	chr16	36,198,001	36,199,000	3	3	3	2	2	2	2	2	2	2	2	3	2	2	2	3	2	3	2	2	2
chr16:36199001	chr16	36,199,001	36,200,000	3	3	3	2	2	2	2	2	2	2	2	3	2	2	2	3	2	3	2	2	2
chr16:36200001	chr16	36,200,001	36,201,000	3	3	3	2	2	2	2	2	2	2	2	3	2	2	2	3	2	3	2	2	2
chr16:36201001	chr16	36,201,001	36,202,000	3	3	3	2	2	2	2	2	2	2	2	3	2	2	2	3	2	3	2	2	2
chr16:36227001	chr16	36,227,001	36,228,000	3	3	3	2	2	2	2	2	2	3	2	3	2	2	2	3	2	2	2	2	2
chr16:36228001	chr16	36,228,001	36,229,000	3	3	3	2	2	2	2	2	2	3	2	3	2	2	2	3	2	2	2	2	2
chr16:36251001	chr16	36,251,001	36,252,000	3	3	3	2	2	2	2	2	2	2	3	3	2	2	2	3	2	2	2	2	2
chr17:21652001	chr17	21,652,001	21,653,000	3	3	3	2	2	2	2	2	2	3	2	3	2	2	2	3	2	2	2	2	2
chr17:21653001	chr17	21,653,001	21,654,000	3	3	3	2	2	2	2	2	2	3	2	3	2	2	2	3	2	2	2	2	2
chr17:21670001	chr17	21,670,001	21,671,000	3	3	3	2	2	2	2	2	2	2	3	2	2	2	2	3	2	2	3	2	2
chr17:21671001	chr17	21,671,001	21,672,000	3	3	3	2	2	2	2	2	2	2	3	2	2	2	2	3	2	2	3	2	2
chr17:21672001	chr17	21,672,001	21,673,000	3	3	3	2	2	2	2	2	2	2	3	2	2	2	2	3	2	2	3	2	2
chr17:21674001	chr17	21,674,001	21,675,000	3	3	3	2	2	2	2	2	2	2	3	3	2	2	2	2	2	2	3	2	2
chr17:21675001	chr17	21,675,001	21,676,000	3	3	3	2	2	2	2	2	2	2	3	3	2	2	2	2	2	2	3	2	2
chr17:21676001	chr17	21,676,001	21,677,000	3	3	3	2	2	2	2	2	2	2	3	3	2	2	2	2	2	2	3	2	2
chr17:22118001	chr17	22,118,001	22,119,000	3	3	3	2	2	2	2	2	2	3	2	3	2	2	2	2	2	3	2	2	2
chr21:8539001	chr21	8,539,001	8,540,000	3	3	3	2	2	2	2	2	2	3	2	3	2	2	2	3	2	2	2	2	2
chr21:8540001	chr21	8,540,001	8,541,000	3	3	3	2	2	2	2	2	2	3	2	3	2	2	2	3	2	2	2	2	2
chr21:8541001	chr21	8,541,001	8,542,000	3	3	3	2	2	2	2	2	2	3	2	3	2	2	2	3	2	2	2	2	2
chr21:8542001	chr21	8,542,001	8,543,000	3	3	3	2	2	2	2	2	2	3	2	3	2	2	2	3	2	2	2	2	2
chr21:8543001	chr21	8,543,001	8,544,000	3	3	3	2	2	2	2	2	2	3	2	3	2	2	2	3	2	2	2	2	2
chr21:8544001	chr21	8,544,001	8,545,000	3	3	3	2	2	2	2	2	2	3	2	3	2	2	2	3	2	2	2	2	2
chr21:8545001	chr21	8,545,001	8,546,000	3	3	3	2	2	2	2	2	2	3	2	3	2	2	2	3	2	2	2	2	2
chr21:8546001	chr21	8,546,001	8,547,000	3	3	3	2	2	2	2	2	2	3	2	3	2	2	2	3	2	2	2	2	2
chr21:8547001	chr21	8,547,001	8,548,000	3	3	3	2	2	2	2	2	2	3	2	3	2	2	2	3	2	2	2	2	2
chr21:8548001	chr21	8,548,001	8,549,000	3	3	3	2	2	2	2	2	2	3	2	3	2	2	2	3	2	2	2	2	2
chr21:8549001	chr21	8,549,001	8,550,000	3	3	3	2	2	2	2	2	2	3	2	3	2	2	2	3	2	2	2	2	2
chr21:8550001	chr21	8,550,001	8,551,000	3	3	3	2	2	2	2	2	2	3	2	3	2	2	2	3	2	2	2	2	2
chr21:8551001	chr21	8,551,001	8,552,000	3	3	3	2	2	2	2	2	2	3	2	3	2	2	2	3	2	2	2	2	2
chr21:8552001	chr21	8,552,001	8,553,000	3	3	3	2	2	2	2	2	2	3	2	3	2	2	2	3	2	2	2	2	2
chr21:8553001	chr21	8,553,001	8,554,000	3	3	3	2	2	2	2	2	2	3	2	3	2	2	2	3	2	2	2	2	2
chr21:8554001	chr21	8,554,001	8,555,000	3	3	3	2	2	2	2	2	2	3	2	3	2	2	2	3	2	2	2	2	2
chr21:8555001	chr21	8,555,001	8,556,000	3	3	3	2	2	2	2	2	2	3	2	3	2	2	2	3	2	2	2	2	2
chr21:8556001	chr21	8,556,001	8,557,000	3	3	3	2	2	2	2	2	2	3	2	3	2	2	2	3	2	2	2	2	2
chr21:8557001	chr21	8,557,001	8,558,000	3	3	3	2	2	2	2	2	2	3	2	3	2	2	2	3	2	2	2	2	2
chr21:8558001	chr21	8,558,001	8,559,000	3	3	3	2	2	2	2	2	2	3	2	3	2	2	2	3	2	2	2	2	2
chr21:8559001	chr21	8,559,001	8,560,000	3	3	3	2	2	2	2	2	2	3	2	3	2	2	2	3	2	2	2	2	2
chr21:8560001	chr21	8,560,001	8,561,000	3	3	3	2	2	2	2	2	2	3	2	3	2	2	2	3	2	2	2	2	2
chr21:8561001	chr21	8,561,001	8,562,000	3	3	3	2	2	2	2	2	2	3	2	3	2	2	2	3	2	2	2	2	2
chr21:8562001	chr21	8,562,001	8,563,000	3	3	3	2	2	2	2	2	2	3	2	3	2	2	2	3	2	2	2	2	2
chr21:8563001	chr21	8,563,001	8,564,000	3	3	3	2	2	2	2	2	2	3	2	3	2	2	2	3	2	2	2	2	2
chr21:8564001	chr21	8,564,001	8,565,000	3	3	3	2	2	2	2	2	2	3	2	3	2	2	2	3	2	2	2	2	2
chr21:8565001	chr21	8,565,001	8,566,000	3	3	3	2	2	2	2	2	2	3	2	3	2	2	2	3	2	2	2	2	2
chr21:8566001	chr21	8,566,001	8,567,000	3	3	3	2	2	2	2	2	2	3	2	3	2	2	2	3	2	2	2	2	2
chr21:8567001	chr21	8,567,001	8,568,000	3	3	3	2	2	2	2	2	2	3	2	3	2	2	2	3	2	2	2	2	2
chr21:8568001	chr21	8,568,001	8,569,000	3	3	3	2	2	2	2	2	2	3	2	3	2	2	2	3	2	2	2	2	2
chr21:8569001	chr21	8,569,001	8,570,000	3	3	3	2	2	2	2	2	2	3	2	3	2	2	2	3	2	2	2	2	2
chr21:8570001	chr21	8,570,001	8,571,000	3	3	3	2	2	2	2	2	2	3	2	3	2	2	2	3	2	2	2	2	2
chr21:8574001	chr21	8,574,001	8,575,000	3	3	3	2	2	2	2	2	2	3	2	3	2	2	2	3	2	2	2	2	2
chr21:8598001	chr21	8,598,001	8,599,000	3	3	3	2	2	2	2	2	2	3	2	3	2	2	2	3	2	2	2	2	2
chr21:8599001	chr21	8,599,001	8,600,000	3	3	3	2	2	2	2	2	2	3	2	3	2	2	2	3	2	2	2	2	2
chr21:8600001	chr21	8,600,001	8,601,000	3	3	3	2	2	2	2	2	2	3	2	3	2	2	2	3	2	2	2	2	2
chr21:9870001	chr21	9,870,001	9,871,000	3	3	3	2	2	2	2	2	2	2	2	2	2	2	2	3	2	3	2	2	3
chr21:9871001	chr21	9,871,001	9,872,000	3	3	3	2	2	2	2	2	2	2	2	2	2	2	2	3	2	3	2	2	3
chr21:9872001	chr21	9,872,001	9,873,000	3	3	3	2	2	2	2	2	2	2	2	2	2	2	2	3	2	3	2	2	3
chr21:9873001	chr21	9,873,001	9,874,000	3	3	3	2	2	2	2	2	2	2	2	2	2	2	2	3	2	3	2	2	3
chr21:9874001	chr21	9,874,001	9,875,000	3	3	3	2	2	2	2	2	2	2	2	2	2	2	2	3	2	3	2	2	3
chr21:9875001	chr21	9,875,001	9,876,000	3	3	3	2	2	2	2	2	2	2	2	2	2	2	2	3	2	3	2	2	3
chr21:9876001	chr21	9,876,001	9,877,000	3	3	3	2	2	2	2	2	2	2	2	2	2	2	2	3	2	3	2	2	3
chr21:9877001	chr21	9,877,001	9,878,000	3	3	3	2	2	2	2	2	2	2	2	2	2	2	2	3	2	3	2	2	3
chr21:9878001	chr21	9,878,001	9,879,000	3	3	3	2	2	2	2	2	2	2	2	2	2	2	2	3	2	3	2	2	3
chr21:9879001	chr21	9,879,001	9,880,000	3	3	3	2	2	2	2	2	2	2	2	2	2	2	2	3	2	3	2	2	3
chr21:10651001	chr21	10,651,001	10,652,000	3	3	3	2	2	2	2	2	2	2	2	2	2	2	2	3	3	3	2	2	2
chr21:10652001	chr21	10,652,001	10,653,000	3	3	3	2	2	2	2	2	2	2	2	2	2	2	2	3	3	3	2	2	2
chr22:10601001	chr22	10,601,001	10,602,000	4	3	3	3	2	3	2	2	2	3	2	2	2	3	2	3	2	2	2	2	2
chr22:10602001	chr22	10,602,001	10,603,000	4	3	3	3	2	3	2	2	2	3	2	2	2	3	2	3	2	2	2	2	2
chr22:10603001	chr22	10,603,001	10,604,000	4	3	3	3	2	3	2	2	2	3	2	2	2	3	2	3	2	2	2	2	2
chr22:10604001	chr22	10,604,001	10,605,000	4	3	3	3	2	3	2	2	2	3	2	2	2	3	2	3	2	2	2	2	2
chr22:10605001	chr22	10,605,001	10,606,000	4	3	3	3	2	3	2	2	2	3	2	2	2	3	2	3	2	2	2	2	2
chr22:10606001	chr22	10,606,001	10,607,000	4	3	3	3	2	3	2	2	2	3	2	2	2	3	2	3	2	2	2	2	2
chr22:10607001	chr22	10,607,001	10,608,000	4	3	3	3	2	3	2	2	2	3	2	2	2	3	2	3	2	2	2	2	2
chr22:10608001	chr22	10,608,001	10,609,000	4	3	3	2	2	3	2	2	2	3	2	2	2	3	2	3	2	2	2	2	2
chr22:12182001	chr22	12,182,001	12,183,000	3	3	3	2	2	2	1	2	2	2	2	2	2	3	2	2	2	3	3	2	2
chr22:12183001	chr22	12,183,001	12,184,000	3	3	3	2	2	2	1	2	2	2	2	2	2	3	2	2	2	3	3	2	2
chr1:500001	chr1	500,001	501,000	2	2	2	3	3	3	3	3	3	2	2	2	3	2	2	3	3	2	2	2	2
chr1:501001	chr1	501,001	502,000	2	2	2	3	3	3	3	3	3	2	2	2	3	2	2	3	3	2	2	2	2
chr2:241590001	chr2	241,590,001	241,591,000	5	5	2	2	2	2	2	2	2	2	2	2	3	2	2	2	2	2	4	2	2
chr5:70015001	chr5	70,015,001	70,016,000	2	2	2	3	3	3	3	3	3	2	2	2	3	3	2	1	3	3	2	2	2
chr6:134001	chr6	134,001	135,000	2	2	2	2	3	4	4	3	3	2	2	2	3	2	3	2	2	2	2	3	2

Region	CHR	start	end	HD2	HD3	HD1	BN1	BN2	BN3	BN4	BN5	BN6	LC1	LC2	LC3	LC4	LC5	LC6	LC7	LC8	LC9	LC10	LC10*	LC11
chr21:44111001	chr21	44,111,001	44,112,000	2	2	2	3	3	3	4	4	4	2	3	2	2	3	3	2	2	2	2	2	2
chr21:44112001	chr21	44,112,001	44,113,000	2	2	2	3	3	3	4	4	3	2	3	2	2	3	3	2	2	2	2	2	2
chr21:44113001	chr21	44,113,001	44,114,000	2	2	2	3	3	3	4	4	3	2	3	2	2	3	3	2	2	2	2	2	2
chr21:44114001	chr21	44,114,001	44,115,000	2	2	2	3	3	3	4	4	3	2	3	2	2	3	3	2	2	2	2	2	2
chr21:44125001	chr21	44,125,001	44,126,000	2	2	2	3	3	3	3	3	3	2	3	2	2	3	3	2	2	2	2	2	2
chr21:44126001	chr21	44,126,001	44,127,000	2	2	2	3	3	3	3	3	3	2	3	2	2	3	3	2	2	2	2	2	2
chr21:44127001	chr21	44,127,001	44,128,000	2	2	2	3	3	3	3	3	3	2	3	2	2	3	3	2	2	2	2	2	2
chr21:44128001	chr21	44,128,001	44,129,000	2	2	2	3	3	3	3	3	3	2	3	2	2	3	3	2	2	2	2	2	2
chr21:44129001	chr21	44,129,001	44,130,000	2	2	2	3	3	3	3	3	3	2	3	2	2	3	3	2	2	2	2	2	2
chr21:44133001	chr21	44,133,001	44,134,000	2	2	2	3	3	3	3	3	3	2	3	2	2	3	3	2	2	2	2	2	2
chr21:44134001	chr21	44,134,001	44,135,000	2	2	2	3	3	3	3	3	3	2	3	2	2	3	3	2	2	2	2	2	2
chr21:44135001	chr21	44,135,001	44,136,000	2	2	2	3	3	3	3	3	3	2	3	2	2	3	3	2	2	2	2	2	2
chr21:44136001	chr21	44,136,001	44,137,000	2	2	2	3	3	3	3	3	3	2	3	2	2	3	3	2	2	2	2	2	2
chr21:44137001	chr21	44,137,001	44,138,000	2	2	2	3	3	3	3	4	3	2	3	2	2	3	3	2	2	2	2	2	2
chr21:44138001	chr21	44,138,001	44,139,000	2	2	2	3	3	3	3	4	3	2	3	2	2	3	3	2	2	2	2	2	2
chr21:44139001	chr21	44,139,001	44,140,000	2	2	2	3	3	3	3	4	3	2	3	2	2	3	3	2	2	2	2	2	2
chr6:170635001	chr6	170,635,001	170,636,000	1	1	1	2	2	2	3	2	2	2	2	2	3	2	3	2	2	2	2	3	2
chr9:40406001	chr9	40,406,001	40,407,000	2	0	1	3	2	3	2	2	2	3	2	2	2	3	2	2	3	2	2	2	2
chr15:101836001	chr15	101,836,001	101,837,000	2	2	1	2	3	3	3	3	3	2	2	3	2	2	3	3	2	2	2	2	2
chr16:18727001	chr16	18,727,001	18,728,000	2	1	1	2	2	2	2	2	2	2	3	2	3	3	2	2	2	2	2	2	2
chr19:132001	chr19	132,001	133,000	1	1	1	2	2	3	4	3	2	2	2	2	3	2	3	2	3	3	2	2	1
chr19:133001	chr19	133,001	134,000	1	1	1	2	2	3	4	3	2	2	2	2	3	2	3	2	3	3	2	2	1
chr21:44140001	chr21	44,140,001	44,141,000	2	2	1	3	3	3	3	4	3	2	3	2	2	3	3	2	2	2	2	2	2
chr21:44141001	chr21	44,141,001	44,142,000	2	2	1	3	3	3	3	4	3	2	3	2	2	3	3	2	2	2	2	2	2
chr4:1707001	chr4	1,707,001	1,708,000	5	4	5	2	2	2	2	2	2	2	2	2	2	2	2	2	2	2	4	2	2
chr7:157725001	chr7	157,725,001	157,726,000	2	4	5	2	2	2	2	2	2	2	2	2	2	2	2	4	2	2	2	2	2
chr13:62003001	chr13	62,003,001	62,004,000	2	5	5	2	2	2	2	2	2	2	2	2	2	2	2	2	2	2	2	4	2
chr2:89033001	chr2	89,033,001	89,034,000	3	3	4	2	2	2	2	2	2	3	3	2	2	2	2	2	2	2	2	2	2
chr2:89048001	chr2	89,048,001	89,049,000	3	3	4	2	2	2	2	2	2	2	2	2	2	2	2	3	2	3	2	2	2
chr2:89049001	chr2	89,049,001	89,050,000	3	3	4	2	2	2	2	2	2	2	2	2	2	2	2	3	2	3	2	2	2
chr3:198168001	chr3	198,168,001	198,169,000	4	3	4	3	2	3	2	2	3	1	2	3	2	3	3	2	1	3	2	2	2
chr3:198170001	chr3	198,170,001	198,171,000	4	3	4	3	2	3	2	2	3	1	2	3	2	2	3	3	1	3	2	2	2
chr3:198171001	chr3	198,171,001	198,172,000	4	3	4	3	3	3	2	2	3	1	2	3	2	2	3	3	1	3	2	2	2
chr3:198172001	chr3	198,172,001	198,173,000	4	3	4	3	3	3	2	2	3	1	2	3	2	2	3	3	1	3	2	2	2
chr4:33843001	chr4	33,843,001	33,844,000	3	3	4	2	2	2	2	2	2	2	2	3	2	2	2	3	2	2	2	2	2
chr9:64052001	chr9	64,052,001	64,053,000	4	3	4	2	2	2	2	2	2	3	3	2	2	2	2	2	2	2	2	2	2
chr9:64053001	chr9	64,053,001	64,054,000	4	3	4	3	2	2	2	2	2	3	3	2	2	2	2	2	2	2	2	2	2
chr9:64054001	chr9	64,054,001	64,055,000	4	3	4	3	2	2	2	2	2	3	3	2	2	2	2	2	2	2	2	2	2
chr9:64055001	chr9	64,055,001	64,056,000	4	3	4	3	2	2	2	2	2	3	3	2	2	2	2	2	2	2	2	2	2
chr9:64056001	chr9	64,056,001	64,057,000	4	3	4	3	2	2	2	2	2	3	3	2	2	2	2	2	2	2	2	2	2
chr13:18460001	chr13	18,460,001	18,461,000	3	4	4	2	2	2	2	3	3	2	3	2	2	2	2	2	2	2	2	2	3
chr13:18461001	chr13	18,461,001	18,462,000	3	4	4	2	2	2	2	3	3	2	3	2	2	2	2	2	2	2	2	2	3
chr13:18463001	chr13	18,463,001	18,464,000	3	4	4	2	2	2	2	3	3	2	3	2	2	2	2	2	2	2	2	2	3
chr13:18464001	chr13	18,464,001	18,465,000	3	4	4	2	2	2	2	3	3	2	3	2	2	2	2	2	2	2	2	2	3
chr13:18465001	chr13	18,465,001	18,466,000	3	4	4	2	2	2	2	3	3	2	3	2	2	2	2	2	2	2	2	2	3
chr13:18466001	chr13	18,466,001	18,467,000	3	4	4	2	2	2	2	3	3	2	3	2	2	2	2	2	2	2	2	2	3
chr13:18467001	chr13	18,467,001	18,468,000	3	4	4	2	2	2	2	3	3	2	3	2	2	2	2	2	2	2	2	2	3
chr13:18468001	chr13	18,468,001	18,469,000	3	4	4	2	2	2	2	3	3	2	3	2	2	2	2	2	2	2	2	2	3
chr13:18469001	chr13	18,469,001	18,470,000	3	4	4	2	2	3	2	3	3	2	3	2	2	2	2	2	2	2	2	2	3
chr13:18470001	chr13	18,470,001	18,471,000	3	4	4	2	2	3	2	3	3	2	3	2	2	2	2	2	2	2	2	2	3
chr13:18471001	chr13	18,471,001	18,472,000	3	4	4	2	2	3	2	3	3	2	3	2	2	2	2	2	2	2	2	2	3
chr13:18472001	chr13	18,472,001	18,473,000	3	4	4	2	2	3	2	3	3	2	3	2	2	2	2	2	2	2	2	2	3
chr13:18473001	chr13	18,473,001	18,474,000	3	4	4	2	2	3	2	3	3	2	3	2	2	2	2	2	2	2	2	2	3
chr13:18478001	chr13	18,478,001	18,479,000	3	4	4	2	2	3	2	3	3	2	3	2	2	2	2	2	2	2	2	2	3
chr13:18479001	chr13	18,479,001	18,480,000	3	4	4	2	2	3	2	3	3	2	3	2	2	2	2	2	2	2	2	2	3
chr13:18480001	chr13	18,480,001	18,481,000	3	4	4	2	2	3	2	3	3	2	3	2	2	2	2	2	2	2	2	2	3
chr13:18481001	chr13	18,481,001	18,482,000	3	4	4	2	2	3	2	2	3	2	3	2	2	2	2	2	2	2	2	2	3
chr13:18482001	chr13	18,482,001	18,483,000	3	4	4	2	2	3	2	2	3	2	3	2	2	2	2	2	2	2	2	2	3
chr13:18489001	chr13	18,489,001	18,490,000	3	4	4	2	2	3	2	2	3	2	2	2	2	2	3	2	2	2	2	2	3
chr13:18495001	chr13	18,495,001	18,496,000	3	4	4	2	2	2	2	2	2	2	3	2	2	2	2	2	2	2	2	2	3
chr13:18496001	chr13	18,496,001	18,497,000	3	4	4	2	2	2	2	2	2	2	3	2	2	2	2	2	2	2	2	2	3
chr13:18497001	chr13	18,497,001	18,498,000	3	4	4	2	2	2	2	2	2	2	3	2	2	2	2	2	2	2	2	2	3
chr13:18498001	chr13	18,498,001	18,499,000	3	4	4	2	2	2	2	2	2	2	3	2	2	2	2	2	2	2	2	2	3
chr21:5348001	chr21	5,348,001	5,349,000	3	3	4	2	2	2	2	2	3	2	2	2	1	2	2	3	2	3	2	2	3
chr21:5360001	chr21	5,360,001	5,361,000	3	3	4	2	2	2	1	2	3	2	2	2	2	2	2	3	2	3	2	2	2
chr21:5361001	chr21	5,361,001	5,362,000	3	3	4	2	2	2	2	2	3	2	2	2	2	2	2	3	2	3	2	2	2
chr2:91807001	chr2	91,807,001	91,808,000	3	3	3	2	2	2	2	2	3	2	2	3	2	2	2	3	2	2	2	2	2
chr2:92054001	chr2	92,054,001	92,055,000	3	3	3	2	2	2	2	2	2	2	2	3	2	2	2	2	2	2	3	2	2
chr2:92055001	chr2	92,055,001	92,056,000	3	3	3	2	2	2	2	2	2	2	2	3	2	2	2	2	2	2	3	2	2
chr2:92056001	chr2	92,056,001	92,057,000	3	3	3	2	2	2	2	2	2	2	2	3	2	2	2	2	2	2	3	2	2
chr2:92057001	chr2	92,057,001	92,058,000	3	3	3	2	2	2	2	2	2	2	2	3	2	2	2	2	2	2	3	2	2
chr2:92058001	chr2	92,058,001	92,059,000	3	3	3	2	2	2	2	2	2	2	2	3	2	2	2	2	2	2	3	2	2
chr2:92059001	chr2	92,059,001	92,060,000	3	3	3	2	2	2	2	2	2	2	2	3	2	2	2	2	2	2	3	2	2
chr2:92060001	chr2	92,060,001	92,061,000	3	3	3	2	2	2	2	2	2	2	2	3	2	2	2	2	2	2	3	2	2
chr2:92061001	chr2	92,061,001	92,062,000	3	3	3	2	2	2	2	2	2	2	2	3	2	2	2	2	2	2	3	2	2
chr2:92062001	chr2	92,062,001	92,063,000	3	3	3	2	2	2	2	2	2	2	2	3	2	2	2	2	2	2	3	2	2
chr2:92063001	chr2	92,063,001	92,064,000	3	3	3	2	2	2	2	2	2	2	2	3	2	2	2	2	2	2	3	2	2
chr2:92064001	chr2	92,064,001	92,065,000	3	3	3	2	2	2	2	2	2	2	2	3	2	2	2	3	2	2	2	2	2
chr2:92065001	chr2	92,065,001	92,066,000	3	3	3	2	2	2	2	2	2	2	2	3	2	2	2	3	2	2	2	2	2
chr7:57619001	chr7	57,619,001	57,620,000	3	3	3	2	3	2	2	2	2	2	3	2	2	2	2	3	2	2	2	2	2
chr7:57620001	chr7	57,620,001	57,621,000	3	3	3	2	3	2	2	2	2	2	3	2	2	2	2	3	2	2	2	2	2
chr7:57621001	chr7	57,621,001	57,622,000	3	3	3	2	3	2	2	2	2	2	3	2	2	2	2	3	2	2	2	2	2
chr7:57622001	chr7	57,622,001	57,623,000	3	3	3	2	3	2	2	2	2	2	3	2	2	2	2	3	2	2	2	2	2
chr7:57623001	chr7	57,623,001	57,624,000	3	3	3	2	3	2	2	2	2	2	3	2	2	2	2	3	2	2	2	2	2
chr7:57624001	chr7	57,624,001	57,625,000	3	3	3	2	3	2	2	2	2	2	3	2	2	2	2	3	2	2	2	2	2
chr7:57625001	chr7	57,625,001	57,626,000	3	3	3	2	3	2	2	2	2	2	3	2	2	2	2	3	2	2	2	2	2
chr7:57628001	chr7	57,628,001	57,629,000	3	3	3	2	3	2	2	2	2	2	2	2	2	2	2	3	2	3	2	2	2
chr7:57634001	chr7	57,634,001	57,635,000	3	3	3	2	3	2	2	2	2	2	2	2	2	2	2	3	2	2	2	2	3
chr7:57635001	chr7	57,635,001	57,636,000	3	3	3	2	3	2	2	2	2	2	2	2	2	2	2	3	2	2	2	2	3
chr7:57636001	chr7	57,636,001	57,637,000	3	3	3	2	3	2	2	2	2	2	2	2	2	2	2	3	2	2	2	2	3
chr7:57637001	chr7	57,637,001	57,638,000	3	3	3	2	3	2	2	2	2	2	2	2	2	2	2	3	2	2	2	2	3
chr7:57709001	chr7	57,709,001	57,710,000	4	3	3	3	2	2	2	2	3	3	2	2	2	2	2	3	2	2	2	2	2
chr7:57710001	chr7	57,710,001	57,711,000	4	3	3	3	2	2	2	2	3	3	2	2	2	2	2	3	2	2	2	2	2
chr7:57711001	chr7	57,711,001	57,712,000	4	3	3	3	2	2	2	2	3	3	2	2	2	2	2	3	2	2	2	2	2
chr7:57712001	chr7	57,712,001	57,713,000	4	3	3	3	2	2	2	2	3	3	2	2	2	2	2	3	2	2	2	2	2
chr7:57713001	chr7	57,713,001	57,714,000	4	3	3	3	2	2	2	2	3	3	2	2	2	2	2	3	2	2	2	2	2
chr7:57714001	chr7	57,714,001	57,715,000	4	3	3	3	2	2	2	2	3	3	2	2	2	2	2	3	2	2	2	2	2
chr7:57715001	chr7	57,715,001	57,716,000	4	3	3	3	2	2	2	2	3	3	2	2	2	2	2	3	2	2	2	2	2
chr7:65147001	chr7	65,147,001	65,148,000	3	3	3	2	2	2	2	2	2	2	3	2	2	3	2	2	2	2	2	2	2
chr7:65148001	chr7	65,148,001	65,149,000	3	3	3	2	2	2	2	2	2	2	3	2	2	3	2	2	2	2	2	2	2
chr7:65149001	chr7	65,149,001	65,150,000	3	3	3	2	2	2	2	2	2	2	3	2	2	3	2	2	2	2	2	2	2
chr7:65150001	chr7	65,150,001	65,151,000	3	3	3	2	2	2	2	2	2	2	3	2	2	3	2	2	2	2	2	2	2
chr9:39874001	chr9	39,874,001	39,875,000	3	3	3	2	2	3	2	2	2	2	2	2	2	3	2	2	3	2	2	2	2
chr9:39875001	chr9	39,875,001	39,876,000	3	3	3	2	2	3	2	2	2	2	2	2	2	3	2	2	3	2	2	2	2
chr9:39876001	chr9	39,876,001	39,877,000	3	3	3	2	2	3	2	2	2	2	2	2	2	3	2	2	3	2	2	2	2
chr9:40172001	chr9	40,172,001	40,173,000	3	3	3	3	3	3	2	3	2	2	2	2	2	3	2	3	2	2	2	2	2
chr9:41720001	chr9	41,720,001	41,721,000	3	3	3	2	2	2	1	1	2	2	3	2	2	2	2	3	2	2	2	2	2
chr9:41721001	chr9	41,721,001	41,722,000	3	3	3	2	2	2	1	1	2	2	3	2	2	2	2	3	2	2	2	2	2
chr9:41722001	chr9	41,722,001	41,723,000	3	3	3	2	2	2	1	1	2	2	3	2	2	2	2	3	2	2	2	2	2
chr9:41723001	chr9	41,723,001	41,724,000	3	3	3	2	2	2	1	2	2	2	3	2	2	2	2	3	2	2	2	2	2
chr9:41724001	chr9	41,724,001	41,725,000	3	3	3	2	2	2	1	2	2	2	3	2	2	2	2	3	2	2	2	2	2
chr9:41725001	chr9	41,725,001	41,726,000	3	3	3	2	2	2	1	2	2	2	3	2	2	2	2	3	2	2	2	2	2
chr9:41726001	chr9	41,726,001	41,727,000	3	3	3	2	2	2	1	2	2	2	3	2	2	2	2	3	2	2	2	2	2

Region	CHR	start	end	HD2	HD3	HD1	BN1	BN2	BN3	BN4	BN5	BN6	LC1	LC2	LC3	LC4	LC5	LC6	LC7	LC8	LC9	LC10	LC10*	LC11
chr9:43292001	chr9	43,292,001	43,293,000	3	3	3	2	2	2	2	2	2	2	2	3	2	2	2	2	2	2	3	2	2
chr9:43293001	chr9	43,293,001	43,294,000	3	3	3	2	2	2	2	2	2	2	2	3	2	2	2	2	2	2	3	2	2
chr9:43294001	chr9	43,294,001	43,295,000	3	3	3	2	2	2	2	2	2	2	2	3	2	2	2	2	2	2	3	2	2
chr9:43295001	chr9	43,295,001	43,296,000	3	3	3	2	2	2	2	2	2	2	2	3	2	2	2	2	2	2	3	2	2
chr9:64851001	chr9	64,851,001	64,852,000	3	3	3	2	2	2	1	1	2	2	3	2	2	2	1	3	3	2	2	2	2
chr9:67430001	chr9	67,430,001	67,431,000	4	3	3	3	2	2	2	2	2	3	3	2	2	2	2	2	2	2	2	2	2
chr10:38969001	chr10	38,969,001	38,970,000	3	3	3	2	2	2	2	2	2	2	2	2	3	2	2	2	2	2	3	2	2
chr13:18410001	chr13	18,410,001	18,411,000	3	4	3	2	3	2	2	3	2	2	3	2	2	2	2	2	2	2	2	2	3
chr13:18411001	chr13	18,411,001	18,412,000	3	4	3	2	3	2	2	3	2	2	3	2	2	2	2	2	2	2	2	2	3
chr13:18412001	chr13	18,412,001	18,413,000	3	4	3	2	3	2	2	3	2	2	3	2	2	2	2	2	2	2	2	2	3
chr13:18413001	chr13	18,413,001	18,414,000	3	4	3	2	3	2	2	3	2	2	3	2	2	2	2	2	2	2	2	2	3
chr13:18414001	chr13	18,414,001	18,415,000	3	4	3	2	3	2	2	3	2	2	3	2	2	2	2	2	2	2	2	2	3
chr13:18419001	chr13	18,419,001	18,420,000	3	3	3	2	3	2	2	2	2	2	3	2	2	2	2	2	2	2	2	2	3
chr13:18420001	chr13	18,420,001	18,421,000	3	3	3	2	3	2	2	2	2	2	3	2	2	2	2	2	2	2	2	2	3
chr13:18421001	chr13	18,421,001	18,422,000	3	3	3	2	3	2	2	2	2	2	3	2	2	2	2	2	2	2	2	2	3
chr13:18422001	chr13	18,422,001	18,423,000	3	3	3	2	3	2	2	2	2	2	3	2	2	2	2	2	2	2	2	2	3
chr13:18423001	chr13	18,423,001	18,424,000	3	3	3	2	2	2	2	2	2	2	3	2	2	2	2	2	2	2	2	2	3
chr16:34151001	chr16	34,151,001	34,152,000	3	3	3	2	2	2	2	2	2	2	3	3	2	2	2	2	2	2	2	2	2
chr16:34257001	chr16	34,257,001	34,258,000	3	3	3	3	2	2	2	2	2	3	3	2	2	2	2	2	2	2	2	2	2
chr16:34267001	chr16	34,267,001	34,268,000	3	3	3	3	2	2	2	2	2	2	3	2	2	2	2	3	2	2	2	2	2
chr16:34268001	chr16	34,268,001	34,269,000	3	3	3	3	2	2	2	2	2	2	3	2	2	2	2	3	2	2	2	2	2
chr16:34269001	chr16	34,269,001	34,270,000	3	3	3	3	2	2	2	2	2	2	3	2	2	2	2	3	2	2	2	2	2
chr16:34270001	chr16	34,270,001	34,271,000	3	3	3	3	2	2	2	2	2	2	3	2	2	2	2	3	2	2	2	2	2
chr16:34271001	chr16	34,271,001	34,272,000	3	3	3	3	2	2	2	2	2	2	3	2	2	2	2	3	2	2	2	2	2
chr16:34272001	chr16	34,272,001	34,273,000	3	3	3	3	2	2	2	2	2	2	3	2	2	2	2	3	2	2	2	2	2
chr16:34750001	chr16	34,750,001	34,751,000	3	3	3	2	2	2	2	2	2	3	2	3	2	2	2	2	2	2	2	2	2
chr16:34751001	chr16	34,751,001	34,752,000	3	3	3	2	2	2	2	2	2	3	2	3	2	2	2	2	2	2	2	2	2
chr16:34752001	chr16	34,752,001	34,753,000	3	3	3	2	2	2	2	2	2	3	2	3	2	2	2	2	2	2	2	2	2
chr16:34753001	chr16	34,753,001	34,754,000	3	3	3	2	2	2	2	2	2	3	2	3	2	2	2	2	2	2	2	2	2
chr16:34754001	chr16	34,754,001	34,755,000	3	3	3	2	2	2	2	2	2	3	2	3	2	2	2	2	2	2	2	2	2
chr16:34755001	chr16	34,755,001	34,756,000	3	3	3	2	2	2	2	2	2	3	2	3	2	2	2	2	2	2	2	2	2
chr16:34756001	chr16	34,756,001	34,757,000	3	3	3	2	2	2	2	2	2	3	2	3	2	2	2	2	2	2	2	2	2
chr16:34757001	chr16	34,757,001	34,758,000	3	3	3	2	2	2	2	2	2	3	2	3	2	2	2	2	2	2	2	2	2
chr16:34758001	chr16	34,758,001	34,759,000	3	3	3	2	2	2	2	2	2	3	2	3	2	2	2	2	2	2	2	2	2
chr16:34759001	chr16	34,759,001	34,760,000	3	3	3	2	2	2	2	2	2	3	2	3	2	2	2	2	2	2	2	2	2
chr16:34760001	chr16	34,760,001	34,761,000	3	3	3	2	2	2	2	2	2	3	2	3	2	2	2	2	2	2	2	2	2
chr16:34761001	chr16	34,761,001	34,762,000	3	3	3	2	2	2	2	2	2	3	2	3	2	2	2	2	2	2	2	2	2
chr16:34762001	chr16	34,762,001	34,763,000	3	3	3	2	2	2	2	2	2	3	2	3	2	2	2	2	2	2	2	2	2
chr16:34763001	chr16	34,763,001	34,764,000	3	3	3	2	2	2	2	2	2	3	2	3	2	2	2	2	2	2	2	2	2
chr16:34764001	chr16	34,764,001	34,765,000	3	3	3	2	2	2	2	2	2	3	2	3	2	2	2	2	2	2	2	2	2
chr16:34765001	chr16	34,765,001	34,766,000	3	3	3	2	2	2	2	2	2	3	2	3	2	2	2	2	2	2	2	2	2
chr16:34766001	chr16	34,766,001	34,767,000	3	3	3	2	2	2	2	2	2	3	2	3	2	2	2	2	2	2	2	2	2
chr16:34767001	chr16	34,767,001	34,768,000	3	3	3	2	2	2	2	2	2	3	2	3	2	2	2	2	2	2	2	2	2
chr16:34917001	chr16	34,917,001	34,918,000	3	3	3	2	2	2	2	2	2	3	2	3	2	2	2	2	2	2	2	2	2
chr16:34918001	chr16	34,918,001	34,919,000	3	3	3	2	2	2	2	2	2	3	2	3	2	2	2	2	2	2	2	2	2
chr16:34919001	chr16	34,919,001	34,920,000	3	3	3	2	2	2	2	2	2	3	2	3	2	2	2	2	2	2	2	2	2
chr16:34920001	chr16	34,920,001	34,921,000	3	3	3	2	2	2	2	2	2	3	2	3	2	2	2	2	2	2	2	2	2
chr16:36051001	chr16	36,051,001	36,052,000	3	3	3	2	2	2	2	2	2	2	3	3	2	2	2	2	2	2	2	2	2
chr16:36052001	chr16	36,052,001	36,053,000	3	3	3	2	2	2	2	2	2	2	3	3	2	2	2	2	2	2	2	2	2
chr16:36053001	chr16	36,053,001	36,054,000	3	3	3	2	2	2	2	2	2	2	3	3	2	2	2	2	2	2	2	2	2
chr16:36054001	chr16	36,054,001	36,055,000	3	3	3	2	2	2	2	2	2	2	3	3	2	2	2	2	2	2	2	2	2
chr16:36055001	chr16	36,055,001	36,056,000	3	3	3	2	2	2	2	2	2	2	3	3	2	2	2	2	2	2	2	2	2
chr16:36067001	chr16	36,067,001	36,068,000	3	3	3	2	2	2	2	2	2	3	3	2	2	2	2	2	2	2	2	2	2
chr16:36070001	chr16	36,070,001	36,071,000	3	3	3	2	2	2	2	2	2	3	3	2	2	2	2	2	2	2	2	2	2
chr16:36071001	chr16	36,071,001	36,072,000	3	3	3	2	2	2	2	2	2	3	3	2	2	2	2	2	2	2	2	2	2
chr16:36072001	chr16	36,072,001	36,073,000	3	3	3	2	2	2	2	2	2	3	3	2	2	2	2	2	2	2	2	2	2
chr16:36073001	chr16	36,073,001	36,074,000	3	3	3	2	2	2	2	2	2	3	3	2	2	2	2	2	2	2	2	2	2
chr16:36226001	chr16	36,226,001	36,227,000	3	3	3	2	2	2	2	2	2	2	2	3	2	2	2	3	2	2	2	2	2
chr17:21651001	chr17	21,651,001	21,652,000	3	3	3	2	2	2	2	2	2	3	2	2	2	2	2	3	2	2	2	2	2
chr17:21677001	chr17	21,677,001	21,678,000	3	3	3	2	2	2	2	2	2	2	3	2	2	2	2	2	2	2	3	2	2
chr17:21678001	chr17	21,678,001	21,679,000	3	3	3	2	2	2	2	2	2	2	3	2	2	2	2	2	2	2	3	2	2
chr17:21679001	chr17	21,679,001	21,680,000	3	3	3	2	2	2	2	2	2	2	3	2	2	2	2	2	2	2	3	2	2
chr17:21680001	chr17	21,680,001	21,681,000	3	3	3	2	2	2	2	2	2	2	3	2	2	2	2	2	2	2	3	2	2
chr17:21681001	chr17	21,681,001	21,682,000	3	3	3	2	2	2	2	2	2	2	3	2	2	2	2	2	2	2	3	2	2
chr20:31070001	chr20	31,070,001	31,071,000	3	3	3	3	2	2	2	2	2	3	3	2	2	2	2	2	2	2	2	2	2
chr20:31071001	chr20	31,071,001	31,072,000	3	3	3	3	2	2	2	2	2	3	3	2	2	2	2	2	2	2	2	2	2
chr20:31072001	chr20	31,072,001	31,073,000	3	3	3	3	2	2	2	2	2	3	3	2	2	2	2	2	2	2	2	2	2
chr20:31073001	chr20	31,073,001	31,074,000	3	3	3	3	2	2	2	2	2	3	3	2	2	2	2	2	2	2	2	2	2
chr20:31074001	chr20	31,074,001	31,075,000	3	3	3	3	2	2	2	2	2	3	3	2	2	2	2	2	2	2	2	2	2
chr21:8528001	chr21	8,528,001	8,529,000	3	3	3	3	2	2	2	2	2	2	2	3	2	2	2	3	2	2	2	2	2
chr21:8529001	chr21	8,529,001	8,530,000	3	3	3	3	2	2	2	2	2	2	2	3	2	2	2	3	2	2	2	2	2
chr21:8530001	chr21	8,530,001	8,531,000	3	3	3	3	2	2	2	2	2	2	2	3	2	2	2	3	2	2	2	2	2
chr21:8531001	chr21	8,531,001	8,532,000	3	3	3	3	2	2	2	2	2	2	2	3	2	2	2	3	2	2	2	2	2
chr21:9857001	chr21	9,857,001	9,858,000	3	3	3	2	2	2	2	1	2	2	2	2	2	2	2	2	2	3	2	2	3
chr21:9858001	chr21	9,858,001	9,859,000	3	3	3	2	2	2	2	2	2	2	2	2	2	2	2	2	2	3	2	2	3
chr21:9859001	chr21	9,859,001	9,860,000	3	3	3	2	2	2	2	2	2	2	2	2	2	2	2	2	2	3	2	2	3
chr21:9880001	chr21	9,880,001	9,881,000	3	3	3	2	2	2	2	2	2	2	2	2	2	2	2	3	2	2	2	2	3
chr21:9882001	chr21	9,882,001	9,883,000	3	3	3	2	2	2	2	2	2	2	2	3	2	2	2	3	2	2	2	2	2
chr21:9883001	chr21	9,883,001	9,884,000	3	3	3	2	2	2	2	2	2	2	2	3	2	2	2	3	2	2	2	2	2
chr21:9884001	chr21	9,884,001	9,885,000	3	3	3	2	2	2	2	2	2	2	2	3	2	2	2	3	2	2	2	2	2
chr21:9988001	chr21	9,988,001	9,989,000	3	3	3	2	2	2	2	2	2	2	2	2	2	2	2	2	3	2	2	2	3
chr21:10400001	chr21	10,400,001	10,401,000	3	3	3	3	2	2	2	2	2	2	3	2	2	2	2	2	2	2	3	2	2
chr21:10401001	chr21	10,401,001	10,402,000	3	3	3	3	2	2	2	2	2	2	3	2	2	2	2	2	2	2	3	2	2
chr21:10402001	chr21	10,402,001	10,403,000	3	3	3	3	2	2	2	2	2	2	3	2	2	2	2	2	2	2	3	2	2
chr21:10403001	chr21	10,403,001	10,404,000	3	3	3	3	2	2	2	2	2	2	3	2	2	2	2	2	2	2	3	2	2
chr21:10404001	chr21	10,404,001	10,405,000	3	3	3	3	2	2	2	2	2	2	3	2	2	2	2	2	2	2	3	2	2
chr21:10405001	chr21	10,405,001	10,406,000	3	3	3	3	2	2	2	2	2	2	3	2	2	2	2	2	2	2	3	2	2
chr21:10406001	chr21	10,406,001	10,407,000	3	3	3	3	2	2	2	2	2	2	3	2	2	2	2	2	2	2	3	2	2
chr21:10407001	chr21	10,407,001	10,408,000	3	3	3	3	2	2	2	2	2	2	3	2	2	2	2	2	2	2	3	2	2
chr21:10408001	chr21	10,408,001	10,409,000	3	3	3	3	2	2	2	2	2	2	3	2	2	2	2	2	2	2	3	2	2
chr21:10409001	chr21	10,409,001	10,410,000	3	3	3	3	2	2	2	2	2	2	3	2	2	2	2	2	2	2	3	2	2
chr21:10410001	chr21	10,410,001	10,411,000	3	3	3	3	2	2	2	2	2	2	3	2	2	2	2	2	2	2	3	2	2
chr21:10411001	chr21	10,411,001	10,412,000	3	3	3	3	2	2	2	2	2	2	3	2	2	2	2	2	2	2	3	2	2
chr21:10412001	chr21	10,412,001	10,413,000	3	3	3	3	2	2	2	2	2	2	3	2	2	2	2	2	2	2	3	2	2
chr21:10413001	chr21	10,413,001	10,414,000	3	3	3	3	2	2	2	2	2	2	3	2	2	2	2	2	2	2	3	2	2
chr21:10653001	chr21	10,653,001	10,654,000	3	3	3	2	2	2	2	2	2	2	2	2	2	2	2	3	3	2	2	2	2
chr21:10654001	chr21	10,654,001	10,655,000	3	3	3	2	2	2	2	2	2	2	2	2	2	2	2	3	3	2	2	2	2
chr21:10655001	chr21	10,655,001	10,656,000	3	3	3	2	2	2	2	2	2	2	2	2	2	2	2	3	3	2	2	2	2
chr21:10750001	chr21	10,750,001	10,751,000	3	3	3	2	2	2	2	2	2	2	2	2	2	2	2	3	2	2	2	2	3
chr22:10540001	chr22	10,540,001	10,541,000	3	3	3	2	2	2	2	2	2	3	2	2	2	2	2	3	2	2	2	2	2
chr22:10541001	chr22	10,541,001	10,542,000	3	3	3	2	2	2	2	2	2	3	2	2	2	2	2	3	2	2	2	2	2
chr22:10542001	chr22	10,542,001	10,543,000	3	3	3	2	2	2	2	2	2	3	2	2	2	2	2	3	2	2	2	2	2
chr22:10543001	chr22	10,543,001	10,544,000	3	3	3	2	2	2	2	2	2	3	2	2	2	2	2	3	2	2	2	2	2
chr22:10544001	chr22	10,544,001	10,545,000	3	3	3	2	2	2	2	1	2	3	2	2	2	2	2	3	2	2	2	2	2
chr22:10545001	chr22	10,545,001	10,546,000	3	3	3	2	2	2	2	2	2	3	2	2	2	2	2	3	2	2	2	2	2
chr22:10554001	chr22	10,554,001	10,555,000	3	3	3	2	2	2	2	2	2	3	2	2	2	2	2	3	2	2	2	2	2
chr22:10555001	chr22	10,555,001	10,556,000	3	3	3	2	2	2	2	2	2	3	2	2	2	2	2	3	2	2	2	2	2
chr22:10556001	chr22	10,556,001	10,557,000	3	3	3	2	2	2	2	2	2	3	2	2	2	2	2	3	2	2	2	2	2
chr22:10557001	chr22	10,557,001	10,558,000	3	3	3	2	2	2	2	2	2	3	2	2	2	2	2	3	2	2	2	2	2
chr22:10558001	chr22	10,558,001	10,559,000	3	3	3	2	2	2	2	2	2	3	2	2	2	2	2	3	2	2	2	2	2
chr22:10559001	chr22	10,559,001	10,560,000	3	3	3	2	2	2	2	2	2	3	2	2	2	2	2	3	2	2	2	2	2
chr22:10560001	chr22	10,560,001	10,561,000	3	3	3	2	2	2	2	2	2	3	2	2	2	2	2	3	2	2	2	2	2
chr22:10609001	chr22	10,609,001	10,610,000	4	3	3	2	2	3	2	2	2	3	2	2	2	3	2	3	2	2	2	2	1

Region	CHR	start	end	HD2	HD3	HD1	BN1	BN2	BN3	BN4	BN5	BN6	LC1	LC2	LC3	LC4	LC5	LC6	LC7	LC8	LC9	LC10	LC10*	LC11
chr22:10610001	chr22	10,610,001	10,611,000	4	3	3	2	2	3	2	2	2	3	2	2	2	3	2	3	2	2	2	2	1
chr22:10626001	chr22	10,626,001	10,627,000	3	3	3	2	2	2	2	2	2	2	2	2	2	3	2	3	2	2	2	2	2
chr22:10627001	chr22	10,627,001	10,628,000	3	3	3	2	2	2	2	2	2	2	2	2	2	3	2	3	2	2	2	2	2
chr22:10628001	chr22	10,628,001	10,629,000	4	3	3	2	2	2	2	2	2	2	2	2	2	3	2	3	2	2	2	2	2
chr22:10633001	chr22	10,633,001	10,634,000	4	3	3	2	2	3	2	2	2	3	2	2	2	3	2	3	2	2	2	2	1
chr22:10634001	chr22	10,634,001	10,635,000	4	3	3	2	2	3	2	2	2	3	2	2	2	3	2	3	2	2	2	2	1
chr22:10717001	chr22	10,717,001	10,718,000	3	3	3	3	2	2	2	2	2	3	3	2	2	2	2	2	2	2	2	2	2
chr1:502001	chr1	502,001	503,000	2	2	2	3	3	3	2	3	3	2	2	2	3	2	2	3	2	2	2	2	2
chr1:759001	chr1	759,001	760,000	2	2	2	3	3	3	3	2	3	2	2	2	2	2	2	3	3	2	2	2	2
chr1:760001	chr1	760,001	761,000	2	2	2	3	3	3	3	2	3	2	2	2	2	2	2	3	3	2	2	2	2
chr5:70550001	chr5	70,550,001	70,551,000	2	2	2	3	3	3	3	2	3	2	3	3	2	2	2	2	2	2	2	2	2
chr5:70712001	chr5	70,712,001	70,713,000	2	2	2	3	3	3	3	2	3	2	3	3	2	2	2	2	2	2	2	2	2
chr6:130001	chr6	130,001	131,000	1	2	2	2	2	4	4	3	3	2	2	2	3	2	3	2	2	2	2	2	2
chr6:131001	chr6	131,001	132,000	1	2	2	2	2	4	4	3	3	2	2	2	3	2	3	2	2	2	2	2	2
chr6:136001	chr6	136,001	137,000	2	2	2	2	3	3	3	3	3	2	2	2	3	2	3	2	2	2	2	2	2
chr6:137001	chr6	137,001	138,000	2	2	2	2	3	3	3	3	3	2	2	2	3	2	3	2	2	2	2	2	2
chr6:138001	chr6	138,001	139,000	2	2	2	2	3	3	3	3	3	2	2	2	3	2	3	2	2	2	2	2	2
chr6:139001	chr6	139,001	140,000	2	2	2	2	3	3	3	3	3	2	2	2	3	2	3	2	2	2	2	2	2
chr6:397001	chr6	397,001	398,000	2	2	2	2	3	3	3	3	3	2	2	2	2	2	3	3	2	2	2	2	2
chr9:60558001	chr9	60,558,001	60,559,000	5	5	2	2	2	2	2	3	3	3	2	2	2	2	2	3	2	2	2	2	2
chr11:96445001	chr11	96,445,001	96,446,000	5	5	2	2	2	2	2	2	2	2	3	2	2	2	2	3	2	2	2	2	2
chr14:106274001	chr14	106,274,001	106,275,000	2	2	2	2	3	3	3	3	3	2	2	2	2	2	3	3	2	2	2	2	2
chr14:106275001	chr14	106,275,001	106,276,000	2	2	2	2	3	3	3	3	3	2	2	2	2	2	3	3	2	2	2	2	2
chr14:106276001	chr14	106,276,001	106,277,000	2	2	2	2	3	3	3	3	3	2	2	2	2	2	3	3	2	2	2	2	2
chr15:101139001	chr15	101,139,001	101,140,000	2	2	2	2	3	3	3	3	3	2	2	2	2	2	3	3	2	2	2	2	2
chr15:101156001	chr15	101,156,001	101,157,000	2	2	2	2	3	3	3	3	3	2	2	2	3	2	3	2	2	2	2	2	2
chr15:101345001	chr15	101,345,001	101,346,000	2	2	2	2	3	3	3	3	3	2	2	2	2	3	3	2	2	2	2	2	2
chr15:101346001	chr15	101,346,001	101,347,000	2	2	2	2	3	3	3	3	3	2	2	2	2	3	3	2	2	2	2	2	2
chr15:101347001	chr15	101,347,001	101,348,000	2	2	2	2	3	3	3	3	3	2	2	2	2	3	3	2	2	2	2	2	2
chr15:101348001	chr15	101,348,001	101,349,000	2	2	2	2	3	3	3	3	3	2	2	2	2	3	3	2	2	2	2	2	2
chr15:101349001	chr15	101,349,001	101,350,000	2	2	2	2	3	3	3	3	3	2	2	2	2	3	3	2	2	2	2	2	2
chr15:101350001	chr15	101,350,001	101,351,000	2	2	2	2	3	3	3	3	3	2	2	2	2	3	3	2	2	2	2	2	2
chr15:101351001	chr15	101,351,001	101,352,000	2	2	2	2	3	3	3	3	3	2	2	2	2	3	3	2	2	2	2	2	2
chr15:101352001	chr15	101,352,001	101,353,000	2	2	2	2	3	3	3	3	3	2	2	2	2	3	3	2	2	2	2	2	2
chr15:101408001	chr15	101,408,001	101,409,000	2	2	2	2	3	3	3	3	3	2	2	3	2	2	3	2	2	2	2	2	2
chr15:101409001	chr15	101,409,001	101,410,000	2	2	2	2	3	3	3	3	3	2	2	3	2	2	3	2	2	2	2	2	2
chr15:101410001	chr15	101,410,001	101,411,000	2	2	2	2	3	3	3	3	3	2	2	3	2	2	3	2	2	2	2	2	2
chr15:101436001	chr15	101,436,001	101,437,000	2	2	2	2	3	3	3	3	3	2	2	2	2	2	3	3	2	2	2	2	2
chr15:101437001	chr15	101,437,001	101,438,000	2	2	2	2	3	3	3	3	3	2	2	2	2	2	3	3	2	2	2	2	2
chr15:101438001	chr15	101,438,001	101,439,000	2	2	2	2	3	3	3	3	3	2	2	2	2	2	3	3	2	2	2	2	2
chr15:101439001	chr15	101,439,001	101,440,000	2	2	2	2	3	3	3	3	3	2	2	2	2	2	3	3	2	2	2	2	2
chr15:101475001	chr15	101,475,001	101,476,000	2	2	2	2	3	3	3	3	3	2	2	2	2	2	3	2	3	2	2	2	2
chr15:101479001	chr15	101,479,001	101,480,000	2	2	2	2	3	3	3	3	3	2	2	2	2	2	3	3	2	2	2	2	2
chr15:101480001	chr15	101,480,001	101,481,000	2	2	2	2	3	3	3	3	3	2	2	2	2	2	3	3	2	2	2	2	2
chr15:101557001	chr15	101,557,001	101,558,000	2	2	2	2	3	3	3	3	3	2	2	2	2	2	3	2	2	2	2	3	2
chr15:101566001	chr15	101,566,001	101,567,000	2	2	2	2	3	3	3	3	3	2	2	2	2	2	3	3	2	2	2	2	2
chr15:101567001	chr15	101,567,001	101,568,000	2	2	2	2	3	3	3	3	3	2	2	2	2	2	3	3	2	2	2	2	2
chr15:101568001	chr15	101,568,001	101,569,000	2	2	2	2	3	3	3	3	3	2	2	2	2	2	3	3	2	2	2	2	2
chr15:101573001	chr15	101,573,001	101,574,000	2	2	2	2	3	3	3	3	3	2	2	2	2	2	3	3	2	2	2	2	2
chr15:101574001	chr15	101,574,001	101,575,000	2	2	2	2	3	3	3	3	3	2	2	2	2	2	3	3	2	2	2	2	2
chr15:101575001	chr15	101,575,001	101,576,000	2	2	2	2	3	3	3	3	3	2	2	2	2	2	3	3	2	2	2	2	2
chr15:101576001	chr15	101,576,001	101,577,000	2	2	2	2	3	3	3	3	3	2	2	2	2	2	3	3	2	2	2	2	2
chr15:101577001	chr15	101,577,001	101,578,000	2	2	2	2	3	3	3	3	3	2	2	2	2	2	3	3	2	2	2	2	2
chr15:101651001	chr15	101,651,001	101,652,000	2	2	2	2	3	3	3	3	3	2	2	2	3	2	3	2	2	2	2	2	2
chr15:101663001	chr15	101,663,001	101,664,000	2	2	2	2	3	3	3	3	3	2	2	2	3	2	3	2	2	2	2	2	2
chr15:101664001	chr15	101,664,001	101,665,000	2	2	2	2	3	3	3	3	3	2	2	2	3	2	3	2	2	2	2	2	2
chr15:101665001	chr15	101,665,001	101,666,000	2	2	2	2	3	3	3	3	3	2	2	2	3	2	3	2	2	2	2	2	2
chr16:140001	chr16	140,001	141,000	2	2	2	2	3	3	3	3	3	2	2	2	3	2	3	2	2	2	2	2	2
chr16:141001	chr16	141,001	142,000	2	2	2	2	3	3	3	3	3	2	2	2	3	2	3	2	2	2	2	2	2
chr16:142001	chr16	142,001	143,000	2	2	2	2	3	3	3	3	3	2	2	2	3	2	3	2	2	2	2	2	2
chr16:143001	chr16	143,001	144,000	2	2	2	2	3	3	3	3	3	2	2	2	3	2	3	2	2	2	2	2	2
chr16:150001	chr16	150,001	151,000	2	2	2	2	3	3	3	3	3	2	2	2	2	2	3	3	2	2	2	2	2
chr16:178001	chr16	178,001	179,000	2	2	2	2	3	3	3	3	3	2	2	2	2	3	3	2	2	2	2	2	2
chr16:179001	chr16	179,001	180,000	2	2	2	2	3	3	3	3	3	2	2	2	2	3	3	2	2	2	2	2	2
chr16:180001	chr16	180,001	181,000	2	2	2	2	3	3	3	3	3	2	2	2	2	3	3	2	2	2	2	2	2
chr16:181001	chr16	181,001	182,000	2	2	2	2	3	3	3	3	3	2	2	2	2	3	3	2	2	2	2	2	2
chr16:182001	chr16	182,001	183,000	2	2	2	2	3	3	3	3	3	2	2	2	2	3	3	2	2	2	2	2	2
chr21:44109001	chr21	44,109,001	44,110,000	2	2	2	2	2	3	4	4	4	2	3	2	2	3	2	2	2	2	2	2	2
chr21:44110001	chr21	44,110,001	44,111,000	2	2	2	3	3	3	4	4	4	2	3	2	2	3	2	2	2	2	2	2	2
chr21:44115001	chr21	44,115,001	44,116,000	2	2	2	3	3	3	4	4	3	2	2	2	2	3	3	2	2	2	2	2	2
chr21:44116001	chr21	44,116,001	44,117,000	2	2	2	3	3	3	4	4	3	2	2	2	2	3	3	2	2	2	2	2	2
chr21:44117001	chr21	44,117,001	44,118,000	2	2	2	3	3	3	4	4	3	2	2	2	2	3	3	2	2	2	2	2	2
chr21:44118001	chr21	44,118,001	44,119,000	2	2	2	3	3	3	4	4	3	2	2	2	2	3	3	2	2	2	2	2	2
chr21:44122001	chr21	44,122,001	44,123,000	2	2	2	3	3	3	3	3	3	2	2	2	2	3	3	2	2	2	2	2	2
chr21:44123001	chr21	44,123,001	44,124,000	2	2	2	3	3	3	3	3	3	2	2	2	2	3	3	2	2	2	2	2	2
chr21:44124001	chr21	44,124,001	44,125,000	2	2	2	3	3	3	3	3	3	2	2	2	2	3	3	2	2	2	2	2	2
chr21:44130001	chr21	44,130,001	44,131,000	2	2	2	3	3	3	3	3	3	1	3	2	2	3	3	2	2	2	2	2	2
chr21:44131001	chr21	44,131,001	44,132,000	2	2	2	3	3	3	3	3	3	1	3	2	2	3	3	2	2	2	2	2	2
chr21:44132001	chr21	44,132,001	44,133,000	2	2	2	3	3	3	3	3	3	1	3	2	2	3	3	2	2	2	2	2	2
chr21:44155001	chr21	44,155,001	44,156,000	2	2	2	3	3	2	4	4	3	2	2	2	2	3	3	2	2	2	2	2	2
chr21:44156001	chr21	44,156,001	44,157,000	2	2	2	3	3	2	4	4	3	2	2	2	2	3	3	2	2	2	2	2	2
chr15:101828001	chr15	101,828,001	101,829,000	2	2	1	2	3	3	3	3	3	2	2	2	2	2	3	2	2	2	2	2	3
chr15:101831001	chr15	101,831,001	101,832,000	2	2	1	2	3	3	3	3	3	2	2	2	2	2	3	3	2	2	2	2	2
chr15:101832001	chr15	101,832,001	101,833,000	2	2	1	2	3	3	3	3	3	2	2	2	2	2	3	3	2	2	2	2	2
chr15:101833001	chr15	101,833,001	101,834,000	2	2	1	2	3	3	3	3	3	2	2	2	2	2	3	3	2	2	2	2	2
chr15:101834001	chr15	101,834,001	101,835,000	2	2	1	2	3	3	3	3	3	2	2	2	2	2	3	3	2	2	2	2	2
chr15:101835001	chr15	101,835,001	101,836,000	2	2	1	2	3	3	3	3	3	2	2	2	2	2	3	3	2	2	2	2	2
chr21:44142001	chr21	44,142,001	44,143,000	2	2	1	3	3	3	3	4	3	2	3	2	1	3	3	2	2	2	2	2	2
chr21:44143001	chr21	44,143,001	44,144,000	2	2	1	3	3	3	3	4	3	2	3	2	1	3	3	2	2	2	2	2	2
chr21:44144001	chr21	44,144,001	44,145,000	2	2	1	3	3	3	4	4	3	2	3	2	1	3	3	2	2	2	2	2	2
chr21:44145001	chr21	44,145,001	44,146,000	2	2	1	3	3	3	4	4	3	2	3	2	1	3	3	2	2	2	2	2	2
chr21:44148001	chr21	44,148,001	44,149,000	2	2	1	3	3	3	4	4	3	2	3	2	2	3	3	2	2	2	2	1	2
chr21:44149001	chr21	44,149,001	44,150,000	2	2	1	3	3	3	4	4	3	2	3	2	2	3	3	2	2	2	2	1	2
chr21:44150001	chr21	44,150,001	44,151,000	2	2	1	3	3	3	4	4	3	2	3	2	2	3	3	2	2	2	2	1	2
chr21:44151001	chr21	44,151,001	44,152,000	2	2	1	3	3	3	4	4	3	2	2	2	2	3	3	2	2	2	2	2	2
chr21:44152001	chr21	44,152,001	44,153,000	2	2	1	3	3	3	4	4	3	2	2	2	2	3	3	2	2	2	2	2	2
chr21:44153001	chr21	44,153,001	44,154,000	2	2	1	3	3	3	4	4	3	2	2	2	2	3	3	2	2	2	2	2	2
chr21:44154001	chr21	44,154,001	44,155,000	2	2	1	3	3	3	4	4	3	2	2	2	2	3	3	2	2	2	2	2	2
chr10:114138001	chr10	114,138,001	114,139,000	4	5	5	2	2	2	2	2	2	2	2	2	2	2	2	2	2	2	3	2	2
chr21:5250001	chr21	5,250,001	5,251,000	5	2	5	4	2	2	2	2	2	2	2	2	2	2	2	2	2	3	2	2	2
chr21:5251001	chr21	5,251,001	5,252,000	5	2	5	4	2	2	2	2	2	2	2	2	2	2	2	2	2	3	2	2	2
chr3:198169001	chr3	198,169,001	198,170,000	4	3	4	3	2	3	2	2	3	1	2	3	2	2	3	2	1	3	2	2	2
chr4:33842001	chr4	33,842,001	33,843,000	3	3	4	2	2	2	2	2	2	2	2	3	2	2	2	2	2	2	2	2	2
chr9:60604001	chr9	60,604,001	60,605,000	3	3	4	2	2	2	1	3	2	2	3	2	2	2	2	3	1	2	2	2	2
chr11:133001	chr11	133,001	134,000	2	3	4	2	2	2	2	2	2	2	2	2	2	3	2	2	2	2	2	2	2
chr11:134001	chr11	134,001	135,000	2	3	4	2	2	2	2	2	2	2	2	2	2	3	2	2	2	2	2	2	2
chr13:18483001	chr13	18,483,001	18,484,000	3	4	4	2	2	3	2	2	3	2	2	2	2	2	2	2	2	2	2	2	3
chr13:18484001	chr13	18,484,001	18,485,000	3	4	4	2	2	3	2	2	3	2	2	2	2	2	2	2	2	2	2	2	3
chr13:18485001	chr13	18,485,001	18,486,000	3	4	4	2	2	3	2	2	3	2	2	2	2	2	2	2	2	2	2	2	3
chr13:18486001	chr13	18,486,001	18,487,000	3	4	4	2	2	3	2	2	3	2	2	2	2	2	2	2	2	2	2	2	3
chr13:18487001	chr13	18,487,001	18,488,000	3	4	4	2	2	3	2	2	3	2	2	2	2	2	2	2	2	2	2	2	3
chr13:18488001	chr13	18,488,001	18,489,000	3	4	4	2	2	3	2	2	3	2	2	2	2	2	2	2	2	2	2	2	3
chr13:18490001	chr13	18,490,001	18,491,000	3	4	4	2	2	3	2	2	3	2	2	2	2	2	2	2	2	2	2	2	3

Region	CHR	start	end	HD2	HD3	HD1	BN1	BN2	BN3	BN4	BN5	BN6	LC1	LC2	LC3	LC4	LC5	LC6	LC7	LC8	LC9	LC10	LC10*	LC11
chr15:21995001	chr15	21,995,001	21,996,000	3	4	4	2	3	2	2	2	3	2	2	2	2	2	3	3	2	1	2	2	2
chr2:91808001	chr2	91,808,001	91,809,000	3	3	3	2	2	2	2	2	3	2	2	3	2	2	2	2	2	2	2	2	2
chr7:57618001	chr7	57,618,001	57,619,000	3	3	3	2	3	2	2	2	2	2	2	2	2	2	2	3	2	2	2	2	2
chr7:57626001	chr7	57,626,001	57,627,000	3	3	3	2	3	2	2	2	2	2	2	2	2	2	2	3	2	2	2	2	2
chr7:57627001	chr7	57,627,001	57,628,000	3	3	3	2	3	2	2	2	2	2	2	2	2	2	2	3	2	2	2	2	2
chr7:65151001	chr7	65,151,001	65,152,000	3	3	3	2	2	2	2	2	2	2	2	2	2	3	2	2	2	2	2	2	2
chr7:65152001	chr7	65,152,001	65,153,000	3	3	3	2	2	2	2	2	2	2	2	2	2	3	2	2	2	2	2	2	2
chr7:65153001	chr7	65,153,001	65,154,000	3	3	3	2	2	2	2	2	2	2	2	2	2	3	2	2	2	2	2	2	2
chr9:40032001	chr9	40,032,001	40,033,000	3	3	3	3	3	3	2	3	2	2	2	2	2	3	2	2	2	2	2	2	2
chr9:41727001	chr9	41,727,001	41,728,000	3	3	3	2	2	2	1	2	2	2	2	2	2	2	2	3	2	2	2	2	2
chr9:41728001	chr9	41,728,001	41,729,000	3	3	3	2	2	2	1	2	2	2	2	2	2	2	2	3	2	2	2	2	2
chr9:43291001	chr9	43,291,001	43,292,000	3	3	3	2	2	2	2	2	2	2	2	2	2	2	2	2	2	2	3	2	2
chr9:43296001	chr9	43,296,001	43,297,000	3	3	3	2	2	2	2	2	2	2	2	3	1	2	2	2	2	2	3	2	2
chr9:43297001	chr9	43,297,001	43,298,000	3	3	3	2	2	2	2	2	2	2	2	3	1	2	2	2	2	2	3	2	2
chr9:43298001	chr9	43,298,001	43,299,000	3	3	3	2	2	2	2	2	2	2	2	3	1	2	2	2	2	2	3	2	2
chr9:43299001	chr9	43,299,001	43,300,000	3	3	3	2	2	2	2	2	2	2	2	3	1	2	2	2	2	2	3	2	2
chr9:43300001	chr9	43,300,001	43,301,000	3	3	3	2	2	2	2	2	2	2	2	3	1	2	2	2	2	2	3	2	2
chr9:43301001	chr9	43,301,001	43,302,000	3	3	3	2	2	2	2	2	2	2	2	3	1	2	2	2	2	2	3	2	2
chr9:61891001	chr9	61,891,001	61,892,000	3	3	3	2	2	2	1	2	2	2	2	2	2	2	2	3	2	2	2	2	2
chr9:61892001	chr9	61,892,001	61,893,000	3	3	3	2	2	2	1	2	2	2	2	2	2	2	2	3	2	2	2	2	2
chr9:61893001	chr9	61,893,001	61,894,000	3	3	3	2	2	2	1	2	2	2	2	2	2	2	2	3	2	2	2	2	2
chr9:61894001	chr9	61,894,001	61,895,000	3	3	3	2	2	2	1	2	2	2	2	2	2	2	2	3	2	2	2	2	2
chr9:61895001	chr9	61,895,001	61,896,000	3	3	3	2	2	2	1	2	2	2	2	2	2	2	2	3	2	2	2	2	2
chr9:64333001	chr9	64,333,001	64,334,000	2	3	3	2	2	2	1	1	2	2	3	2	2	2	2	2	2	2	2	2	2
chr9:64852001	chr9	64,852,001	64,853,000	3	3	3	2	2	2	1	1	2	2	3	2	2	2	1	3	2	2	2	2	2
chr9:64853001	chr9	64,853,001	64,854,000	3	3	3	2	2	2	1	1	2	2	3	2	2	2	1	3	2	2	2	2	2
chr9:64854001	chr9	64,854,001	64,855,000	3	3	3	2	2	2	1	1	2	2	3	2	2	2	1	3	2	2	2	2	2
chr9:64855001	chr9	64,855,001	64,856,000	3	3	3	2	2	2	1	1	2	2	3	2	2	2	1	3	2	2	2	2	2
chr9:67431001	chr9	67,431,001	67,432,000	4	3	3	3	2	2	2	2	2	2	3	2	2	2	2	2	2	2	2	2	2
chr9:67432001	chr9	67,432,001	67,433,000	4	3	3	2	2	2	2	2	2	2	3	2	2	2	2	2	2	2	2	2	2
chr9:67433001	chr9	67,433,001	67,434,000	4	3	3	2	2	2	2	2	2	2	3	2	2	2	2	2	2	2	2	2	2
chr10:38970001	chr10	38,970,001	38,971,000	3	3	3	2	2	2	2	2	2	2	2	2	2	2	2	2	2	2	3	2	2
chr10:38971001	chr10	38,971,001	38,972,000	3	3	3	2	2	2	2	2	2	2	2	2	2	2	2	2	2	2	3	2	2
chr13:18424001	chr13	18,424,001	18,425,000	3	3	3	2	2	2	2	2	2	2	2	2	2	2	2	2	2	2	2	2	3
chr13:18425001	chr13	18,425,001	18,426,000	3	3	3	2	2	2	2	2	2	2	2	2	2	2	2	2	2	2	2	2	3
chr13:18426001	chr13	18,426,001	18,427,000	3	3	3	2	2	2	2	2	2	2	2	2	2	2	2	2	2	2	2	2	3
chr13:18427001	chr13	18,427,001	18,428,000	3	3	3	2	2	2	2	2	2	2	2	2	2	2	2	2	2	2	2	2	3
chr13:18428001	chr13	18,428,001	18,429,000	3	3	3	2	2	2	2	2	2	2	2	2	2	2	2	2	2	2	2	2	3
chr13:18429001	chr13	18,429,001	18,430,000	3	3	3	2	2	3	2	2	2	2	2	2	2	2	2	2	2	2	2	2	3
chr13:18430001	chr13	18,430,001	18,431,000	3	3	3	2	2	3	2	2	2	2	2	2	2	2	2	2	2	2	2	2	3
chr13:18431001	chr13	18,431,001	18,432,000	3	3	3	2	2	3	2	2	2	2	2	2	2	2	2	2	2	2	2	2	3
chr13:18432001	chr13	18,432,001	18,433,000	3	3	3	2	2	3	2	2	2	2	2	2	2	2	2	2	2	2	2	2	3
chr13:18433001	chr13	18,433,001	18,434,000	3	3	3	2	2	3	2	2	2	2	2	2	2	2	2	2	2	2	2	2	3
chr13:18434001	chr13	18,434,001	18,435,000	3	3	3	2	2	3	2	2	2	2	2	2	2	2	2	2	2	2	2	2	3
chr14:18529001	chr14	18,529,001	18,530,000	3	3	3	2	2	2	2	2	2	2	2	3	2	1	2	2	2	2	3	2	2
chr14:18530001	chr14	18,530,001	18,531,000	3	3	3	2	2	2	2	2	2	2	2	3	2	1	2	2	2	2	3	2	2
chr16:34101001	chr16	34,101,001	34,102,000	3	3	3	2	2	2	2	2	2	3	2	2	2	2	2	2	2	2	2	2	2
chr16:34102001	chr16	34,102,001	34,103,000	3	3	3	2	2	2	2	2	2	3	2	2	2	2	2	2	2	2	2	2	2
chr16:34103001	chr16	34,103,001	34,104,000	3	3	3	2	2	2	2	2	2	3	2	2	2	2	2	2	2	2	2	2	2
chr16:34104001	chr16	34,104,001	34,105,000	3	3	3	2	2	2	2	2	2	3	2	2	2	2	2	2	2	2	2	2	2
chr16:34105001	chr16	34,105,001	34,106,000	3	3	3	2	2	2	2	2	2	3	2	2	2	2	2	2	2	2	2	2	2
chr16:34106001	chr16	34,106,001	34,107,000	3	3	3	2	2	2	2	2	2	3	2	2	2	2	2	2	2	2	2	2	2
chr16:34107001	chr16	34,107,001	34,108,000	3	3	3	2	2	2	2	2	2	3	2	2	2	2	2	2	2	2	2	2	2
chr16:34150001	chr16	34,150,001	34,151,000	3	3	3	2	2	2	2	2	2	2	2	3	2	2	2	2	2	2	2	2	2
chr16:34263001	chr16	34,263,001	34,264,000	3	3	3	3	2	1	2	2	2	2	3	2	2	2	2	2	2	2	2	2	2
chr16:34264001	chr16	34,264,001	34,265,000	3	3	3	3	2	1	2	2	2	2	3	2	2	2	2	2	2	2	2	2	2
chr16:34265001	chr16	34,265,001	34,266,000	3	3	3	3	2	1	2	2	2	2	3	2	2	2	2	2	2	2	2	2	2
chr16:34266001	chr16	34,266,001	34,267,000	3	3	3	3	2	2	2	2	2	2	3	2	2	2	2	2	2	2	2	2	2
chr16:34748001	chr16	34,748,001	34,749,000	3	3	3	2	2	2	2	2	2	2	2	3	2	2	2	2	2	2	2	2	2
chr16:34749001	chr16	34,749,001	34,750,000	3	3	3	2	2	2	2	2	2	2	2	3	2	2	2	2	2	2	2	2	2
chr16:36056001	chr16	36,056,001	36,057,000	3	3	3	2	2	2	2	2	2	2	3	2	2	2	2	2	2	2	2	2	2
chr16:36057001	chr16	36,057,001	36,058,000	3	3	3	2	2	2	2	2	2	2	3	2	2	2	2	2	2	2	2	2	2
chr16:36058001	chr16	36,058,001	36,059,000	3	3	3	2	2	2	2	2	2	2	3	2	2	2	2	2	2	2	2	2	2
chr16:36059001	chr16	36,059,001	36,060,000	3	3	3	2	2	2	2	2	2	2	3	2	2	2	2	2	2	2	2	2	2
chr16:36060001	chr16	36,060,001	36,061,000	3	3	3	2	2	2	2	2	2	2	3	2	2	2	2	2	2	2	2	2	2
chr16:36061001	chr16	36,061,001	36,062,000	3	3	3	2	2	2	2	2	2	2	3	2	2	2	2	2	2	2	2	2	2
chr16:36062001	chr16	36,062,001	36,063,000	3	3	3	2	2	2	2	2	2	2	3	2	2	2	2	2	2	2	2	2	2
chr16:36063001	chr16	36,063,001	36,064,000	3	3	3	2	2	2	2	2	2	2	3	2	2	2	2	2	2	2	2	2	2
chr16:36064001	chr16	36,064,001	36,065,000	3	3	3	2	2	2	2	2	2	2	3	2	2	2	2	2	2	2	2	2	2
chr16:36065001	chr16	36,065,001	36,066,000	3	3	3	2	2	2	2	2	2	2	3	2	2	2	2	2	2	2	2	2	2
chr16:36066001	chr16	36,066,001	36,067,000	3	3	3	2	2	2	2	2	2	2	3	2	2	2	2	2	2	2	2	2	2
chr16:36068001	chr16	36,068,001	36,069,000	3	3	3	2	2	2	2	2	2	3	3	2	2	2	2	2	1	2	2	2	2
chr16:36069001	chr16	36,069,001	36,070,000	3	3	3	2	2	2	2	2	2	3	3	2	2	2	2	2	1	2	2	2	2
chr16:36252001	chr16	36,252,001	36,253,000	3	3	3	2	2	2	2	2	2	2	3	2	2	2	2	2	2	2	2	2	2
chr16:36253001	chr16	36,253,001	36,254,000	3	3	3	2	2	2	2	2	2	2	3	2	2	2	2	2	2	2	2	2	2
chr17:21649001	chr17	21,649,001	21,650,000	3	3	3	2	2	2	2	2	2	2	2	2	2	2	2	3	2	2	2	2	2
chr17:21650001	chr17	21,650,001	21,651,000	3	3	3	2	2	2	2	2	2	2	2	2	2	2	2	3	2	2	2	2	2
chr17:21682001	chr17	21,682,001	21,683,000	3	3	3	2	2	2	2	2	2	2	3	2	2	2	2	2	2	2	2	2	2
chr21:7230001	chr21	7,230,001	7,231,000	3	3	3	2	2	2	2	2	2	2	2	3	2	2	2	2	2	2	2	2	2
chr21:7231001	chr21	7,231,001	7,232,000	3	3	3	2	2	2	2	2	2	2	2	3	2	2	2	2	2	2	2	2	2
chr21:7232001	chr21	7,232,001	7,233,000	3	3	3	2	2	2	2	2	2	2	2	3	2	2	2	2	2	2	2	2	2
chr21:7233001	chr21	7,233,001	7,234,000	3	3	3	2	2	2	2	2	2	2	2	3	2	2	2	2	2	2	2	2	2
chr21:7234001	chr21	7,234,001	7,235,000	3	3	3	2	2	2	2	2	2	2	2	3	2	2	2	2	2	2	2	2	2
chr21:7235001	chr21	7,235,001	7,236,000	3	3	3	2	2	2	2	2	2	2	2	3	2	2	2	2	2	2	2	2	2
chr21:7236001	chr21	7,236,001	7,237,000	3	3	3	2	2	2	2	2	2	2	2	3	2	2	2	2	2	2	2	2	2
chr21:7237001	chr21	7,237,001	7,238,000	3	3	3	2	2	2	2	2	2	2	2	3	2	2	2	2	2	2	2	2	2
chr21:7238001	chr21	7,238,001	7,239,000	3	3	3	2	2	2	2	2	2	2	2	3	2	2	2	2	2	2	2	2	2
chr21:7239001	chr21	7,239,001	7,240,000	3	3	3	2	2	2	2	2	2	2	2	3	2	2	2	2	2	2	2	2	2
chr21:7240001	chr21	7,240,001	7,241,000	3	3	3	2	2	2	2	2	2	2	2	3	2	2	2	2	2	2	2	2	2
chr21:7241001	chr21	7,241,001	7,242,000	3	3	3	2	2	2	2	2	2	2	2	3	2	2	2	2	2	2	2	2	2
chr21:7242001	chr21	7,242,001	7,243,000	3	3	3	2	2	2	2	2	2	2	2	3	2	2	2	2	2	2	2	2	2
chr21:7243001	chr21	7,243,001	7,244,000	3	3	3	2	2	2	2	2	2	2	2	3	2	2	2	2	2	2	2	2	2
chr21:7244001	chr21	7,244,001	7,245,000	3	3	3	2	2	2	2	2	2	2	2	3	2	2	2	2	2	2	2	2	2
chr21:7245001	chr21	7,245,001	7,246,000	3	3	3	2	2	2	2	2	2	2	2	3	2	2	2	2	2	2	2	2	2
chr21:8523001	chr21	8,523,001	8,524,000	5	3	3	2	2	2	2	1	2	2	2	2	2	2	2	3	2	2	2	2	2
chr21:8524001	chr21	8,524,001	8,525,000	5	3	3	2	2	2	2	1	2	2	2	2	2	2	2	3	2	2	2	2	2
chr21:8525001	chr21	8,525,001	8,526,000	3	3	3	2	2	2	2	1	2	2	2	2	2	2	2	3	2	2	2	2	2
chr21:8526001	chr21	8,526,001	8,527,000	3	3	3	2	2	2	2	1	2	2	2	2	2	2	2	3	2	2	2	2	2
chr21:8527001	chr21	8,527,001	8,528,000	3	3	3	2	2	2	2	2	2	2	2	2	2	2	2	3	2	2	2	2	2
chr21:9847001	chr21	9,847,001	9,848,000	3	3	3	2	2	2	2	1	2	2	2	2	2	2	2	2	2	3	2	2	2
chr21:9848001	chr21	9,848,001	9,849,000	3	3	3	2	2	2	2	1	2	2	2	2	2	2	2	2	2	3	2	2	2
chr21:9854001	chr21	9,854,001	9,855,000	3	3	3	2	2	2	2	1	2	2	2	2	2	2	2	2	2	3	2	2	2
chr21:9855001	chr21	9,855,001	9,856,000	3	3	3	2	2	2	2	1	2	2	2	2	2	2	2	2	2	3	2	2	2
chr21:9856001	chr21	9,856,001	9,857,000	3	3	3	2	2	2	2	1	2	2	2	2	2	2	2	2	2	3	2	2	2
chr21:9881001	chr21	9,881,001	9,882,000	3	3	3	2	2	2	2	2	2	2	2	2	2	2	2	3	2	2	2	2	2
chr21:9985001	chr21	9,985,001	9,986,000	3	3	3	2	2	2	2	2	2	2	2	2	2	2	2	2	3	2	2	2	2
chr21:9986001	chr21	9,986,001	9,987,000	3	3	3	2	2	2	2	2	2	2	2	2	2	2	2	2	3	2	2	2	2
chr21:9987001	chr21	9,987,001	9,988,000	3	3	3	2	2	2	2	2	2	2	2	2	2	2	2	2	3	2	2	2	2
chr21:10656001	chr21	10,656,001	10,657,000	3	3	3	2	2	2	2	2	2	2	2	2	2	2	2	3	2	2	2	2	2
chr21:10657001	chr21	10,657,001	10,658,000	3	3	3	2	2	2	2	2	2	2	2	2	2	2	2	3	2	2	2	2	2
chr21:10745001	chr21	10,745,001	10,746,000	3	3	3	2	2	2	2	2	2	2	2	2	2	2	2	3	2	2	2	2	2
chr21:10746001	chr21	10,746,001	10,747,000	3	3	3	2	2	2	2	2	2	2	2	2	2	2	2	3	2	2	2	2	2
chr21:10747001	chr21	10,747,001	10,748,000	3	3	3	2	2	2	2	2	2	2	2	2	2	2	2	3	2	2	2	2	2

Region	CHR	start	end	HD2	HD3	HD1	BN1	BN2	BN3	BN4	BN5	BN6	LC1	LC2	LC3	LC4	LC5	LC6	LC7	LC8	LC9	LC10	LC10*	LC11
chr21:10748001	chr21	10,748,001	10,749,000	3	3	3	2	2	2	2	2	2	2	2	2	2	2	2	3	2	2	2	2	2
chr21:10749001	chr21	10,749,001	10,750,000	3	3	3	2	2	2	2	2	2	2	2	2	2	2	2	3	2	2	2	2	2
chr21:10751001	chr21	10,751,001	10,752,000	3	3	3	2	2	2	2	2	2	2	2	2	2	2	2	2	2	2	2	2	3
chr21:10752001	chr21	10,752,001	10,753,000	3	3	3	2	2	2	2	2	2	2	2	2	2	2	2	2	2	2	2	2	3
chr21:10753001	chr21	10,753,001	10,754,000	3	3	3	2	2	2	2	2	2	2	2	2	2	2	2	2	2	2	2	2	3
chr21:10754001	chr21	10,754,001	10,755,000	3	3	3	2	2	2	2	2	2	2	2	2	2	2	2	2	2	2	2	2	3
chr21:10755001	chr21	10,755,001	10,756,000	3	3	3	2	2	2	2	2	2	2	2	2	2	2	2	2	2	2	2	2	3
chr21:10756001	chr21	10,756,001	10,757,000	3	3	3	2	2	2	2	2	2	2	2	2	2	2	2	2	2	2	2	2	3
chr21:10757001	chr21	10,757,001	10,758,000	3	3	3	2	2	2	2	2	2	2	2	2	2	2	2	2	2	2	2	2	3
chr21:10758001	chr21	10,758,001	10,759,000	3	3	3	2	2	2	2	2	2	2	2	2	2	2	2	2	2	2	2	2	3
chr21:10759001	chr21	10,759,001	10,760,000	3	3	3	2	2	2	2	2	2	2	2	2	2	2	2	2	2	2	2	2	3
chr21:10760001	chr21	10,760,001	10,761,000	3	3	3	2	2	2	2	2	2	2	2	2	2	2	2	2	2	2	2	2	3
chr22:10524001	chr22	10,524,001	10,525,000	3	3	3	2	2	2	2	1	2	2	2	2	2	2	2	3	2	2	2	2	2
chr22:10525001	chr22	10,525,001	10,526,000	3	3	3	2	2	2	2	1	2	2	2	2	2	2	2	3	2	2	2	2	2
chr22:10526001	chr22	10,526,001	10,527,000	3	3	3	2	2	2	2	1	2	2	2	2	2	2	2	3	2	2	2	2	2
chr22:10527001	chr22	10,527,001	10,528,000	3	3	3	2	2	2	2	1	2	2	2	2	2	2	2	3	2	2	2	2	2
chr22:10528001	chr22	10,528,001	10,529,000	3	3	3	2	2	2	2	1	2	2	2	2	2	2	2	3	2	2	2	2	2
chr22:10529001	chr22	10,529,001	10,530,000	3	3	3	2	2	2	2	1	2	2	2	2	2	2	2	3	2	2	2	2	2
chr22:10530001	chr22	10,530,001	10,531,000	3	3	3	2	2	2	2	2	2	2	2	2	2	2	2	3	2	2	2	2	2
chr22:10531001	chr22	10,531,001	10,532,000	3	3	3	2	2	2	2	2	2	2	2	2	2	2	2	3	2	2	2	2	2
chr22:10532001	chr22	10,532,001	10,533,000	3	3	3	2	2	2	2	2	2	2	2	2	2	2	2	3	2	2	2	2	2
chr22:10533001	chr22	10,533,001	10,534,000	3	3	3	2	2	2	2	2	2	2	2	2	2	2	2	3	2	2	2	2	2
chr22:10534001	chr22	10,534,001	10,535,000	3	3	3	2	2	2	2	2	2	2	2	2	2	2	2	3	2	2	2	2	2
chr22:10535001	chr22	10,535,001	10,536,000	3	3	3	2	2	2	2	2	2	2	2	2	2	2	2	3	2	2	2	2	2
chr22:10536001	chr22	10,536,001	10,537,000	3	3	3	2	2	2	2	2	2	2	2	2	2	2	2	3	2	2	2	2	2
chr22:10537001	chr22	10,537,001	10,538,000	3	3	3	2	2	2	2	2	2	2	2	2	2	2	2	3	2	2	2	2	2
chr22:10538001	chr22	10,538,001	10,539,000	3	3	3	2	2	2	2	2	2	2	2	2	2	2	2	3	2	2	2	2	2
chr22:10539001	chr22	10,539,001	10,540,000	3	3	3	2	2	2	2	2	2	2	2	2	2	2	2	3	2	2	2	2	2
chr22:10546001	chr22	10,546,001	10,547,000	3	3	3	2	2	2	2	2	2	2	2	2	2	2	2	3	2	2	2	2	2
chr22:10547001	chr22	10,547,001	10,548,000	3	3	3	2	2	2	2	2	2	2	2	2	2	2	2	3	2	2	2	2	2
chr22:10548001	chr22	10,548,001	10,549,000	3	3	3	2	2	2	2	2	2	2	2	2	2	2	2	3	2	2	2	2	2
chr22:10549001	chr22	10,549,001	10,550,000	3	3	3	2	2	2	2	2	2	2	2	2	2	2	2	3	2	2	2	2	2
chr22:10550001	chr22	10,550,001	10,551,000	3	3	3	2	2	2	2	2	2	2	2	2	2	2	2	3	2	2	2	2	2
chr22:10551001	chr22	10,551,001	10,552,000	3	3	3	2	2	2	2	2	2	2	2	2	2	2	2	3	2	2	2	2	2
chr22:10552001	chr22	10,552,001	10,553,000	3	3	3	2	2	2	2	2	2	2	2	2	2	2	2	3	2	2	2	2	2
chr22:10553001	chr22	10,553,001	10,554,000	3	3	3	2	2	2	2	2	2	2	2	2	2	2	2	3	2	2	2	2	2
chr22:10561001	chr22	10,561,001	10,562,000	3	3	3	2	2	2	2	2	2	3	2	2	2	2	2	2	2	2	2	2	2
chr22:10568001	chr22	10,568,001	10,569,000	3	3	3	3	2	2	2	2	2	2	2	2	2	2	2	3	2	2	2	2	2
chr22:10569001	chr22	10,569,001	10,570,000	3	3	3	3	2	2	2	2	2	2	2	2	2	2	2	3	2	2	2	2	2
chr22:10570001	chr22	10,570,001	10,571,000	3	3	3	3	2	2	2	2	2	2	2	2	2	2	2	3	2	2	2	2	2
chr22:10571001	chr22	10,571,001	10,572,000	3	3	3	3	2	2	2	2	3	2	2	2	2	2	2	3	2	2	2	2	2
chr22:10572001	chr22	10,572,001	10,573,000	3	3	3	3	2	2	2	2	3	2	2	2	2	2	2	3	2	2	2	2	2
chr22:10573001	chr22	10,573,001	10,574,000	3	3	3	3	2	2	2	2	3	2	2	2	2	2	2	3	2	2	2	2	2
chr22:10574001	chr22	10,574,001	10,575,000	3	3	3	3	2	2	2	2	3	2	2	2	2	2	2	3	2	2	2	2	2
chr22:10611001	chr22	10,611,001	10,612,000	4	3	3	2	2	2	2	2	2	3	2	2	2	3	2	2	2	2	2	2	1
chr22:10623001	chr22	10,623,001	10,624,000	3	3	3	2	2	2	2	2	2	2	2	2	2	3	2	2	2	2	2	2	2
chr22:10624001	chr22	10,624,001	10,625,000	3	3	3	2	2	2	2	2	2	2	2	2	2	3	2	2	2	2	2	2	2
chr22:10625001	chr22	10,625,001	10,626,000	3	3	3	2	2	2	2	2	2	2	2	2	2	3	2	2	2	2	2	2	2
chr22:10629001	chr22	10,629,001	10,630,000	4	3	3	2	2	2	2	2	2	2	2	2	2	3	2	3	2	2	2	2	1
chr22:10630001	chr22	10,630,001	10,631,000	4	3	3	2	2	2	2	2	2	2	2	2	2	3	2	3	2	2	2	2	1
chr22:10631001	chr22	10,631,001	10,632,000	4	3	3	2	2	3	2	2	2	2	2	2	2	3	2	3	2	2	2	2	1
chr22:10632001	chr22	10,632,001	10,633,000	4	3	3	2	2	3	2	2	2	2	2	2	2	3	2	3	2	2	2	2	1
chr22:10684001	chr22	10,684,001	10,685,000	3	3	3	3	2	2	2	1	2	2	2	2	2	2	2	3	2	2	2	2	2
chr22:10685001	chr22	10,685,001	10,686,000	3	3	3	3	2	2	2	2	2	2	2	2	2	2	2	3	2	2	2	2	2
chr22:10686001	chr22	10,686,001	10,687,000	3	3	3	3	2	2	2	2	2	2	2	2	2	2	2	3	2	2	2	2	2
chr22:10687001	chr22	10,687,001	10,688,000	3	3	3	3	2	2	2	2	2	2	2	2	2	2	2	3	2	2	2	2	2
chr22:10688001	chr22	10,688,001	10,689,000	3	3	3	3	2	2	2	2	2	2	2	2	2	2	2	3	2	2	2	2	2
chr1:761001	chr1	761,001	762,000	2	2	2	3	3	3	3	2	3	2	2	2	2	2	2	2	3	2	2	2	2
chr1:762001	chr1	762,001	763,000	2	2	2	3	3	3	3	2	3	2	2	2	2	2	2	2	3	2	2	2	2
chr1:763001	chr1	763,001	764,000	2	2	2	3	3	3	2	2	3	2	2	2	2	2	2	2	3	2	2	2	2
chr1:764001	chr1	764,001	765,000	2	2	2	3	3	3	2	2	3	2	2	2	2	2	2	2	3	2	2	2	2
chr3:198099001	chr3	198,099,001	198,100,000	5	5	2	2	2	2	2	2	2	2	2	2	3	2	2	2	2	2	2	2	2
chr5:69960001	chr5	69,960,001	69,961,000	2	2	2	3	3	2	3	3	3	2	2	2	2	3	2	1	3	2	2	2	2
chr5:70549001	chr5	70,549,001	70,550,000	2	2	2	3	3	3	3	2	3	2	2	3	2	2	2	2	2	2	2	2	2
chr5:70704001	chr5	70,704,001	70,705,000	2	2	2	2	3	3	3	2	3	2	3	2	2	2	2	2	2	2	2	2	2
chr5:70705001	chr5	70,705,001	70,706,000	2	2	2	2	3	3	3	2	3	2	3	2	2	2	2	2	2	2	2	2	2
chr5:70706001	chr5	70,706,001	70,707,000	2	2	2	2	3	3	3	2	3	2	3	2	2	2	2	2	2	2	2	2	2
chr5:70707001	chr5	70,707,001	70,708,000	2	2	2	2	3	3	3	2	3	2	3	2	2	2	2	2	2	2	2	2	2
chr5:70713001	chr5	70,713,001	70,714,000	2	2	2	3	3	3	3	2	3	2	3	2	2	2	2	2	2	2	2	2	2
chr5:70714001	chr5	70,714,001	70,715,000	2	2	2	3	3	3	3	2	3	2	3	2	2	2	2	2	2	2	2	2	2
chr6:140001	chr6	140,001	141,000	2	2	2	2	3	3	3	3	3	2	2	2	2	2	3	2	2	2	2	2	2
chr6:141001	chr6	141,001	142,000	2	2	2	2	3	3	3	3	3	2	2	2	2	2	3	2	2	2	2	2	2
chr6:142001	chr6	142,001	143,000	2	2	2	2	3	3	3	3	3	2	2	2	2	2	3	2	2	2	2	2	2
chr6:143001	chr6	143,001	144,000	2	2	2	2	3	3	3	3	3	2	2	2	2	2	3	2	2	2	2	2	2
chr6:144001	chr6	144,001	145,000	2	2	2	2	3	3	3	3	3	2	2	2	2	2	3	2	2	2	2	2	2
chr6:145001	chr6	145,001	146,000	2	2	2	2	3	3	3	3	3	2	2	2	2	2	3	2	2	2	2	2	2
chr6:146001	chr6	146,001	147,000	2	2	2	2	3	3	3	2	3	2	2	2	2	2	3	2	2	2	2	2	2
chr6:147001	chr6	147,001	148,000	2	2	2	2	3	3	3	2	3	2	2	2	2	2	3	2	2	2	2	2	2
chr6:148001	chr6	148,001	149,000	2	2	2	2	3	3	3	2	3	2	2	2	2	2	3	2	2	2	2	2	2
chr6:398001	chr6	398,001	399,000	2	2	2	2	3	3	3	3	3	2	2	2	2	2	3	2	2	2	2	2	2
chr6:399001	chr6	399,001	400,000	2	2	2	2	3	3	3	3	3	2	2	2	2	2	3	2	2	2	2	2	2
chr6:400001	chr6	400,001	401,000	2	2	2	2	3	3	3	3	3	2	2	2	2	2	3	2	2	2	2	2	2
chr6:401001	chr6	401,001	402,000	2	2	2	2	3	3	3	3	3	2	2	2	2	2	3	2	2	2	2	2	2
chr8:12097001	chr8	12,097,001	12,098,000	1	1	2	1	1	2	1	1	1	2	2	3	2	2	2	2	1	2	2	2	3
chr8:12098001	chr8	12,098,001	12,099,000	1	1	2	1	1	2	1	1	1	2	2	3	2	2	2	2	1	2	2	2	3
chr8:57216001	chr8	57,216,001	57,217,000	2	2	2	2	2	4	4	5	4	2	2	2	2	2	3	2	2	2	2	2	2
chr15:101132001	chr15	101,132,001	101,133,000	2	2	2	2	3	3	3	3	3	2	2	2	2	2	3	2	2	2	2	2	2
chr15:101133001	chr15	101,133,001	101,134,000	2	2	2	2	3	3	3	3	3	2	2	2	2	2	3	2	2	2	2	2	2
chr15:101134001	chr15	101,134,001	101,135,000	2	2	2	2	3	3	3	3	3	2	2	2	2	2	3	2	2	2	2	2	2
chr15:101135001	chr15	101,135,001	101,136,000	2	2	2	2	3	3	3	3	3	2	2	2	2	2	3	2	2	2	2	2	2
chr15:101136001	chr15	101,136,001	101,137,000	2	2	2	2	3	3	3	3	3	2	2	2	2	2	3	2	2	2	2	2	2
chr15:101137001	chr15	101,137,001	101,138,000	2	2	2	2	3	3	3	3	3	2	2	2	2	2	3	2	2	2	2	2	2
chr15:101138001	chr15	101,138,001	101,139,000	2	2	2	2	3	3	3	3	3	2	2	2	2	2	3	2	2	2	2	2	2
chr15:101158001	chr15	101,158,001	101,159,000	2	2	2	2	3	3	3	2	3	2	2	2	2	2	3	2	2	2	2	2	2
chr15:101161001	chr15	101,161,001	101,162,000	2	2	2	2	3	3	3	2	3	2	2	2	2	2	3	2	2	2	2	2	2
chr15:101162001	chr15	101,162,001	101,163,000	2	2	2	2	3	3	3	2	3	2	2	2	2	2	3	2	2	2	2	2	2
chr15:101163001	chr15	101,163,001	101,164,000	2	2	2	2	3	3	3	2	3	2	2	2	2	2	3	2	2	2	2	2	2
chr15:101164001	chr15	101,164,001	101,165,000	2	2	2	2	3	3	3	2	3	2	2	2	2	2	3	2	2	2	2	2	2
chr15:101165001	chr15	101,165,001	101,166,000	2	2	2	2	3	3	3	2	3	2	2	2	2	2	3	2	2	2	2	2	2
chr15:101166001	chr15	101,166,001	101,167,000	2	2	2	2	3	3	3	2	3	2	2	2	2	2	3	2	2	2	2	2	2
chr15:101167001	chr15	101,167,001	101,168,000	2	2	2	2	3	3	3	2	3	2	2	2	2	2	3	2	2	2	2	2	2
chr15:101168001	chr15	101,168,001	101,169,000	2	2	2	2	3	3	3	2	3	2	2	2	2	2	3	2	2	2	2	2	2
chr15:101169001	chr15	101,169,001	101,170,000	2	2	2	2	3	3	3	2	3	2	2	2	2	2	3	2	2	2	2	2	2
chr15:101170001	chr15	101,170,001	101,171,000	2	2	2	2	3	3	3	2	3	2	2	2	2	2	3	2	2	2	2	2	2
chr15:101171001	chr15	101,171,001	101,172,000	2	2	2	2	3	3	3	2	3	2	2	2	2	2	3	2	2	2	2	2	2
chr15:101172001	chr15	101,172,001	101,173,000	2	2	2	2	3	3	3	2	3	2	2	2	2	2	3	2	2	2	2	2	2
chr15:101173001	chr15	101,173,001	101,174,000	2	2	2	2	3	3	3	2	3	2	2	2	2	2	3	2	2	2	2	2	2
chr15:101174001	chr15	101,174,001	101,175,000	2	2	2	2	3	3	3	2	3	2	2	2	2	2	3	2	2	2	2	2	2
chr15:101175001	chr15	101,175,001	101,176,000	2	2	2	2	3	3	3	2	3	2	2	2	2	2	3	2	2	2	2	2	2
chr15:101176001	chr15	101,176,001	101,177,000	2	2	2	2	3	3	3	2	3	2	2	2	2	2	3	2	2	2	2	2	2
chr15:101177001	chr15	101,177,001	101,178,000	2	2	2	2	3	3	3	2	3	2	2	2	2	2	3	2	2	2	2	2	2
chr15:101178001	chr15	101,178,001	101,179,000	2	2	2	2	3	3	3	2	3	2	2	2	2	2	3	2	2	2	2	2	2
chr15:101179001	chr15	101,179,001	101,180,000	2	2	2	2	3	3	3	2	3	2	2	2	2	2	3	2	2	2	2	2	2

Region	CHR	start	end	HD2	HD3	HD1	BN1	BN2	BN3	BN4	BN5	BN6	LC1	LC2	LC3	LC4	LC5	LC6	LC7	LC8	LC9	LC10	LC10*	LC11
chr15:101180001	chr15	101,180,001	101,181,000	2	2	2	2	3	3	3	2	3	2	2	2	2	2	3	2	2	2	2	2	2
chr15:101181001	chr15	101,181,001	101,182,000	2	2	2	2	3	3	3	2	3	2	2	2	2	2	3	2	2	2	2	2	2
chr15:101182001	chr15	101,182,001	101,183,000	2	2	2	2	3	3	3	2	3	2	2	2	2	2	3	2	2	2	2	2	2
chr15:101183001	chr15	101,183,001	101,184,000	2	2	2	2	3	3	3	2	3	2	2	2	2	2	3	2	2	2	2	2	2
chr15:101339001	chr15	101,339,001	101,340,000	2	2	2	2	3	3	3	2	3	2	2	2	2	2	3	2	2	2	2	2	2
chr15:101340001	chr15	101,340,001	101,341,000	2	2	2	2	3	3	3	2	3	2	2	2	2	2	3	2	2	2	2	2	2
chr15:101341001	chr15	101,341,001	101,342,000	2	2	2	2	3	3	3	3	3	2	2	2	2	2	3	2	2	2	2	2	2
chr15:101342001	chr15	101,342,001	101,343,000	2	2	2	2	3	3	3	3	3	2	2	2	2	2	3	2	2	2	2	2	2
chr15:101343001	chr15	101,343,001	101,344,000	2	2	2	2	3	3	3	3	3	2	2	2	2	2	3	2	2	2	2	2	2
chr15:101344001	chr15	101,344,001	101,345,000	2	2	2	2	3	3	3	3	3	2	2	2	2	2	3	2	2	2	2	2	2
chr15:101353001	chr15	101,353,001	101,354,000	2	2	2	2	3	3	3	3	3	2	2	2	2	2	3	2	2	2	2	2	2
chr15:101354001	chr15	101,354,001	101,355,000	2	2	2	2	3	3	3	3	3	2	2	2	2	2	3	2	2	2	2	2	2
chr15:101355001	chr15	101,355,001	101,356,000	2	2	2	2	3	3	3	3	3	2	2	2	2	2	3	2	2	2	2	2	2
chr15:101356001	chr15	101,356,001	101,357,000	2	2	2	2	3	3	3	3	2	2	2	2	2	2	3	2	2	2	2	2	2
chr15:101357001	chr15	101,357,001	101,358,000	2	2	2	2	3	3	3	3	2	2	2	2	2	2	3	2	2	2	2	2	2
chr15:101358001	chr15	101,358,001	101,359,000	2	2	2	2	3	3	3	3	2	2	2	2	2	2	3	2	2	2	2	2	2
chr15:101359001	chr15	101,359,001	101,360,000	2	2	2	2	3	3	3	3	2	2	2	2	2	2	3	2	2	2	2	2	2
chr15:101360001	chr15	101,360,001	101,361,000	2	2	2	2	3	3	3	3	2	2	2	2	2	2	3	2	2	2	2	2	2
chr15:101365001	chr15	101,365,001	101,366,000	2	2	2	2	3	3	3	2	3	2	2	2	2	2	3	2	2	2	2	2	2
chr15:101389001	chr15	101,389,001	101,390,000	2	2	2	2	2	3	3	3	3	2	2	2	2	2	3	2	2	2	2	2	2
chr15:101390001	chr15	101,390,001	101,391,000	2	2	2	2	2	3	3	3	3	2	2	2	2	2	3	2	2	2	2	2	2
chr15:101391001	chr15	101,391,001	101,392,000	2	2	2	2	2	3	3	3	3	2	2	2	2	2	3	2	2	2	2	2	2
chr15:101392001	chr15	101,392,001	101,393,000	2	2	2	2	2	3	3	3	3	2	2	2	2	2	3	2	2	2	2	2	2
chr15:101393001	chr15	101,393,001	101,394,000	2	2	2	2	3	3	3	3	3	2	2	2	2	2	3	2	2	2	2	2	2
chr15:101394001	chr15	101,394,001	101,395,000	2	2	2	2	3	3	3	3	3	2	2	2	2	2	3	2	2	2	2	2	2
chr15:101395001	chr15	101,395,001	101,396,000	2	2	2	2	3	3	3	3	3	2	2	2	2	2	3	2	2	2	2	2	2
chr15:101396001	chr15	101,396,001	101,397,000	2	2	2	2	3	3	3	3	3	2	2	2	2	2	3	2	2	2	2	2	2
chr15:101397001	chr15	101,397,001	101,398,000	2	2	2	2	3	3	3	3	3	2	2	2	2	2	3	2	2	2	2	2	2
chr15:101398001	chr15	101,398,001	101,399,000	2	2	2	2	3	3	3	3	3	2	2	2	2	2	3	2	2	2	2	2	2
chr15:101399001	chr15	101,399,001	101,400,000	2	2	2	2	3	3	3	3	3	2	2	2	2	2	3	2	2	2	2	2	2
chr15:101400001	chr15	101,400,001	101,401,000	2	2	2	2	3	3	3	3	3	2	2	2	2	2	3	2	2	2	2	2	2
chr15:101401001	chr15	101,401,001	101,402,000	2	2	2	2	3	3	3	3	3	2	2	2	2	2	3	2	2	2	2	2	2
chr15:101402001	chr15	101,402,001	101,403,000	2	2	2	2	3	3	3	3	3	2	2	2	2	2	3	2	2	2	2	2	2
chr15:101403001	chr15	101,403,001	101,404,000	2	2	2	2	3	3	3	3	3	2	2	2	2	2	3	2	2	2	2	2	2
chr15:101404001	chr15	101,404,001	101,405,000	2	2	2	2	3	3	3	3	3	2	2	2	2	2	3	2	2	2	2	2	2
chr15:101405001	chr15	101,405,001	101,406,000	2	2	2	2	3	3	3	3	3	2	2	2	2	2	3	2	2	2	2	2	2
chr15:101406001	chr15	101,406,001	101,407,000	2	2	2	2	3	3	3	3	3	2	2	2	2	2	3	2	2	2	2	2	2
chr15:101407001	chr15	101,407,001	101,408,000	2	2	2	2	3	3	3	3	3	2	2	2	2	2	3	2	2	2	2	2	2
chr15:101440001	chr15	101,440,001	101,441,000	2	2	2	2	3	3	3	3	3	2	2	2	2	2	3	2	2	2	2	2	2
chr15:101441001	chr15	101,441,001	101,442,000	1	2	2	2	3	3	3	3	3	2	2	2	2	2	3	2	2	2	2	2	2
chr15:101442001	chr15	101,442,001	101,443,000	1	2	2	2	3	3	3	3	3	2	2	2	2	2	3	2	2	2	2	2	2
chr15:101443001	chr15	101,443,001	101,444,000	1	2	2	2	3	3	3	2	3	2	2	2	2	2	3	2	2	2	2	2	2
chr15:101444001	chr15	101,444,001	101,445,000	1	2	2	2	3	3	3	2	3	2	2	2	2	2	3	2	2	2	2	2	2
chr15:101476001	chr15	101,476,001	101,477,000	2	2	2	2	3	3	3	3	3	2	2	2	2	2	3	2	2	2	2	2	2
chr15:101477001	chr15	101,477,001	101,478,000	2	2	2	2	3	3	3	3	3	2	2	2	2	2	3	2	2	2	2	2	2
chr15:101478001	chr15	101,478,001	101,479,000	2	2	2	2	3	3	3	3	3	2	2	2	2	2	3	2	2	2	2	2	2
chr15:101493001	chr15	101,493,001	101,494,000	2	2	2	2	3	3	3	2	3	2	2	2	2	2	3	2	2	2	2	2	2
chr15:101494001	chr15	101,494,001	101,495,000	2	2	2	2	3	3	3	2	3	2	2	2	2	2	3	2	2	2	2	2	2
chr15:101495001	chr15	101,495,001	101,496,000	2	2	2	2	3	3	3	2	3	2	2	2	2	2	3	2	2	2	2	2	2
chr15:101496001	chr15	101,496,001	101,497,000	2	2	2	2	3	3	3	2	3	2	2	2	2	2	3	2	2	2	2	2	2
chr15:101497001	chr15	101,497,001	101,498,000	2	2	2	2	3	3	3	2	3	2	2	2	2	2	3	2	2	2	2	2	2
chr15:101498001	chr15	101,498,001	101,499,000	2	2	2	2	3	3	3	2	3	2	2	2	2	2	3	2	2	2	2	2	2
chr15:101499001	chr15	101,499,001	101,500,000	2	2	2	2	3	3	3	2	3	2	2	2	2	2	3	2	2	2	2	2	2
chr15:101500001	chr15	101,500,001	101,501,000	2	2	2	2	3	3	3	2	3	2	2	2	2	2	3	2	2	2	2	2	2
chr15:101501001	chr15	101,501,001	101,502,000	2	2	2	2	3	3	3	2	3	2	2	2	2	2	3	2	2	2	2	2	2
chr15:101502001	chr15	101,502,001	101,503,000	2	2	2	2	3	3	3	2	3	2	2	2	2	2	3	2	2	2	2	2	2
chr15:101503001	chr15	101,503,001	101,504,000	2	2	2	2	3	3	3	2	3	2	2	2	2	2	3	2	2	2	2	2	2
chr15:101504001	chr15	101,504,001	101,505,000	2	2	2	2	3	3	3	2	3	2	2	2	2	2	3	2	2	2	2	2	2
chr15:101505001	chr15	101,505,001	101,506,000	2	2	2	2	3	3	3	2	3	2	2	2	2	2	3	2	2	2	2	2	2
chr15:101506001	chr15	101,506,001	101,507,000	2	2	2	2	3	3	3	2	3	2	2	2	2	2	3	2	2	2	2	2	2
chr15:101507001	chr15	101,507,001	101,508,000	2	2	2	2	3	3	3	2	3	2	2	2	2	2	3	2	2	2	2	2	2
chr15:101537001	chr15	101,537,001	101,538,000	2	2	2	2	3	3	3	2	3	2	2	2	2	2	3	2	2	2	2	2	2
chr15:101538001	chr15	101,538,001	101,539,000	2	2	2	2	3	3	3	2	3	2	2	2	2	2	3	2	2	2	2	2	2
chr15:101539001	chr15	101,539,001	101,540,000	2	2	2	2	3	3	3	2	3	2	2	2	2	2	3	2	2	2	2	2	2
chr15:101540001	chr15	101,540,001	101,541,000	2	2	2	2	3	3	3	2	3	2	2	2	2	2	3	2	2	2	2	2	2
chr15:101558001	chr15	101,558,001	101,559,000	2	2	2	2	3	3	3	3	3	2	2	2	2	2	3	2	2	2	2	2	2
chr15:101559001	chr15	101,559,001	101,560,000	2	2	2	2	3	3	3	3	3	2	2	2	2	2	3	2	2	2	2	2	2
chr15:101560001	chr15	101,560,001	101,561,000	2	2	2	2	3	3	3	3	3	2	2	2	2	2	3	2	2	2	2	2	2
chr15:101561001	chr15	101,561,001	101,562,000	2	2	2	2	3	3	3	3	3	2	2	2	2	2	3	2	2	2	2	2	2
chr15:101562001	chr15	101,562,001	101,563,000	2	2	2	2	3	3	3	3	3	2	2	2	2	2	3	2	2	2	2	2	2
chr15:101563001	chr15	101,563,001	101,564,000	2	2	2	2	3	3	3	3	3	2	2	2	2	2	3	2	2	2	2	2	2
chr15:101564001	chr15	101,564,001	101,565,000	2	2	2	2	3	3	3	3	3	2	2	2	2	2	3	2	2	2	2	2	2
chr15:101565001	chr15	101,565,001	101,566,000	2	2	2	2	3	3	3	3	3	2	2	2	2	2	3	2	2	2	2	2	2
chr15:101652001	chr15	101,652,001	101,653,000	2	2	2	2	3	3	3	3	3	2	2	2	2	2	3	2	2	2	2	2	2
chr15:101653001	chr15	101,653,001	101,654,000	2	2	2	2	3	3	3	3	3	2	2	2	2	2	3	2	2	2	2	2	2
chr15:101654001	chr15	101,654,001	101,655,000	2	2	2	2	3	3	3	3	3	2	2	2	2	2	3	2	2	2	2	2	2
chr15:101655001	chr15	101,655,001	101,656,000	2	2	2	2	3	3	3	3	3	2	2	2	2	2	3	2	2	2	2	2	2
chr15:101656001	chr15	101,656,001	101,657,000	2	2	2	2	3	3	3	3	3	2	2	2	2	2	3	2	2	2	2	2	2
chr16:151001	chr16	151,001	152,000	2	2	2	2	3	3	3	3	2	2	2	2	2	2	3	2	2	2	2	2	2
chr16:207001	chr16	207,001	208,000	2	2	2	2	3	3	3	3	2	2	2	2	2	2	2	2	2	2	2	3	2
chr16:208001	chr16	208,001	209,000	2	2	2	2	3	3	3	3	2	2	2	2	2	2	2	2	2	2	2	3	2
chr16:209001	chr16	209,001	210,000	2	2	2	2	3	3	3	3	3	2	2	2	2	2	2	2	2	2	2	3	2
chr17:120001	chr17	120,001	121,000	2	2	2	4	3	3	2	3	4	2	2	2	2	2	3	2	2	2	2	2	2
chr21:8522001	chr21	8,522,001	8,523,000	5	3	2	2	2	2	2	2	2	2	2	2	2	2	2	3	2	2	2	2	2
chr21:44119001	chr21	44,119,001	44,120,000	2	2	2	3	3	3	4	3	3	2	2	2	2	3	3	2	2	2	2	2	1
chr21:44120001	chr21	44,120,001	44,121,000	2	2	2	3	3	3	3	3	3	2	2	2	2	3	3	2	2	2	2	2	1
chr21:44121001	chr21	44,121,001	44,122,000	2	2	2	3	3	3	3	3	3	2	2	2	2	3	3	2	2	2	2	2	1
chr6:170633001	chr6	170,633,001	170,634,000	1	1	1	2	2	2	3	2	2	2	1	2	3	2	3	2	2	2	2	2	2
chr6:170634001	chr6	170,634,001	170,635,000	1	1	1	2	2	2	3	2	2	2	1	2	3	2	3	2	2	2	2	2	2
chr8:150001	chr8	150,001	151,000	1	2	1	2	2	2	2	2	2	2	2	2	2	2	3	2	2	2	2	2	2
chr10:47704001	chr10	47,704,001	47,705,000	2	2	1	2	3	3	2	3	3	2	2	2	2	2	2	3	2	2	2	2	2
chr10:47705001	chr10	47,705,001	47,706,000	2	2	1	2	3	3	2	3	3	2	2	2	2	2	2	3	2	2	2	2	2
chr15:101829001	chr15	101,829,001	101,830,000	2	2	1	2	3	3	3	3	3	2	2	2	2	2	3	2	2	2	2	2	2
chr15:101830001	chr15	101,830,001	101,831,000	2	2	1	2	3	3	3	3	3	2	2	2	2	2	3	2	2	2	2	2	2
chr17:41275001	chr17	41,275,001	41,276,000	1	2	1	2	2	2	2	2	2	2	2	2	2	2	3	2	2	2	2	2	2
chr17:76369001	chr17	76,369,001	76,370,000	1	2	1	2	2	2	2	2	2	2	2	2	2	3	2	2	2	2	2	2	2
chr21:44146001	chr21	44,146,001	44,147,000	2	2	1	3	3	3	4	4	3	2	3	2	1	3	3	2	2	2	2	1	2
chr21:44147001	chr21	44,147,001	44,148,000	2	2	1	3	3	3	4	4	3	2	3	2	1	3	3	2	2	2	2	1	2
chr5:562001	chr5	562,001	563,000	1	1	0	2	1	2	2	2	2	2	2	2	2	2	4	2	1	2	2	2	2
chr21:44157001	chr21	44,157,001	44,158,000	2	2	0	5	3	2	4	4	3	2	2	2	2	3	2	2	2	2	2	2	2
chr1:64416001	chr1	64,416,001	64,417,000	2	4	5	2	2	2	2	2	2	2	2	2	2	2	2	2	2	2	2	2	2
chr2:151010001	chr2	151,010,001	151,011,000	2	4	5	2	2	2	2	2	2	2	2	2	2	2	2	2	2	2	2	2	2
chr3:14180001	chr3	14,180,001	14,181,000	2	4	5	2	2	2	2	2	2	2	2	2	2	2	2	2	2	2	2	2	2
chr6:6068001	chr6	6,068,001	6,069,000	2	5	5	2	2	2	2	2	2	2	2	2	2	2	2	2	2	2	2	2	2
chr6:29720001	chr6	29,720,001	29,721,000	2	5	5	2	2	2	2	2	2	2	2	2	2	2	2	2	2	2	2	2	2
chr7:142383001	chr7	142,383,001	142,384,000	2	5	5	2	2	2	2	2	2	2	2	3	1	2	2	2	2	2	2	2	2
chr8:3660001	chr8	3,660,001	3,661,000	2	4	5	2	2	2	2	2	2	2	2	2	2	2	2	2	2	2	2	2	2
chr11:1510001	chr11	1,510,001	1,511,000	2	4	5	2	2	2	2	2	2	2	2	2	2	2	2	2	2	2	2	2	2
chr12:48712001	chr12	48,712,001	48,713,000	2	4	5	2	2	2	2	2	2	2	2	2	2	2	2	2	2	2	2	2	2
chr13:39796001	chr13	39,796,001	39,797,000	3	5	5	2	2	2	2	2	2	2	2	2	2	2	2	2	2	2	2	2	2
chr21:5252001	chr21	5,252,001	5,253,000	5	2	5	4	2	2	2	2	2	2	2	2	2	2	2	2	2	2	2	2	2
chr21:5253001	chr21	5,253,001	5,254,000	5	2	5	4	2	2	2	2	2	2	2	2	2	2	2	2	2	2	2	2	2
chr21:5254001	chr21	5,254,001	5,255,000	5	2	5	2	2	2	2	2	2	2	2	2	2	2	2	2	2	2	2	2	2

Region	CHR	start	end	HD2	HD3	HD1	BN1	BN2	BN3	BN4	BN5	BN6	LC1	LC2	LC3	LC4	LC5	LC6	LC7	LC8	LC9	LC10	LC10*	LC11
chr21:9837001	chr21	9,837,001	9,838,000	3	5	5	2	2	2	2	2	3	2	1	2	2	1	2	2	2	2	2	2	4
chr2:132287001	chr2	132,287,001	132,288,000	2	4	4	2	2	2	2	2	2	2	2	2	2	2	2	2	2	2	2	2	2
chr3:93783001	chr3	93,783,001	93,784,000	3	3	4	2	2	2	2	2	2	2	2	2	2	2	2	2	2	2	2	2	2
chr3:93784001	chr3	93,784,001	93,785,000	3	3	4	2	2	2	2	2	2	2	2	2	2	2	2	2	2	2	2	2	2
chr4:33841001	chr4	33,841,001	33,842,000	3	3	4	2	2	2	2	2	2	2	2	2	2	2	2	2	2	2	2	2	2
chr5:131516001	chr5	131,516,001	131,517,000	3	2	4	2	2	2	2	2	2	2	2	2	2	2	2	2	2	2	2	2	2
chr6:56893001	chr6	56,893,001	56,894,000	2	5	4	2	2	2	2	2	2	2	2	2	2	2	2	2	2	2	2	2	2
chr9:41351001	chr9	41,351,001	41,352,000	2	3	4	2	2	2	1	1	2	2	3	2	2	2	1	2	2	2	2	2	2
chr9:41352001	chr9	41,352,001	41,353,000	2	3	4	2	2	2	1	1	2	2	3	2	2	2	1	2	2	2	2	2	2
chr9:41353001	chr9	41,353,001	41,354,000	2	3	4	2	2	2	1	1	2	2	3	2	2	2	1	2	2	2	2	2	2
chr9:41354001	chr9	41,354,001	41,355,000	2	3	4	2	2	2	1	1	2	2	3	2	2	2	1	2	2	2	2	2	2
chr9:41355001	chr9	41,355,001	41,356,000	2	3	4	2	2	2	1	1	2	2	3	2	2	2	1	2	2	2	2	2	2
chr11:48853001	chr11	48,853,001	48,854,000	3	2	4	2	3	2	2	2	2	2	2	2	2	2	2	2	2	2	2	2	2
chr12:37265001	chr12	37,265,001	37,266,000	5	2	4	2	2	2	2	2	2	2	2	2	2	2	2	2	2	2	2	2	2
chr13:18491001	chr13	18,491,001	18,492,000	3	4	4	2	2	3	2	2	3	2	2	2	2	1	2	2	2	2	2	2	3
chr13:18492001	chr13	18,492,001	18,493,000	3	4	4	2	2	3	2	2	2	2	2	2	2	1	2	2	2	2	2	2	3
chr13:18493001	chr13	18,493,001	18,494,000	3	4	4	2	2	2	2	2	2	2	2	2	2	1	2	2	2	2	2	2	3
chr13:18494001	chr13	18,494,001	18,495,000	3	4	4	2	2	2	2	2	2	2	2	2	2	1	2	2	2	2	2	2	3
chr5:1073001	chr5	1,073,001	1,074,000	2	2	3	1	1	2	2	1	2	2	2	2	2	2	2	2	2	2	2	2	2
chr7:57617001	chr7	57,617,001	57,618,000	3	2	3	2	2	2	2	2	2	2	2	2	2	2	2	2	2	2	2	2	2
chr7:57702001	chr7	57,702,001	57,703,000	3	2	3	2	2	2	2	2	2	2	2	2	2	2	2	2	2	2	2	2	2
chr7:57703001	chr7	57,703,001	57,704,000	3	2	3	2	2	2	2	2	2	2	2	2	2	2	2	2	2	2	2	2	2
chr9:39740001	chr9	39,740,001	39,741,000	3	2	3	2	2	2	1	2	2	2	2	2	2	2	2	2	2	2	2	2	2
chr9:40013001	chr9	40,013,001	40,014,000	3	3	3	3	2	3	2	2	2	2	2	2	2	3	1	2	2	2	2	2	2
chr9:40025001	chr9	40,025,001	40,026,000	3	3	3	3	2	3	2	2	2	2	2	2	2	2	2	2	2	2	2	2	2
chr9:40026001	chr9	40,026,001	40,027,000	3	3	3	3	3	3	2	2	2	2	2	2	2	2	2	2	2	2	2	2	2
chr9:40027001	chr9	40,027,001	40,028,000	3	3	3	3	3	3	2	3	2	2	2	2	2	2	2	2	2	2	2	2	2
chr9:40028001	chr9	40,028,001	40,029,000	3	3	3	3	3	3	2	3	2	2	2	2	2	2	2	2	2	2	2	2	2
chr9:40029001	chr9	40,029,001	40,030,000	3	3	3	3	3	3	2	3	2	2	2	2	2	2	2	2	2	2	2	2	2
chr9:40030001	chr9	40,030,001	40,031,000	3	3	3	3	3	3	2	3	2	2	2	2	2	2	2	2	2	2	2	2	2
chr9:40031001	chr9	40,031,001	40,032,000	3	3	3	3	3	3	2	3	2	2	2	2	2	2	2	2	2	2	2	2	2
chr9:40048001	chr9	40,048,001	40,049,000	3	2	3	3	3	3	2	2	2	2	2	2	2	2	2	2	2	2	2	2	2
chr9:40049001	chr9	40,049,001	40,050,000	3	2	3	3	3	3	2	2	2	2	2	2	2	2	2	2	2	2	2	2	2
chr9:40050001	chr9	40,050,001	40,051,000	3	2	3	3	3	3	2	2	2	2	2	2	2	2	2	2	2	2	2	2	2
chr9:40051001	chr9	40,051,001	40,052,000	3	2	3	3	3	3	2	2	2	2	2	2	2	2	2	2	2	2	2	2	2
chr9:40052001	chr9	40,052,001	40,053,000	3	2	3	3	3	3	2	2	2	2	2	2	2	2	2	2	2	2	2	2	2
chr9:40053001	chr9	40,053,001	40,054,000	3	2	3	3	3	3	2	2	2	2	2	2	2	2	2	2	2	2	2	2	2
chr9:40054001	chr9	40,054,001	40,055,000	3	2	3	3	3	3	2	2	2	2	2	2	2	2	2	2	2	2	2	2	2
chr9:40055001	chr9	40,055,001	40,056,000	3	2	3	3	3	3	2	2	2	2	2	2	2	2	2	2	2	2	2	2	2
chr9:40056001	chr9	40,056,001	40,057,000	3	2	3	3	3	3	2	2	2	2	2	2	2	2	2	2	2	2	2	2	2
chr9:40057001	chr9	40,057,001	40,058,000	3	2	3	3	3	3	2	2	2	2	2	2	2	2	2	2	2	2	2	2	2
chr9:40058001	chr9	40,058,001	40,059,000	3	2	3	3	3	3	2	2	2	2	2	2	2	2	2	2	2	2	2	2	2
chr9:40059001	chr9	40,059,001	40,060,000	3	2	3	3	3	3	2	2	2	2	2	2	2	2	2	2	2	2	2	2	2
chr9:40060001	chr9	40,060,001	40,061,000	3	2	3	3	3	3	2	2	2	2	2	2	2	2	2	2	2	2	2	2	2
chr9:40079001	chr9	40,079,001	40,080,000	3	3	3	3	2	3	2	2	2	2	2	2	2	3	1	2	2	2	2	2	2
chr9:40080001	chr9	40,080,001	40,081,000	3	3	3	3	2	3	2	2	2	2	2	2	2	3	1	2	2	2	2	2	2
chr9:40081001	chr9	40,081,001	40,082,000	3	3	3	3	2	3	2	2	2	2	2	2	2	3	1	2	2	2	2	2	2
chr9:40082001	chr9	40,082,001	40,083,000	3	3	3	3	2	3	2	2	2	2	2	2	2	3	1	2	2	2	2	2	2
chr9:40083001	chr9	40,083,001	40,084,000	3	3	3	3	2	3	2	2	2	2	2	2	2	3	1	2	2	2	2	2	2
chr9:40084001	chr9	40,084,001	40,085,000	3	3	3	3	2	3	2	2	2	2	2	2	2	3	1	2	2	2	2	2	2
chr9:40085001	chr9	40,085,001	40,086,000	3	3	3	3	3	3	2	2	2	2	2	2	2	3	1	2	2	2	2	2	2
chr9:40086001	chr9	40,086,001	40,087,000	3	3	3	3	3	3	2	2	2	2	2	2	2	3	1	2	2	2	2	2	2
chr9:40088001	chr9	40,088,001	40,089,000	3	3	3	3	3	3	2	2	2	2	3	2	2	2	1	2	2	2	2	2	2
chr9:40089001	chr9	40,089,001	40,090,000	3	3	3	3	3	3	2	2	2	2	3	2	2	2	1	2	2	2	2	2	2
chr9:40090001	chr9	40,090,001	40,091,000	3	3	3	3	3	3	2	2	2	2	3	2	2	2	1	2	2	2	2	2	2
chr9:40091001	chr9	40,091,001	40,092,000	3	3	3	3	3	3	2	2	2	2	3	2	2	2	1	2	2	2	2	2	2
chr9:40092001	chr9	40,092,001	40,093,000	3	3	3	3	3	3	2	2	2	2	3	2	2	2	1	2	2	2	2	2	2
chr9:40093001	chr9	40,093,001	40,094,000	3	3	3	3	3	3	2	2	2	2	3	2	2	2	1	2	2	2	2	2	2
chr9:40094001	chr9	40,094,001	40,095,000	3	3	3	3	3	3	2	2	2	2	3	2	2	2	1	2	2	2	2	2	2
chr9:40127001	chr9	40,127,001	40,128,000	2	2	3	4	3	3	2	3	2	2	2	2	2	2	2	2	2	2	2	2	2
chr9:41340001	chr9	41,340,001	41,341,000	4	3	3	2	2	2	1	1	2	2	3	2	2	2	1	2	2	2	2	2	2
chr9:41341001	chr9	41,341,001	41,342,000	4	3	3	2	2	2	1	1	2	2	3	2	2	2	1	2	2	2	2	2	2
chr9:41730001	chr9	41,730,001	41,731,000	2	3	3	2	2	2	1	2	2	2	2	2	2	2	2	2	2	2	2	2	2
chr9:41731001	chr9	41,731,001	41,732,000	2	3	3	2	2	2	1	1	2	2	2	2	2	2	2	2	2	2	2	2	2
chr9:42820001	chr9	42,820,001	42,821,000	3	2	3	2	2	2	2	2	2	2	2	2	2	2	2	2	2	2	2	2	2
chr9:42821001	chr9	42,821,001	42,822,000	3	2	3	2	2	2	2	2	2	2	2	2	2	2	2	2	2	2	2	2	2
chr9:42825001	chr9	42,825,001	42,826,000	3	2	3	2	2	2	2	2	2	2	2	2	2	2	2	2	2	2	2	2	2
chr9:42826001	chr9	42,826,001	42,827,000	3	2	3	2	2	2	2	2	2	2	2	2	2	2	2	2	2	2	2	2	2
chr9:42827001	chr9	42,827,001	42,828,000	3	2	3	2	2	2	2	2	2	2	2	2	2	2	2	2	2	2	2	2	2
chr9:42828001	chr9	42,828,001	42,829,000	3	2	3	2	2	2	2	2	2	2	2	2	2	2	2	2	2	2	2	2	2
chr9:42829001	chr9	42,829,001	42,830,000	3	2	3	2	2	2	2	2	2	2	2	2	2	2	2	2	2	2	2	2	2
chr9:42830001	chr9	42,830,001	42,831,000	3	2	3	2	2	2	2	2	2	2	2	2	2	2	2	2	2	2	2	2	2
chr9:42831001	chr9	42,831,001	42,832,000	3	2	3	2	2	2	2	2	2	2	2	2	2	2	2	2	2	2	2	2	2
chr9:42897001	chr9	42,897,001	42,898,000	3	2	3	2	2	2	2	2	2	2	2	2	2	2	2	2	2	2	2	2	2
chr9:42898001	chr9	42,898,001	42,899,000	3	2	3	2	2	2	2	2	2	2	2	2	2	2	2	2	2	2	2	2	2
chr9:42899001	chr9	42,899,001	42,900,000	3	5	3	2	2	2	2	2	2	2	2	2	2	2	2	2	2	2	2	2	2
chr9:42901001	chr9	42,901,001	42,902,000	5	2	3	2	2	2	2	2	2	2	2	2	2	2	2	2	2	2	2	2	2
chr9:43302001	chr9	43,302,001	43,303,000	3	3	3	2	2	2	2	2	2	2	2	2	1	2	2	2	2	2	3	2	2
chr9:43303001	chr9	43,303,001	43,304,000	3	3	3	2	2	2	2	2	2	2	2	2	1	2	2	2	2	2	3	2	2
chr9:43304001	chr9	43,304,001	43,305,000	3	3	3	2	2	2	2	2	2	2	2	2	1	2	2	2	2	2	3	2	2
chr9:43355001	chr9	43,355,001	43,356,000	3	3	3	2	2	2	2	2	2	2	3	2	1	2	2	2	2	2	2	2	2
chr9:43356001	chr9	43,356,001	43,357,000	3	3	3	2	2	2	2	2	2	2	3	2	1	2	2	2	2	2	2	2	2
chr9:61889001	chr9	61,889,001	61,890,000	3	3	3	2	2	2	1	2	2	2	2	2	2	2	2	2	2	2	2	2	2
chr9:61890001	chr9	61,890,001	61,891,000	3	3	3	2	2	2	1	2	2	2	2	2	2	2	2	2	2	2	2	2	2
chr9:64331001	chr9	64,331,001	64,332,000	2	3	3	2	2	2	1	1	2	2	2	2	2	2	2	2	2	2	2	2	2
chr9:64332001	chr9	64,332,001	64,333,000	2	3	3	2	2	2	1	1	2	2	2	2	2	2	2	2	2	2	2	2	2
chr9:64822001	chr9	64,822,001	64,823,000	3	3	3	2	3	3	2	2	2	2	3	2	2	2	1	2	2	2	2	2	2
chr9:65275001	chr9	65,275,001	65,276,000	2	3	3	2	2	3	2	2	2	2	2	2	2	2	2	2	2	2	2	2	2
chr9:65276001	chr9	65,276,001	65,277,000	2	3	3	2	2	3	2	2	2	2	2	2	2	2	2	2	2	2	2	2	2
chr9:66177001	chr9	66,177,001	66,178,000	3	3	3	3	3	3	2	2	2	2	2	2	3	2	1	2	2	2	2	2	2
chr9:66178001	chr9	66,178,001	66,179,000	3	3	3	3	2	3	2	2	2	2	2	2	3	2	1	2	2	2	2	2	2
chr9:66179001	chr9	66,179,001	66,180,000	3	3	3	3	2	3	2	2	2	2	2	2	3	2	1	2	2	2	2	2	2
chr9:66180001	chr9	66,180,001	66,181,000	3	3	3	3	2	3	2	2	2	2	2	2	3	2	1	2	2	2	2	2	2
chr9:66181001	chr9	66,181,001	66,182,000	3	3	3	3	2	3	2	2	2	2	2	2	3	2	1	2	2	2	2	2	2
chr9:67434001	chr9	67,434,001	67,435,000	4	3	3	2	2	2	2	2	2	2	2	2	2	2	2	2	2	2	2	2	2
chr9:67435001	chr9	67,435,001	67,436,000	4	3	3	2	2	2	2	2	2	2	2	2	2	2	2	2	2	2	2	2	2
chr9:67436001	chr9	67,436,001	67,437,000	4	3	3	2	2	2	1	2	1	2	2	2	2	2	2	2	2	2	2	2	2
chr9:67622001	chr9	67,622,001	67,623,000	4	3	3	2	2	2	2	2	2	2	2	1	2	2	2	3	2	2	2	2	2
chr9:68335001	chr9	68,335,001	68,336,000	3	2	3	2	2	3	2	2	2	2	2	2	2	2	2	2	2	2	2	2	2
chr9:81711001	chr9	81,711,001	81,712,000	2	4	3	2	2	2	2	2	2	2	2	2	2	2	2	2	2	2	2	2	2
chr13:18409001	chr13	18,409,001	18,410,000	3	4	3	2	3	2	2	3	2	2	2	2	2	2	2	2	2	2	2	2	2
chr13:18534001	chr13	18,534,001	18,535,000	2	3	3	2	2	2	2	3	2	2	2	2	2	2	2	2	2	2	2	2	2
chr13:18535001	chr13	18,535,001	18,536,000	2	3	3	2	2	2	2	3	2	2	2	2	2	2	2	2	2	2	2	2	2
chr13:18536001	chr13	18,536,001	18,537,000	2	3	3	2	2	2	2	3	2	2	2	2	2	2	2	2	2	2	2	2	2
chr13:18537001	chr13	18,537,001	18,538,000	2	3	3	2	2	2	2	3	2	2	2	2	2	2	2	2	2	2	2	2	2
chr13:18538001	chr13	18,538,001	18,539,000	2	3	3	2	2	2	2	3	2	2	2	2	2	2	2	2	2	2	2	2	2
chr13:18539001	chr13	18,539,001	18,540,000	2	3	3	2	2	2	2	3	2	2	2	2	2	2	2	2	2	2	2	2	2
chr13:52289001	chr13	52,289,001	52,290,000	2	3	3	2	2	2	2	2	2	2	2	2	2	2	2	2	2	2	2	2	2
chr13:52290001	chr13	52,290,001	52,291,000	2	3	3	2	2	2	2	2	2	2	2	2	2	2	2	2	2	2	2	2	2
chr13:52291001	chr13	52,291,001	52,292,000	2	3	3	2	2	2	2	2	2	2	2	2	2	2	2	2	2	2	2	2	2
chr13:52292001	chr13	52,292,001	52,293,000	2	3	3	2	2	2	2	2	2	2	2	2	2	2	2	2	2	2	2	2	2
chr13:52293001	chr13	52,293,001	52,294,000	2	3	3	2	2	2	2	2	2	2	2	2	2	2	2	2	2	2	2	2	2
chr13:52294001	chr13	52,294,001	52,295,000	2	3	3	2	2	2	2	2	2	2	2	2	2	2	2	2	2	2	2	2	2

Region	CHR	start	end	HD2	HD3	HD1	BN1	BN2	BN3	BN4	BN5	BN6	LC1	LC2	LC3	LC4	LC5	LC6	LC7	LC8	LC9	LC10	LC10*	LC11
chr13:52295001	chr13	52,295,001	52,296,000	2	3	3	2	2	2	2	2	2	2	2	2	2	2	2	2	2	2	2	2	2
chr13:52296001	chr13	52,296,001	52,297,000	2	3	3	2	2	2	2	2	2	2	2	2	2	2	2	2	2	2	2	2	2
chr13:52297001	chr13	52,297,001	52,298,000	2	3	3	2	2	2	2	2	2	2	2	2	2	2	2	2	2	2	2	2	2
chr13:52298001	chr13	52,298,001	52,299,000	2	3	3	2	2	2	2	2	2	2	2	2	2	2	2	2	2	2	2	2	2
chr13:52299001	chr13	52,299,001	52,300,000	2	3	3	2	2	2	2	2	2	2	2	2	2	2	2	2	2	2	2	2	2
chr13:52300001	chr13	52,300,001	52,301,000	2	3	3	2	2	2	2	2	2	2	2	2	2	2	2	2	2	2	2	2	2
chr13:52301001	chr13	52,301,001	52,302,000	2	3	3	2	2	2	2	2	2	2	2	2	2	2	2	2	2	2	2	2	2
chr13:52302001	chr13	52,302,001	52,303,000	2	3	3	2	2	2	2	2	2	2	2	2	2	2	2	2	2	2	2	2	2
chr16:34114001	chr16	34,114,001	34,115,000	3	3	3	2	2	2	2	2	2	2	2	2	2	2	2	2	2	2	2	2	2
chr16:34115001	chr16	34,115,001	34,116,000	3	3	3	2	2	2	2	2	2	2	2	2	2	2	2	2	2	2	2	2	2
chr16:34116001	chr16	34,116,001	34,117,000	3	3	3	2	2	2	2	2	2	2	2	2	2	2	2	2	2	2	2	2	2
chr16:34117001	chr16	34,117,001	34,118,000	3	3	3	2	2	2	2	2	2	2	2	2	2	2	2	2	2	2	2	2	2
chr16:34118001	chr16	34,118,001	34,119,000	3	3	3	2	2	2	2	2	2	2	2	2	2	2	2	2	2	2	2	2	2
chr16:34119001	chr16	34,119,001	34,120,000	3	3	3	2	2	2	2	2	2	2	2	2	2	2	2	2	2	2	2	2	2
chr16:34120001	chr16	34,120,001	34,121,000	3	3	3	2	2	2	2	2	2	2	2	2	2	2	2	2	2	2	2	2	2
chr16:34121001	chr16	34,121,001	34,122,000	3	3	3	2	2	2	2	2	2	2	2	2	2	2	2	2	2	2	2	2	2
chr16:34122001	chr16	34,122,001	34,123,000	3	3	3	2	2	2	2	2	2	2	2	2	2	2	2	2	2	2	2	2	2
chr16:34123001	chr16	34,123,001	34,124,000	3	3	3	2	2	2	2	2	2	2	2	2	2	2	2	2	2	2	2	2	2
chr16:34124001	chr16	34,124,001	34,125,000	3	2	3	2	2	2	2	2	2	2	2	2	2	2	2	2	2	2	2	2	2
chr16:34125001	chr16	34,125,001	34,126,000	3	2	3	2	2	2	2	2	2	2	2	2	2	2	2	2	2	2	2	2	2
chr16:34126001	chr16	34,126,001	34,127,000	3	2	3	3	2	2	2	2	2	2	2	2	2	2	2	2	2	2	2	2	2
chr16:34127001	chr16	34,127,001	34,128,000	3	2	3	3	2	2	2	2	2	2	2	2	2	2	2	2	2	2	2	2	2
chr16:34128001	chr16	34,128,001	34,129,000	3	2	3	3	2	2	2	2	2	2	2	2	2	2	2	2	2	2	2	2	2
chr16:34129001	chr16	34,129,001	34,130,000	3	2	3	3	2	2	2	2	2	2	2	2	2	2	2	2	2	2	2	2	2
chr16:34130001	chr16	34,130,001	34,131,000	3	2	3	3	2	2	2	2	2	2	2	2	2	2	2	2	2	2	2	2	2
chr16:34131001	chr16	34,131,001	34,132,000	3	2	3	2	2	2	2	2	2	2	2	2	2	2	2	2	2	2	2	2	2
chr16:34132001	chr16	34,132,001	34,133,000	3	2	3	2	2	2	2	2	2	2	2	2	2	2	2	2	2	2	2	2	2
chr16:34133001	chr16	34,133,001	34,134,000	3	2	3	2	2	2	2	2	2	2	2	2	2	2	2	2	2	2	2	2	2
chr16:34134001	chr16	34,134,001	34,135,000	3	2	3	2	2	2	2	2	2	2	2	2	2	2	2	2	2	2	2	2	2
chr16:34135001	chr16	34,135,001	34,136,000	3	2	3	2	2	2	2	2	2	2	2	2	2	2	2	2	2	2	2	2	2
chr16:34136001	chr16	34,136,001	34,137,000	3	2	3	2	2	2	2	2	2	2	2	2	2	2	2	2	2	2	2	2	2
chr16:34137001	chr16	34,137,001	34,138,000	3	2	3	2	2	2	2	2	2	2	2	2	2	2	2	2	2	2	2	2	2
chr16:34138001	chr16	34,138,001	34,139,000	3	2	3	2	2	2	2	2	2	2	2	2	2	2	2	2	2	2	2	2	2
chr16:34139001	chr16	34,139,001	34,140,000	3	2	3	2	2	2	2	2	2	2	2	2	2	2	2	2	2	2	2	2	2
chr16:34140001	chr16	34,140,001	34,141,000	3	2	3	2	2	2	2	2	2	2	2	2	2	2	2	2	2	2	2	2	2
chr16:34141001	chr16	34,141,001	34,142,000	3	2	3	2	2	2	2	2	2	2	2	2	2	2	2	2	2	2	2	2	2
chr16:34142001	chr16	34,142,001	34,143,000	3	2	3	2	2	2	2	2	2	2	2	2	2	2	2	2	2	2	2	2	2
chr16:34143001	chr16	34,143,001	34,144,000	3	3	3	2	2	2	2	2	2	2	2	2	2	2	2	2	2	2	2	2	2
chr16:34144001	chr16	34,144,001	34,145,000	3	3	3	2	2	2	2	2	2	2	2	2	2	2	2	2	2	2	2	2	2
chr16:34145001	chr16	34,145,001	34,146,000	3	3	3	2	2	2	2	2	2	2	2	2	2	2	2	2	2	2	2	2	2
chr16:34146001	chr16	34,146,001	34,147,000	3	3	3	2	2	2	2	2	2	2	2	2	2	2	2	2	2	2	2	2	2
chr16:34147001	chr16	34,147,001	34,148,000	3	3	3	2	2	2	2	2	2	2	2	2	2	2	2	2	2	2	2	2	2
chr16:34148001	chr16	34,148,001	34,149,000	3	3	3	2	2	2	2	2	2	2	2	2	2	2	2	2	2	2	2	2	2
chr16:34149001	chr16	34,149,001	34,150,000	3	3	3	2	2	2	2	2	2	2	2	2	2	2	2	2	2	2	2	2	2
chr16:36144001	chr16	36,144,001	36,145,000	3	2	3	2	2	2	2	2	2	2	2	2	2	2	2	2	2	2	2	2	2
chr16:36145001	chr16	36,145,001	36,146,000	3	2	3	2	2	2	2	2	2	2	2	2	2	2	2	2	2	2	2	2	2
chr16:36161001	chr16	36,161,001	36,162,000	3	2	3	2	2	2	2	2	2	2	2	2	2	2	2	2	2	2	2	2	2
chr16:36162001	chr16	36,162,001	36,163,000	3	2	3	2	2	2	2	2	2	2	2	2	2	2	2	2	2	2	2	2	2
chr16:36163001	chr16	36,163,001	36,164,000	3	2	3	2	2	2	2	2	2	2	2	2	2	2	2	2	2	2	2	2	2
chr16:36164001	chr16	36,164,001	36,165,000	3	2	3	2	2	2	2	2	2	2	2	2	2	2	2	2	2	2	2	2	2
chr16:36165001	chr16	36,165,001	36,166,000	3	2	3	2	2	2	2	2	2	2	2	2	2	2	2	2	2	2	2	2	2
chr17:18444001	chr17	18,444,001	18,445,000	3	3	3	2	2	2	2	2	2	2	1	2	2	2	2	2	2	3	2	2	2
chr19:55759001	chr19	55,759,001	55,760,000	3	2	3	2	2	2	2	2	2	2	2	2	2	2	2	2	2	2	2	2	2
chr19:55760001	chr19	55,760,001	55,761,000	3	2	3	1	2	2	2	2	2	2	2	2	2	2	2	2	2	2	2	2	2
chr19:55761001	chr19	55,761,001	55,762,000	3	2	3	1	2	2	2	2	2	2	2	2	2	2	2	2	2	2	2	2	2
chr20:26087001	chr20	26,087,001	26,088,000	3	2	3	2	2	2	2	2	2	2	2	2	2	2	2	2	2	2	2	2	2
chr21:5255001	chr21	5,255,001	5,256,000	5	2	3	2	2	2	2	2	2	2	2	2	2	2	2	2	2	2	2	2	2
chr21:7246001	chr21	7,246,001	7,247,000	3	3	3	2	2	2	2	2	2	2	2	2	2	2	2	2	2	2	2	2	2
chr21:7247001	chr21	7,247,001	7,248,000	3	3	3	2	2	2	2	2	2	2	2	2	2	2	2	2	2	2	2	2	2
chr21:7248001	chr21	7,248,001	7,249,000	3	3	3	2	2	2	2	2	2	2	2	2	2	2	2	2	2	2	2	2	2
chr21:7249001	chr21	7,249,001	7,250,000	3	3	3	2	2	2	2	2	2	2	2	2	2	2	2	2	2	2	2	2	2
chr21:7250001	chr21	7,250,001	7,251,000	3	3	3	2	2	2	2	2	2	2	2	2	2	2	2	2	2	2	2	2	2
chr21:7251001	chr21	7,251,001	7,252,000	3	3	3	2	2	2	2	2	2	2	2	2	2	2	2	2	2	2	2	2	2
chr21:7252001	chr21	7,252,001	7,253,000	3	3	3	2	2	2	2	2	2	2	2	2	2	2	2	2	2	2	2	2	2
chr21:7253001	chr21	7,253,001	7,254,000	3	3	3	2	2	2	2	2	2	2	2	2	2	2	2	2	2	2	2	2	2
chr21:7254001	chr21	7,254,001	7,255,000	3	3	3	2	2	2	2	2	2	2	2	2	2	2	2	2	2	2	2	2	2
chr21:7255001	chr21	7,255,001	7,256,000	3	3	3	2	2	2	2	2	2	2	2	2	2	2	2	2	2	2	2	2	2
chr21:7256001	chr21	7,256,001	7,257,000	3	3	3	2	2	2	2	2	2	2	2	2	2	2	2	2	2	2	2	2	2
chr21:7257001	chr21	7,257,001	7,258,000	3	3	3	2	2	2	2	2	2	2	2	2	2	2	2	2	2	2	2	2	2
chr21:7258001	chr21	7,258,001	7,259,000	3	3	3	2	2	2	2	2	2	2	2	2	2	2	2	2	2	2	2	2	2
chr21:7259001	chr21	7,259,001	7,260,000	3	3	3	2	2	2	2	2	2	2	2	2	2	2	2	2	2	2	2	2	2
chr21:7260001	chr21	7,260,001	7,261,000	3	3	3	2	2	2	2	2	2	2	2	2	2	2	2	2	2	2	2	2	2
chr21:7261001	chr21	7,261,001	7,262,000	3	3	3	2	2	2	2	2	2	2	2	2	2	2	2	2	2	2	2	2	2
chr21:7262001	chr21	7,262,001	7,263,000	3	2	3	2	2	2	2	2	2	2	2	2	2	2	2	2	2	2	2	2	2
chr21:7263001	chr21	7,263,001	7,264,000	3	2	3	2	2	2	2	2	2	2	2	2	2	2	2	2	2	2	2	2	2
chr21:7264001	chr21	7,264,001	7,265,000	3	2	3	2	2	2	2	2	2	2	2	2	2	2	2	2	2	2	2	2	2
chr21:7265001	chr21	7,265,001	7,266,000	3	2	3	2	2	2	2	2	2	2	2	2	2	2	2	2	2	2	2	2	2
chr21:7266001	chr21	7,266,001	7,267,000	3	2	3	2	2	2	2	2	2	2	2	2	2	2	2	2	2	2	2	2	2
chr21:7267001	chr21	7,267,001	7,268,000	3	2	3	2	2	2	2	2	2	2	2	2	2	2	2	2	2	2	2	2	2
chr21:7268001	chr21	7,268,001	7,269,000	3	2	3	2	2	2	2	2	2	2	2	2	2	2	2	2	2	2	2	2	2
chr21:7269001	chr21	7,269,001	7,270,000	3	2	3	2	2	2	2	2	2	2	2	2	2	2	2	2	2	2	2	2	2
chr21:7270001	chr21	7,270,001	7,271,000	3	2	3	2	2	2	2	2	2	2	2	2	2	2	2	2	2	2	2	2	2
chr21:7271001	chr21	7,271,001	7,272,000	3	2	3	2	2	2	2	2	2	2	2	2	2	2	2	2	2	2	2	2	2
chr21:7272001	chr21	7,272,001	7,273,000	3	2	3	2	2	2	2	2	2	2	2	2	2	2	2	2	2	2	2	2	2
chr21:7273001	chr21	7,273,001	7,274,000	3	2	3	2	2	2	2	2	2	2	2	2	2	2	2	2	2	2	2	2	2
chr21:7274001	chr21	7,274,001	7,275,000	3	2	3	2	2	2	2	2	2	2	2	2	2	2	2	2	2	2	2	2	2
chr21:7275001	chr21	7,275,001	7,276,000	3	2	3	2	2	2	2	2	2	2	2	2	2	2	2	2	2	2	2	2	2
chr21:7276001	chr21	7,276,001	7,277,000	3	2	3	2	2	2	2	2	2	2	2	2	2	2	2	2	2	2	2	2	2
chr21:7293001	chr21	7,293,001	7,294,000	3	2	3	2	2	2	2	2	3	2	2	2	2	2	2	2	2	2	2	2	2
chr21:7294001	chr21	7,294,001	7,295,000	3	2	3	2	2	2	2	2	3	2	2	2	2	2	2	2	2	2	2	2	2
chr21:7295001	chr21	7,295,001	7,296,000	3	2	3	2	2	2	2	2	2	2	2	2	2	2	2	2	2	2	2	2	2
chr21:7296001	chr21	7,296,001	7,297,000	3	2	3	2	2	2	2	2	2	2	2	2	2	2	2	2	2	2	2	2	2
chr21:7297001	chr21	7,297,001	7,298,000	3	2	3	2	2	2	2	2	2	2	2	2	2	2	2	2	2	2	2	2	2
chr21:7313001	chr21	7,313,001	7,314,000	3	2	3	2	2	2	2	2	2	2	2	2	2	2	2	2	2	2	2	2	2
chr21:7314001	chr21	7,314,001	7,315,000	3	2	3	2	2	2	2	2	2	2	2	2	2	2	2	2	2	2	2	2	2
chr21:7315001	chr21	7,315,001	7,316,000	3	2	3	2	2	2	2	2	2	2	2	2	2	2	2	2	2	2	2	2	2
chr21:7316001	chr21	7,316,001	7,317,000	3	2	3	2	2	2	2	2	2	2	2	2	2	2	2	2	2	2	2	2	2
chr21:7317001	chr21	7,317,001	7,318,000	3	2	3	2	2	2	2	2	2	2	2	2	2	2	2	2	2	2	2	2	2
chr21:7318001	chr21	7,318,001	7,319,000	3	2	3	2	2	2	2	2	2	2	2	2	2	2	2	2	2	2	2	2	2
chr21:7319001	chr21	7,319,001	7,320,000	3	2	3	2	2	2	2	2	2	2	2	2	2	2	2	2	2	2	2	2	2
chr21:7320001	chr21	7,320,001	7,321,000	3	2	3	2	2	2	2	2	2	2	2	2	2	2	2	2	2	2	2	2	2
chr21:7321001	chr21	7,321,001	7,322,000	3	2	3	2	2	2	2	2	2	2	2	2	2	2	2	2	2	2	2	2	2
chr21:7322001	chr21	7,322,001	7,323,000	3	2	3	2	2	2	2	2	2	2	2	2	2	2	2	2	2	2	2	2	2
chr21:7323001	chr21	7,323,001	7,324,000	3	2	3	2	2	2	2	2	2	2	2	2	2	2	2	2	2	2	2	2	2
chr21:7324001	chr21	7,324,001	7,325,000	3	2	3	2	2	2	2	2	2	2	2	2	2	2	2	2	2	2	2	2	2
chr21:7325001	chr21	7,325,001	7,326,000	3	2	3	2	2	2	2	2	2	2	2	2	2	2	2	2	2	2	2	2	2
chr21:7326001	chr21	7,326,001	7,327,000	3	2	3	2	2	2	2	2	2	2	2	2	2	2	2	2	2	2	2	2	2
chr21:7327001	chr21	7,327,001	7,328,000	3	2	3	2	2	2	2	2	2	2	2	2	2	2	2	2	2	2	2	2	2
chr21:8989001	chr21	8,989,001	8,990,000	3	2	3	2	2	2	2	2	2	2	2	2	2	2	2	2	2	2	2	2	2
chr21:8990001	chr21	8,990,001	8,991,000	3	2	3	2	2	2	2	2	2	2	2	2	2	2	2	2	2	2	2	2	2
chr21:8991001	chr21	8,991,001	8,992,000	3	2	3	2	2	2	2	2	2	2	2	2	2	2	2	2	2	2	2	2	2
chr21:9841001	chr21	9,841,001	9,842,000	3	2	3	2	2	2	2	2	2	2	2	2	2	2	2	2	2	2	2	2	2
chr21:9842001	chr21	9,842,001	9,843,000	3	2	3	2	2	2	2	2	2	2	2	2	2	2	2	2	2	2	2	2	2

Region	CHR	start	end	HD2	HD3	HD1	BN1	BN2	BN3	BN4	BN5	BN6	LC1	LC2	LC3	LC4	LC5	LC6	LC7	LC8	LC9	LC10	LC10*	LC11
chr21:9843001	chr21	9,843,001	9,844,000	3	2	3	2	2	2	2	2	2	2	2	2	2	2	2	2	2	2	2	2	2
chr21:9844001	chr21	9,844,001	9,845,000	3	3	3	2	2	2	2	1	2	2	2	2	2	2	2	2	2	2	2	2	2
chr21:9845001	chr21	9,845,001	9,846,000	3	3	3	2	2	2	2	1	2	2	2	2	2	2	2	2	2	2	2	2	2
chr21:9846001	chr21	9,846,001	9,847,000	3	3	3	2	2	2	2	1	2	2	2	2	2	2	2	2	2	2	2	2	2
chr21:9849001	chr21	9,849,001	9,850,000	3	3	3	2	2	2	2	1	2	2	2	2	2	2	2	2	2	2	2	2	2
chr21:9850001	chr21	9,850,001	9,851,000	3	3	3	2	2	2	2	1	2	2	2	2	2	2	2	2	2	2	2	2	2
chr21:9851001	chr21	9,851,001	9,852,000	3	3	3	2	2	2	2	1	2	2	2	2	2	2	2	2	2	2	2	2	2
chr21:9852001	chr21	9,852,001	9,853,000	3	3	3	2	2	2	2	1	2	2	2	2	2	2	2	2	2	2	2	2	2
chr21:9853001	chr21	9,853,001	9,854,000	3	3	3	2	2	2	2	1	2	2	2	2	2	2	2	2	2	2	2	2	2
chr21:9938001	chr21	9,938,001	9,939,000	3	2	3	2	2	2	2	2	2	2	2	2	2	2	2	2	2	2	2	2	2
chr21:9939001	chr21	9,939,001	9,940,000	3	2	3	2	2	2	2	2	2	2	2	2	2	2	2	2	2	2	2	2	2
chr21:9940001	chr21	9,940,001	9,941,000	3	2	3	2	2	2	2	2	2	2	2	2	2	2	2	2	2	2	2	2	2
chr21:9941001	chr21	9,941,001	9,942,000	3	2	3	2	2	2	2	2	2	2	2	2	2	2	2	2	2	2	2	2	2
chr21:9942001	chr21	9,942,001	9,943,000	3	2	3	2	2	2	2	2	2	2	2	2	2	2	2	2	2	2	2	2	2
chr21:9943001	chr21	9,943,001	9,944,000	3	2	3	2	2	2	2	2	2	2	2	2	2	2	2	2	2	2	2	2	2
chr21:9944001	chr21	9,944,001	9,945,000	3	2	3	2	2	2	2	2	2	2	2	2	2	2	2	2	2	2	2	2	2
chr21:9945001	chr21	9,945,001	9,946,000	3	2	3	2	2	2	2	2	2	2	2	2	2	2	2	2	2	2	2	2	2
chr21:9946001	chr21	9,946,001	9,947,000	3	2	3	2	2	2	2	2	2	2	2	2	2	2	2	2	2	2	2	2	2
chr21:9947001	chr21	9,947,001	9,948,000	3	2	3	2	2	2	2	2	2	2	2	2	2	2	2	2	2	2	2	2	2
chr21:9948001	chr21	9,948,001	9,949,000	3	2	3	2	2	2	2	2	2	2	2	2	2	2	2	2	2	2	2	2	2
chr21:9949001	chr21	9,949,001	9,950,000	3	2	3	2	2	2	2	2	2	2	2	2	2	2	2	2	2	2	2	2	2
chr21:9950001	chr21	9,950,001	9,951,000	3	2	3	2	2	2	2	2	2	2	2	2	2	2	2	2	2	2	2	2	2
chr21:9951001	chr21	9,951,001	9,952,000	3	3	3	2	2	2	2	2	2	2	2	2	2	2	2	2	2	2	2	2	2
chr21:9952001	chr21	9,952,001	9,953,000	3	3	3	2	2	2	2	2	2	2	2	2	2	2	2	2	2	2	2	2	2
chr21:9953001	chr21	9,953,001	9,954,000	3	3	3	2	2	2	2	2	2	2	2	2	2	2	2	2	2	2	2	2	2
chr21:9954001	chr21	9,954,001	9,955,000	2	3	3	2	2	2	2	1	2	2	2	2	2	2	2	2	2	2	2	2	2
chr21:9955001	chr21	9,955,001	9,956,000	2	3	3	2	2	2	2	1	2	2	2	2	2	2	2	2	2	2	2	2	2
chr21:9961001	chr21	9,961,001	9,962,000	3	3	3	2	2	2	2	1	2	2	2	2	2	2	2	2	2	2	2	2	2
chr21:9962001	chr21	9,962,001	9,963,000	3	3	3	2	2	2	2	1	2	2	2	2	2	2	2	2	2	2	2	2	2
chr21:9963001	chr21	9,963,001	9,964,000	3	3	3	2	2	2	2	1	2	2	2	2	2	2	2	2	2	2	2	2	2
chr21:9964001	chr21	9,964,001	9,965,000	3	3	3	2	2	2	2	1	2	2	2	2	2	2	2	2	2	2	2	2	2
chr21:9965001	chr21	9,965,001	9,966,000	3	3	3	2	2	2	2	1	2	2	2	2	2	2	2	2	2	2	2	2	2
chr21:9966001	chr21	9,966,001	9,967,000	3	3	3	2	2	2	2	1	2	2	2	2	2	2	2	2	2	2	2	2	2
chr21:9967001	chr21	9,967,001	9,968,000	3	3	3	2	2	2	2	1	2	2	2	2	2	2	2	2	2	2	2	2	2
chr21:9968001	chr21	9,968,001	9,969,000	3	3	3	2	2	2	2	1	2	2	2	2	2	2	2	2	2	2	2	2	2
chr21:9969001	chr21	9,969,001	9,970,000	3	3	3	2	2	2	2	2	2	2	2	2	2	2	2	2	2	2	2	2	2
chr21:9970001	chr21	9,970,001	9,971,000	3	3	3	2	2	2	2	2	2	2	2	2	2	2	2	2	2	2	2	2	2
chr21:9971001	chr21	9,971,001	9,972,000	3	3	3	2	2	2	2	2	2	2	2	2	2	2	2	2	2	2	2	2	2
chr21:9972001	chr21	9,972,001	9,973,000	3	3	3	2	2	2	2	2	2	2	2	2	2	2	2	2	2	2	2	2	2
chr21:9973001	chr21	9,973,001	9,974,000	3	3	3	2	2	2	2	2	2	2	2	2	2	2	2	2	2	2	2	2	2
chr21:9974001	chr21	9,974,001	9,975,000	3	3	3	2	2	2	2	2	2	2	2	2	2	2	2	2	2	2	2	2	2
chr21:9975001	chr21	9,975,001	9,976,000	3	3	3	2	2	2	2	2	2	2	2	2	2	2	2	2	2	2	2	2	2
chr21:9976001	chr21	9,976,001	9,977,000	3	3	3	2	2	2	2	2	2	2	2	2	2	2	2	2	2	2	2	2	2
chr21:9977001	chr21	9,977,001	9,978,000	3	3	3	2	2	2	2	2	2	2	2	2	2	2	2	2	2	2	2	2	2
chr21:9978001	chr21	9,978,001	9,979,000	3	3	3	2	2	2	2	2	2	2	2	2	2	2	2	2	2	2	2	2	2
chr21:9979001	chr21	9,979,001	9,980,000	3	3	3	2	2	2	2	2	2	2	2	2	2	2	2	2	2	2	2	2	2
chr21:9980001	chr21	9,980,001	9,981,000	3	3	3	2	2	2	2	2	2	2	2	2	2	2	2	2	2	2	2	2	2
chr21:9981001	chr21	9,981,001	9,982,000	3	3	3	2	2	2	2	2	2	2	2	2	2	2	2	2	2	2	2	2	2
chr21:9982001	chr21	9,982,001	9,983,000	3	3	3	2	2	2	2	2	2	2	2	2	2	2	2	2	2	2	2	2	2
chr21:9983001	chr21	9,983,001	9,984,000	3	3	3	2	2	2	2	2	2	2	2	2	2	2	2	2	2	2	2	2	2
chr21:9984001	chr21	9,984,001	9,985,000	3	3	3	2	2	2	2	2	2	2	2	2	2	2	2	2	2	2	2	2	2
chr21:10376001	chr21	10,376,001	10,377,000	2	3	3	2	2	2	2	2	2	2	2	2	2	2	2	2	2	2	2	2	2
chr21:10377001	chr21	10,377,001	10,378,000	2	3	3	2	2	2	2	2	2	2	2	2	2	2	2	2	2	2	2	2	2
chr21:10378001	chr21	10,378,001	10,379,000	2	3	3	2	2	2	2	2	2	2	2	2	2	2	2	2	2	2	2	2	2
chr21:10379001	chr21	10,379,001	10,380,000	2	3	3	2	2	2	2	2	2	2	2	2	2	2	2	2	2	2	2	2	2
chr21:10380001	chr21	10,380,001	10,381,000	2	3	3	2	2	2	2	2	2	2	2	2	2	2	2	2	2	2	2	2	2
chr21:10381001	chr21	10,381,001	10,382,000	2	3	3	2	2	2	2	2	2	2	2	2	2	2	2	2	2	2	2	2	2
chr21:10382001	chr21	10,382,001	10,383,000	2	3	3	2	2	2	2	2	2	2	2	2	2	2	2	2	2	2	2	2	2
chr21:10383001	chr21	10,383,001	10,384,000	2	3	3	2	2	2	2	2	2	2	2	2	2	2	2	2	2	2	2	2	2
chr21:10384001	chr21	10,384,001	10,385,000	2	3	3	2	2	2	2	2	2	2	2	2	2	2	2	2	2	2	2	2	2
chr21:10385001	chr21	10,385,001	10,386,000	2	3	3	2	2	2	2	2	2	2	2	2	2	2	2	2	2	2	2	2	2
chr21:10386001	chr21	10,386,001	10,387,000	2	3	3	2	2	2	2	2	2	2	2	2	2	2	2	2	2	2	2	2	2
chr21:10387001	chr21	10,387,001	10,388,000	2	3	3	2	2	2	2	2	2	2	2	2	2	2	2	2	2	2	2	2	2
chr21:10718001	chr21	10,718,001	10,719,000	3	2	3	2	2	2	2	2	2	2	2	2	2	2	2	2	2	2	2	2	2
chr21:10719001	chr21	10,719,001	10,720,000	3	2	3	2	2	2	2	2	2	2	2	2	2	2	2	2	2	2	2	2	2
chr21:10720001	chr21	10,720,001	10,721,000	3	2	3	2	2	2	2	2	2	2	2	2	2	2	2	2	2	2	2	2	2
chr21:10721001	chr21	10,721,001	10,722,000	3	2	3	2	2	2	2	2	2	2	2	2	2	2	2	2	2	2	2	2	2
chr21:10722001	chr21	10,722,001	10,723,000	3	2	3	2	2	2	2	2	2	2	2	2	2	2	2	2	2	2	2	2	2
chr21:10723001	chr21	10,723,001	10,724,000	3	2	3	2	2	2	2	2	2	2	2	2	2	2	2	2	2	2	2	2	2
chr21:10724001	chr21	10,724,001	10,725,000	3	2	3	2	2	2	2	2	2	2	2	2	2	2	2	2	2	2	2	2	2
chr21:10725001	chr21	10,725,001	10,726,000	3	2	3	2	2	2	2	2	2	2	2	2	2	2	2	2	2	2	2	2	2
chr21:10726001	chr21	10,726,001	10,727,000	3	2	3	2	2	2	2	2	2	2	2	2	2	2	2	2	2	2	2	2	2
chr21:10727001	chr21	10,727,001	10,728,000	3	2	3	2	2	2	2	2	2	2	2	2	2	2	2	2	2	2	2	2	2
chr21:10728001	chr21	10,728,001	10,729,000	3	2	3	2	2	2	2	2	2	2	2	2	2	2	2	2	2	2	2	2	2
chr21:10729001	chr21	10,729,001	10,730,000	3	2	3	2	2	2	2	2	2	2	2	2	2	2	2	2	2	2	2	2	2
chr21:10730001	chr21	10,730,001	10,731,000	3	2	3	2	2	2	2	2	2	2	2	2	2	2	2	2	2	2	2	2	2
chr21:10731001	chr21	10,731,001	10,732,000	3	2	3	2	2	2	2	2	2	2	2	2	2	2	2	2	2	2	2	2	2
chr21:10732001	chr21	10,732,001	10,733,000	3	2	3	2	2	2	2	2	2	2	2	2	2	2	2	2	2	2	2	2	2
chr21:10733001	chr21	10,733,001	10,734,000	3	2	3	2	2	2	2	2	2	2	2	2	2	2	2	2	2	2	2	2	2
chr21:10734001	chr21	10,734,001	10,735,000	3	2	3	2	2	2	2	2	2	2	2	2	2	2	2	2	2	2	2	2	2
chr21:10735001	chr21	10,735,001	10,736,000	3	2	3	2	2	2	2	2	2	2	2	2	2	2	2	2	2	2	2	2	2
chr21:10736001	chr21	10,736,001	10,737,000	3	2	3	2	2	2	2	2	2	2	2	2	2	2	2	2	2	2	2	2	2
chr21:10737001	chr21	10,737,001	10,738,000	3	2	3	2	2	2	2	2	2	2	2	2	2	2	2	2	2	2	2	2	2
chr21:10738001	chr21	10,738,001	10,739,000	3	2	3	2	2	2	2	2	2	2	2	2	2	2	2	2	2	2	2	2	2
chr21:10739001	chr21	10,739,001	10,740,000	3	2	3	2	2	2	2	2	2	2	2	2	2	2	2	2	2	2	2	2	2
chr21:10743001	chr21	10,743,001	10,744,000	3	3	3	2	2	2	2	2	2	2	2	2	2	1	2	3	2	2	2	2	2
chr21:10744001	chr21	10,744,001	10,745,000	3	3	3	2	2	2	2	2	2	2	2	2	2	1	2	3	2	2	2	2	2
chr21:10781001	chr21	10,781,001	10,782,000	3	2	3	2	2	2	2	2	2	2	2	2	2	2	2	2	2	2	2	2	2
chr21:10782001	chr21	10,782,001	10,783,000	3	2	3	2	2	2	2	2	2	2	2	2	2	2	2	2	2	2	2	2	2
chr21:10783001	chr21	10,783,001	10,784,000	3	2	3	2	2	2	2	2	2	2	2	2	2	2	2	2	2	2	2	2	2
chr21:10784001	chr21	10,784,001	10,785,000	3	2	3	2	2	2	2	2	2	2	2	2	2	2	2	2	2	2	2	2	2
chr21:10785001	chr21	10,785,001	10,786,000	3	2	3	2	2	2	2	2	2	2	2	2	2	2	2	2	2	2	2	2	2
chr21:10786001	chr21	10,786,001	10,787,000	3	2	3	2	2	2	2	2	2	2	2	2	2	2	2	2	2	2	2	2	2
chr21:10787001	chr21	10,787,001	10,788,000	3	2	3	2	2	2	2	2	2	2	2	2	2	2	2	2	2	2	2	2	2
chr21:10796001	chr21	10,796,001	10,797,000	3	2	3	2	2	2	2	2	2	2	2	2	2	2	2	2	2	2	2	2	2
chr21:10797001	chr21	10,797,001	10,798,000	3	2	3	2	2	2	2	2	2	2	2	2	2	2	2	2	2	2	2	2	2
chr21:10798001	chr21	10,798,001	10,799,000	3	2	3	2	2	2	2	2	2	2	2	2	2	2	2	2	2	2	2	2	2
chr21:10799001	chr21	10,799,001	10,800,000	3	2	3	2	2	2	2	2	2	2	2	2	2	2	2	2	2	2	2	2	2
chr21:10800001	chr21	10,800,001	10,801,000	3	2	3	2	2	2	2	2	2	2	2	2	2	2	2	2	2	2	2	2	2
chr21:10801001	chr21	10,801,001	10,802,000	3	2	3	2	2	2	2	2	2	2	2	2	2	2	2	2	2	2	2	2	2
chr21:10802001	chr21	10,802,001	10,803,000	3	2	3	2	2	2	2	2	2	2	2	2	2	2	2	2	2	2	2	2	2
chr21:10803001	chr21	10,803,001	10,804,000	3	2	3	2	2	2	2	2	2	2	2	2	2	2	2	2	2	2	2	2	2
chr21:10804001	chr21	10,804,001	10,805,000	3	2	3	2	2	2	2	2	2	2	2	2	2	2	2	2	2	2	2	2	2
chr21:10805001	chr21	10,805,001	10,806,000	3	2	3	2	2	2	2	2	2	2	2	2	2	2	2	2	2	2	2	2	2
chr21:10806001	chr21	10,806,001	10,807,000	3	2	3	2	2	2	2	2	2	2	2	2	2	2	2	2	2	2	2	2	2
chr21:10807001	chr21	10,807,001	10,808,000	3	2	3	2	2	2	2	2	2	2	2	2	2	2	2	2	2	2	2	2	2
chr21:10808001	chr21	10,808,001	10,809,000	3	2	3	2	2	2	2	2	2	2	2	2	2	2	2	2	2	2	2	2	2
chr21:10809001	chr21	10,809,001	10,810,000	3	2	3	2	2	2	2	2	2	2	2	2	2	2	2	2	2	2	2	2	2
chr21:10810001	chr21	10,810,001	10,811,000	3	2	3	2	2	2	2	2	2	2	2	2	2	2	2	2	2	2	2	2	2
chr21:10811001	chr21	10,811,001	10,812,000	3	2	3	2	2	2	2	2	2	2	2	2	2	2	2	2	2	2	2	2	2
chr21:10812001	chr21	10,812,001	10,813,000	3	2	3	2	2	2	2	2	2	2	2	2	2	2	2	2	2	2	2	2	2
chr21:10813001	chr21	10,813,001	10,814,000	3	2	3	2	2	2	2	2	2	2	2	2	2	2	2	2	2	2	2	2	2
chr21:10814001	chr21	10,814,001	10,815,000	3	2	3	2	2	2	2	2	2	2	2	2	2	2	2	2	2	2	2	2	2

Region	CHR	start	end	HD2	HD3	HD1	BN1	BN2	BN3	BN4	BN5	BN6	LC1	LC2	LC3	LC4	LC5	LC6	LC7	LC8	LC9	LC10	LC10*	LC11
chr22:10567001	chr22	10,567,001	10,568,000	3	3	3	3	2	2	2	2	2	2	2	2	2	2	2	2	2	2	2	2	2
chr22:10689001	chr22	10,689,001	10,690,000	3	3	3	3	2	2	2	2	2	2	2	2	2	2	2	2	2	2	2	2	2
chr22:11935001	chr22	11,935,001	11,936,000	3	2	3	2	2	2	2	2	2	2	2	2	2	2	2	2	2	2	2	2	2
chr1:768001	chr1	768,001	769,000	2	2	2	3	3	3	2	2	2	2	2	2	2	2	2	2	2	2	2	2	2
chr1:769001	chr1	769,001	770,000	2	2	2	3	3	3	2	2	2	2	2	2	2	2	2	2	2	2	2	2	2
chr1:793001	chr1	793,001	794,000	2	2	2	3	3	3	2	2	2	2	2	2	2	2	2	2	2	2	2	2	2
chr1:794001	chr1	794,001	795,000	2	2	2	3	3	3	2	2	2	2	2	2	2	2	2	2	2	2	2	2	2
chr1:795001	chr1	795,001	796,000	2	2	2	3	3	3	2	2	2	2	2	2	2	2	2	2	2	2	2	2	2
chr1:796001	chr1	796,001	797,000	2	2	2	3	3	3	2	2	2	2	2	2	2	2	2	2	2	2	2	2	2
chr1:797001	chr1	797,001	798,000	2	2	2	3	3	3	2	2	2	2	2	2	2	2	2	2	2	2	2	2	2
chr1:798001	chr1	798,001	799,000	2	2	2	3	3	3	2	2	2	2	2	2	2	2	2	2	2	2	2	2	2
chr1:799001	chr1	799,001	800,000	2	2	2	3	3	3	2	2	2	2	2	2	2	2	2	2	2	2	2	2	2
chr1:800001	chr1	800,001	801,000	2	2	2	3	3	3	2	2	2	2	2	2	2	2	2	2	2	2	2	2	2
chr1:801001	chr1	801,001	802,000	2	2	2	3	3	3	2	2	2	2	2	2	2	2	2	2	2	2	2	2	2
chr1:802001	chr1	802,001	803,000	2	2	2	3	3	3	2	2	2	2	2	2	2	2	2	2	2	2	2	2	2
chr1:803001	chr1	803,001	804,000	2	2	2	3	3	3	2	2	2	2	2	2	2	2	2	2	2	2	2	2	2
chr1:804001	chr1	804,001	805,000	2	2	2	3	3	3	2	2	2	2	2	2	2	2	2	2	2	2	2	2	2
chr1:805001	chr1	805,001	806,000	2	2	2	3	3	3	2	2	2	2	2	2	2	2	2	2	2	2	2	2	2
chr1:806001	chr1	806,001	807,000	2	2	2	3	3	3	2	2	2	2	2	2	2	2	2	2	2	2	2	2	2
chr1:807001	chr1	807,001	808,000	2	2	2	3	3	3	2	2	2	2	2	2	2	2	2	2	2	2	2	2	2
chr1:808001	chr1	808,001	809,000	2	2	2	3	3	3	2	2	2	2	2	2	2	2	2	2	2	2	2	2	2
chr1:809001	chr1	809,001	810,000	2	2	2	3	3	3	2	2	2	2	2	2	2	2	2	2	2	2	2	2	2
chr1:810001	chr1	810,001	811,000	2	2	2	3	3	3	2	2	2	2	2	2	2	2	2	2	2	2	2	2	2
chr1:811001	chr1	811,001	812,000	2	2	2	3	3	3	2	2	2	2	2	2	2	2	2	2	2	2	2	2	2
chr1:124941001	chr1	124,941,001	124,942,000	2	2	2	2	3	2	3	3	2	2	2	2	2	2	2	2	2	2	2	2	2
chr1:144040001	chr1	144,040,001	144,041,000	2	2	2	1	2	2	2	1	1	2	2	2	2	2	2	2	2	2	2	2	2
chr1:144041001	chr1	144,041,001	144,042,000	2	2	2	1	2	2	2	1	1	2	2	2	2	2	2	2	2	2	2	2	2
chr1:146996001	chr1	146,996,001	146,997,000	2	2	2	2	2	3	3	2	3	2	2	2	2	2	2	2	2	2	2	2	2
chr1:146997001	chr1	146,997,001	146,998,000	2	2	2	2	2	3	3	2	3	2	2	2	2	2	2	2	2	2	2	2	2
chr2:97498001	chr2	97,498,001	97,499,000	1	2	2	2	2	2	3	3	3	2	2	2	2	2	2	2	2	2	2	2	2
chr2:97502001	chr2	97,502,001	97,503,000	2	2	2	2	2	2	3	3	3	2	2	2	2	2	2	2	2	2	2	2	2
chr3:77293001	chr3	77,293,001	77,294,000	2	2	2	2	2	2	1	1	1	2	2	2	2	2	2	2	2	2	2	2	2
chr3:77294001	chr3	77,294,001	77,295,000	2	2	2	2	2	2	1	1	1	2	2	2	2	2	2	2	2	2	2	2	2
chr3:90543001	chr3	90,543,001	90,544,000	2	2	2	2	3	2	2	3	3	2	2	2	2	2	2	2	2	2	2	2	2
chr3:93785001	chr3	93,785,001	93,786,000	3	3	2	2	2	2	2	2	2	2	2	2	2	2	2	2	2	2	2	2	2
chr3:93786001	chr3	93,786,001	93,787,000	3	3	2	2	2	2	2	2	2	2	2	2	2	2	2	2	2	2	2	2	2
chr3:93787001	chr3	93,787,001	93,788,000	3	3	2	2	2	2	2	2	2	2	2	2	2	2	2	2	2	2	2	2	2
chr3:93788001	chr3	93,788,001	93,789,000	3	3	2	2	2	2	2	2	2	2	2	2	2	2	2	2	2	2	2	2	2
chr3:93789001	chr3	93,789,001	93,790,000	3	3	2	2	2	2	2	2	2	2	2	2	2	2	2	2	2	2	2	2	2
chr3:93790001	chr3	93,790,001	93,791,000	3	3	2	2	2	2	2	2	2	2	2	2	2	2	2	2	2	2	2	2	2
chr3:93796001	chr3	93,796,001	93,797,000	3	3	2	2	2	2	2	2	2	2	2	2	2	2	2	2	2	2	2	2	2
chr3:93797001	chr3	93,797,001	93,798,000	3	3	2	2	2	2	2	2	2	2	2	2	2	2	2	2	2	2	2	2	2
chr3:93798001	chr3	93,798,001	93,799,000	3	3	2	2	2	2	2	2	2	2	2	2	2	2	2	2	2	2	2	2	2
chr3:148451001	chr3	148,451,001	148,452,000	5	4	2	2	2	2	2	2	2	2	2	2	2	2	2	2	2	2	2	2	2
chr3:198100001	chr3	198,100,001	198,101,000	5	5	2	2	2	2	4	2	2	2	2	2	3	2	2	1	2	2	2	2	2
chr5:70715001	chr5	70,715,001	70,716,000	2	2	2	3	3	3	3	2	3	2	3	2	1	2	2	2	2	2	2	2	2
chr5:70716001	chr5	70,716,001	70,717,000	2	2	2	3	3	3	3	2	3	2	3	2	1	2	2	2	2	2	2	2	2
chr5:180947001	chr5	180,947,001	180,948,000	1	1	2	2	2	2	2	2	2	2	2	2	2	2	2	2	2	2	2	2	2
chr7:4588001	chr7	4,588,001	4,589,000	3	3	2	2	2	2	2	2	2	2	2	2	2	2	2	2	2	2	2	2	2
chr7:4593001	chr7	4,593,001	4,594,000	3	3	2	2	2	2	2	2	2	2	2	2	2	2	2	2	2	2	2	2	2
chr7:4594001	chr7	4,594,001	4,595,000	3	3	2	2	2	2	2	2	2	2	2	2	2	2	2	2	2	2	2	2	2
chr8:206001	chr8	206,001	207,000	2	2	2	2	3	3	2	2	3	2	2	2	2	2	2	2	2	2	2	2	2
chr8:2253001	chr8	2,253,001	2,254,000	3	3	2	2	2	2	3	2	2	2	2	2	2	2	2	2	2	2	2	2	2
chr8:4265001	chr8	4,265,001	4,266,000	1	1	2	2	2	2	2	2	2	2	2	2	2	2	2	2	2	2	2	2	2
chr8:7424001	chr8	7,424,001	7,425,000	1	1	2	4	3	3	4	2	3	0	2	2	2	2	2	3	1	2	3	3	2
chr8:7431001	chr8	7,431,001	7,432,000	1	1	2	3	2	3	3	2	2	2	2	2	3	2	2	2	1	2	2	2	2
chr8:7910001	chr8	7,910,001	7,911,000	1	1	2	3	2	3	3	2	2	2	2	2	2	2	2	2	1	2	3	2	2
chr8:12054001	chr8	12,054,001	12,055,000	1	1	2	2	2	2	2	2	2	2	2	2	2	2	2	2	2	2	2	2	2
chr9:60837001	chr9	60,837,001	60,838,000	2	2	2	2	2	2	1	1	1	2	2	2	2	2	2	2	2	2	2	2	2
chr9:60838001	chr9	60,838,001	60,839,000	2	2	2	2	2	2	1	1	1	2	2	2	2	2	2	2	2	2	2	2	2
chr9:61081001	chr9	61,081,001	61,082,000	2	2	2	2	2	2	1	1	1	2	2	2	2	2	2	2	2	2	2	2	2
chr9:61082001	chr9	61,082,001	61,083,000	2	2	2	2	2	2	1	1	1	2	2	2	2	2	2	2	2	2	2	2	2
chr9:64609001	chr9	64,609,001	64,610,000	1	1	2	2	2	2	1	2	2	2	2	2	2	2	2	2	2	2	2	2	2
chr10:47551001	chr10	47,551,001	47,552,000	2	2	2	2	3	3	2	2	3	2	2	2	2	2	2	2	2	2	2	2	2
chr10:47552001	chr10	47,552,001	47,553,000	2	2	2	2	3	3	2	2	3	2	2	2	2	2	2	2	2	2	2	2	2
chr10:47553001	chr10	47,553,001	47,554,000	2	2	2	2	3	3	2	2	3	2	2	2	2	2	2	2	2	2	2	2	2
chr10:47554001	chr10	47,554,001	47,555,000	2	2	2	2	3	3	2	2	3	2	2	2	2	2	2	2	2	2	2	2	2
chr10:47555001	chr10	47,555,001	47,556,000	2	2	2	2	3	3	2	2	3	2	2	2	2	2	2	2	2	2	2	2	2
chr10:47556001	chr10	47,556,001	47,557,000	2	2	2	2	3	3	2	2	3	2	2	2	2	2	2	2	2	2	2	2	2
chr10:47557001	chr10	47,557,001	47,558,000	2	2	2	2	3	3	2	2	3	2	2	2	2	2	2	2	2	2	2	2	2
chr11:3255001	chr11	3,255,001	3,256,000	1	1	2	2	2	2	2	2	2	2	2	2	2	2	2	2	2	2	2	2	2
chr11:50793001	chr11	50,793,001	50,794,000	3	3	2	2	2	2	2	2	2	2	2	2	2	2	2	2	2	2	2	2	2
chr11:50794001	chr11	50,794,001	50,795,000	3	3	2	2	2	2	2	2	2	2	2	2	2	2	2	2	2	2	2	2	2
chr11:65875001	chr11	65,875,001	65,876,000	2	2	2	2	2	0	0	2	0	2	2	2	2	2	2	2	2	2	2	2	2
chr11:99779001	chr11	99,779,001	99,780,000	1	1	2	2	2	2	2	2	2	2	2	2	2	2	2	2	2	2	2	2	2
chr11:99780001	chr11	99,780,001	99,781,000	1	1	2	2	2	2	2	2	2	2	2	2	2	2	2	2	2	2	2	2	2
chr11:99784001	chr11	99,784,001	99,785,000	1	1	2	2	2	2	2	2	2	2	2	2	2	2	2	2	2	2	2	2	2
chr11:99785001	chr11	99,785,001	99,786,000	1	1	2	2	2	2	2	2	2	2	2	2	2	2	2	2	2	2	2	2	2
chr12:10424001	chr12	10,424,001	10,425,000	1	1	2	2	2	2	3	2	2	2	2	2	2	2	2	2	2	2	2	2	2
chr12:10425001	chr12	10,425,001	10,426,000	1	1	2	2	2	2	3	2	2	2	2	2	2	2	2	2	2	2	2	2	2
chr12:10426001	chr12	10,426,001	10,427,000	1	1	2	2	2	2	3	2	2	2	2	2	2	2	2	2	2	2	2	2	2
chr12:10427001	chr12	10,427,001	10,428,000	1	1	2	2	2	2	3	2	2	2	2	2	2	2	2	2	2	2	2	2	2
chr12:10428001	chr12	10,428,001	10,429,000	1	1	2	2	2	2	3	2	2	2	2	2	2	2	2	2	2	2	2	2	2
chr12:104979001	chr12	104,979,001	104,980,000	1	1	2	2	2	2	2	2	2	2	2	2	2	2	2	2	2	2	2	2	2
chr12:132232001	chr12	132,232,001	132,233,000	1	0	2	2	2	1	2	2	1	2	2	2	2	2	2	2	2	2	2	2	2
chr14:106701001	chr14	106,701,001	106,702,000	1	1	2	2	2	2	2	2	2	2	2	2	2	2	2	2	2	2	2	2	2
chr14:106702001	chr14	106,702,001	106,703,000	1	1	2	2	3	2	2	2	2	2	2	2	2	2	2	2	2	2	2	2	2
chr15:101102001	chr15	101,102,001	101,103,000	2	2	2	2	3	3	3	2	2	2	2	2	2	2	2	2	2	2	2	2	2
chr15:101103001	chr15	101,103,001	101,104,000	2	2	2	2	3	3	3	2	2	2	2	2	2	2	2	2	2	2	2	2	2
chr15:101104001	chr15	101,104,001	101,105,000	2	2	2	2	3	3	3	2	2	2	2	2	2	2	2	2	2	2	2	2	2
chr15:101105001	chr15	101,105,001	101,106,000	2	2	2	2	3	3	3	2	2	2	2	2	2	2	2	2	2	2	2	2	2
chr15:101106001	chr15	101,106,001	101,107,000	2	2	2	2	3	3	3	2	2	2	2	2	2	2	2	2	2	2	2	2	2
chr15:101107001	chr15	101,107,001	101,108,000	2	2	2	2	3	3	3	2	2	2	2	2	2	2	2	2	2	2	2	2	2
chr15:101108001	chr15	101,108,001	101,109,000	2	2	2	2	3	3	3	2	2	2	2	2	2	2	2	2	2	2	2	2	2
chr15:101109001	chr15	101,109,001	101,110,000	2	2	2	2	3	3	3	2	2	2	2	2	2	2	2	2	2	2	2	2	2
chr15:101110001	chr15	101,110,001	101,111,000	2	2	2	2	3	3	3	2	2	2	2	2	2	2	2	2	2	2	2	2	2
chr15:101111001	chr15	101,111,001	101,112,000	2	2	2	2	3	3	3	2	2	2	2	2	2	2	2	2	2	2	2	2	2
chr16:168001	chr16	168,001	169,000	2	2	2	2	3	3	3	2	2	2	2	2	2	2	2	2	2	2	2	2	2
chr16:210001	chr16	210,001	211,000	2	2	2	2	3	3	3	3	3	2	2	2	2	2	2	2	2	2	2	2	2
chr16:211001	chr16	211,001	212,000	2	2	2	2	3	3	3	2	3	2	2	2	2	2	2	2	2	2	2	2	2
chr16:212001	chr16	212,001	213,000	2	2	2	2	3	3	3	2	3	2	2	2	2	2	2	2	2	2	2	2	2
chr16:213001	chr16	213,001	214,000	2	2	2	2	3	3	3	2	3	2	2	2	2	2	2	2	2	2	2	2	2
chr16:214001	chr16	214,001	215,000	2	2	2	2	3	3	3	2	3	2	2	2	2	2	2	2	2	2	2	2	2
chr16:215001	chr16	215,001	216,000	2	2	2	2	3	3	3	2	3	2	2	2	2	2	2	2	2	2	2	2	2
chr16:216001	chr16	216,001	217,000	2	2	2	2	3	2	3	2	3	2	2	2	2	2	2	2	2	2	2	2	2
chr16:18377001	chr16	18,377,001	18,378,000	3	3	2	2	2	2	2	2	2	2	2	2	2	2	2	2	2	2	2	2	2
chr16:18378001	chr16	18,378,001	18,379,000	3	3	2	2	2	2	2	2	2	2	2	2	2	2	2	2	2	2	2	2	2
chr16:18379001	chr16	18,379,001	18,380,000	3	3	2	2	2	2	2	2	2	2	2	2	2	2	2	2	2	2	2	2	2
chr16:35981001	chr16	35,981,001	35,982,000	3	3	2	2	2	2	2	2	2	2	2	2	2	2	2	2	2	2	2	2	2
chr16:35982001	chr16	35,982,001	35,983,000	3	3	2	2	2	2	2	2	2	2	2	2	2	2	2	2	2	2	2	2	2
chr16:35983001	chr16	35,983,001	35,984,000	3	3	2	3	2	2	2	2	2	2	2	2	2	2	2	2	2	2	2	2	2
chr16:35984001	chr16	35,984,001	35,985,000	3	3	2	3	2	2	2	2	2	2	2	2	2	2	2	2	2	2	2	2	2
chr16:35985001	chr16	35,985,001	35,986,000	3	3	2	3	2	2	2	2	2	2	2	2	2	2	2	2	2	2	2	2	2

Region	CHR	start	end	HD2	HD3	HD1	BN1	BN2	BN3	BN4	BN5	BN6	LC1	LC2	LC3	LC4	LC5	LC6	LC7	LC8	LC9	LC10	LC10*	LC11
chr17:129001	chr17	129,001	130,000	2	2	2	3	3	3	2	2	2	2	2	2	2	2	2	2	2	2	2	2	2
chr17:318001	chr17	318,001	319,000	2	2	2	2	2	3	3	2	3	2	2	2	2	2	2	2	2	2	2	2	2
chr17:319001	chr17	319,001	320,000	2	2	2	2	2	3	3	2	3	2	2	2	2	2	2	2	2	2	2	2	2
chr17:320001	chr17	320,001	321,000	2	2	2	2	2	3	3	2	3	2	2	2	2	2	2	2	2	2	2	2	2
chr17:18843001	chr17	18,843,001	18,844,000	2	2	2	2	3	3	2	3	2	2	2	2	2	2	2	2	2	2	2	2	2
chr17:64910001	chr17	64,910,001	64,911,000	3	3	2	3	2	2	2	2	2	2	2	2	2	2	2	2	2	2	2	2	2
chr17:64911001	chr17	64,911,001	64,912,000	3	3	2	3	2	2	2	2	2	2	2	2	2	2	2	2	2	2	2	2	2
chr17:64912001	chr17	64,912,001	64,913,000	3	3	2	3	2	2	2	2	2	2	2	2	2	2	2	2	2	2	2	2	2
chr18:14273001	chr18	14,273,001	14,274,000	3	3	2	2	2	2	2	2	2	2	2	2	2	2	2	2	2	2	2	2	2
chr18:14274001	chr18	14,274,001	14,275,000	3	3	2	2	2	2	2	2	2	2	2	2	2	2	2	2	2	2	2	2	2
chr18:14275001	chr18	14,275,001	14,276,000	3	3	2	2	2	2	2	2	2	2	2	2	2	2	2	2	2	2	2	2	2
chr18:14296001	chr18	14,296,001	14,297,000	3	3	2	2	2	2	2	2	2	2	2	2	2	2	2	2	2	2	2	2	2
chr18:29368001	chr18	29,368,001	29,369,000	2	2	2	2	2	1	1	1	2	2	2	2	2	2	2	2	2	2	2	2	2
chr18:29371001	chr18	29,371,001	29,372,000	2	2	2	2	2	1	1	1	2	2	2	2	2	2	2	2	2	2	2	2	2
chr18:29372001	chr18	29,372,001	29,373,000	2	2	2	2	2	1	1	1	2	2	2	2	2	2	2	2	2	2	2	2	2
chr18:29373001	chr18	29,373,001	29,374,000	2	2	2	2	2	1	1	1	2	2	2	2	2	2	2	2	2	2	2	2	2
chr18:29374001	chr18	29,374,001	29,375,000	2	2	2	2	2	1	1	1	2	2	2	2	2	2	2	2	2	2	2	2	2
chr18:29375001	chr18	29,375,001	29,376,000	2	2	2	2	2	1	1	1	2	2	2	2	2	2	2	2	2	2	2	2	2
chr18:29376001	chr18	29,376,001	29,377,000	2	2	2	2	2	1	1	1	2	2	2	2	2	2	2	2	2	2	2	2	2
chr18:29688001	chr18	29,688,001	29,689,000	2	3	2	2	2	1	1	1	2	2	2	2	2	2	2	2	2	2	2	2	2
chr18:29689001	chr18	29,689,001	29,690,000	2	3	2	2	2	1	1	1	2	2	2	2	2	2	2	2	2	2	2	2	2
chr18:29690001	chr18	29,690,001	29,691,000	2	3	2	2	2	1	1	1	2	2	2	2	2	2	2	2	2	2	2	2	2
chr18:30146001	chr18	30,146,001	30,147,000	3	3	2	2	2	2	1	2	2	2	2	2	2	2	2	2	2	2	2	2	2
chr18:30147001	chr18	30,147,001	30,148,000	3	3	2	2	2	2	1	2	2	2	2	2	2	2	2	2	2	2	2	2	2
chr18:30148001	chr18	30,148,001	30,149,000	3	3	2	2	2	2	1	2	2	2	2	2	2	2	2	2	2	2	2	2	2
chr18:30149001	chr18	30,149,001	30,150,000	3	3	2	2	2	2	1	2	2	2	2	2	2	2	2	2	2	2	2	2	2
chr18:30150001	chr18	30,150,001	30,151,000	3	3	2	2	2	2	1	2	2	2	2	2	2	2	2	2	2	2	2	2	2
chr19:35372001	chr19	35,372,001	35,373,000	1	1	2	1	2	2	2	2	2	2	2	2	2	2	2	2	2	2	2	2	2
chr21:5108001	chr21	5,108,001	5,109,000	2	2	2	2	2	2	1	1	1	2	2	2	2	2	2	2	2	2	2	2	2
chr21:5109001	chr21	5,109,001	5,110,000	2	2	2	2	2	2	1	1	1	2	2	2	2	2	2	2	2	2	2	2	2
chr21:5110001	chr21	5,110,001	5,111,000	2	2	2	2	2	2	1	1	1	2	2	2	2	2	2	2	2	2	2	2	2
chr21:5111001	chr21	5,111,001	5,112,000	2	2	2	2	2	2	1	1	1	2	2	2	2	2	2	2	2	2	2	2	2
chr21:5112001	chr21	5,112,001	5,113,000	2	2	2	2	2	2	1	1	1	2	2	2	2	2	2	2	2	2	2	2	2
chr21:5113001	chr21	5,113,001	5,114,000	2	2	2	2	2	2	1	1	1	2	2	2	2	2	2	2	2	2	2	2	2
chr21:5114001	chr21	5,114,001	5,115,000	2	2	2	2	2	2	1	1	1	2	2	2	2	2	2	2	2	2	2	2	2
chr21:5115001	chr21	5,115,001	5,116,000	2	2	2	2	2	2	1	1	1	2	2	2	2	2	2	2	2	2	2	2	2
chr21:5116001	chr21	5,116,001	5,117,000	2	2	2	2	2	2	1	1	1	2	2	2	2	2	2	2	2	2	2	2	2
chr21:5117001	chr21	5,117,001	5,118,000	2	2	2	2	2	2	1	1	1	2	2	2	2	2	2	2	2	2	2	2	2
chr21:5118001	chr21	5,118,001	5,119,000	2	2	2	2	2	2	1	1	1	2	2	2	2	2	2	2	2	2	2	2	2
chr21:5148001	chr21	5,148,001	5,149,000	2	2	2	2	2	2	1	1	1	2	2	2	2	2	2	2	2	2	2	2	2
chr21:44158001	chr21	44,158,001	44,159,000	2	2	2	5	3	2	4	4	2	2	2	2	2	2	2	2	2	2	2	2	2
chr21:44190001	chr21	44,190,001	44,191,000	2	2	2	1	2	2	2	1	1	2	2	2	2	2	2	2	2	2	2	2	2
chr22:10563001	chr22	10,563,001	10,564,000	3	3	2	2	2	2	2	2	2	2	2	2	2	2	2	2	2	2	2	2	2
chr22:10564001	chr22	10,564,001	10,565,000	3	3	2	2	2	2	2	2	2	2	2	2	2	2	2	2	2	2	2	2	2
chr22:10565001	chr22	10,565,001	10,566,000	3	3	2	2	2	2	2	2	2	2	2	2	2	2	2	2	2	2	2	2	2
chr22:10566001	chr22	10,566,001	10,567,000	3	3	2	2	2	2	2	2	2	2	2	2	2	2	2	2	2	2	2	2	2
chr22:10690001	chr22	10,690,001	10,691,000	3	3	2	3	2	2	2	2	2	2	2	2	2	2	2	2	2	2	2	2	2
chr22:10691001	chr22	10,691,001	10,692,000	3	3	2	3	2	2	2	2	2	2	2	2	2	2	2	2	2	2	2	2	2
chr22:10692001	chr22	10,692,001	10,693,000	3	3	2	3	2	2	2	2	2	2	2	2	2	2	2	2	2	2	2	2	2
chr22:10693001	chr22	10,693,001	10,694,000	3	3	2	3	2	2	2	2	2	2	2	2	2	2	2	2	2	2	2	2	2
chr22:10694001	chr22	10,694,001	10,695,000	3	3	2	3	2	2	2	2	2	2	2	2	2	2	2	2	2	2	2	2	2
chr22:10695001	chr22	10,695,001	10,696,000	3	3	2	3	2	2	2	2	2	2	2	2	2	2	2	2	2	2	2	2	2
chr22:38898001	chr22	38,898,001	38,899,000	2	2	2	2	1	0	1	2	0	2	2	2	2	2	2	2	2	2	2	2	2
chr22:38899001	chr22	38,899,001	38,900,000	2	2	2	2	1	0	1	2	0	2	2	2	2	2	2	2	2	2	2	2	2
chr22:38900001	chr22	38,900,001	38,901,000	2	2	2	2	1	0	1	2	0	2	2	2	2	2	2	2	2	2	2	2	2
chr22:38901001	chr22	38,901,001	38,902,000	2	2	2	2	1	0	1	2	0	2	2	2	2	2	2	2	2	2	2	2	2
chr22:38902001	chr22	38,902,001	38,903,000	2	2	2	2	1	1	1	2	1	2	2	2	2	2	2	2	2	2	2	2	2
chr22:44114001	chr22	44,114,001	44,115,000	2	2	2	1	1	2	1	1	1	2	2	2	2	1	2	2	2	2	2	3	2
chr1:108387001	chr1	108,387,001	108,388,000	2	1	1	2	2	2	2	1	2	2	2	2	2	2	2	2	2	2	2	2	2
chr1:108388001	chr1	108,388,001	108,389,000	2	1	1	2	2	2	2	1	2	2	2	2	2	2	2	2	2	2	2	2	2
chr1:108389001	chr1	108,389,001	108,390,000	2	1	1	2	2	2	2	1	2	2	2	2	2	2	2	2	2	2	2	2	2
chr1:108390001	chr1	108,390,001	108,391,000	2	1	1	2	2	2	2	1	2	2	2	2	2	2	2	2	2	2	2	2	2
chr1:108391001	chr1	108,391,001	108,392,000	2	1	1	2	2	2	2	1	2	2	2	2	2	2	2	2	2	2	2	2	2
chr1:108392001	chr1	108,392,001	108,393,000	2	1	1	2	2	2	2	1	2	2	2	2	2	2	2	2	2	2	2	2	2
chr1:108393001	chr1	108,393,001	108,394,000	2	1	1	2	2	2	2	1	2	2	2	2	2	2	2	2	2	2	2	2	2
chr1:108406001	chr1	108,406,001	108,407,000	2	1	1	2	2	2	2	1	2	2	2	2	2	2	2	2	2	2	2	2	2
chr1:108407001	chr1	108,407,001	108,408,000	2	1	1	2	2	2	2	1	2	2	2	2	2	2	2	2	2	2	2	2	2
chr1:108408001	chr1	108,408,001	108,409,000	2	1	1	2	2	2	2	1	2	2	2	2	2	2	2	2	2	2	2	2	2
chr1:108409001	chr1	108,409,001	108,410,000	2	1	1	2	2	2	2	1	2	2	2	2	2	2	2	2	2	2	2	2	2
chr4:31462001	chr4	31,462,001	31,463,000	1	2	1	2	1	1	2	1	2	2	2	2	2	2	2	2	2	2	2	2	2
chr4:31463001	chr4	31,463,001	31,464,000	1	2	1	2	1	1	2	1	2	2	2	2	2	2	2	2	2	2	2	2	2
chr4:31464001	chr4	31,464,001	31,465,000	1	2	1	2	1	1	2	1	2	2	2	2	2	2	2	2	2	2	2	2	2
chr4:31465001	chr4	31,465,001	31,466,000	1	2	1	2	1	1	2	1	2	2	2	2	2	2	2	2	2	2	2	2	2
chr4:31466001	chr4	31,466,001	31,467,000	1	2	1	2	1	1	1	1	2	2	2	2	2	2	2	2	2	2	2	2	2
chr5:177809001	chr5	177,809,001	177,810,000	2	1	1	2	2	2	2	2	1	2	2	2	2	2	2	2	2	2	2	2	2
chr5:177810001	chr5	177,810,001	177,811,000	2	1	1	2	2	2	2	2	2	2	2	2	2	2	2	2	2	2	2	2	2
chr8:7499001	chr8	7,499,001	7,500,000	2	1	1	2	2	3	2	2	2	2	2	2	2	2	2	2	2	2	2	2	2
chr8:7500001	chr8	7,500,001	7,501,000	2	1	1	2	2	3	2	2	2	2	2	2	2	2	2	2	2	2	2	2	2
chr9:61077001	chr9	61,077,001	61,078,000	2	1	1	2	2	2	1	1	1	2	2	2	2	2	2	2	2	2	2	2	2
chr9:61431001	chr9	61,431,001	61,432,000	2	1	1	2	2	2	2	2	2	2	2	2	2	2	2	2	2	2	2	2	2
chr9:64629001	chr9	64,629,001	64,630,000	1	2	1	2	2	3	2	2	2	2	2	2	2	2	2	2	2	2	2	2	2
chr9:64630001	chr9	64,630,001	64,631,000	1	2	1	2	2	3	2	2	2	2	2	2	2	2	2	2	2	2	2	2	2
chr10:98928001	chr10	98,928,001	98,929,000	1	2	1	2	2	2	2	2	2	2	2	2	2	2	2	2	2	2	2	2	2
chr10:98929001	chr10	98,929,001	98,930,000	1	2	1	2	2	2	2	2	2	2	2	2	2	2	2	2	2	2	2	2	2
chr10:98930001	chr10	98,930,001	98,931,000	1	2	1	2	2	2	2	2	2	2	2	2	2	2	2	2	2	2	2	2	2
chr10:98931001	chr10	98,931,001	98,932,000	1	2	1	2	2	2	2	2	2	2	2	2	2	2	2	2	2	2	2	2	2
chr10:98932001	chr10	98,932,001	98,933,000	1	2	1	2	2	2	2	2	2	2	2	2	2	2	2	2	2	2	2	2	2
chr10:98933001	chr10	98,933,001	98,934,000	1	2	1	2	2	2	2	2	2	2	2	2	2	2	2	2	2	2	2	2	2
chr10:98934001	chr10	98,934,001	98,935,000	1	2	1	2	2	2	2	2	2	2	2	2	2	2	2	2	2	2	2	2	2
chr10:98935001	chr10	98,935,001	98,936,000	1	2	1	2	2	2	2	2	2	2	2	2	2	2	2	2	2	2	2	2	2
chr10:98936001	chr10	98,936,001	98,937,000	1	2	1	2	2	2	2	2	2	2	2	2	2	2	2	2	2	2	2	2	2
chr10:98937001	chr10	98,937,001	98,938,000	1	2	1	2	2	2	2	2	2	2	2	2	2	2	2	2	2	2	2	2	2
chr10:98938001	chr10	98,938,001	98,939,000	1	2	1	2	2	2	2	2	2	2	2	2	2	2	2	2	2	2	2	2	2
chr10:98939001	chr10	98,939,001	98,940,000	1	2	1	2	2	2	2	2	2	2	2	2	2	2	2	2	2	2	2	2	2
chr11:3253001	chr11	3,253,001	3,254,000	1	1	1	2	2	2	1	2	2	2	2	2	2	2	2	2	2	2	2	2	2
chr11:3254001	chr11	3,254,001	3,255,000	1	1	1	2	2	2	1	2	2	2	2	2	2	2	2	2	2	2	2	2	2
chr12:70484001	chr12	70,484,001	70,485,000	1	2	1	2	2	2	2	2	2	2	2	2	2	2	2	2	2	2	2	2	2
chr12:132234001	chr12	132,234,001	132,235,000	1	1	1	2	2	1	2	2	1	2	2	2	2	2	2	2	2	2	2	2	2
chr13:111683001	chr13	111,683,001	111,684,000	2	1	1	1	2	2	2	2	2	2	2	2	2	2	2	2	2	2	2	2	2
chr13:111684001	chr13	111,684,001	111,685,000	2	1	1	1	2	2	2	2	2	2	2	2	2	2	2	2	2	2	2	2	2
chr13:111695001	chr13	111,695,001	111,696,000	1	1	1	2	1	2	2	2	2	2	2	2	2	2	2	2	2	2	2	2	2
chr13:111696001	chr13	111,696,001	111,697,000	1	1	1	2	1	2	2	2	2	2	2	2	2	2	2	2	2	2	2	2	2
chr13:111697001	chr13	111,697,001	111,698,000	1	1	1	2	1	2	2	2	2	2	2	2	2	2	2	2	2	2	2	2	2
chr22:12790001	chr22	12,790,001	12,791,000	1	1	1	2	2	3	2	2	2	2	2	2	2	2	2	2	2	2	2	2	2
chr22:12791001	chr22	12,791,001	12,792,000	1	1	1	2	2	3	2	2	2	2	2	2	2	2	2	2	2	2	2	2	2
chr22:12792001	chr22	12,792,001	12,793,000	1	1	1	2	2	3	2	2	2	2	2	2	2	2	2	2	2	2	2	2	2
chr22:12793001	chr22	12,793,001	12,794,000	1	1	1	2	2	3	2	2	2	2	2	2	2	2	2	2	2	2	2	2	2
chr22:12794001	chr22	12,794,001	12,795,000	1	2	1	2	2	3	2	2	2	2	2	2	2	2	2	2	2	2	2	2	2
chr22:12795001	chr22	12,795,001	12,796,000	1	2	1	2	2	3	2	2	2	2	2	2	2	2	2	2	2	2	2	2	2
chr22:12796001	chr22	12,796,001	12,797,000	1	2	1	2	2	3	2	2	2	2	2	2	2	2	2	2	2	2	2	2	2
chr3:149551001	chr3	149,551,001	149,552,000	2	0	0	2	2	2	2	2	2	2	2	2	2	2	2	2	2	2	2	2	2
chr4:31439001	chr4	31,439,001	31,440,000	1	2	0	2	2	1	1	2	2	2	2	2	2	2	2	2	2	2	2	2	2
chr22:12788001	chr22	12,788,001	12,789,000	1	1	0	2	2	3	2	2	2	2	2	2	2	2	2	2	2	2	2	2	2

Region	CHR	start	end	HD2	HD3	HD1	BN1	BN2	BN3	BN4	BN5	BN6	LC1	LC2	LC3	LC4	LC5	LC6	LC7	LC8	LC9	LC10	LC10*	LC11
chr22:12789001	chr22	12,789,001	12,790,000	1	1	0	2	2	3	2	2	2	2	2	2	2	2	2	2	2	2	2	2	2
chr9:41350001	chr9	41,350,001	41,351,000	2	3	4	2	2	2	1	1	2	2	2	2	2	2	1	2	2	2	2	2	2
chr9:40014001	chr9	40,014,001	40,015,000	3	3	3	3	2	3	2	2	2	2	2	2	2	2	1	2	2	2	2	2	2
chr9:40015001	chr9	40,015,001	40,016,000	3	3	3	3	2	3	2	2	2	2	2	2	2	2	1	2	2	2	2	2	2
chr9:40016001	chr9	40,016,001	40,017,000	3	3	3	3	2	3	2	2	2	2	2	2	2	2	1	2	2	2	2	2	2
chr9:40017001	chr9	40,017,001	40,018,000	3	3	3	3	2	3	2	2	2	2	2	2	2	2	1	2	2	2	2	2	2
chr9:40018001	chr9	40,018,001	40,019,000	3	3	3	3	2	3	2	2	2	2	2	2	2	2	1	2	2	2	2	2	2
chr9:40019001	chr9	40,019,001	40,020,000	3	3	3	3	2	3	2	2	2	2	2	2	2	2	1	2	2	2	2	2	2
chr9:40020001	chr9	40,020,001	40,021,000	3	3	3	3	2	3	2	2	2	2	2	2	2	2	1	2	2	2	2	2	2
chr9:40021001	chr9	40,021,001	40,022,000	3	3	3	3	2	3	2	2	2	2	2	2	2	2	1	2	2	2	2	2	2
chr9:40022001	chr9	40,022,001	40,023,000	3	3	3	3	2	3	2	2	2	2	2	2	2	2	1	2	2	2	2	2	2
chr9:40023001	chr9	40,023,001	40,024,000	3	3	3	3	2	3	2	2	2	2	2	2	2	2	1	2	2	2	2	2	2
chr9:40024001	chr9	40,024,001	40,025,000	3	3	3	3	2	3	2	2	2	2	2	2	2	2	1	2	2	2	2	2	2
chr9:40038001	chr9	40,038,001	40,039,000	3	2	3	3	3	3	2	2	2	2	2	2	2	2	1	2	2	2	2	2	2
chr9:40039001	chr9	40,039,001	40,040,000	3	2	3	3	3	3	2	2	2	2	2	2	2	2	1	2	2	2	2	2	2
chr9:40040001	chr9	40,040,001	40,041,000	3	2	3	3	3	3	2	2	2	2	2	2	2	2	1	2	2	2	2	2	2
chr9:40041001	chr9	40,041,001	40,042,000	3	2	3	3	3	3	2	2	2	2	2	2	2	2	1	2	2	2	2	2	2
chr9:40042001	chr9	40,042,001	40,043,000	3	2	3	3	3	3	2	2	2	2	2	2	2	2	1	2	2	2	2	2	2
chr9:40043001	chr9	40,043,001	40,044,000	3	2	3	3	3	3	2	2	2	2	2	2	2	2	1	2	2	2	2	2	2
chr9:40044001	chr9	40,044,001	40,045,000	3	2	3	3	3	3	2	2	2	2	2	2	2	2	1	2	2	2	2	2	2
chr9:40045001	chr9	40,045,001	40,046,000	3	2	3	3	3	3	2	2	2	2	2	2	2	2	1	2	2	2	2	2	2
chr9:40046001	chr9	40,046,001	40,047,000	3	2	3	3	3	3	2	2	2	2	2	2	2	2	1	2	2	2	2	2	2
chr9:40047001	chr9	40,047,001	40,048,000	3	2	3	3	3	3	2	2	2	2	2	2	2	2	1	2	2	2	2	2	2
chr9:40073001	chr9	40,073,001	40,074,000	3	2	3	3	3	3	2	2	2	2	2	2	2	2	1	2	2	2	2	2	2
chr9:40074001	chr9	40,074,001	40,075,000	3	2	3	3	3	3	2	2	2	2	2	2	2	2	1	2	2	2	2	2	2
chr9:40075001	chr9	40,075,001	40,076,000	3	2	3	3	3	3	2	2	2	2	2	2	2	2	1	2	2	2	2	2	2
chr9:40097001	chr9	40,097,001	40,098,000	2	3	3	3	3	3	2	2	2	2	2	2	2	2	1	2	2	2	2	2	2
chr9:40098001	chr9	40,098,001	40,099,000	2	3	3	3	3	3	2	2	2	2	2	2	2	2	1	2	2	2	2	2	2
chr9:40099001	chr9	40,099,001	40,100,000	2	3	3	3	3	3	2	2	2	2	2	2	2	2	1	2	2	2	2	2	2
chr9:40100001	chr9	40,100,001	40,101,000	2	3	3	3	3	3	2	2	2	2	2	2	2	2	1	2	2	2	2	2	2
chr9:40101001	chr9	40,101,001	40,102,000	2	3	3	3	3	3	2	2	2	2	2	2	2	2	1	2	2	2	2	2	2
chr9:40102001	chr9	40,102,001	40,103,000	2	3	3	3	3	3	2	2	2	2	2	2	2	2	1	2	2	2	2	2	2
chr9:40126001	chr9	40,126,001	40,127,000	2	2	3	4	3	3	2	3	2	2	2	2	1	2	2	2	2	2	2	2	2
chr9:40250001	chr9	40,250,001	40,251,000	3	2	3	2	2	2	2	2	2	2	2	2	2	2	2	2	2	1	2	2	2
chr9:41344001	chr9	41,344,001	41,345,000	2	3	3	2	2	2	1	1	2	2	2	2	2	2	1	2	2	2	2	2	2
chr9:41345001	chr9	41,345,001	41,346,000	2	3	3	2	2	2	1	1	2	2	2	2	2	2	1	2	2	2	2	2	2
chr9:41346001	chr9	41,346,001	41,347,000	2	3	3	2	2	2	1	1	2	2	2	2	2	2	1	2	2	2	2	2	2
chr9:41347001	chr9	41,347,001	41,348,000	2	3	3	2	2	2	1	1	2	2	2	2	2	2	1	2	2	2	2	2	2
chr9:41348001	chr9	41,348,001	41,349,000	2	3	3	2	2	2	1	1	2	2	2	2	2	2	1	2	2	2	2	2	2
chr9:41349001	chr9	41,349,001	41,350,000	2	3	3	2	2	2	1	1	2	2	2	2	2	2	1	2	2	2	2	2	2
chr9:41365001	chr9	41,365,001	41,366,000	3	3	3	2	3	2	1	2	2	2	2	2	2	2	1	2	2	2	2	2	2
chr9:41371001	chr9	41,371,001	41,372,000	2	3	3	2	2	2	1	1	2	2	2	2	2	2	1	2	2	2	2	2	2
chr9:41372001	chr9	41,372,001	41,373,000	2	3	3	2	2	2	1	1	2	2	2	2	2	2	1	2	2	2	2	2	2
chr9:41373001	chr9	41,373,001	41,374,000	2	3	3	2	2	2	1	1	2	2	2	2	2	2	1	2	2	2	2	2	2
chr9:41374001	chr9	41,374,001	41,375,000	2	3	3	2	2	2	1	1	2	2	2	2	2	2	1	2	2	2	2	2	2
chr9:41375001	chr9	41,375,001	41,376,000	2	3	3	2	2	2	1	1	2	2	2	2	2	2	1	2	2	2	2	2	2
chr9:41376001	chr9	41,376,001	41,377,000	2	3	3	2	2	2	1	1	2	2	2	2	2	2	1	2	2	2	2	2	2
chr9:41377001	chr9	41,377,001	41,378,000	2	3	3	2	2	2	1	1	2	2	2	2	2	2	1	2	2	2	2	2	2
chr9:41473001	chr9	41,473,001	41,474,000	2	3	3	2	2	2	1	1	2	2	2	2	2	2	1	2	2	2	2	2	2
chr9:41474001	chr9	41,474,001	41,475,000	2	3	3	2	2	2	1	1	2	2	2	2	2	2	1	2	2	2	2	2	2
chr9:41475001	chr9	41,475,001	41,476,000	2	3	3	2	2	2	1	1	2	2	2	2	2	2	1	2	2	2	2	2	2
chr9:41476001	chr9	41,476,001	41,477,000	2	3	3	2	2	2	1	1	2	2	2	2	2	2	1	2	2	2	2	2	2
chr9:41477001	chr9	41,477,001	41,478,000	2	3	3	2	2	2	1	1	2	2	2	2	2	2	1	2	2	2	2	2	2
chr9:41478001	chr9	41,478,001	41,479,000	2	3	3	2	2	2	1	1	2	2	2	2	2	2	1	2	2	2	2	2	2
chr9:41479001	chr9	41,479,001	41,480,000	2	3	3	2	2	2	1	1	2	2	2	2	2	2	1	2	2	2	2	2	2
chr9:41480001	chr9	41,480,001	41,481,000	2	3	3	2	2	2	1	1	2	2	2	2	2	2	1	2	2	2	2	2	2
chr9:41481001	chr9	41,481,001	41,482,000	2	3	3	2	2	2	1	1	2	2	2	2	2	2	1	2	2	2	2	2	2
chr9:42832001	chr9	42,832,001	42,833,000	3	2	3	2	2	2	2	2	2	2	2	2	1	2	2	2	2	2	2	2	2
chr9:42833001	chr9	42,833,001	42,834,000	3	2	3	2	2	2	2	2	2	2	2	2	1	2	2	2	2	2	2	2	2
chr9:42834001	chr9	42,834,001	42,835,000	3	2	3	2	2	2	2	2	2	2	2	2	1	2	2	2	2	2	2	2	2
chr9:43290001	chr9	43,290,001	43,291,000	3	2	3	2	2	2	2	2	2	2	2	2	1	2	2	2	2	2	2	2	2
chr9:43357001	chr9	43,357,001	43,358,000	3	3	3	2	2	2	2	2	2	2	2	2	1	2	2	2	2	2	2	2	2
chr9:43358001	chr9	43,358,001	43,359,000	3	3	3	2	2	2	2	2	2	2	2	2	1	2	2	2	2	2	2	2	2
chr9:67623001	chr9	67,623,001	67,624,000	4	3	3	2	3	2	2	2	2	2	3	1	1	2	2	3	2	2	2	1	2
chr9:67624001	chr9	67,624,001	67,625,000	4	3	3	2	3	3	2	2	2	2	3	1	1	2	2	3	2	2	2	1	2
chr5:69871001	chr5	69,871,001	69,872,000	2	2	2	2	3	3	3	2	3	2	2	2	2	2	2	1	2	2	2	2	2
chr5:69872001	chr5	69,872,001	69,873,000	2	2	2	2	3	3	3	2	3	2	2	2	2	2	2	1	2	2	2	2	2
chr5:70717001	chr5	70,717,001	70,718,000	2	2	2	3	3	3	3	2	3	2	3	2	1	2	2	1	2	2	2	2	2
chr5:70724001	chr5	70,724,001	70,725,000	2	2	2	2	3	3	3	2	3	2	2	2	2	2	2	1	2	2	2	2	2
chr5:70725001	chr5	70,725,001	70,726,000	2	2	2	2	3	3	3	2	3	2	2	2	2	2	2	1	2	2	2	2	2
chr5:71253001	chr5	71,253,001	71,254,000	2	2	2	2	3	3	3	2	3	2	2	2	2	2	2	1	2	2	2	2	2
chr5:71254001	chr5	71,254,001	71,255,000	2	2	2	2	3	3	3	2	3	2	2	2	2	2	2	1	2	2	2	2	2
chr5:71255001	chr5	71,255,001	71,256,000	2	2	2	2	3	3	3	2	3	2	2	2	2	2	2	1	2	2	2	2	2
chr5:71256001	chr5	71,256,001	71,257,000	2	2	2	2	3	3	3	2	3	2	2	2	2	2	2	1	2	2	2	2	2
chr5:71266001	chr5	71,266,001	71,267,000	2	2	2	3	3	3	3	2	3	2	3	2	2	2	2	1	2	2	2	1	2
chr5:71268001	chr5	71,268,001	71,269,000	2	2	2	2	3	3	3	2	3	2	2	2	2	2	2	1	2	2	2	2	2
chr5:71269001	chr5	71,269,001	71,270,000	2	2	2	2	3	3	3	2	3	2	2	2	2	2	2	1	2	2	2	2	2
chr5:71270001	chr5	71,270,001	71,271,000	2	2	2	2	3	3	3	2	3	2	2	2	2	2	2	1	2	2	2	2	2
chr5:71273001	chr5	71,273,001	71,274,000	2	2	2	2	3	3	3	2	3	2	2	2	2	2	2	1	2	2	2	2	2
chr5:71274001	chr5	71,274,001	71,275,000	2	2	2	2	3	3	3	2	3	2	2	2	2	2	2	1	2	2	2	2	2
chr5:71275001	chr5	71,275,001	71,276,000	2	2	2	2	3	3	3	2	3	2	2	2	2	2	2	1	2	2	2	2	2
chr5:127880001	chr5	127,880,001	127,881,000	5	5	2	2	2	2	2	2	2	2	2	2	2	2	2	2	2	2	2	1	2
chr7:4589001	chr7	4,589,001	4,590,000	3	3	2	2	2	2	2	2	2	2	2	2	2	2	2	2	1	2	2	2	2
chr7:4590001	chr7	4,590,001	4,591,000	3	3	2	2	2	2	2	2	2	2	2	2	2	2	2	2	1	2	2	2	2
chr7:4591001	chr7	4,591,001	4,592,000	3	3	2	2	2	2	2	2	2	2	2	2	2	2	2	2	1	2	2	2	2
chr7:4592001	chr7	4,592,001	4,593,000	3	3	2	2	2	2	2	2	2	2	2	2	2	2	2	2	1	2	2	2	2
chr7:4595001	chr7	4,595,001	4,596,000	3	3	2	2	2	2	2	2	2	2	2	2	2	2	2	1	2	2	2	2	2
chr7:4596001	chr7	4,596,001	4,597,000	3	3	2	2	2	2	2	2	2	2	2	2	2	2	2	1	2	2	2	2	2
chr7:4597001	chr7	4,597,001	4,598,000	3	3	2	2	2	2	2	2	2	2	2	2	2	2	2	1	2	2	2	2	2
chr7:4598001	chr7	4,598,001	4,599,000	3	3	2	2	2	2	2	2	2	2	2	2	2	2	2	1	2	2	2	2	2
chr7:4599001	chr7	4,599,001	4,600,000	3	3	2	2	2	2	2	2	2	2	2	2	2	2	2	1	2	2	2	2	2
chr7:100730001	chr7	100,730,001	100,731,000	2	2	2	2	1	1	1	2	1	2	2	1	2	2	2	2	2	2	2	2	2
chr7:100731001	chr7	100,731,001	100,732,000	2	2	2	2	1	1	1	2	1	2	2	1	2	2	2	2	2	2	2	2	2
chr7:100732001	chr7	100,732,001	100,733,000	2	2	2	2	1	1	1	2	1	2	2	1	2	2	2	2	2	2	2	2	2
chr7:100733001	chr7	100,733,001	100,734,000	2	2	2	2	1	1	1	2	1	2	2	1	2	2	2	2	2	2	2	2	2
chr7:100734001	chr7	100,734,001	100,735,000	2	2	2	2	1	1	1	2	1	2	2	1	2	2	2	2	2	2	2	2	2
chr7:100735001	chr7	100,735,001	100,736,000	2	2	2	2	1	1	1	2	1	2	2	1	2	2	2	2	2	2	2	2	2
chr7:100736001	chr7	100,736,001	100,737,000	2	2	2	2	1	1	1	2	1	2	2	1	2	2	2	2	2	2	2	2	2
chr7:100737001	chr7	100,737,001	100,738,000	2	2	2	2	1	1	1	2	1	2	2	1	2	2	2	2	2	2	2	2	2
chr7:100738001	chr7	100,738,001	100,739,000	2	2	2	2	1	1	1	2	1	2	2	1	2	2	2	2	2	2	2	2	2
chr7:100739001	chr7	100,739,001	100,740,000	2	2	2	2	1	1	1	2	1	2	2	1	2	2	2	2	2	2	2	2	2
chr8:7528001	chr8	7,528,001	7,529,000	2	1	2	3	2	3	3	2	3	2	2	2	2	2	2	2	1	2	2	2	2
chr8:7529001	chr8	7,529,001	7,530,000	2	1	2	3	2	3	3	2	3	2	2	2	2	2	2	2	1	2	2	2	2
chr8:7530001	chr8	7,530,001	7,531,000	2	1	2	3	2	3	3	2	3	2	2	2	2	2	2	2	1	2	2	2	2
chr8:7531001	chr8	7,531,001	7,532,000	2	1	2	3	2	3	3	2	3	2	2	2	2	2	2	2	1	2	2	2	2
chr8:7532001	chr8	7,532,001	7,533,000	2	1	2	3	2	3	3	2	3	2	2	2	2	2	2	2	1	2	2	2	2
chr8:12352001	chr8	12,352,001	12,353,000	1	1	2	3	2	3	4	2	2	2	2	2	2	2	2	2	1	2	2	2	2
chr8:12353001	chr8	12,353,001	12,354,000	1	1	2	3	2	3	4	2	2	2	2	2	2	2	2	2	1	2	2	2	2
chr8:12354001	chr8	12,354,001	12,355,000	1	1	2	3	2	3	4	2	2	2	2	2	2	2	2	2	1	2	2	2	2
chr8:12469001	chr8	12,469,001	12,470,000	1	1	2	2	2	3	3	2	2	2	2	2	2	2	2	2	1	2	2	2	2
chr8:12470001	chr8	12,470,001	12,471,000	1	1	2	2	2	3	3	2	2	2	2	2	2	2	2	2	1	2	2	2	2
chr9:67437001	chr9	67,437,001	67,438,000	4	3	2	2	2	2	1	2	1	2	2	2	2	2	2	2	2	2	2	1	2
chr16:18380001	chr16	18,380,001	18,381,000	3	3	2	2	2	2	2	2	2	2	2	2	2	2	2	2	2	1	2	2	2

Region	CHR	start	end	HD2	HD3	HD1	BN1	BN2	BN3	BN4	BN5	BN6	LC1	LC2	LC3	LC4	LC5	LC6	LC7	LC8	LC9	LC10	LC10*	LC11
chr18:14276001	chr18	14,276,001	14,277,000	3	3	2	2	2	2	2	2	2	2	2	2	2	2	2	2	2	2	2	1	2
chr18:14295001	chr18	14,295,001	14,296,000	3	3	2	2	2	2	2	2	2	2	2	2	2	2	2	2	2	2	2	1	2
chr18:28701001	chr18	28,701,001	28,702,000	2	2	2	2	1	1	1	1	2	2	2	2	2	2	1	2	2	2	2	2	2
chr18:29599001	chr18	29,599,001	29,600,000	2	2	2	2	2	1	1	1	1	2	2	2	2	2	1	2	2	2	2	2	2
chr18:29600001	chr18	29,600,001	29,601,000	2	2	2	2	2	1	1	1	1	2	2	2	2	2	1	2	2	2	2	2	2
chr2:308001	chr2	308,001	309,000	1	1	1	2	3	2	2	2	3	2	2	2	2	1	1	3	2	2	2	2	2
chr5:69772001	chr5	69,772,001	69,773,000	1	1	1	2	2	2	2	1	2	1	2	2	2	2	2	2	2	2	2	2	2
chr5:69773001	chr5	69,773,001	69,774,000	1	1	1	2	2	2	3	1	2	1	2	2	2	2	2	2	2	2	2	2	2
chr5:69774001	chr5	69,774,001	69,775,000	1	1	1	2	2	2	3	2	2	1	2	2	2	2	2	2	2	2	2	2	2
chr9:70730001	chr9	70,730,001	70,731,000	1	1	1	2	2	2	2	2	2	2	2	2	2	2	2	2	2	2	2	2	1
chr9:70731001	chr9	70,731,001	70,732,000	1	1	1	2	2	2	2	2	2	2	2	2	2	2	2	2	2	2	2	2	1
chr9:70732001	chr9	70,732,001	70,733,000	1	1	1	2	2	2	2	2	2	2	2	2	2	2	2	2	2	2	2	2	1
chr11:3252001	chr11	3,252,001	3,253,000	1	1	1	2	2	1	1	2	2	2	2	2	2	2	2	2	2	2	1	2	2
chr13:111692001	chr13	111,692,001	111,693,000	1	1	1	2	1	2	3	2	2	2	2	2	2	1	2	2	2	2	2	2	2
chr13:111693001	chr13	111,693,001	111,694,000	1	1	1	2	1	2	3	2	2	2	2	2	2	1	2	2	2	2	2	2	2
chr13:111694001	chr13	111,694,001	111,695,000	1	1	1	2	1	2	3	2	2	2	2	2	2	1	2	2	2	2	2	2	2
chr13:111698001	chr13	111,698,001	111,699,000	1	1	1	1	2	2	2	2	2	1	2	2	2	2	2	2	2	2	2	2	2
chr13:111699001	chr13	111,699,001	111,700,000	1	1	1	1	2	2	2	2	2	1	2	2	2	2	2	2	2	2	2	2	2
chr20:64326001	chr20	64,326,001	64,327,000	1	1	1	2	2	2	2	2	2	2	2	2	2	2	2	2	2	1	2	2	2
chr22:50343001	chr22	50,343,001	50,344,000	1	1	1	2	1	2	2	2	1	2	2	2	2	2	2	2	2	2	1	2	2
chr4:132260001	chr4	132,260,001	132,261,000	0	2	0	2	2	2	2	2	2	2	2	2	2	2	2	2	2	2	1	2	2
chr5:114989001	chr5	114,989,001	114,990,000	2	1	0	2	2	2	2	2	2	2	2	2	2	2	2	2	2	2	2	1	2
chr9:40096001	chr9	40,096,001	40,097,000	2	3	3	3	3	3	2	2	2	2	1	2	2	2	1	2	2	2	2	2	2
chr9:67597001	chr9	67,597,001	67,598,000	3	2	3	2	2	2	2	2	2	2	2	2	2	2	2	2	2	1	2	1	2
chr9:67598001	chr9	67,598,001	67,599,000	3	2	3	2	2	2	2	2	2	2	2	2	2	2	2	2	2	1	2	1	2
chr3:198101001	chr3	198,101,001	198,102,000	5	5	2	2	2	2	4	2	2	2	1	2	3	2	2	1	1	2	2	2	2
chr3:198102001	chr3	198,102,001	198,103,000	5	5	2	2	2	2	4	2	2	2	1	2	3	2	2	1	1	2	2	2	2
chr5:70723001	chr5	70,723,001	70,724,000	2	2	2	2	3	3	3	2	3	2	2	2	1	2	2	1	2	2	2	2	2
chr5:71265001	chr5	71,265,001	71,266,000	2	2	2	2	3	3	3	2	3	2	2	2	2	2	2	1	2	2	2	1	2
chr5:71271001	chr5	71,271,001	71,272,000	2	2	2	2	3	3	3	2	3	2	2	2	2	2	2	1	2	2	1	2	2
chr5:71272001	chr5	71,272,001	71,273,000	2	2	2	2	3	3	3	2	3	2	2	2	2	2	2	1	2	2	1	2	2
chr8:12350001	chr8	12,350,001	12,351,000	1	1	2	3	2	3	4	2	2	1	2	2	2	2	2	2	1	2	2	2	2
chr8:12351001	chr8	12,351,001	12,352,000	1	1	2	3	2	3	4	2	2	1	2	2	2	2	2	2	1	2	2	2	2
chr8:143552001	chr8	143,552,001	143,553,000	0	0	2	0	0	0	2	1	0	2	2	2	2	2	2	2	0	2	2	2	2
chr9:40561001	chr9	40,561,001	40,562,000	3	3	2	2	2	2	2	2	2	2	2	2	2	2	2	2	2	2	1	1	2
chr10:19708001	chr10	19,708,001	19,709,000	2	2	2	0	0	0	0	2	2	2	2	2	2	2	2	2	2	2	2	2	0
chr13:57178001	chr13	57,178,001	57,179,000	2	2	2	1	1	2	1	1	1	2	2	2	2	1	2	2	2	2	2	2	1
chr18:14277001	chr18	14,277,001	14,278,000	3	3	2	2	2	2	2	2	2	2	2	2	2	2	2	2	2	2	1	1	2
chr18:14278001	chr18	14,278,001	14,279,000	3	3	2	2	2	2	2	2	2	2	2	2	2	2	2	2	2	2	1	1	2
chr18:14279001	chr18	14,279,001	14,280,000	3	3	2	2	2	2	2	2	2	2	2	2	2	2	2	2	2	2	1	1	2
chr18:14280001	chr18	14,280,001	14,281,000	3	3	2	2	2	2	2	2	2	2	2	2	2	2	2	2	2	2	1	1	2
chr18:14281001	chr18	14,281,001	14,282,000	3	3	2	2	2	2	2	2	2	2	2	2	2	2	2	2	2	2	1	1	2
chr18:14282001	chr18	14,282,001	14,283,000	3	3	2	2	2	2	2	2	2	2	2	2	2	2	2	2	2	2	1	1	2
chr18:14283001	chr18	14,283,001	14,284,000	3	3	2	2	2	2	2	2	2	2	2	2	2	2	2	2	2	2	1	1	2
chr18:14284001	chr18	14,284,001	14,285,000	3	3	2	2	2	2	2	2	2	2	2	2	2	2	2	2	2	2	1	1	2
chr18:14285001	chr18	14,285,001	14,286,000	3	3	2	2	2	2	2	2	2	2	2	2	2	2	2	2	2	2	1	1	2
chr18:14286001	chr18	14,286,001	14,287,000	3	3	2	2	2	2	2	2	2	2	2	2	2	2	2	2	2	2	1	1	2
chr18:14287001	chr18	14,287,001	14,288,000	3	3	2	2	2	2	2	2	2	2	2	2	2	2	2	2	2	2	1	1	2
chr18:14288001	chr18	14,288,001	14,289,000	3	3	2	2	2	2	2	2	2	2	2	2	2	2	2	2	2	2	1	1	2
chr18:14289001	chr18	14,289,001	14,290,000	3	3	2	2	2	2	2	2	2	2	2	2	2	2	2	2	2	2	1	1	2
chr18:14290001	chr18	14,290,001	14,291,000	3	3	2	2	2	2	2	2	2	2	2	2	2	2	2	2	2	2	1	1	2
chr18:14291001	chr18	14,291,001	14,292,000	3	3	2	2	2	2	2	2	2	2	2	2	2	2	2	2	2	2	1	1	2
chr18:14292001	chr18	14,292,001	14,293,000	3	3	2	2	2	2	2	2	2	2	2	2	2	2	2	2	2	2	1	1	2
chr18:14293001	chr18	14,293,001	14,294,000	3	3	2	2	2	2	2	2	2	2	2	2	2	2	2	2	2	2	1	1	2
chr18:14294001	chr18	14,294,001	14,295,000	3	3	2	2	2	2	2	2	2	2	2	2	2	2	2	2	2	2	1	1	2
chr18:28699001	chr18	28,699,001	28,700,000	2	2	2	2	1	1	1	1	1	2	2	2	2	2	1	2	2	2	2	1	2
chr7:91402001	chr7	91,402,001	91,403,000	0	1	1	2	2	1	1	1	1	2	1	2	2	2	2	1	2	2	2	2	2
chr7:91412001	chr7	91,412,001	91,413,000	0	1	1	1	2	1	1	1	1	2	1	2	2	2	2	1	2	2	2	2	2
chr8:7211001	chr8	7,211,001	7,212,000	1	1	1	2	1	2	2	2	2	2	2	2	1	2	2	2	2	1	2	2	2
chr8:7212001	chr8	7,212,001	7,213,000	1	1	1	2	1	2	2	2	2	2	2	2	1	2	2	2	2	1	2	2	2
chr8:12059001	chr8	12,059,001	12,060,000	1	1	1	2	2	2	2	2	2	1	1	2	2	2	2	2	2	2	2	2	2
chr8:12060001	chr8	12,060,001	12,061,000	1	1	1	2	2	2	2	2	1	1	1	2	2	2	2	2	2	2	2	2	2
chr9:63157001	chr9	63,157,001	63,158,000	1	1	1	2	2	2	1	2	2	2	2	2	2	2	2	1	2	1	2	2	2
chr9:64654001	chr9	64,654,001	64,655,000	1	1	1	2	2	2	2	1	2	2	1	2	2	2	2	2	2	1	2	2	2
chr9:64655001	chr9	64,655,001	64,656,000	1	1	1	2	2	2	2	1	2	2	1	2	2	2	2	2	2	1	2	2	2
chr9:64656001	chr9	64,656,001	64,657,000	1	1	1	2	2	2	2	1	2	2	1	2	2	2	2	2	2	1	2	2	2
chr9:70733001	chr9	70,733,001	70,734,000	1	1	1	2	2	2	2	2	2	2	2	2	2	2	2	2	2	2	2	1	1
chr13:111700001	chr13	111,700,001	111,701,000	1	1	1	1	2	2	2	2	2	1	2	2	2	2	2	2	2	2	1	2	2
chr20:64327001	chr20	64,327,001	64,328,000	1	1	1	2	2	2	2	2	2	2	2	2	2	2	2	2	2	1	1	2	2
chr2:1703001	chr2	1,703,001	1,704,000	0	1	0	2	2	1	2	2	2	2	1	2	2	2	2	2	1	2	2	2	2
chr1:174832001	chr1	174,832,001	174,833,000	3	2	2	3	5	2	2	4	3	2	2	1	1	2	1	1	2	2	2	2	3
chr5:69948001	chr5	69,948,001	69,949,000	2	2	2	3	3	2	3	2	3	2	2	2	2	2	2	1	2	2	1	1	2
chr2:97621001	chr2	97,621,001	97,622,000	0	2	1	2	2	2	4	3	4	2	1	2	2	1	1	2	2	2	2	2	2
chr5:69775001	chr5	69,775,001	69,776,000	1	1	1	2	2	2	3	2	2	1	2	2	2	2	2	1	2	2	2	2	1
chr7:91403001	chr7	91,403,001	91,404,000	0	1	1	1	2	1	1	1	1	2	1	2	2	2	2	1	2	2	2	2	1
chr7:91404001	chr7	91,404,001	91,405,000	0	1	1	1	2	1	1	1	1	2	1	2	2	2	2	1	2	2	2	2	1
chr7:91405001	chr7	91,405,001	91,406,000	0	1	1	1	2	1	1	1	1	2	1	2	2	2	2	1	2	2	2	2	1
chr7:91406001	chr7	91,406,001	91,407,000	0	1	1	1	2	1	1	1	1	2	1	2	2	2	2	1	2	2	2	2	1
chr7:91407001	chr7	91,407,001	91,408,000	0	1	1	1	2	1	1	1	1	2	1	2	2	2	2	1	2	2	2	2	1
chr7:91408001	chr7	91,408,001	91,409,000	0	1	1	1	2	1	1	1	1	2	1	2	2	2	2	1	2	2	2	2	1
chr7:91409001	chr7	91,409,001	91,410,000	0	1	1	1	2	1	1	1	1	2	1	2	2	2	2	1	2	2	2	2	1
chr7:91410001	chr7	91,410,001	91,411,000	0	1	1	1	2	1	1	1	1	2	1	2	2	2	2	1	2	2	2	2	1
chr7:91411001	chr7	91,411,001	91,412,000	0	1	1	1	2	1	1	1	1	2	1	2	2	2	2	1	2	2	2	2	1
chr9:63191001	chr9	63,191,001	63,192,000	1	1	1	2	2	3	2	2	2	2	2	2	1	2	1	2	2	2	1	2	2
chr9:63192001	chr9	63,192,001	63,193,000	1	1	1	2	2	3	2	2	2	2	2	2	1	2	1	2	2	2	1	2	2
chr9:63193001	chr9	63,193,001	63,194,000	1	1	1	2	2	3	2	2	2	2	2	2	1	2	1	2	2	2	1	2	2
chr9:63194001	chr9	63,194,001	63,195,000	1	1	1	2	2	3	2	2	2	2	2	2	1	2	1	2	2	2	1	2	2
chr9:63195001	chr9	63,195,001	63,196,000	1	1	1	2	2	3	2	2	2	2	2	2	1	2	1	2	2	2	1	2	2
chr9:70734001	chr9	70,734,001	70,735,000	1	1	1	2	2	2	2	2	2	2	2	2	2	2	2	2	2	2	1	1	1
chr13:111690001	chr13	111,690,001	111,691,000	1	1	1	2	2	2	3	2	2	2	2	1	2	1	2	2	2	2	1	2	2
chr10:76500001	chr10	76,500,001	76,501,000	1	1	0	2	2	2	2	2	2	2	1	1	2	2	2	2	2	2	1	2	2
chr14:106077001	chr14	106,077,001	106,078,000	1	0	0	2	2	4	2	4	2	2	3	2	0	2	2	0	1	2	2	2	3
chr7:74414001	chr7	74,414,001	74,415,000	2	1	2	0	0	0	0	0	2	1	2	1	2	0	2	3	2	1	2	2	2
chr5:70254001	chr5	70,254,001	70,255,000	1	1	1	2	2	2	2	2	2	2	2	2	1	2	2	1	2	2	1	1	2
chr9:63190001	chr9	63,190,001	63,191,000	1	1	1	2	2	3	2	2	2	2	2	2	1	2	1	2	2	1	1	2	2
chr9:70735001	chr9	70,735,001	70,736,000	1	1	1	2	2	2	2	2	2	2	2	2	2	1	2	2	2	2	1	1	1
chr9:70736001	chr9	70,736,001	70,737,000	1	1	1	2	2	2	2	2	2	2	2	2	2	1	2	2	2	2	1	1	1
chr13:86269001	chr13	86,269,001	86,270,000	1	1	1	2	2	2	2	2	2	2	2	2	2	1	2	1	2	1	1	2	2
chr13:50495001	chr13	50,495,001	50,496,000	1	1	0	2	2	2	2	2	2	1	1	2	2	2	2	2	0	2	2	2	2
chr13:52524001	chr13	52,524,001	52,525,000	1	1	0	2	2	1	2	2	2	1	1	2	1	2	2	2	2	2	2	2	1
chr13:52525001	chr13	52,525,001	52,526,000	1	1	0	2	2	1	2	2	2	1	1	2	1	2	2	2	2	2	2	2	1
chr13:52529001	chr13	52,529,001	52,530,000	1	1	0	2	2	1	2	2	2	1	1	2	1	2	2	2	2	2	2	2	1
chr9:40174001	chr9	40,174,001	40,175,000	3	3	3	3	3	3	1	3	1	2	2	2	1	2	1	1	1	2	2	2	1
chr9:40177001	chr9	40,177,001	40,178,000	3	3	3	3	3	3	1	3	1	2	2	2	1	2	1	1	1	2	2	2	1
chr8:12114001	chr8	12,114,001	12,115,000	1	2	2	3	2	3	3	2	2	2	2	2	1	1	2	2	1	1	2	2	1
chr1:121836001	chr1	121,836,001	121,837,000	1	1	1	2	2	2	2	2	2	2	2	1	1	2	2	1	2	2	1	1	2
chr8:7672001	chr8	7,672,001	7,673,000	1	1	1	2	2	2	2	2	2	2	2	1	1	2	2	2	2	1	1	1	2
chr9:61447001	chr9	61,447,001	61,448,000	1	1	1	2	2	2	2	2	2	1	1	2	2	1	2	2	2	1	1	2	2
chr13:111759001	chr13	111,759,001	111,760,000	1	1	1	2	2	2	2	2	2	2	2	2	1	1	2	1	2	1	2	2	1
chr18:67292001	chr18	67,292,001	67,293,000	0	0	1	2	2	2	1	2	2	1	2	0	1	2	1	2	2	2	2	2	2
chr18:67293001	chr18	67,293,001	67,294,000	0	0	1	2	2	2	1	2	2	1	2	0	1	2	1	2	2	2	2	2	2
chr18:67294001	chr18	67,294,001	67,295,000	0	0	1	2	2	2	1	2	2	1	2	0	1	2	1	2	2	2	2	2	2
chr18:67295001	chr18	67,295,001	67,296,000	0	0	1	2	2	2	1	2	2	1	2	0	1	2	1	2	2	2	2	2	2

Region	CHR	start	end	HD2	HD3	HD1	BN1	BN2	BN3	BN4	BN5	BN6	LC1	LC2	LC3	LC4	LC5	LC6	LC7	LC8	LC9	LC10	LC10*	LC11
chr18:67296001	chr18	67,296,001	67,297,000	0	0	1	2	2	2	1	2	2	1	2	0	1	2	1	2	2	2	2	2	2
chr18:67297001	chr18	67,297,001	67,298,000	0	0	1	2	2	2	1	2	2	1	2	0	1	2	1	2	2	2	2	2	2
chr18:67298001	chr18	67,298,001	67,299,000	0	0	1	2	2	2	1	2	2	1	2	0	1	2	1	2	2	2	2	2	2
chr15:22070001	chr15	22,070,001	22,071,000	3	2	3	2	2	1	1	1	2	2	2	1	1	1	2	2	2	1	1	2	1
chr5:70183001	chr5	70,183,001	70,184,000	1	1	2	2	3	3	3	2	3	1	3	2	1	1	2	1	2	2	1	1	1
chr8:12110001	chr8	12,110,001	12,111,000	1	1	2	3	2	3	3	2	2	2	2	1	1	1	2	2	1	1	2	2	1
chr8:12111001	chr8	12,111,001	12,112,000	1	1	2	3	2	3	3	2	2	2	2	1	1	1	2	2	1	1	2	2	1
chr8:12112001	chr8	12,112,001	12,113,000	1	1	2	3	2	3	3	2	2	2	2	1	1	1	2	2	1	1	2	2	1
chr8:12113001	chr8	12,113,001	12,114,000	1	2	2	3	2	3	3	2	2	2	2	1	1	1	2	2	1	1	2	2	1
chr5:69789001	chr5	69,789,001	69,790,000	1	1	1	1	2	3	3	2	3	1	2	2	1	2	2	1	2	1	2	1	1
chr8:7673001	chr8	7,673,001	7,674,000	1	1	1	2	2	2	2	2	2	2	2	1	1	2	2	1	2	1	1	1	2
chr9:61434001	chr9	61,434,001	61,435,000	1	1	1	2	2	2	2	2	2	1	2	2	2	1	2	1	2	1	1	1	2
chr9:61446001	chr9	61,446,001	61,447,000	1	1	1	2	2	2	2	2	2	1	1	2	1	1	2	2	2	1	1	2	2
chr13:86268001	chr13	86,268,001	86,269,000	1	1	1	2	2	2	2	2	2	1	2	2	1	1	2	1	2	1	1	2	2
chr2:131462001	chr2	131,462,001	131,463,000	2	1	2	2	3	2	3	2	2	2	1	2	2	1	1	1	2	1	1	1	2
chr8:12115001	chr8	12,115,001	12,116,000	2	2	2	3	2	2	3	2	3	2	2	1	1	1	1	2	1	1	2	2	1
chr9:67560001	chr9	67,560,001	67,561,000	3	2	2	2	2	2	2	2	2	1	2	1	1	2	2	2	2	1	1	1	1
chr9:67561001	chr9	67,561,001	67,562,000	3	2	2	2	2	2	2	2	2	1	2	1	1	2	2	2	2	1	1	1	1
chr9:67562001	chr9	67,562,001	67,563,000	3	2	2	2	2	2	2	2	2	1	2	1	1	2	2	2	2	1	1	1	1
chr9:67563001	chr9	67,563,001	67,564,000	3	2	2	2	2	2	2	2	2	1	2	1	1	2	2	2	2	1	1	1	1
chr9:67564001	chr9	67,564,001	67,565,000	3	2	2	2	2	2	2	2	2	1	2	1	1	2	2	2	2	1	1	1	1
chr9:67565001	chr9	67,565,001	67,566,000	3	2	2	2	2	3	2	2	2	1	2	1	1	2	2	2	2	1	1	1	1
chr9:67566001	chr9	67,566,001	67,567,000	3	2	2	2	2	3	2	2	2	1	2	1	1	2	2	2	2	1	1	1	1
chr16:85408001	chr16	85,408,001	85,409,000	1	1	2	2	4	3	3	3	2	2	2	1	1	2	1	1	2	1	1	1	2
chr9:61448001	chr9	61,448,001	61,449,000	1	1	1	2	2	2	2	2	2	1	1	2	1	1	2	2	2	1	1	1	2
chr9:61449001	chr9	61,449,001	61,450,000	1	1	1	2	2	2	2	2	2	1	1	2	1	1	2	2	2	1	1	1	2
chr9:66763001	chr9	66,763,001	66,764,000	2	2	1	2	2	3	2	3	2	1	1	2	1	2	1	2	2	1	1	1	2
chr9:66764001	chr9	66,764,001	66,765,000	2	2	1	2	2	3	2	3	2	1	1	2	1	2	1	2	2	1	1	1	2
chr13:111760001	chr13	111,760,001	111,761,000	1	1	1	2	2	2	2	2	2	2	1	2	1	1	2	1	2	1	1	2	1
chr22:23932001	chr22	23,932,001	23,933,000	2	4	4	5	2	2	5	2	2	2	2	0	0	0	2	2	2	1	2	2	1
chr4:189836001	chr4	189,836,001	189,837,000	1	5	2	5	5	5	4	2	1	1	2	1	1	1	1	1	1	2	2	2	1
chr4:189837001	chr4	189,837,001	189,838,000	1	5	2	5	5	5	4	2	1	1	2	1	1	1	1	1	1	2	2	2	1
chr5:69793001	chr5	69,793,001	69,794,000	1	1	1	1	2	3	3	2	2	1	2	2	2	1	2	1	1	1	1	1	1
chr5:69794001	chr5	69,794,001	69,795,000	1	1	1	2	2	3	3	2	2	1	2	2	2	1	2	1	1	1	1	1	1
chr5:69795001	chr5	69,795,001	69,796,000	1	1	1	1	2	3	3	2	2	1	2	2	2	1	2	1	1	1	1	1	1
chr9:66772001	chr9	66,772,001	66,773,000	1	1	1	2	2	3	2	3	2	1	1	1	2	2	1	3	1	1	1	1	1
chr13:111761001	chr13	111,761,001	111,762,000	1	1	1	2	2	2	2	2	2	2	1	2	1	1	2	1	2	1	1	1	1
chr13:111762001	chr13	111,762,001	111,763,000	1	1	1	2	2	2	2	2	2	2	1	2	1	1	2	1	2	1	1	1	1
chr14:106112001	chr14	106,112,001	106,113,000	1	0	0	2	2	4	2	3	2	2	2	2	0	2	2	0	0	1	2	1	2
chr8:7169001	chr8	7,169,001	7,170,000	2	2	2	1	2	2	3	2	2	2	1	2	1	2	1	1	1	1	1	1	1
chr8:12116001	chr8	12,116,001	12,117,000	2	2	2	3	2	2	3	2	3	2	2	2	0	1	1	2	0	0	2	2	1
chr9:67550001	chr9	67,550,001	67,551,000	3	2	2	2	2	2	1	2	1	1	2	1	1	1	2	1	2	1	1	1	1
chr13:111763001	chr13	111,763,001	111,764,000	1	1	1	2	2	2	2	2	2	1	1	2	1	2	2	1	1	1	1	1	1
chr13:111764001	chr13	111,764,001	111,765,000	1	1	1	2	2	2	2	2	2	1	1	2	1	2	2	1	1	1	1	1	1
chr7:61613001	chr7	61,613,001	61,614,000	2	2	2	0	1	1	1	1	1	2	1	1	1	1	1	2	1	1	1	1	1
chr8:7175001	chr8	7,175,001	7,176,000	1	2	2	1	2	3	3	2	2	1	1	1	1	2	1	1	1	2	1	1	1
chr8:7176001	chr8	7,176,001	7,177,000	1	2	2	1	2	3	3	2	2	1	1	1	1	2	1	1	1	2	1	1	1
chr8:7181001	chr8	7,181,001	7,182,000	1	1	2	2	2	2	3	2	2	1	1	2	1	2	1	1	1	1	1	1	1
chr8:7183001	chr8	7,183,001	7,184,000	2	1	2	2	2	2	3	2	2	1	1	1	1	2	1	1	2	1	1	1	1
chr8:7184001	chr8	7,184,001	7,185,000	2	1	2	2	2	2	3	2	2	1	1	2	1	2	1	1	1	1	1	1	1
chr8:7185001	chr8	7,185,001	7,186,000	2	2	2	1	2	2	3	2	2	1	1	2	1	2	1	1	1	1	1	1	1
chr8:7186001	chr8	7,186,001	7,187,000	1	2	2	1	2	2	3	2	2	1	1	2	1	2	1	1	1	1	1	1	1
chr8:7675001	chr8	7,675,001	7,676,000	1	1	1	2	2	2	2	2	1	1	1	1	1	2	2	1	1	1	1	1	1
chr8:7676001	chr8	7,676,001	7,677,000	1	1	1	2	2	2	2	2	1	1	1	1	1	2	2	1	1	1	1	1	1
chr8:8076001	chr8	8,076,001	8,077,000	1	1	1	2	2	2	3	2	2	1	1	2	2	1	1	1	1	1	1	1	1
chr8:12288001	chr8	12,288,001	12,289,000	1	1	1	1	2	2	2	2	2	1	1	1	1	2	2	1	1	1	1	1	1
chr9:61435001	chr9	61,435,001	61,436,000	1	1	1	2	2	2	2	2	2	1	1	1	2	1	2	1	1	1	1	1	1
chr9:61436001	chr9	61,436,001	61,437,000	1	1	1	2	2	2	2	2	2	1	1	1	2	1	2	1	1	1	1	1	1
chr9:61437001	chr9	61,437,001	61,438,000	1	1	1	2	1	2	2	2	2	1	1	1	2	1	2	1	1	1	1	1	1
chr9:61438001	chr9	61,438,001	61,439,000	1	1	1	2	1	2	2	2	2	1	1	1	2	1	2	1	1	1	1	1	1
chr9:66767001	chr9	66,767,001	66,768,000	1	1	1	2	2	3	1	3	2	1	1	1	1	2	1	2	1	1	1	1	1
chr9:66768001	chr9	66,768,001	66,769,000	1	1	1	2	2	3	1	3	2	1	1	1	1	2	1	2	1	1	1	1	1
chr13:111769001	chr13	111,769,001	111,770,000	1	1	1	1	2	2	2	2	2	1	1	2	1	1	2	1	1	1	1	1	1
chr13:111773001	chr13	111,773,001	111,774,000	1	1	1	1	2	2	2	2	2	1	1	1	1	1	2	1	2	1	1	1	1
chr14:106089001	chr14	106,089,001	106,090,000	1	0	0	1	2	4	2	3	2	1	2	1	0	1	2	1	0	1	1	1	3
chr14:106090001	chr14	106,090,001	106,091,000	1	0	0	1	2	4	2	3	2	1	2	1	0	1	2	1	0	1	1	1	3
chr14:106109001	chr14	106,109,001	106,110,000	1	0	0	1	2	4	2	3	2	2	2	2	0	1	2	0	0	1	1	1	2
chr14:106110001	chr14	106,110,001	106,111,000	1	0	0	1	2	4	2	3	2	2	2	2	0	1	2	0	0	1	1	1	2
chr22:23934001	chr22	23,934,001	23,935,000	2	4	4	5	4	2	5	2	2	0	4	0	0	0	2	2	0	0	2	2	1
chr2:131460001	chr2	131,460,001	131,461,000	3	1	2	2	3	2	3	2	2	2	1	2	2	1	1	1	2	1	0	0	0
chr2:147188001	chr2	147,188,001	147,189,000	2	2	2	2	2	3	2	2	2	1	1	1	1	1	1	0	2	1	2	2	0
chr6:61469001	chr6	61,469,001	61,470,000	2	2	2	2	1	1	1	1	1	1	2	1	1	1	1	1	1	1	1	1	1
chr6:61470001	chr6	61,470,001	61,471,000	2	2	2	2	1	1	1	1	2	1	2	1	1	1	1	1	1	1	1	1	1
chr7:61612001	chr7	61,612,001	61,613,000	2	2	2	1	1	1	1	1	1	2	1	1	1	1	1	1	1	1	1	1	1
chr8:7174001	chr8	7,174,001	7,175,000	1	2	2	1	2	3	3	2	2	1	1	1	1	2	1	1	1	1	1	1	1
chr8:7182001	chr8	7,182,001	7,183,000	1	1	2	2	2	2	3	2	2	1	1	1	1	2	1	1	1	1	1	1	1
chr9:66771001	chr9	66,771,001	66,772,000	1	1	1	2	2	3	1	3	2	1	1	1	1	2	1	1	1	1	1	1	1
chr12:1090001	chr12	1,090,001	1,091,000	0	0	1	1	2	2	2	1	2	1	1	2	1	1	1	1	1	1	1	1	1
chr13:111770001	chr13	111,770,001	111,771,000	1	1	1	1	2	2	2	2	2	1	1	1	1	1	2	1	1	1	1	1	1
chr13:111771001	chr13	111,771,001	111,772,000	1	1	1	1	2	2	2	2	2	1	1	1	1	1	2	1	1	1	1	1	1
chr13:111772001	chr13	111,772,001	111,773,000	1	1	1	1	2	2	2	2	2	1	1	1	1	1	2	1	1	1	1	1	1
chr14:106107001	chr14	106,107,001	106,108,000	1	0	0	1	2	4	2	3	2	2	2	1	0	1	2	0	0	1	1	1	2
chr14:106108001	chr14	106,108,001	106,109,000	1	0	0	1	2	4	2	3	2	2	2	1	0	1	2	0	0	1	1	1	2
chr7:61608001	chr7	61,608,001	61,609,000	2	1	2	1	1	1	1	1	1	1	1	1	1	1	1	1	1	1	1	1	1
chr7:61609001	chr7	61,609,001	61,610,000	2	2	2	1	1	1	1	1	1	1	1	1	1	1	1	1	1	1	1	1	1
chr7:61610001	chr7	61,610,001	61,611,000	2	2	2	1	1	1	1	1	1	1	1	1	1	1	1	1	1	1	1	1	1
chr7:61611001	chr7	61,611,001	61,612,000	2	2	2	1	1	1	1	1	1	1	1	1	1	1	1	1	1	1	1	1	1
chr9:40535001	chr9	40,535,001	40,536,000	1	2	2	1	1	1	1	1	1	1	1	1	1	1	1	1	1	1	1	1	1
chr8:8077001	chr8	8,077,001	8,078,000	1	1	1	1	2	2	3	2	2	1	1	2	1	1	1	1	0	1	1	1	1
chr8:12304001	chr8	12,304,001	12,305,000	1	1	1	1	1	2	2	1	2	1	1	1	1	1	1	1	1	1	1	1	1
chr9:66769001	chr9	66,769,001	66,770,000	1	1	1	2	2	3	1	3	2	1	1	1	1	2	1	1	1	1	1	0	1
chr9:66770001	chr9	66,770,001	66,771,000	1	1	1	2	2	3	1	3	2	1	1	1	1	2	1	1	1	1	1	0	1
chr8:12074001	chr8	12,074,001	12,075,000	1	0	0	1	1	1	1	1	1	1	1	1	1	1	1	1	1	1	1	1	1
chr14:106099001	chr14	106,099,001	106,100,000	1	0	0	1	2	4	2	3	2	1	2	1	0	1	2	0	0	1	1	1	2
chr14:106100001	chr14	106,100,001	106,101,000	1	0	0	1	2	4	2	3	2	1	2	1	0	1	2	0	0	1	1	1	2
chr14:106101001	chr14	106,101,001	106,102,000	1	0	0	1	2	4	2	3	2	1	2	1	0	1	2	0	0	1	1	1	2
chr14:106102001	chr14	106,102,001	106,103,000	1	0	0	1	2	4	2	3	2	1	2	1	0	1	2	0	0	1	1	1	2
chr14:106103001	chr14	106,103,001	106,104,000	1	0	0	1	2	4	2	3	2	1	2	1	0	1	2	0	0	1	1	1	2
chr14:106104001	chr14	106,104,001	106,105,000	1	0	0	1	2	4	2	3	2	1	2	1	0	1	2	0	0	1	1	1	2
chr14:106105001	chr14	106,105,001	106,106,000	1	0	0	1	2	4	2	3	2	1	2	1	0	1	2	0	0	1	1	1	2
chr14:106106001	chr14	106,106,001	106,107,000	1	0	0	1	2	4	2	3	2	1	2	1	0	1	2	0	0	1	1	1	2
chr2:131461001	chr2	131,461,001	131,462,000	3	1	2	2	3	2	3	2	2	2	1	2	2	0	1	1	2	0	0	0	0
chr16:11590001	chr16	11,590,001	11,591,000	2	2	2	3	2	2	2	2	2	0	2	2	0	0	0	2	2	2	0	0	0
chr12:1089001	chr12	1,089,001	1,090,000	0	0	0	1	2	2	2	1	1	1	1	1	1	1	1	1	0	1	0	1	1
chr4:10212001	chr4	10,212,001	10,213,000	0	1	1	1	3	3	2	3	1	1	0	0	1	1	2	1	1	1	0	0	1
chr4:10213001	chr4	10,213,001	10,214,000	0	1	1	1	3	3	2	3	1	1	0	0	1	1	2	1	1	1	0	0	1
chr4:10214001	chr4	10,214,001	10,215,000	0	1	1	1	3	3	2	3	1	1	0	0	1	1	2	1	1	1	0	0	1
chr4:10215001	chr4	10,215,001	10,216,000	0	1	1	1	3	3	2	3	1	1	0	0	1	1	2	1	1	1	0	0	1
chr4:10216001	chr4	10,216,001	10,217,000	0	1	1	1	3	3	2	3	1	1	0	0	1	1	2	1	1	1	0	0	1
chr4:10217001	chr4	10,217,001	10,218,000	0	1	1	1	3	3	2	3	1	1	0	0	1	1	2	1	1	1	0	0	1
chr4:10218001	chr4	10,218,001	10,219,000	0	1	1	1	3	3	2	3	1	1	0	0	1	1	2	1	1	1	0	0	1
chr4:10219001	chr4	10,219,001	10,220,000	0	1	1	1	3	3	2	3	2	1	0	0	1	1	2	1	1	1	0	0	1
chr4:10220001	chr4	10,220,001	10,221,000	0	1	1	1	3	3	2	3	2	1	0	0	1	1	2	1	1	1	0	0	1

**Region**	**CHR**	**start**	**end**	HD2	HD3	HD1	BN1	BN2	BN3	BN4	BN5	BN6	LC1	LC2	LC3	LC4	LC5	LC6	LC7	LC8	LC9	LC10	LC10*	LC11
chr4:10221001	chr4	10,221,001	10,222,000	0	1	1	1	3	3	2	3	1	1	0	0	1	1	2	1	1	1	0	0	1
chr4:10222001	chr4	10,222,001	10,223,000	0	1	1	1	3	3	2	3	1	1	0	0	1	1	2	1	1	1	0	0	1
chr4:10223001	chr4	10,223,001	10,224,000	0	1	1	1	3	3	2	3	1	1	0	0	1	1	2	1	1	1	0	0	1
chr4:10224001	chr4	10,224,001	10,225,000	0	1	1	1	3	3	2	3	1	1	0	0	1	1	2	1	1	1	0	0	1
chr4:10225001	chr4	10,225,001	10,226,000	0	1	1	1	3	3	2	3	1	1	0	0	1	1	2	1	1	1	0	0	1
chr4:10226001	chr4	10,226,001	10,227,000	0	1	1	1	2	3	2	3	1	1	0	0	1	1	2	1	1	1	0	0	1
chr4:10227001	chr4	10,227,001	10,228,000	0	1	1	1	2	3	2	3	1	1	0	0	1	1	2	1	1	1	0	0	1
chr22:23935001	chr22	23,935,001	23,936,000	0	0	0	5	4	2	5	0	2	0	0	1	0	0	2	2	0	0	2	2	0
chr4:10228001	chr4	10,228,001	10,229,000	0	1	1	1	2	3	2	3	1	1	0	0	1	1	1	1	1	1	0	0	1
chr4:10229001	chr4	10,229,001	10,230,000	0	1	1	1	2	3	2	3	1	1	0	0	1	1	1	1	1	1	0	0	1
chr4:10230001	chr4	10,230,001	10,231,000	0	1	1	1	2	2	2	2	1	1	0	0	1	1	1	1	1	1	0	0	1
chr4:10231001	chr4	10,231,001	10,232,000	0	1	1	1	2	2	2	2	1	1	0	0	1	1	1	1	1	1	0	0	1
chr1:105474001	chr1	105,474,001	105,475,000	0	2	0	2	3	2	2	3	2	0	1	0	2	2	2	0	1	0	0	0	0
chr1:105475001	chr1	105,475,001	105,476,000	0	2	0	2	3	2	2	3	2	0	1	0	2	2	2	0	1	0	0	0	0
chr1:105477001	chr1	105,477,001	105,478,000	0	2	0	2	3	2	2	3	2	0	1	0	1	2	2	0	1	0	0	0	0
chr8:1399001	chr8	1,399,001	1,400,000	0	0	0	1	1	1	1	1	1	0	0	0	1	0	1	1	1	1	0	0	1
chr2:125686001	chr2	125,686,001	125,687,000	1	1	2	0	0	0	0	0	0	0	1	0	0	0	0	0	0	0	1	1	0
chr2:125687001	chr2	125,687,001	125,688,000	1	1	2	0	0	0	0	0	0	0	1	0	0	0	0	0	0	0	1	1	0
chr2:125688001	chr2	125,688,001	125,689,000	1	1	2	0	0	0	0	0	0	0	1	0	0	0	0	0	0	0	1	1	0
chr2:125689001	chr2	125,689,001	125,690,000	1	1	1	0	0	0	0	0	0	0	1	0	0	0	0	0	0	0	1	1	0
chr5:58028001	chr5	58,028,001	58,029,000	2	2	0	0	0	0	0	0	0	0	0	0	0	0	0	0	0	0	1	1	0
chr8:1395001	chr8	1,395,001	1,396,000	0	0	1	1	0	1	1	1	1	0	0	0	0	0	0	0	0	1	0	0	0
chr12:8417001	chr12	8,417,001	8,418,000	0	0	0	0	1	1	1	1	1	0	0	0	0	0	0	0	0	0	0	0	0

Supplemental Table 3. Clinical samples were used for the detection of mutations by NGS method

Sample ID	Institution	type	stage	TNM	other info.	mutation site	tissue NGS%	RBC NGS%	tissue IHC	tissue NGS(3rd party)	ctDNA(3rd party)
LC44	GL	LUAD	IA2	T1b,N0,cM0	N/A	EGFR 19del	15%(+)	0.09%(+)	+	N/A	N/A
						EGFR L858R	0%	0.00%	-		
						KRAS	4% (+ G12S)	0.00%	N/A		
LC45	GL	LUAD	IA2	T1b,N0,cM0	N/A	EGFR 19del	26%	0.10%(+)	+	N/A	N/A
						EGFR L858R	0%	0.00%	-		
						KRAS	0.9%	0.00%	N/A		
LC46	GL	LUAD	IIIA	T2a,N2,cM0	N/A	EGFR 19del	0%	0.00%	-	N/A	N/A
						EGFR L858R	45%(+)	0.32%(+)	+		
						KRAS	0%	0.02%	N/A		
LC47	GL	LUAD	IA1	T1mi,N0,cM0	in situ	EGFR 19del	0%	0.00%	-	N/A	N/A
						EGFR L858R	0%	0.00%	-		
						KRAS	0%	0.19%(+ G12S)	N/A		
LC48	GL	LUAD	IB	T2a,N0,cM0	N/A	EGFR 19del	0%	0.00%	-	N/A	N/A
						EGFR L858R	0%	0.00%	-		
						KRAS	0%	0.05%	N/A		
LC49	GL	LUSC	IIB	T3,N0,cM0	N/A	EGFR 19del	0%	0.00%	-	N/A	N/A
						EGFR L858R	0%	0.01%	-		
						KRAS	0%	0.01%	N/A		
LC50	GL	LUAD	IA3	T1c,N0,Mx	N/A	EGFR 19del	0%	0.00%	-	N/A	N/A
						EGFR L858R	0%	0.02%	-		
						KRAS	0.6%	0.06%	N/A		
LC51	GL	LUSC	IIB	T1c,N1,cM0	N/A	EGFR 19del	0%	0.00%	-	N/A	N/A
						EGFR L858R	0%	0.01%	-		
						KRAS	0%	1.67%(+ G13D)	N/A		
LC52	GL	LUAD	IB	T2a,N0,cM0	N/A	EGFR 19del	0%	0.00%	-	N/A	N/A
						EGFR L858R	0%	0.67%(+)	+		
						KRAS	0%	0.00%	N/A		
LC53	GL	LUAD	IA3	T1c,N1,cM0	N/A	EGFR 19del	28%(+)	9.60%(+)	N/A	N/A	N/A
						EGFR L858R	0%	0.00%			
						KRAS	0%	0.05%			
LC54	GL	LUAD	IB	T2a,N0,cM0	N/A	EGFR 19del	31%(+)	0.10%(+)	+	N/A	N/A
						EGFR L858R	0%	0.05%	-		
						KRAS	0%	0.01%	N/A		
LC55	GL	LUAD	IA2	T1b,N0,cM0	N/A	EGFR 19del	0%	0.00%	-	N/A	N/A
						EGFR L858R	34%(+)	0.16%(+)	+		
						KRAS	0%	0.01%	N/A		
LC56	GL	LUSC	IIIB	T3,N2,cM0	N/A	EGFR 19del	0%	0.00%	-	N/A	N/A
						EGFR L858R	0%	5.01%(+)	+		
						KRAS	0%	0.06%	N/A		
LC57	XH	LUAD	IA1	T1a,N0,M0	N/A	EGFR 19del	0%	0.01%(+)	N/A	+	-
						EGFR L858R	0%	0.01%		-	-
						KRAS	0%	0.00%		-	-
LC58	XH	LUAD	IA1	T1a,N0,M0	N/A	EGFR 19del	0%	0.00%	N/A	-	-
						EGFR L858R	0%	1.07%(+)		-	-
						KRAS	0%	0.02%		-	-
LC59	XH	LUAD	IB	T2a,N0,M0	N/A	EGFR 19del	0%	0.00%	N/A	-	N/A
						EGFR L858R	24%(+)	0.25%(+)		-	
LC60	XH	LUAD	IA2	T1b,N0,M0	Multiple Primary	EGFR 19del	0%	1.23%(+)	N/A	N/A	N/A
						EGFR L858R	15%(+)	0.75%(+)			
LC61	XH	LUAD	IA2	T1b,N0,M0	N/A	EGFR 19del	0%	0.00%	N/A	N/A	N/A
						EGFR L858R	7%(+)	0.07%(+)			
LC62	XH	LUAD	IB	T2a,N0,M0	Multiple Primary	EGFR 19del	0%	0.00%	N/A	-	-
						EGFR L858R	10%(+)	0.78%(+)		+	-
LC63	XH	LUAD	IA3	T1c,N1,M0	N/A	EGFR 19del	0%	0.00%	N/A	-	-
						EGFR L858R	0%	2.83%(+)		-	-
						KRAS	36% (+ G12A	0.37%(+ G12A)		+	-
LC64	XH	LUAD	IA2	T1b,N0,M0	N/A	EGFR 19del	0%	1.92%(+)	N/A	N/A	N/A
						EGFR L858R	0%	0.00%			
						KRAS	0%	0.21%(+ G12S)			
LC65	XH	LUAD	IIIA	T1b,N2,M0	N/A	EGFR 19del	36%(+)	0.36%(+)	N/A	+	-
						EGFR L858R	0%	0.00%		-	-
LC66	XH	LUAD	IA2	T1b,N0,M0	Multiple Primary	EGFR 19del	0%	0.00%	N/A	-	-
						EGFR L858R	10%(+)	2.25%(+)		+	-
LC67	XH	LUAD	IA1	T1a,N0,M1	N/A	EGFR 19del	0%	0.00%	N/A	N/A	N/A
						EGFR L858R	9%(+)	0.10%(+)			
LC68	XH	LUAD	IA2	T1b,N0,M0	Multiple Primary	EGFR 19del	25%(+)	0.21%(+)	N/A	+	-
						EGFR L858R	0%	1.28%(+)		+	-
LC69	XH	LUAD	IB	T2a,N0,M0	Multiple Primary	EGFR 19del	0%	7.98%(+)	N/A	-	-
						EGFR L858R	0%	0.00%		-	-

Because mutation detection results are affected by sampling bias of cancer tissues, we determine the mutation results of cancer tissues by combining our NGS method, hospital IHC method or NGS test results from third-party testing organizations (3rd party). LUAD: Lung adenocarcinoma; LUSC: Lung squamous carcinoma; XH: Peking Union Medical College Hospital; GL: Nanjing Drum Tower Hospital.

Supplemental Table 4. Clinical samples were used for detection of *EGFR* L858R mutation by ddPCR

Sample ID	Institution	Type	Stage	TNM	Tissue NGS	RBC ddPCR	Tissue IHC	Sample type		Positives	Negatives	Ch1+Ch2+	Ch1+Ch2-	Ch1-Ch2+	Ch1-Ch2-	Accepted Droplets	Reads
LC70	GL	LUAD	IA2	T1b,Nx,cM0	30%	0.000%	+	PCR product	FN	0	18340	0	0	8796	9544	18340	8796
LC56	GL	LUSC	IIIB	T3,N2,cM0	0%	0.052%	+		TP	10	18041	4	6	11828	6213	18051	11838
LC71	GL	LUAD	IA2	T1b,N0,cM0	0%	0.000%	-		TN	0	17847	0	0	11920	5927	17847	11920
LC72	GL	LUAD	IIA	T2b,N0,cM0	40%	0.004%	+		TP	2	19148	0	2	17594	1554	19150	17596
LC73	GL	LUAD	IB	T2a,N0,cM0	22%	0.018%	+		TP	4	18927	1	3	12940	5987	18931	12944
LC74	GL	LUAD	IA1	T1mi,Nx,cM0	68%	0.068%	+		TP	13	20887	5	8	12571	8316	20900	12584
LC75	GL	LUAD	IA2	T1b,N0,cM0	0%	0.000%	-	original DNA	TN	0	16657	0	0	7329	9328	16657	7329
LC56	GL	LUSC	IIIB	T3,N2,cM0	0%	0.042%	+		TP	4	18006	3	1	7372	10634	18010	7376
LC73	GL	LUAD	IB	T2a,N0,cM0	22%	0.020%	+		TP	2	12063	1	1	6825	5238	12065	6827
LC76	GL	LUAD	IB	T2a,N0,cM0	54%	0.071%	+		TP	6	14914	4	2	6453	8461	14920	6459
LC77	GL	LUAD	IA3	T1c,N0,cM0	19%	0.063%	+		TP	5	16075	2	3	6238	9837	16080	6243

LUAD: Lung adenocarcinoma; LUSC: Lung squamous carcinoma. GL: Nanjing Drum Tower Hospital.

Ch1: FAM signal, representing the *EGFR* L858R mutation
Ch2: HEX signal, representing the wild type

Supplemental Table 5. Primers used in this study

Name	Sequence(5′→3′)	Purpose
EGFR-19del-F	ACTCTGGATCCCAGAAGGTGA	Amplification of *EGFR* 19del for sequencing
EGFR-19del-R	ACCCACTAGAGCTAGAAAGGGA	
EGFR-T790M-F	ATGGCCAGCGTGGACAAC	Amplification of *EGFR* T790M for sequencing
EGFR-T790M-R	CTCTTGCTATCCCAGGAGCG	
EGFR-L858R-F	AGCCAGGAACGTACTGGTGA	Amplification of *EGFR* L858R for sequencing or ddPCR; *EGFR* gene copy number detection by qPCR
EGFR-L858R-R	ACCCAGAATGTCTGGAGAGC	
KRAS-G12/13-F	TAAGGCCTGCTGAAAATGACTG	Amplification of *KRAS* G12/13 for sequencing
KRAS-G12/13-R	TACTCATGAAAATGGTCAGAGAAAC	
GAPDH-F	GACAACTCTTTTCATCTTCTAGGTA	*GAPDH* gene copy number detection by qPCR
GAPDH-R	GGTTGAGCACAGGGTACTTTA	
MT-CO1-F	TGATCTGCTGCAGTGCTCTGA	mitochondrially encoded *CO1* gene copy number detection by qPCR*
MT-CO1-R	TCAGGCCACCTACGGTGAA	

* Primer reference to other papers
